# Enlarged External Occipital Protuberance in young French individuals’ head CT: stability in prevalence, size and type between 2011 and 2019

**DOI:** 10.1038/s41598-020-63554-y

**Published:** 2020-04-16

**Authors:** Thibaut Jacques, Alexandre Jaouen, Grégory Kuchcinski, Sammy Badr, Xavier Demondion, Anne Cotten

**Affiliations:** 1 CHU Lille, Department of Musculoskeletal Radiology, F-59000 Lille, France; 2 Univ. Lille, Lille School of Medicine, F-59000 Lille, France; 3 CHU Lille, Department of Neuroradiology, F-59000 Lille, France; 4 Univ. Lille, Laboratory of Anatomy, F-59000 Lille, France

**Keywords:** Musculoskeletal system, Medical imaging

## Abstract

External Occipital Protuberance (EOP) enlargement has been recently reported to increase in young adults, with a putative link with postural factors such as the use of smartphones. This study aims to analyze finely the changes in prevalence and size of EOP enlargement in *millennials*, throughout the smartphone era (2011 – 2019). Anonymized head Computerized Tomography (CT) examinations from patients aged 18-30 in 2011 (n = 205) or 2019 (n = 240), were reviewed to assess the type of EOP and to measure its volume in case of enlargement. Additional CT analyses were performed on two ancient skulls, from a XVI^th^ century young male and a young female Egyptian mummy. There was no significant evolution in the prevalence of EOP enlargement between 2011 (92/205, 44.9%) and 2019 (106/240; 44.2%) (P = 0.92). There was no significant evolution either in the distribution of enlarged EOP volumes (P = 0.14) or of EOP types (P = 0.92) between 2011 and 2019. In the meantime, rates of smartphone ownership in *millennials* rose from 35% to 98%. Compared to 2019 volumes, the Egyptian mummy displayed an EOP enlargement corresponding to the 85^th^ percentile for young women, and the XVI^th^ century skull to the 73^rd^ percentile for young men. In conclusion, on a population scale, prevalence and volume of enlarged EOP in *millennials* remain stable between 2011 and 2019, which makes the impact of rapidly growing modern environmental factors on EOP changes unlikely. EOP enlargement was also already present in ancient skulls from young individuals, with measurements within today’s upper ranges.

## Introduction

External Occipital Protuberance (EOP) is a normal anatomical structure located on the posterior surface of the occipital bone, at the level of the superior nuchal line. It is the insertion site of the nuchal ligament^1^. There are three types of EOP depending on its shape^2,3^: “flat type” (type 1), “crest type” (type 2) and “spine type” (type 3). Previous studies have shown that type 1 was more frequent in women and type 3 more frequent in men^2,4^. Type 3 can sometimes manifest as a subcutaneous scalp pseudotumor^5^.

Even if the EOP is widely studied in the anthropological literature, medical publications remain rare on this topic^6^. EOP enlargement (sometimes referred to as “occipital spur”) is defined as a bony outgrowth of the EOP, and is usually considered as an anatomic variant^7^. While common and frequently asymptomatic^6^, it can be the source of discomfort^5,6,8^ and sometimes be painful enough to require surgical excision^3^ or rarely fracture after a trauma^9^.

Recent studies have reported that the prevalence of EOP enlargement seemed to be rapidly increasing in the young adult population^4,10^ with a possible role of mechanical factors^11^ such as sustained forward head protraction^10^, and raise the question of musculoskeletal disorders related to environmental factors among *millennials*. The hypothesis of a possible link with smartphone use has been suggested, however without clear link established so far, due to an important number of confounding factors.

Nevertheless, a true modification in enlarged EOP prevalence and size in *millennials* would suggest a significant evolutionary pressure over a short period of time. Moreover, these previous reports^4,10^ were based on the analysis of X-ray examinations, which is an imperfect method to assess a three-dimensional structure. Finally, no longitudinal data have ever been published on this topic so far. Therefore, further analyses are needed to better understand the potential changes in EOP size over time.

The aim of this study was to compare the prevalence and size of EOP enlargement in 2 populations of young adults, from the present time (2019) and from the beginning of the smartphone era (2011), to evaluate whether or not any change could be observed in this period of time. The secondary objective was to assess the presence of EOP enlargement in ancient skulls from two young individuals (XVI^th^ century France and Ptolemaic Egypt).

## Methods

### Population

This retrospective study was conducted in the Emergency Radiology Department of a single University Hospital (Lille University Hospital, Lille, France). Anonymized head Computerized Tomography (CT) examinations from young patients aged from 18 to 30 years, who were referred to the Division for an acute event between January and June, 2011, and between January and June, 2019, were retrospectively analyzed.

The anonymization process retained only age, gender and year of examination (2011 or 2019).

All examinations from both periods were performed on the same CT device providing 0.6 mm slices (Somatom Definition AS, Siemens Healthcare GmbH).

### EOP measurements

**Figure 1:**
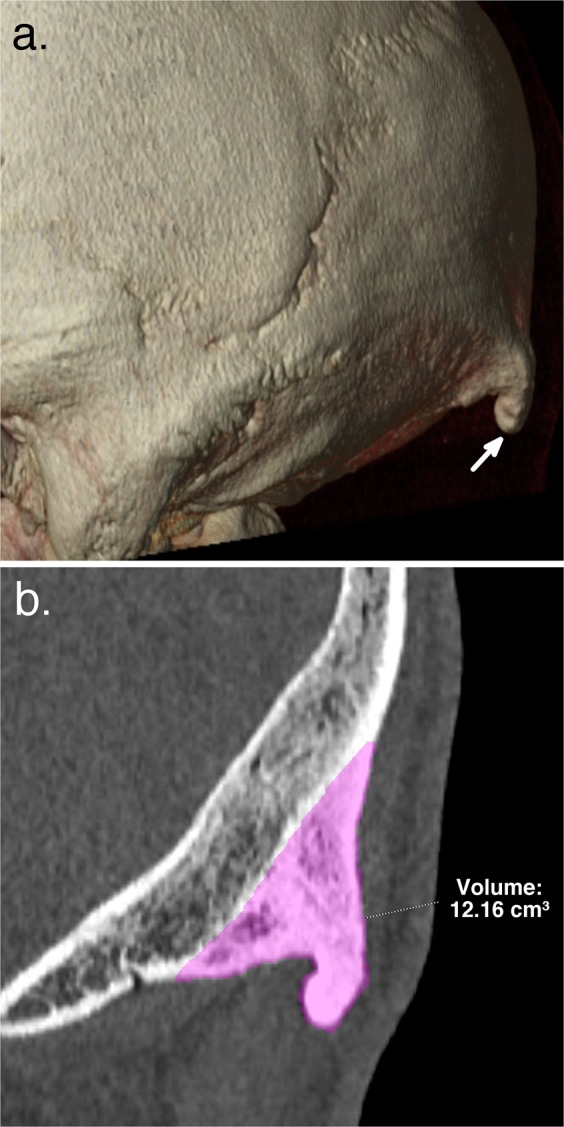
EOP measurement method. Panel a. shows volume rendering (VR) of the skull CT with a type 3 EOP (arrow) in a male patient from the 2019 dataset; Panel b. shows the corresponding measurement technique of the volume of the EOP, using the “Freehand V.O.I.” tool (SyngoVia). This EOP enlargement was the largest among all examinations, with a volume of 12.16 cm^3^.

The anonymized head CT examinations were reviewed retrospectively by two readers: a senior musculoskeletal radiologist and a radiology resident specializing in musculoskeletal imaging. The readers were blinded for the gender and age of patients, as well as for the year of examination. The CT examinations were visualized with SyngoVia software (Siemens Healthcare GmbH), using multiplanar reconstruction with a bone window. The type of EOP was graded in consensus between readers: type 1 (“flat type”), type 2 (“crest type”) or type 3 (“spine type”). Type 2 and type 3 EOP were considered as enlarged, and their volume was then measured manually, in consensus between readers, using the “Freehand Volume of Interest (V.O.I)” tool from SyngoVia. The size was expressed as a volume in cm^3^ (Fig. 1). Measurement data are provided as Supplementary Information files.

### Ancient skulls analysis

In order to obtain further insights on EOP over a longer period of time, CT analyses were performed on two skulls from young adults of ancient times.

The first analysis was performed on an Egyptian mummy (ML4, Natural History Museum, Lille, France) excavated in the early XIX^th^ century from the tombs of Thebes, Egypt, and dated of the Ptolemaic (332 - 30 BC) or the Graeco-Roman (30 BC - 641 AD) periods^12,13^. According to previous local works^12,13^, this mummy was a female young adult aged 25 +/− 4.9 years old. It underwent a whole body CT, including skull, in our institution for archaeological purposes in June 2014. Images were analyzed retrospectively for the purpose of this study.

The second analysis was performed on a dry skull (Laboratory of Anatomy, Lille University, Lille, France) from a skeleton excavated in 1989 in the vault of a Middle-Ages convent from the Dominican Order established in the XIII^th^ century (Valenciennes, France). Previous unpublished work on the full skeleton estimated that it belonged to a young male from *circa* XVI^th^ century, aged approximately 30 years old. The skull underwent a dedicated CT for the purpose of this study.

The images from both skulls were analyzed exactly as described in the previous section.

### Use of smartphones

Since this study was performed on anonymized CT examinations and no personal information were available, it was thus impossible to quantify directly the individual use of smartphones for each patient. To obtain indirect information on the evolution of the use of smartphone use in young patients during the time period between the two datasets, we used publicly available official data summarizing the use of these devices, from 2011 to 2019 and by subgroups of age^14^.

### Ethics

All analyses were performed retrospectively on anonymized data, and were compliant to the EU General Data Protection Regulation (GDPR) and the declaration of Helsinki. Our institutional review board approved the data analysis (Department of Numeric Resources, University Hospital of Lille, reference DEC19-279) and waived the requirement for an informed patient consent, given the design of the study (retrospective analysis on anonymized examinations). The authorization for the analysis of the two ancient skulls was provided by their respective right-holders.

### Statistical analysis

Statistical analyses and graphical plotting were performed using GraphPad Prism software version 8.0.1 for Mac OS X (GraphPad Software). Quantitative variables are expressed as mean +/− standard deviation (SD). Comparisons between quantitative variables were performed using Kolmogorov-Smirnov test. Qualitative variables are expressed as raw numbers, proportions and percentages. Comparisons between qualitative variables were performed using Chi-square test. Statistical significance was set at P < 0.05.

## Results

### Population

The dataset was composed of 205 anonymous head CT examinations for the 2011 period, and 240 for the 2019 period. All 445 examinations came from different patients, without visible condition affecting the occipital bone (fracture or osteolysis for example). The 2011 dataset was composed of 87 women (87/205; 42.4%) and 118 men (118/205; 57.6%). The 2019 dataset was composed of 106 women (106/240; 44.2%) and 134 men (134/240; 55.8%). There was no significant difference in the repartition of genders between the two periods (P = 0.71). The mean age of patients was 23.9 +/− 3.6 years for the 2011 period and 23.4 +/− 3.7 years for the 2019 period (Table 1). The birth years ranged from 1980 to 1993 for the 2011 dataset, and from 1988 to 2001 for the 2019 dataset, which corresponds to the usual birth years of individuals referred to as “millennials”^15^.

**Table 1:** Detail of the study datasets from 2011 and 2019, in terms of gender and age.

Number of patients	2011	2019
	205	240
Women	87 (42.4%)	106 (44.2%)
Men	118 (57.6%)	134 (55.8%)
**Mean age** +**/**− **SD (years)**	**23**.**9** +**/− 3**.**6**	**23**.**4** +/− **3**.**7**
Women	23.3 +/− 3.8	23.3 +/− 3.9
Men	24.4 +/− 3.4	23.4 +/− 3.5

### EOP type and volume in the 2011 and 2019 CT datasets

**Figure 2:**
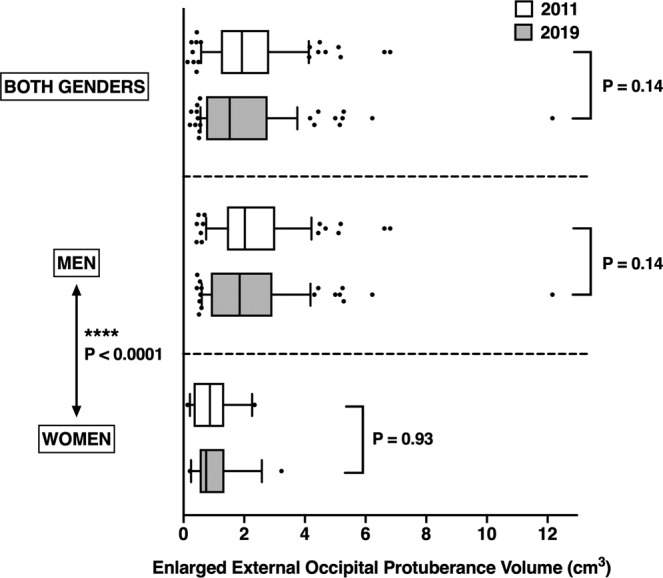
Distribution of EOP volumes in patients with an enlarged EOP, depending on the year and gender (boxplots). Boxes extend from 25^th^ to 75^th^ percentiles; whiskers extend from 10^th^ to 90^th^ percentiles; median value is plotted as a vertical bar. P-values were obtained using Kolmogorov-Smirnov test. Outlying points were included in the analysis.

Regarding the volume of EOP enlargement, there was no significant evolution between the 2011 and 2019 datasets regarding the distribution of EOP measurements values (2.13 +/−1.36 cm^3^ versus 2.00 +/−1.66 cm^3^ respectively, Kolmogorov-Smirnov D = 0.16; P = 0.14) (Fig. 2).

In men with EOP enlargement, the mean volume was 2.34 +/−1.35 cm^3^ in the 2011 dataset versus 2.19 +/−1.72 cm^3^ in the 2019 dataset, without significant difference in the distribution of values (Kolmogorov-Smirnov D = 0.18; P = 0.14). In women with EOP enlargement, the mean volume was 1.05 +/−0.73 cm^3^ in the 2011 dataset versus 1.05 +/−0.79 cm^3^ in the 2019 dataset, without significant difference in the distribution of values (Kolmogorov-Smirnov D = 0.19; P = 0.93) (Fig. 2). The distribution of values were significantly different between men and women, with men having significantly higher EOP volumes than women (P < 0.0001) (Fig. 2).

Regarding the prevalence of EOP enlargement, in the 2011 dataset, 92 examinations (92/205, 44.9%) showed an EOP enlargement, being either type 2 (64/205, 31.2%) or type 3 EOP (28/205, 13.7%). In the 2019 dataset, 106 cases (106/240, 44.2%) showed an EOP enlargement, being either type 2 (71/240, 29.6%) or type 3 EOP (35/240, 14.6%).There was thus no significant evolution between the 2011 and 2019 datasets regarding the prevalence of EOP enlargement (P = 0.92) (Table 2).

**Table 2:** Evolution of the repartition of EOP types between 2011 and 2019 datasets, by year and gender.

	2011	2019	P-value (Χ^2^, df)
All patients	N = 205	N = 240	
Type 1 EOP (“flat type”) Type 2 EOP (“crest type”) Type 3 EOP (“spine type”)	55.1% (N = 113) 31.2% (N = 64) 13.7% (N = 28)	55.8% (N = 134) 29.6% (N = 71) 14.6% (N = 35)	0.92 (0.17, 2)
**Women**	**N** = **87**	**N** = **106**	0.99 (0.01, 2)
Type 1 EOP Type 2 EOP Type 3 EOP	82.8% (N = 72) 10.3% (N = 9) 6.9% (N = 6)	83.0% (N = 88) 10.4% (N = 11) 6.6% (N = 7)	
**Men**	**N** = **118**	**N** = **134**	0.90 (0.21, 2)
Type 1 EOP Type 2 EOP Type 3 EOP	34.7% (N = 41) 46.6% (N = 55) 18.7% (N = 22)	34.3% (N = 46) 44.8% (N = 60) 20.9% (N = 28)	

In the 2011 dataset, EOP enlargement was common in men since it was seen in 77 cases (77/118, 65.3%) whereas it was present in only 15 women (15/87, 17.2%). The results were similar in the 2019 dataset, with EOP enlargement being seen in 88 cases (88/134, 65.7%) for men, versus 18 cases (18/106, 17.0%) for women (Table 2).

When pooling both datasets from 2011 and 2019 regarding EOP shape by gender, 160 women (160/193, 82.9%) displayed a type 1 EOP, 20 women (20/193, 10.4%) a type 2 EOP and 13 women (13/193, 6.7%) a type 3 EOP. These results were significantly different (p < 0.0001) from the findings seen in men, with respectively 87 cases (87/252, 34.5%), 115 cases (115/252, 45.7%) and 50 cases (50/252, 19.8%) (Table 3).

**Table 3:** Overall repartition of EOP types by gender (pooled 2011 and 2019 datasets).

	Women	Men	P-value (Χ^2^, df)
	N = 193	N = 252	
Type 1 EOP Type 2 EOP Type 3 EOP	82.9% (N = 160) 10.4% (N = 20) 6.7% (N = 13)	34.5% (N = 87) 45.7% (N = 115) 19.8% (N = 50)	< 0.0001 (104.2, 2)

### EOP measurement in ancient skulls

**Figure 3:**
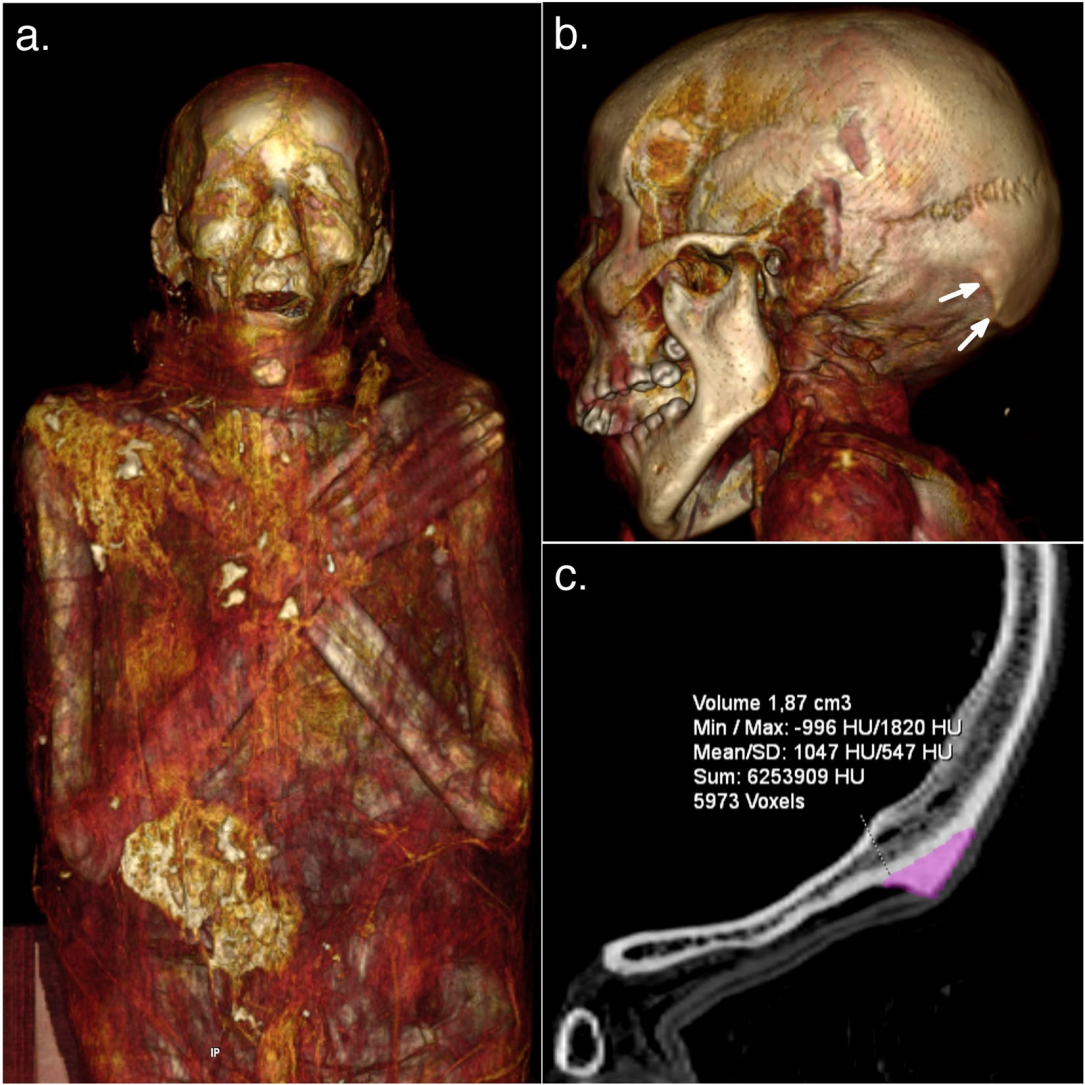
CT analysis of a young female Egyptian mummy. Panel a. shows an anterior frontal Volume Rendering (VR) view of the mummy’s head and trunk. Panel b. shows a postero-lateral VR view of the skull with visible enlarged type 2 EOP (arrows). Panel c. shows EOP measurement (1.87 cm^3^) which corresponds to the 85^th^ percentile of 2019 values for young women with EOP enlargement.

The CT of the female Egyptian mummy showed a type 2 EOP enlargement, with a volume of 1.87 cm^3^ (Fig. 3). This value corresponds to the 85^th^ percentile of the 2019 dataset for young women with EOP enlargement.

**Figure 4:**
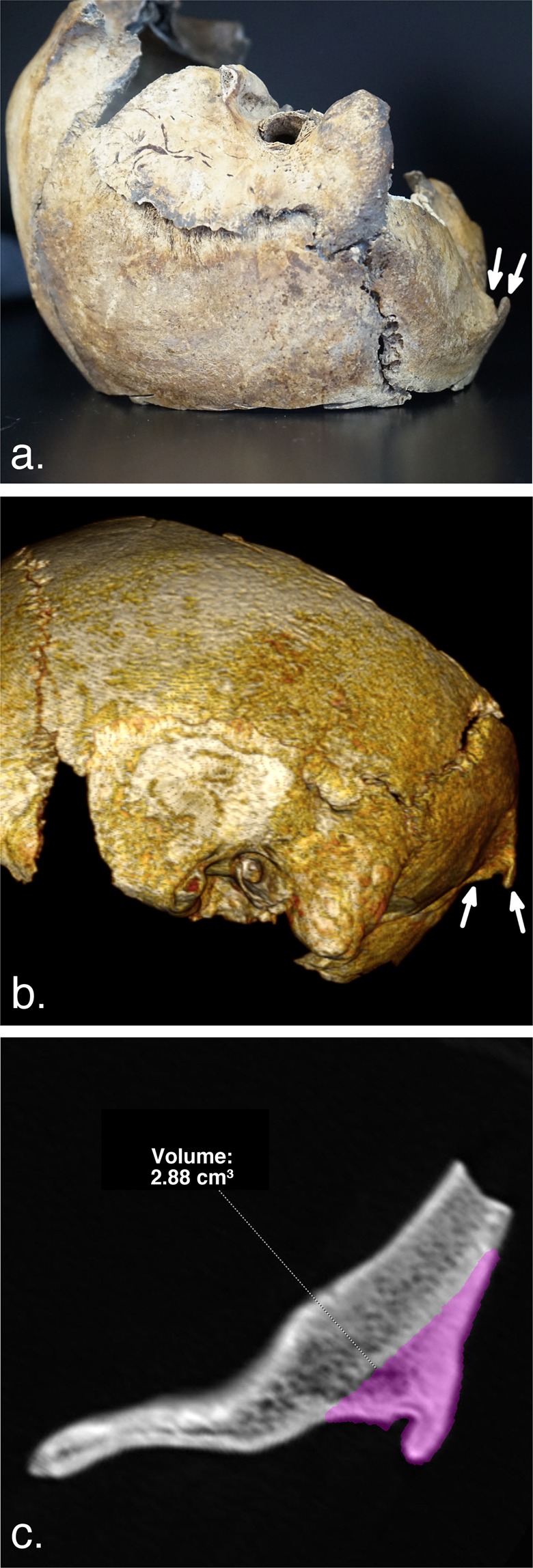
CT analysis of a young male’s skull from *circa* XVI^th^ century. Panel a. shows a lateral photograph of the skull, with visible enlarged type 3 EOP (arrows). Panel b. shows a lateral VR view of the skull with corresponding EOP (arrows). Panel c. shows EOP measurement (2.88 cm^3^), which corresponds to the 73^rd^ percentile of 2019 values for young men with EOP enlargement.

The CT of the XVI^th^ century male skull showed a type 3 EOP enlargement, with a volume of 2.88 cm^3^ (Fig. 4). This value corresponds to the 73^rd^ percentile of the 2019 dataset for young men with EOP enlargement.

### Usage of smartphones

**Figure 5:**
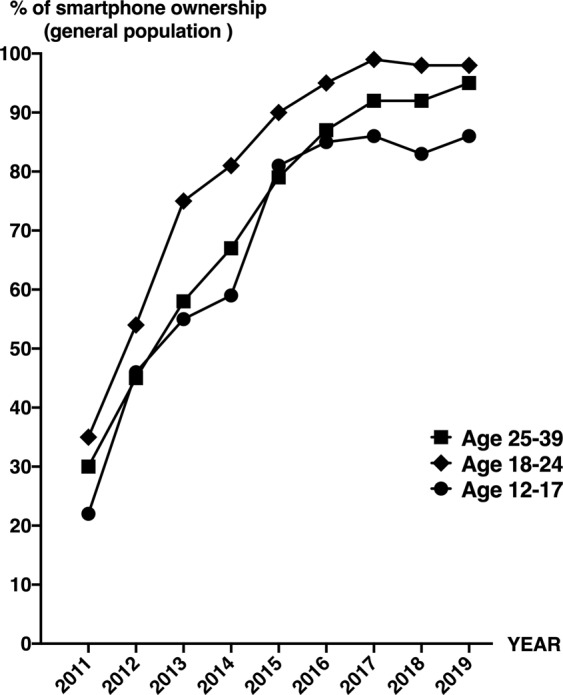
Evolution between 2011 and 2019 of the ownership of smartphones at a national scale (adapted from^14^**)**.

On a national scale^14^, the rate of ownership of smartphones in 2011 was estimated to 22% for the 12–17 year-olds, 35% for the 18–24 year-olds and 30% for the 25–39 year-olds. It drastically increased respectively to 86%, 98% and 95% in 2019 (Fig. 5). Though no direct relationship can be established, this major increase contrasts with the stability of the rate and volume of EOP enlargement during the same time period.

## Discussion

The impact of modern life on EOP changes in millennials has been the subject of recent publications^4,10,11^. However, these publications relied on data from conventional X-ray examinations and did not provide several time points, to better understand the evolution of EOP measurements over time. Actually, the medical literature regarding EOP size remains poor, and no volumetric data were ever published on this topic so far. Thus, a dedicated scientific study with a more precise evaluation of EOP size and shape, and including data from different times seemed necessary.

On a population scale, our study shows that EOP enlargement is a frequent finding in *millennials*, and was already present at the very beginning of the smartphone era, without significant differences in prevalence or size as compared to today, which is not in favor of a rapidly changing environmental factor during this period.

This study is the first to study EOP using Computerized Tomography (CT). This imaging modality is more sensitive than conventional radiography for the depiction of changes in the bone. This highly accurate assessment might have led to an overestimation on prevalence and size of EOP as compared to radiographic or morphologic studies. However, it is interesting to note that our results are consistent with a previous study based on conventional radiographs^4^ that reported an enlarged EOP in 41% of the population (44.2–44.9% in our study), more frequently in men (67.4% *versus* 65.3–65.7% in our study) than in women (20.3% *versus* 17–17.2% in our study). On the other hand, a prevalence of only 10% was reported in one morphologic study that analyzed cadaveric skulls^7^. It has also been reported^16^ that male individuals have higher occipital bone thickness around the EOP. The use of imaging techniques could thus be overestimating the prevalence of EOP enlargement compared to morphological studies, partly because it is sometimes difficult to differentiate the deep part of the EOP from a focal enlargement of the occipital bone.

There is no consensus on the definition of an enlarged EOP. Previous studies^4,7,10^ relied on only one or two linear measurements to define EOP enlargement. However, this approach does not take the shape of the EOP into account, which can be highly variable between individuals. To overcome this issue, we decided to measure EOP volume instead, because it enabled a more precise and suitable delineation of the EOP.

This study focused on a limited time (8 years) and cannot rule out a more progressive impact of other environmental factors from older decades on EOP size in the general population. However, this study also shows, through two older examples, that EOP enlargement was already present in young individuals from ancient populations. Interestingly, the CT measurement showed in both cases values within today’s upper ranges (respectively 85^th^ and 73^rd^ percentiles gender-wise), underlining the fact that if mechanical constraints are to be implied in EOP enlargement, some of them were already faced by our ancestors. This study only shows two of these examples and cannot rule out, however, that the results would have been different on a larger population of ancient skulls and that the evolution of EOP over time follows a slowly progressive path. Precise assessment of the changes in EOP over longer periods of time, with adjustment towards confounding factors that might change neck constraints (e.g. an individual’s size, neck musculature, working conditions or physical activities) would be useful.

On an individual scale, it is noticeable that few cases from both 2011 and 2019 datasets were clear outliers (Fig. 2) with some of them displaying an unusually large EOP (Fig. 1). It would be interesting to focus individually on patients with this kind of EOP to look for innate or environmental factors that could have led to this condition. One study^11^ reported that inflammatory or genetic factors did not seem to be involved in EOP enlargement, but it relied on a small number of patients. It would be interesting to undertake such a study on a larger scale, especially in symptomatic patients.

We acknowledge several limitations in our study. Firstly, it was a retrospective study performed at a single institution (university hospital). Studies performed in different hospitals with different CT indications or in different countries might show different results. Nevertheless, we believe our methodology favored a comparison between homogenous populations.

Secondly, since the work was performed retrospectively on anonymized CT datasets, it was not possible to assess directly the individual use of smartphones of patients. The data we used were derived from national official data on this topic, and give an overall glimpse of the use of smartphones. However, while interesting, they represent only one environmental parameter, among many potential confounding factors. Though the impact of rapidly growing modern environmental factors - such as smartphones - on EOP changes seems unlikely given the stability of EOP size in the last decade, the design of our study does not allow thorough correlation with risk factors. Thus, further studies with a robust design enabling an adjustment of EOP size with confounding factors are needed to draw more robust conclusions on the impact of environment on EOP changes.

Thirdly, since the datasets were retrospective and anonymized, we could not establish a correlation between the size of the EOP and potential cervical symptoms. However, the datasets were derived from head CT performed in the Emergency Radiology Department of our institution, for which the main imaging indications are related to an acute event (head trauma, acute headache or acute neurological symptom for example), and without *a priori* specific reasons to suffer from cervical symptoms more than the general population. The choice to work on head CT examinations rather than cervical spine CT examinations was driven partly by this reason, because the dataset could have been biased in the latter, with an over-representation of patient with cervical symptoms that could have altered the generalization of our description. Finally, one can argue that 2011 is not far enough to draw conclusions on the evolution of EOP over a longer period of time. This study specifically focuses on *millennials* aged 18-30 and the potential rapidly evolving impact on modern life on this population, since they were the principal age group for whom concerns were raised^10^. Going further back in time would have led to the inclusion of patient from previous generation X, who underwent different life events and environmental constraints^15^. Moreover, the number of confounding factors resulting in ossification of the entheses (mechanical, inflammatory, metabolic…) increases with age^17^. Since our design did not enable a robust adjustment for confounding factors, the addition of older patients could have introduced a significant bias.

## Conclusion

On a population scale, prevalence and volume of enlarged EOP (“occipital spur”) in millennials remained stable between 2011 and 2019, although the use of smartphones has risen drastically in the meantime. Interestingly, CT analysis of ancient skulls from young individuals (XVI^th^ century France and Ptolemaic Egypt) showed values within today’s upper ranges (gender-wise) and comforts these findings. These findings suggest that EOP enlargement was already present and frequent at the beginning of the last decade, and probably even long before. This makes the impact of rapidly growing modern environmental factors - such as smartphones - on EOP changes unlikely. However, further studies are needed to fully understand the innate and/or environmental factors that could lead to a major EOP enlargement in certain patients, without forgetting that this finding is very rarely symptomatic.

## Supplementary information

Supplementary information.

Dataset.
