# Supplementary material for: Enlarged External Occipital Protuberance in young French individuals’ head CT: stability in prevalence, size and type between 2011 and 2019
Source: Sci Rep. 2020 Apr 16;10:6518. doi: 10.1038/s41598-020-63554-y (PMC7162866; doi:10.1038/s41598-020-63554-y)
Supplement: Supplementary file 1 — Supplementary information. [file 41598_2020_63554_MOESM1_ESM.pdf]

SUPPLEMENTARY INFORMATION FILE

« Enlarged External Occipital Protuberance in young French individuals' head CT: stability in prevalence, size and type between 2011 and 2019. »

Thibaut JACQUES*, Alexandre JAOUEN, Gregory KUCHCINSKI, Sammy BADR, Xavier DEMONDION, Anne COTTEN.

Volume measurement images are labelled as follows: Year YY/gender/number (ex: 11f1 = 2011 dataset / female / case 1 of EOP enlargement)

11f1

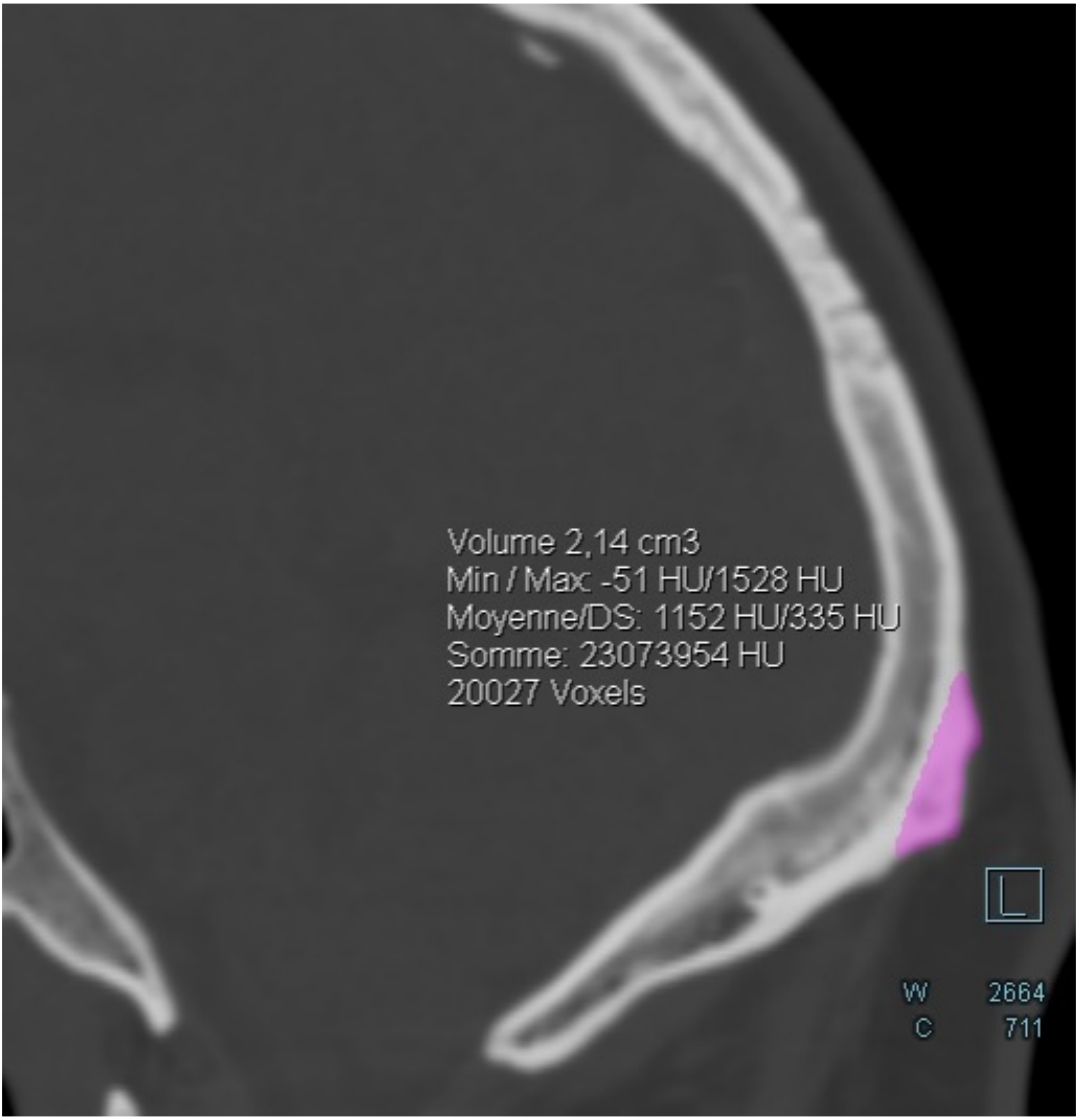

11f2

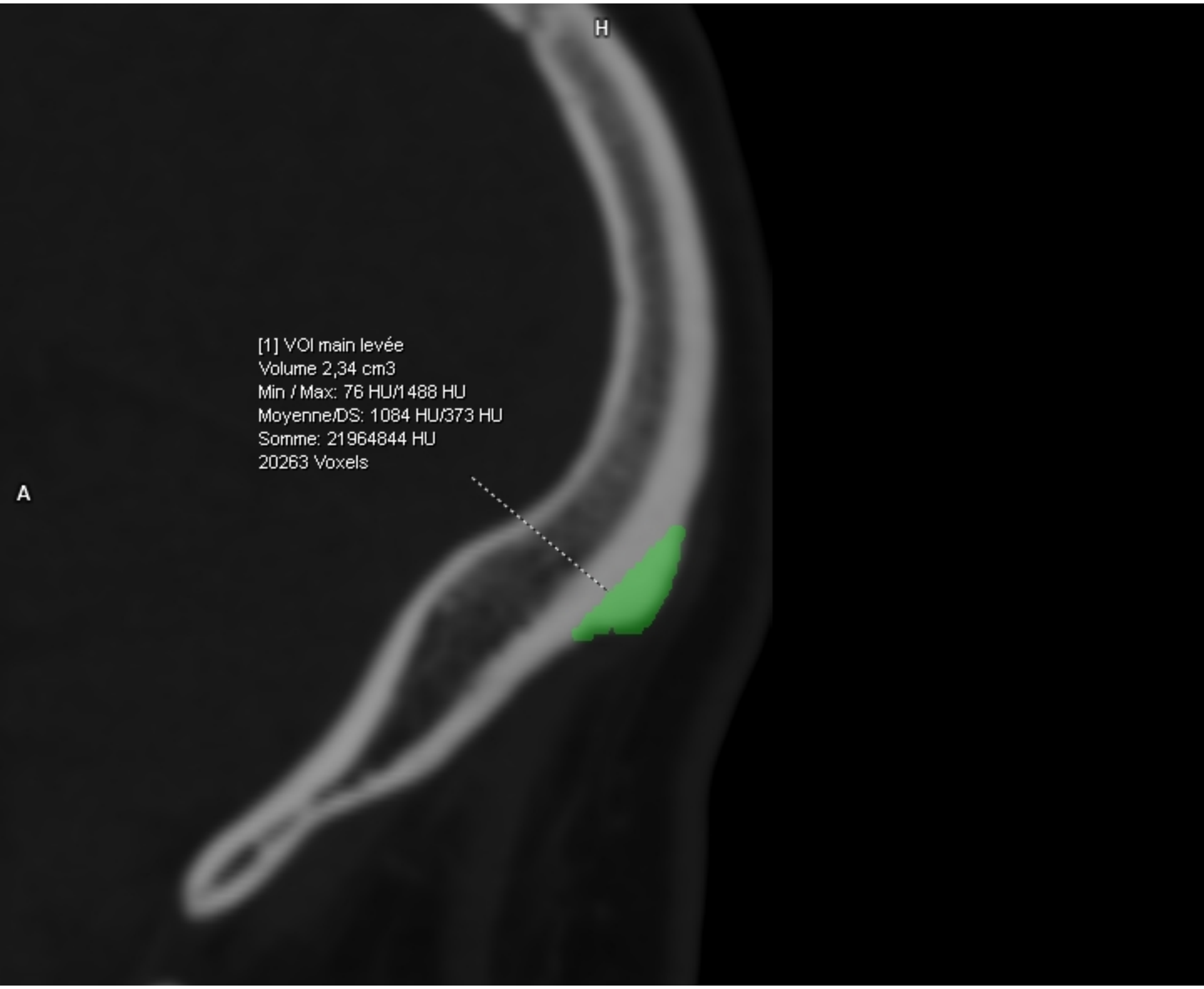
H
[1] VOI main levée
Volume 2,34 cm3
Min / Max: 76 HU/1488 HU
Moyenne/DS: 1084 HU/373 HU
Somme: 21964844 HU
20263 Voxels
A

11f3

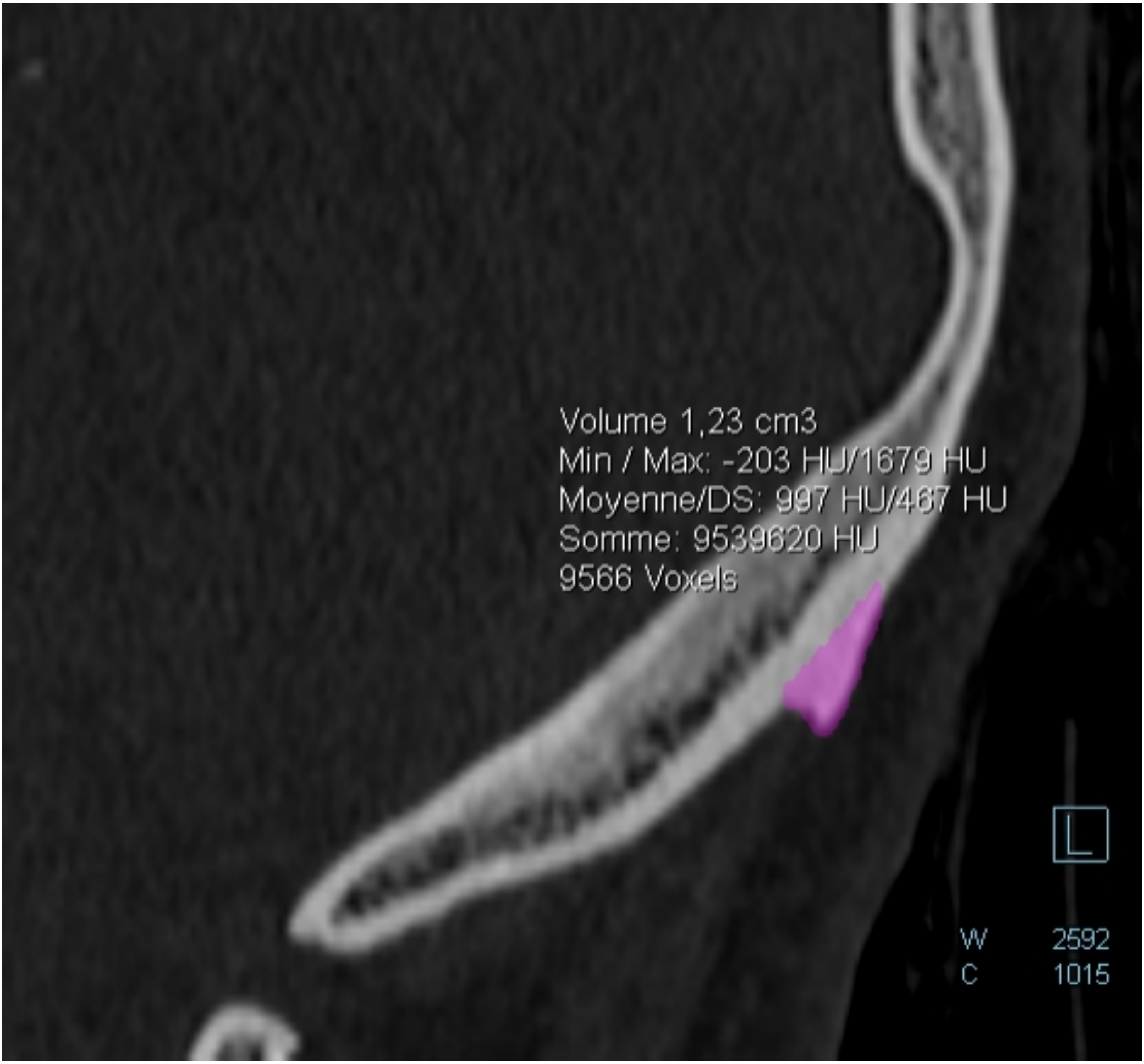
Volume 1,23 cm3
Min / Max: -203 HU/1679 HU
Moyenne/DS: 997 HU/467 HU
Somme: 9539620 HU
9566 Voxels
L
W 2592
C 1015

11f4

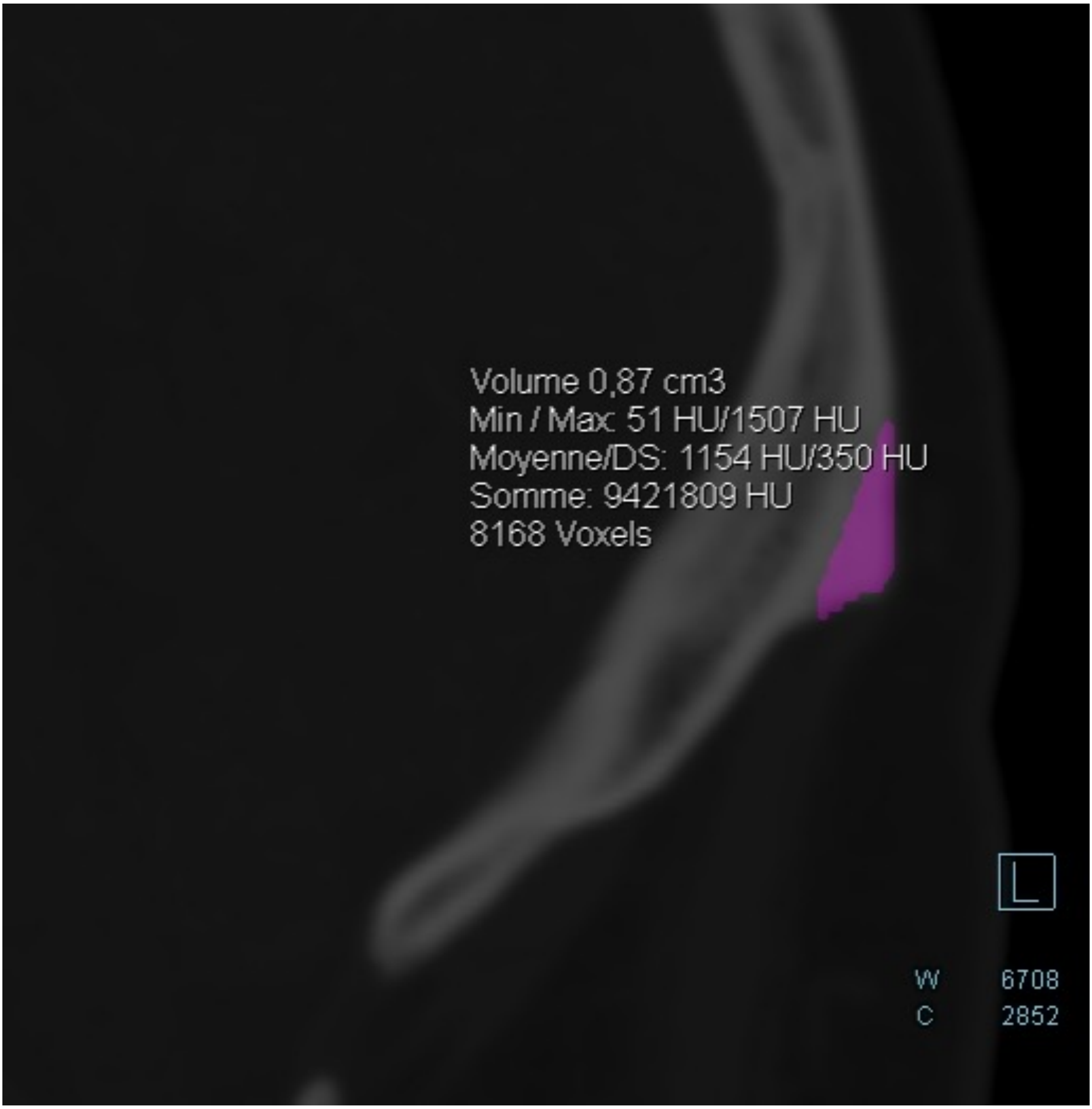
Volume 0,87 cm3
Min / Max: 51 HU/1507 HU
Moyenne/DS: 1154 HU/350 HU
Somme: 9421809 HU
8168 Voxels
L
W 6708
C 2852

11f5

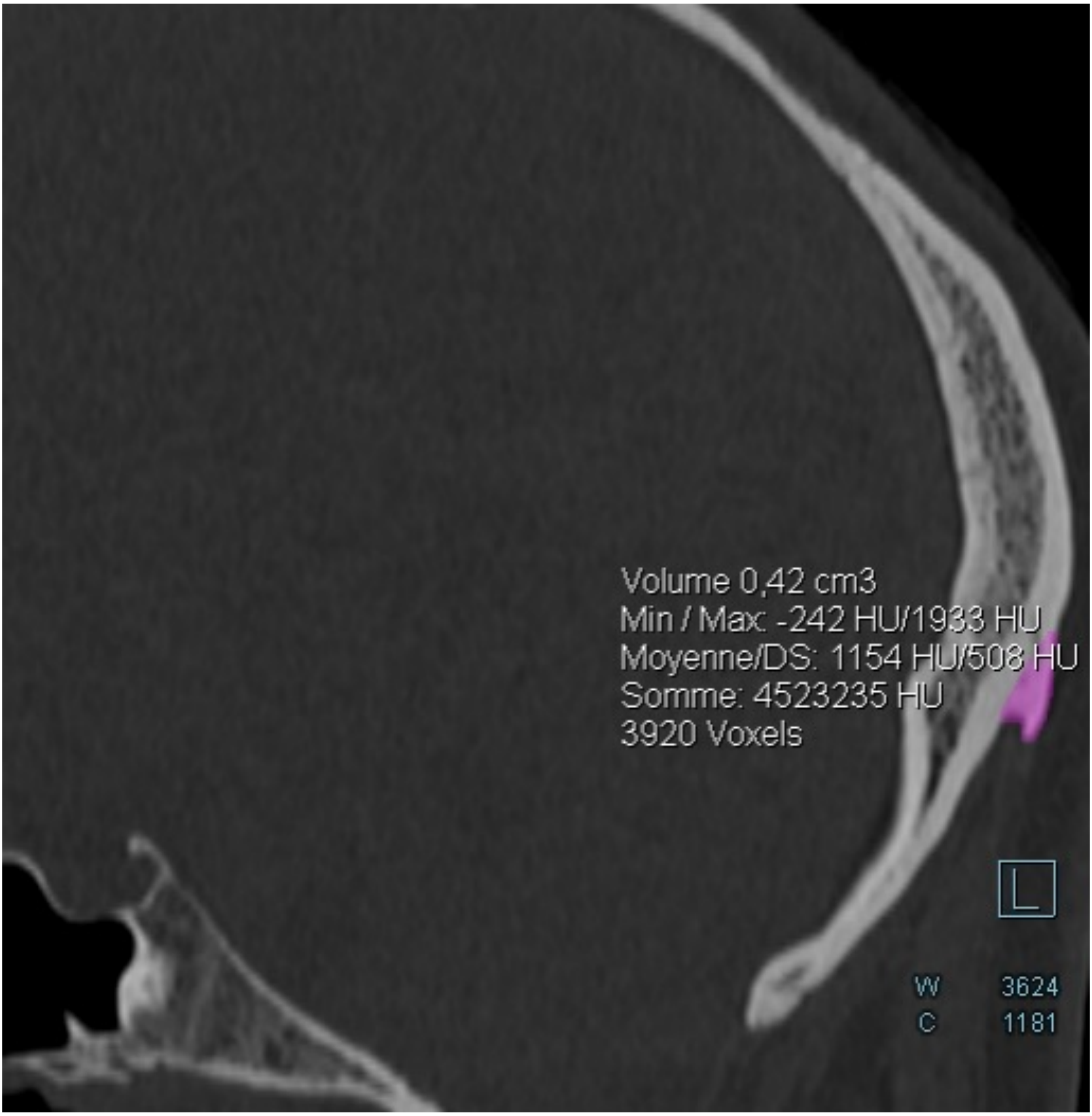

Volume 0,42 cm3
Min / Max: -242 HU/1933 HU
Moyenne/DS: 1154 HU/508 HU
Somme: 4523235 HU
3920 Voxels
L
W 3624
C 1181

11f6

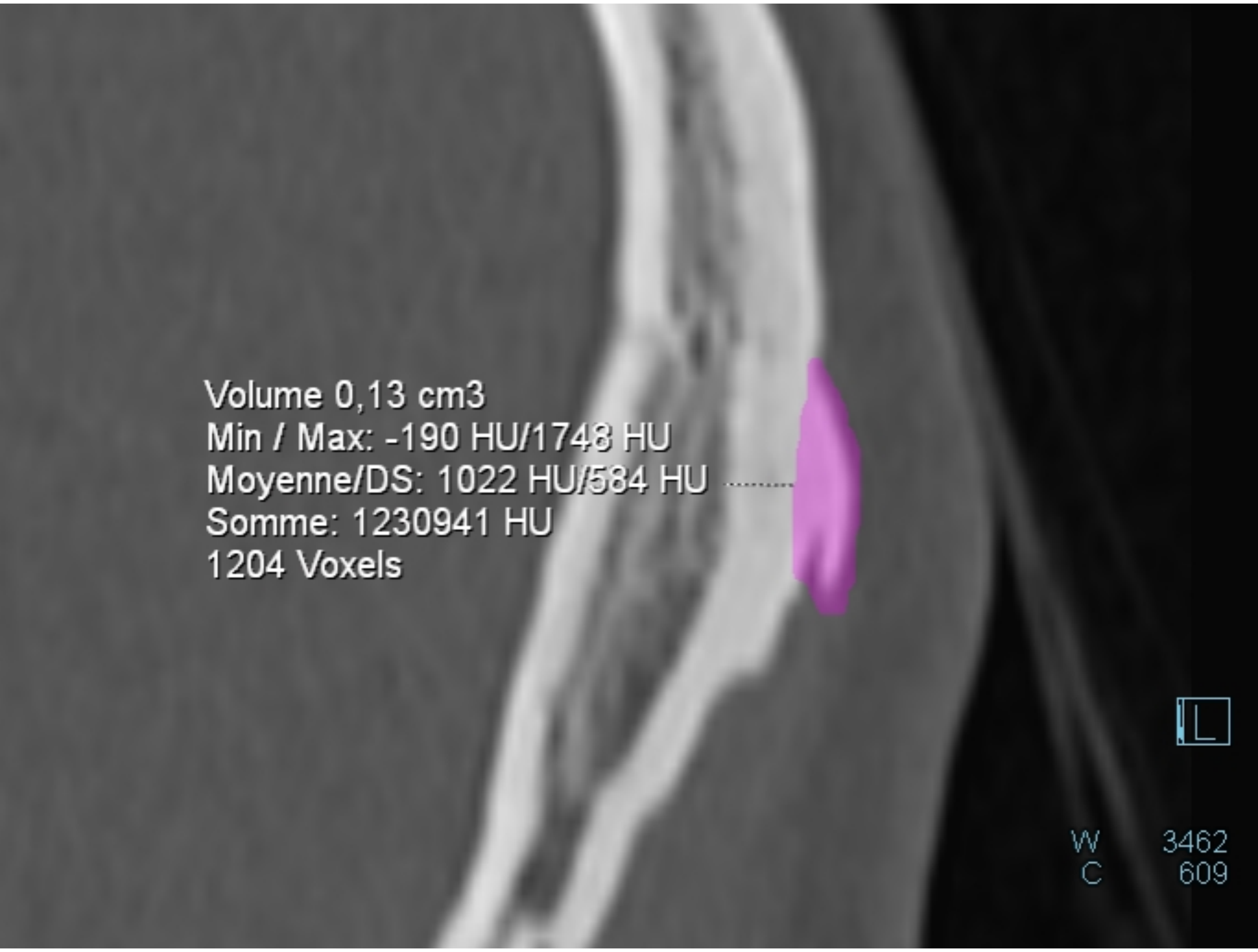
Volume 0,13 cm3
Min / Max: -190 HU/1748 HU
Moyenne/DS: 1022 HU/584 HU
Somme: 1230941 HU
1204 Voxels
L
W 3462
C 609

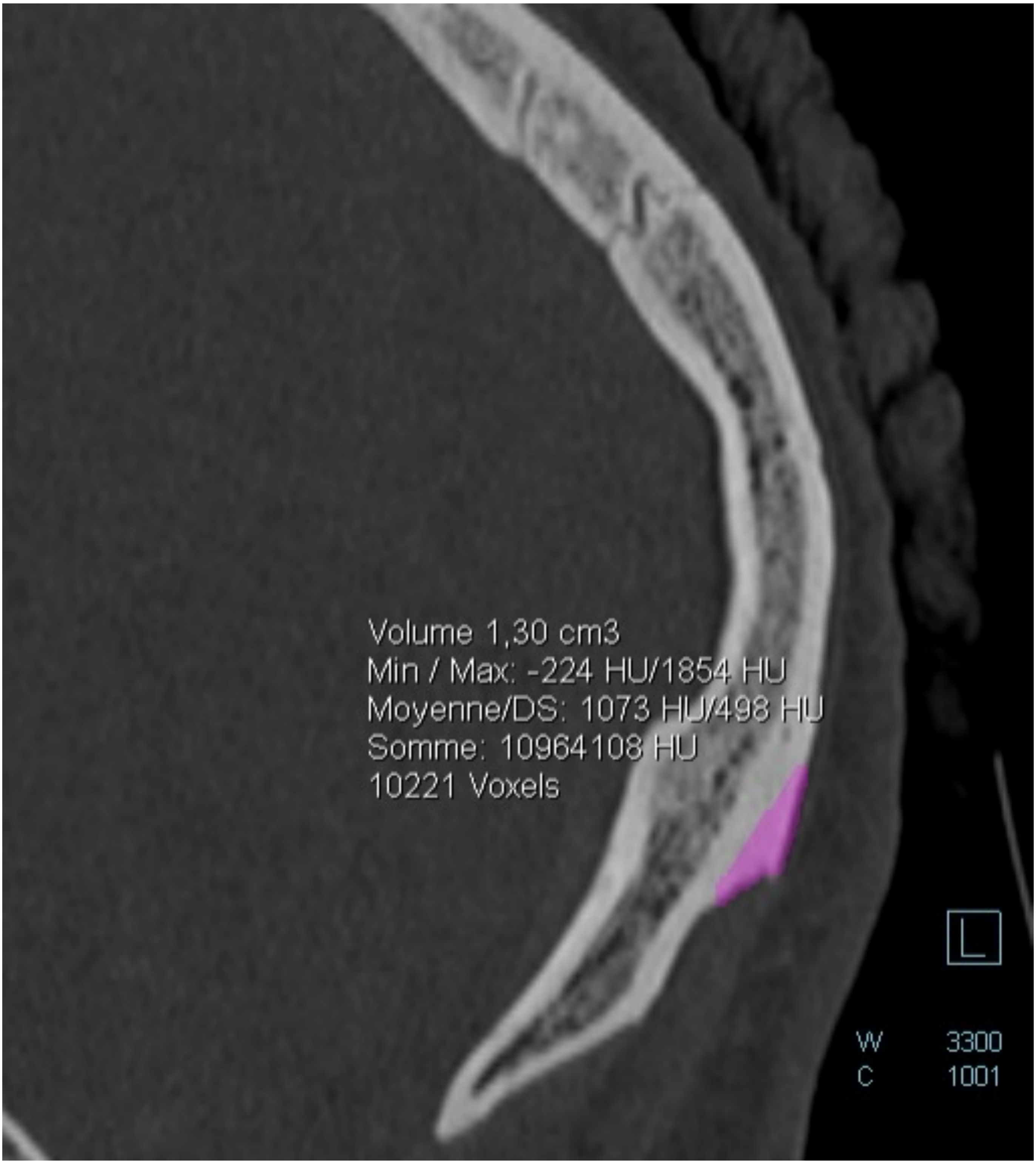
Volume 1,30 cm3
Min / Max: -224 HU/1854 HU
Moyenne/DS: 1073 HU/498 HU
Somme: 10964108 HU
10221 Voxels
L
W 3300
C 1001

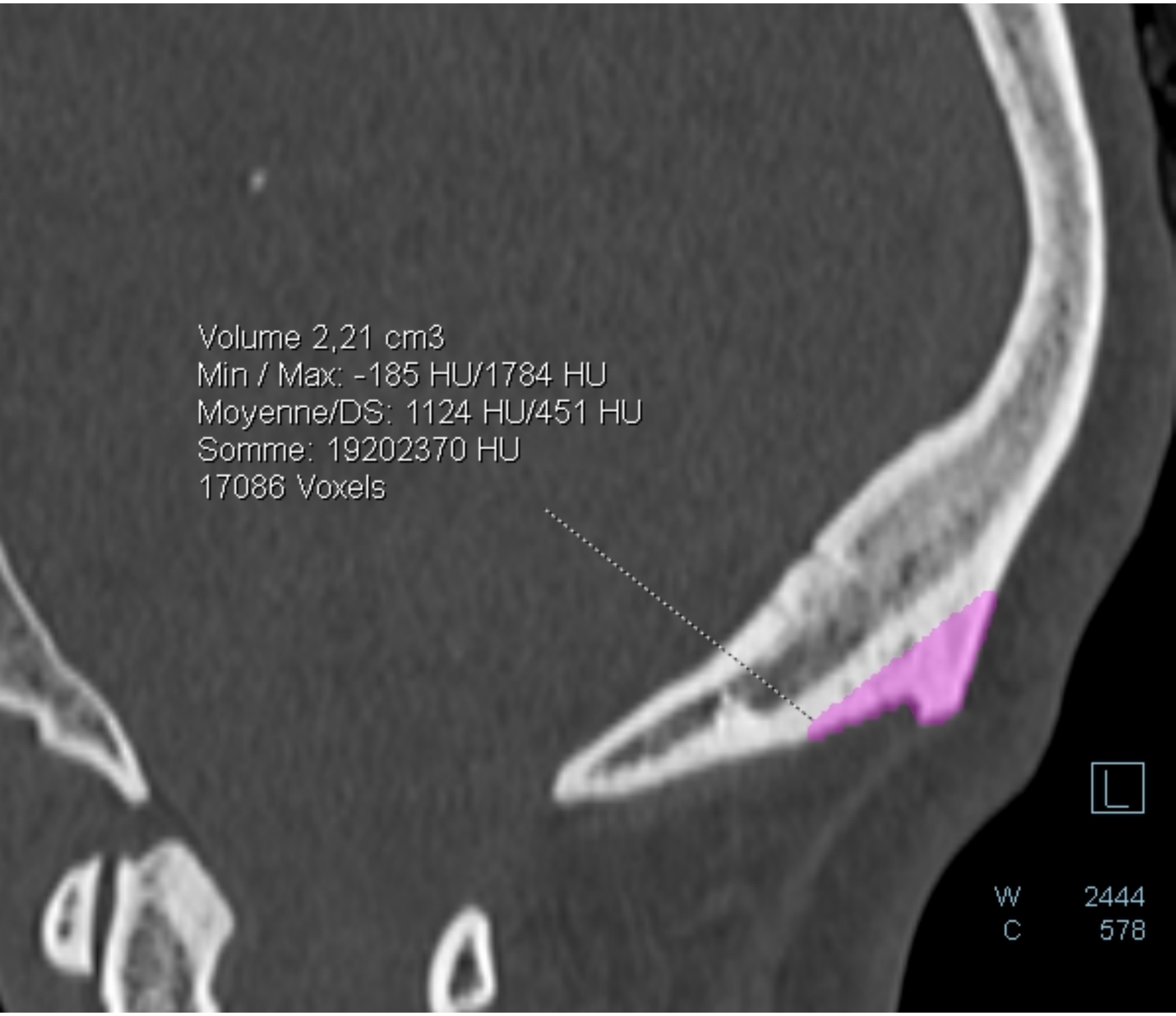
Volume 2,21 cm3
Min / Max: -185 HU/1784 HU
Moyenne/DS: 1124 HU/451 HU
Somme: 19202370 HU
17086 Voxels
L
W 2444
C 578

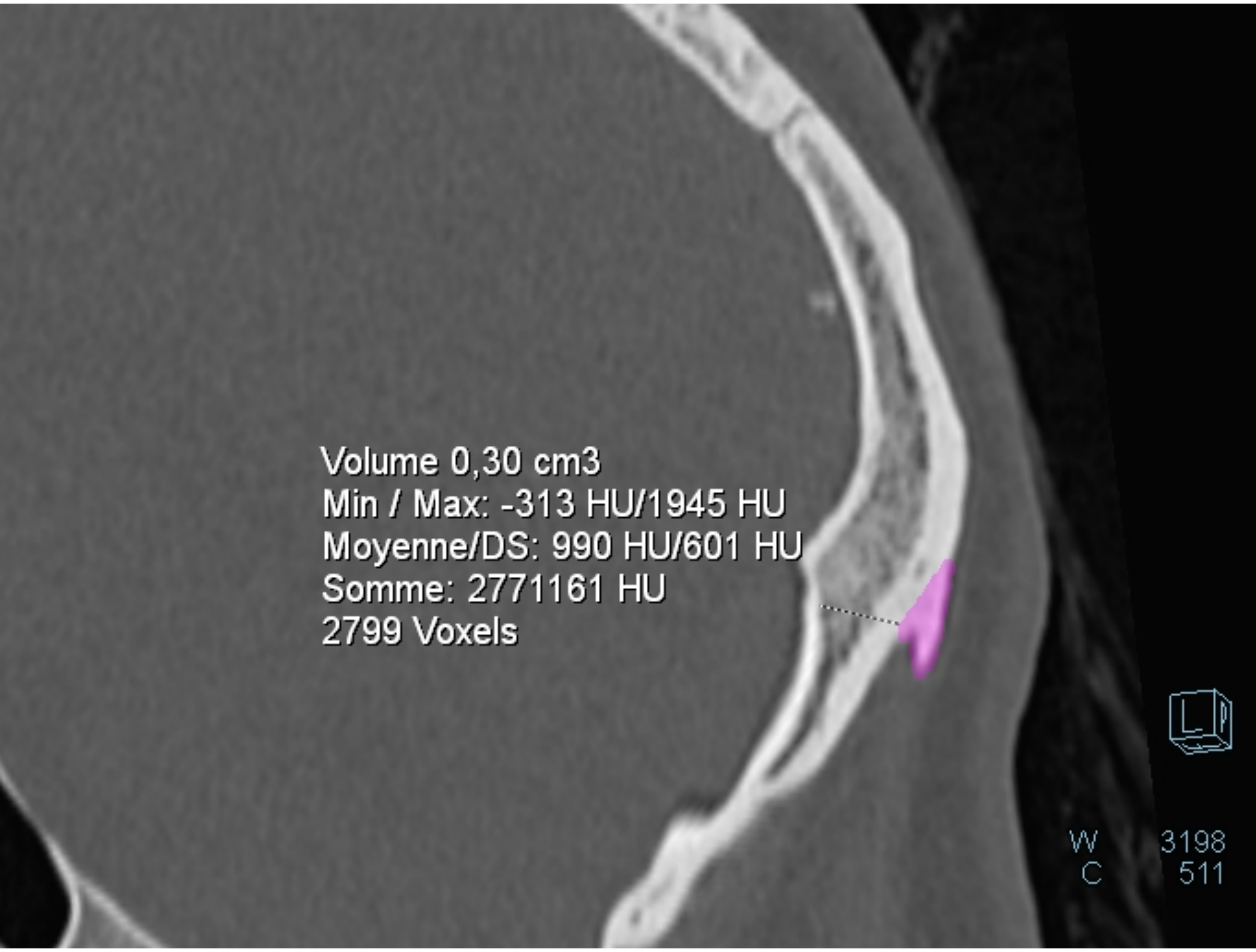
Volume 0,30 cm3
Min / Max: -313 HU/1945 HU
Moyenne/DS: 990 HU/601 HU
Somme: 2771161 HU
2799 Voxels
W 3198
C 511

11f10

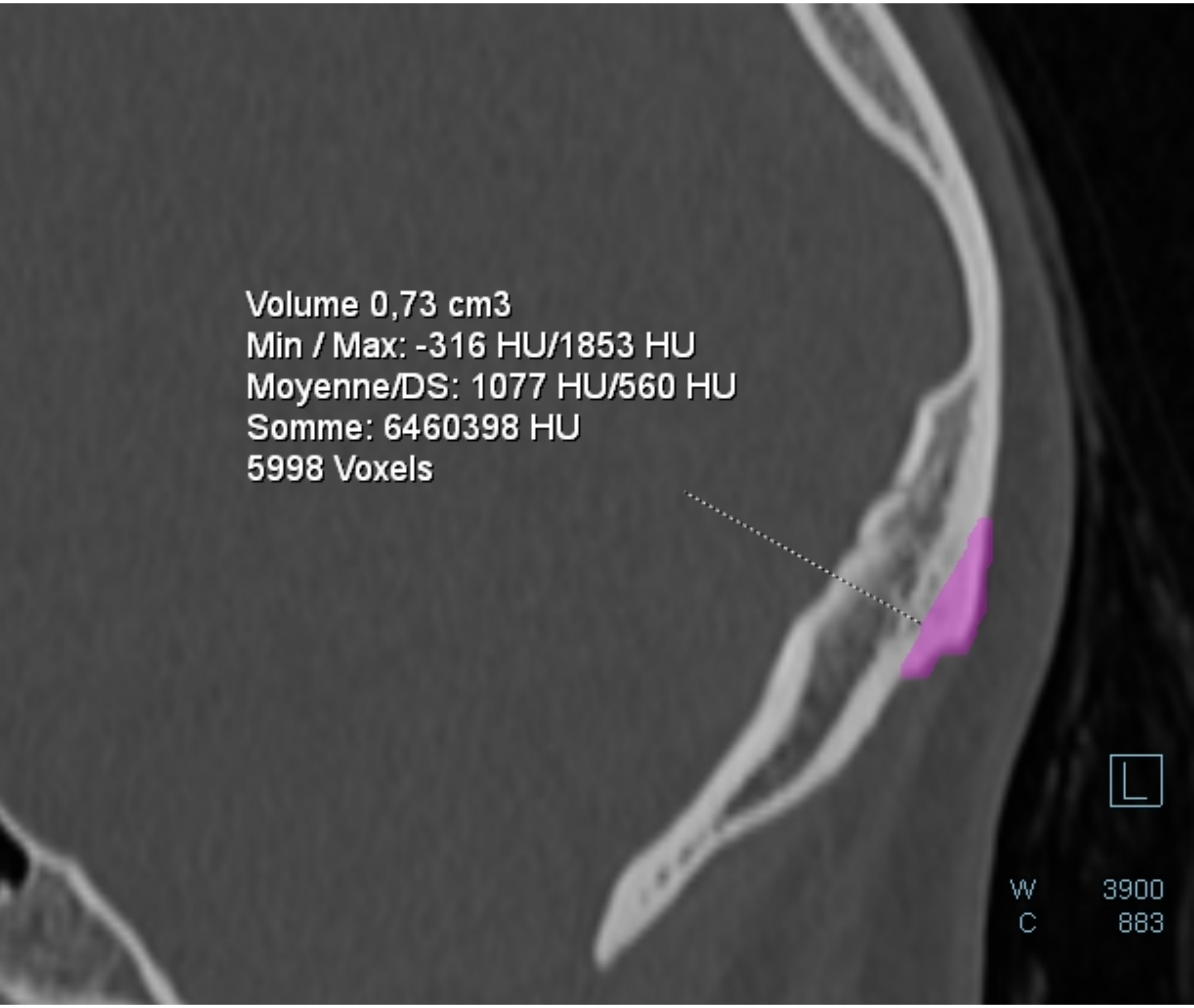
Volume 0,73 cm3
Min / Max: -316 HU/1853 HU
Moyenne/DS: 1077 HU/560 HU
Somme: 6460398 HU
5998 Voxels
L
W 3900
C 883

11f11

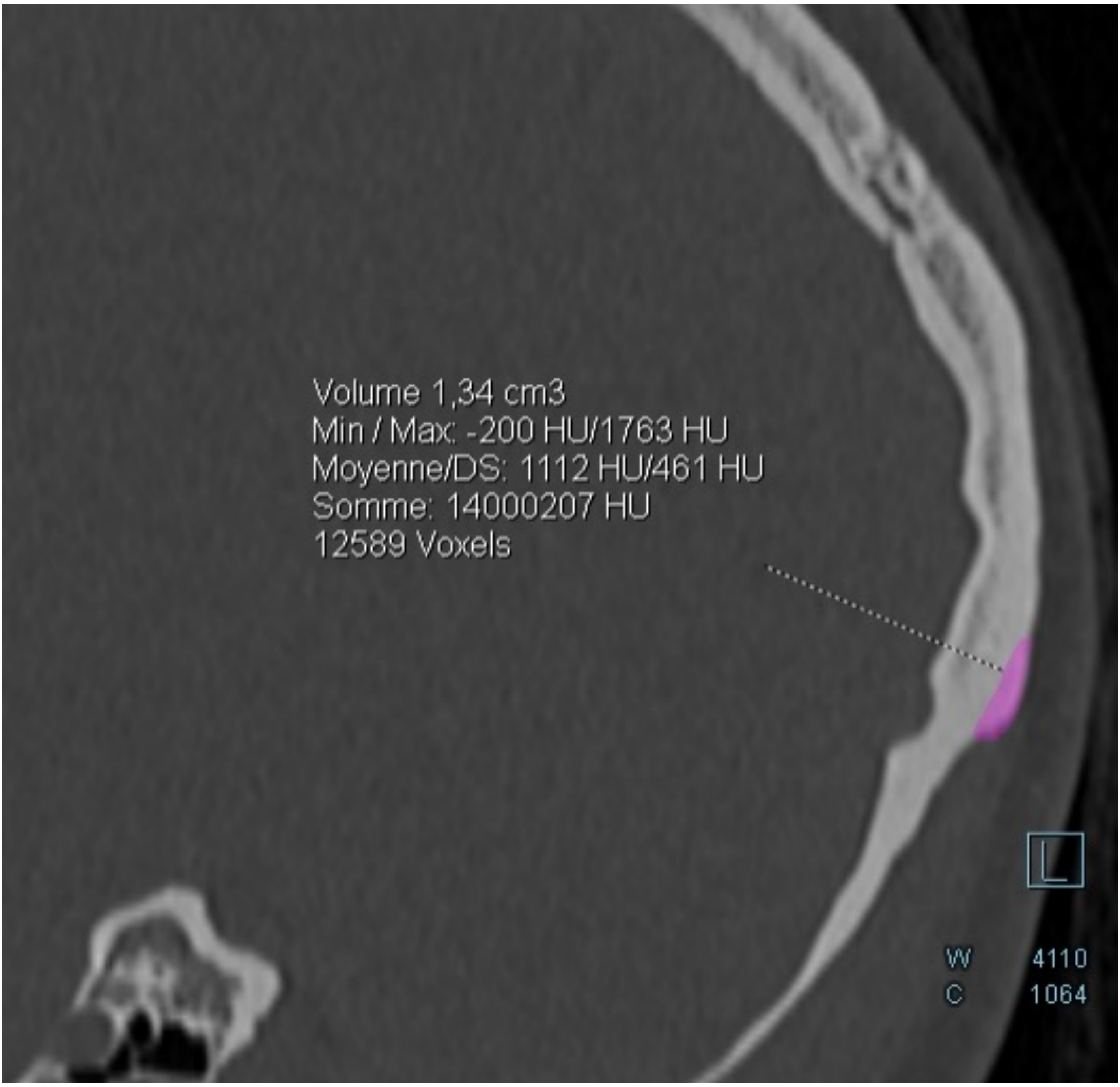

Volume 1,34 cm3
Min / Max: -200 HU/1763 HU
Moyenne/DS: 1112 HU/461 HU
Somme: 14000207 HU
12589 Voxels
L
W 4110
C 1064

11f12

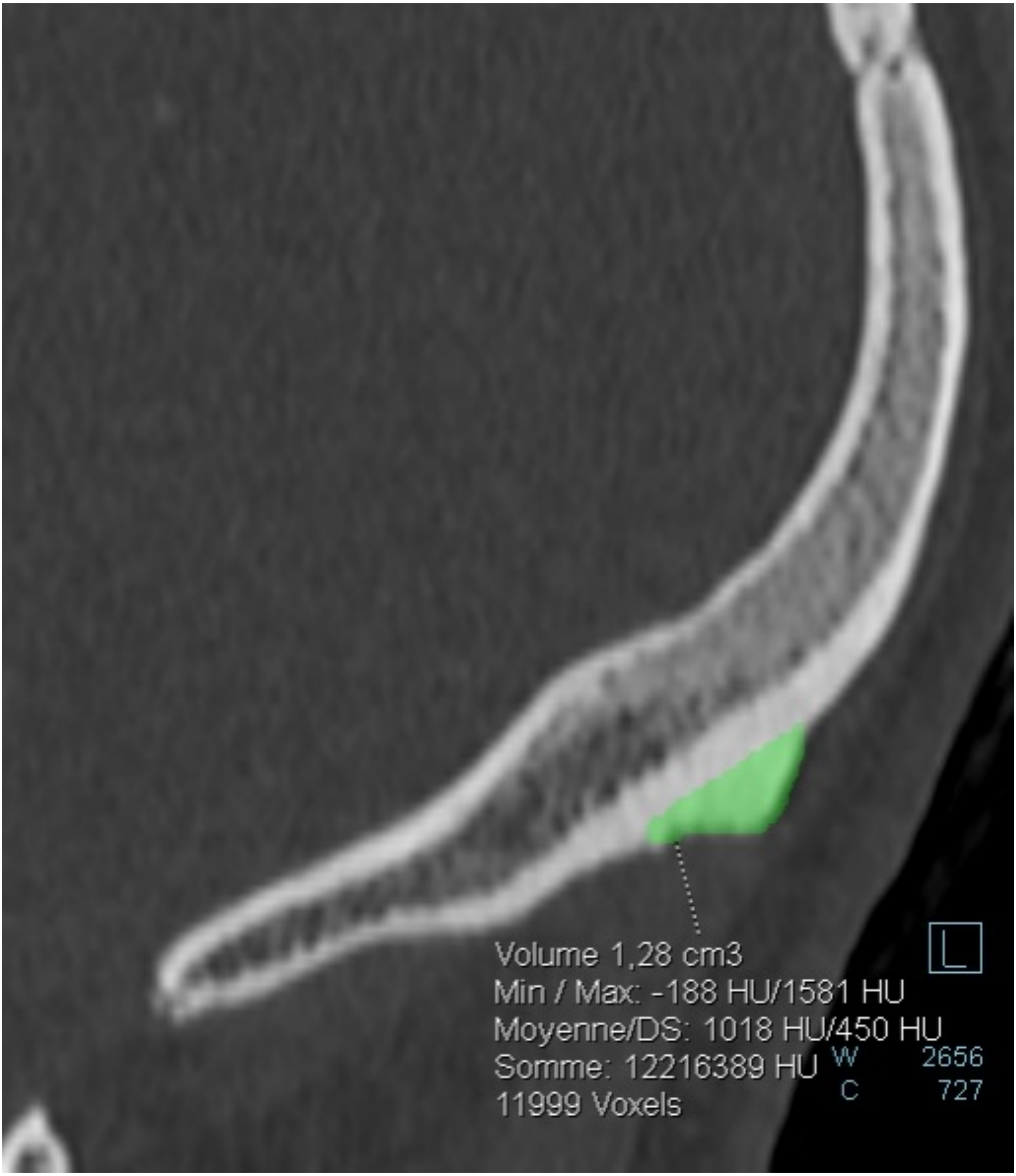
Volume 1,28 cm3
Min / Max: -188 HU/1581 HU
Moyenne/DS: 1018 HU/450 HU
Somme: 12216389 HU
11999 Voxels
W 2656
C 727
L

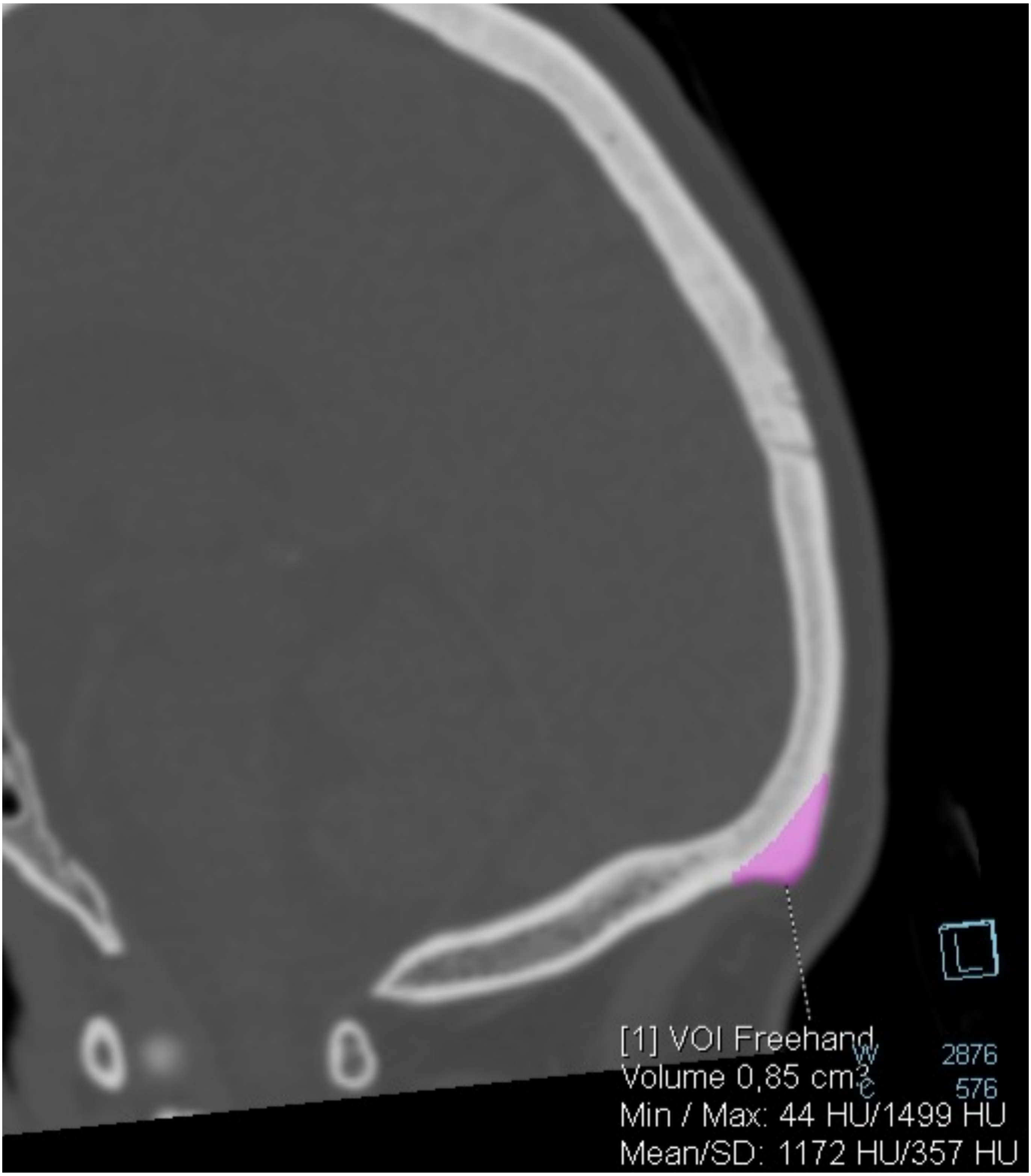
[1] VOI Freehand
Volume 0,85 cm3
Min / Max: 44 HU/1499 HU
Mean/SD: 1172 HU/357 HU
W 2876
C 576

11f14

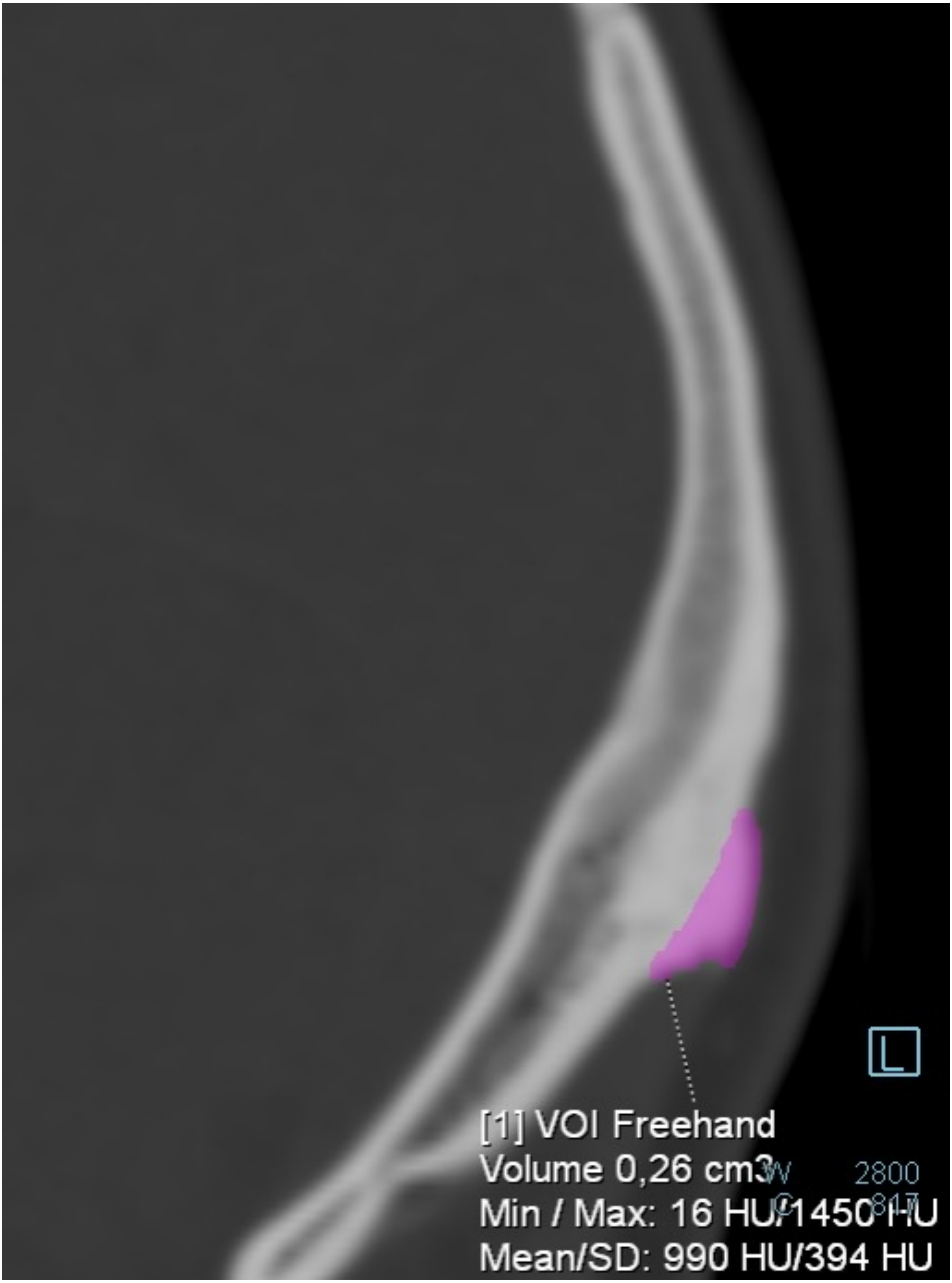

[1] VOI Freehand
Volume 0,26 cm3
Min / Max: 16 HU/1450 HU
Mean/SD: 990 HU/394 HU
W 2800
L

11f15

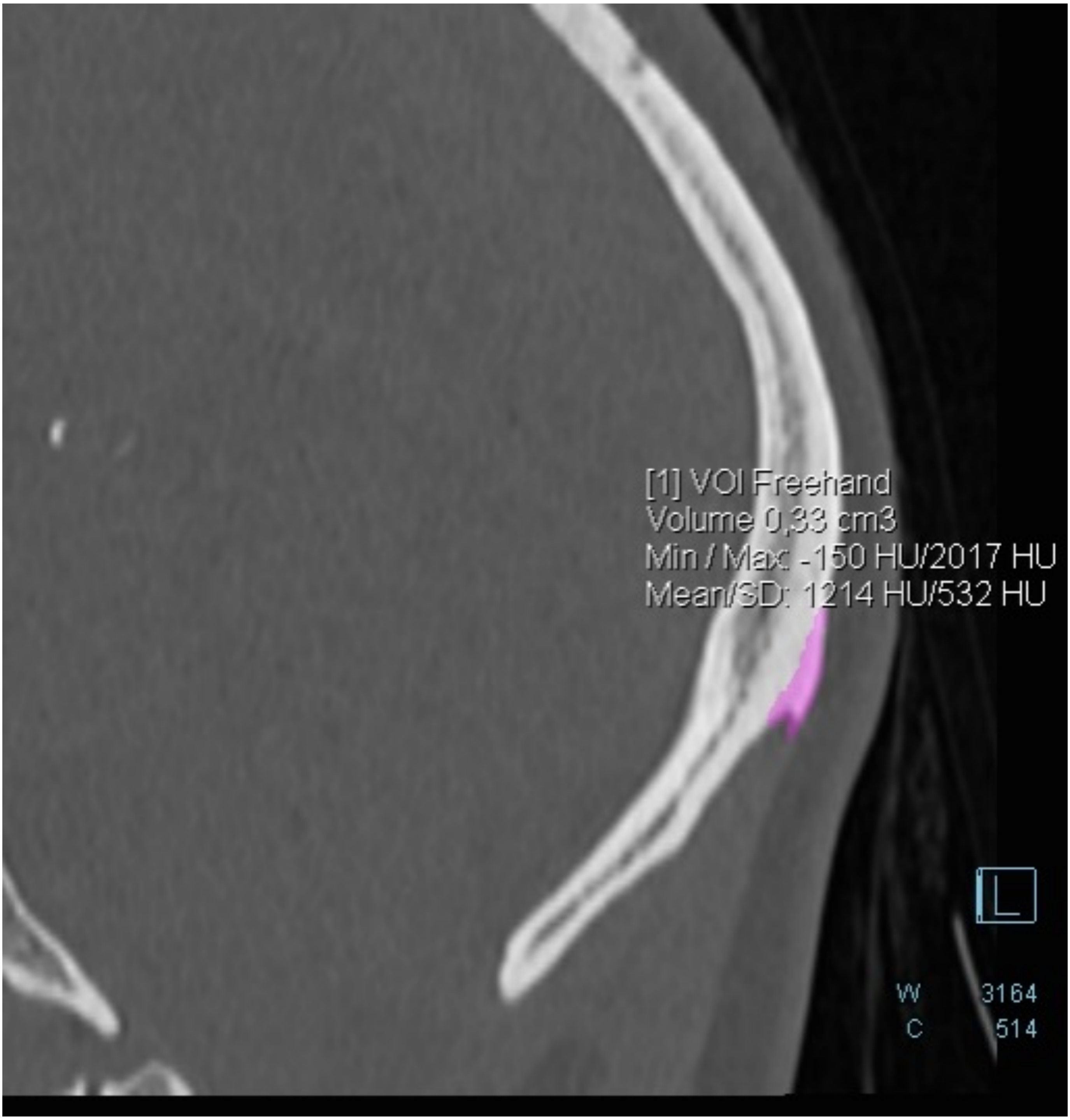
[1] VOI Freehand
Volume 0,33 cm3
Min / Max: -150 HU/2017 HU
Mean/SD: 1214 HU/532 HU
L
W 3164
C 514

11m1

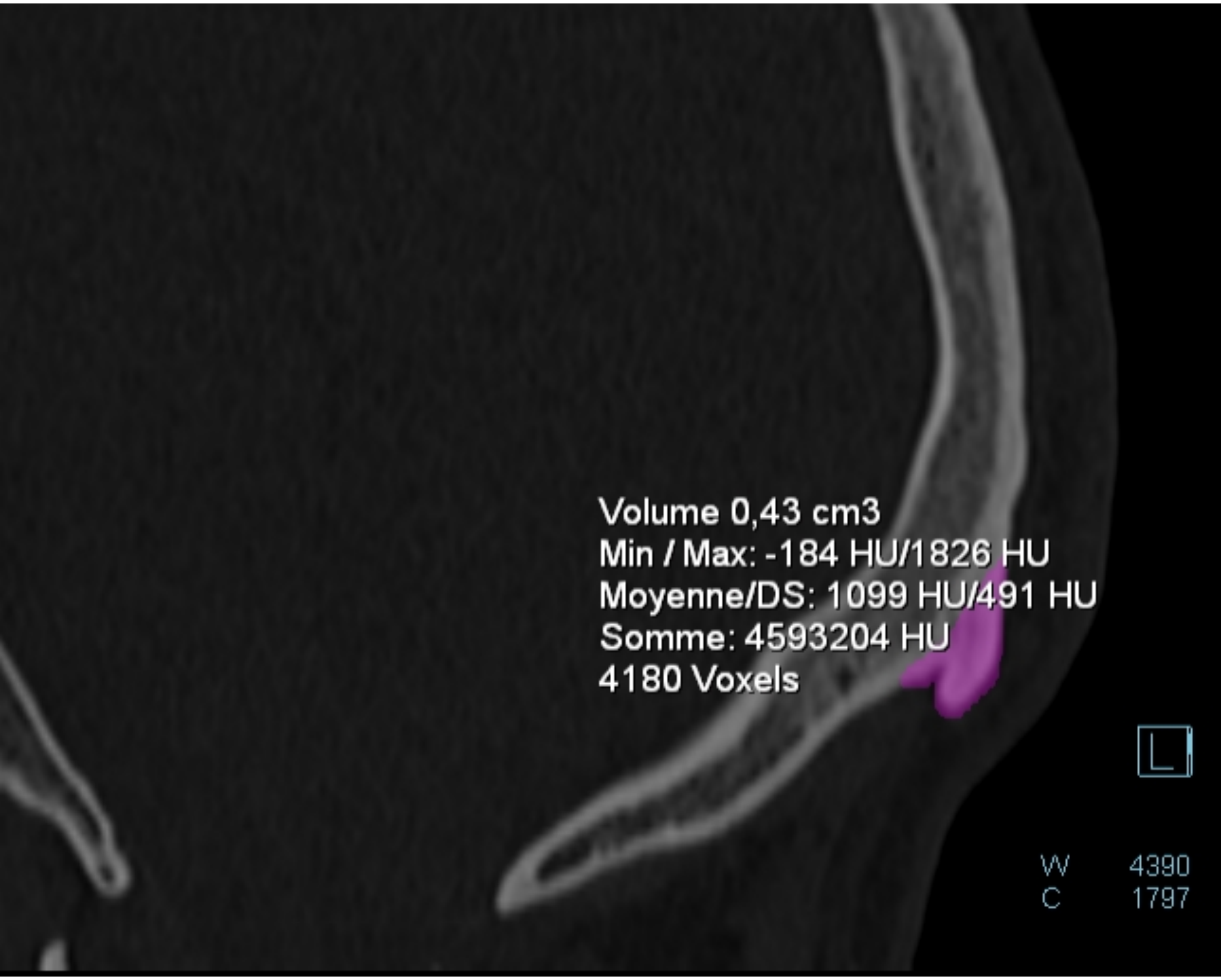

Volume 0,43 cm3
Min / Max: -184 HU/1826 HU
Moyenne/DS: 1099 HU/491 HU
Somme: 4593204 HU
4180 Voxels
L
W 4390
C 1797

11m2

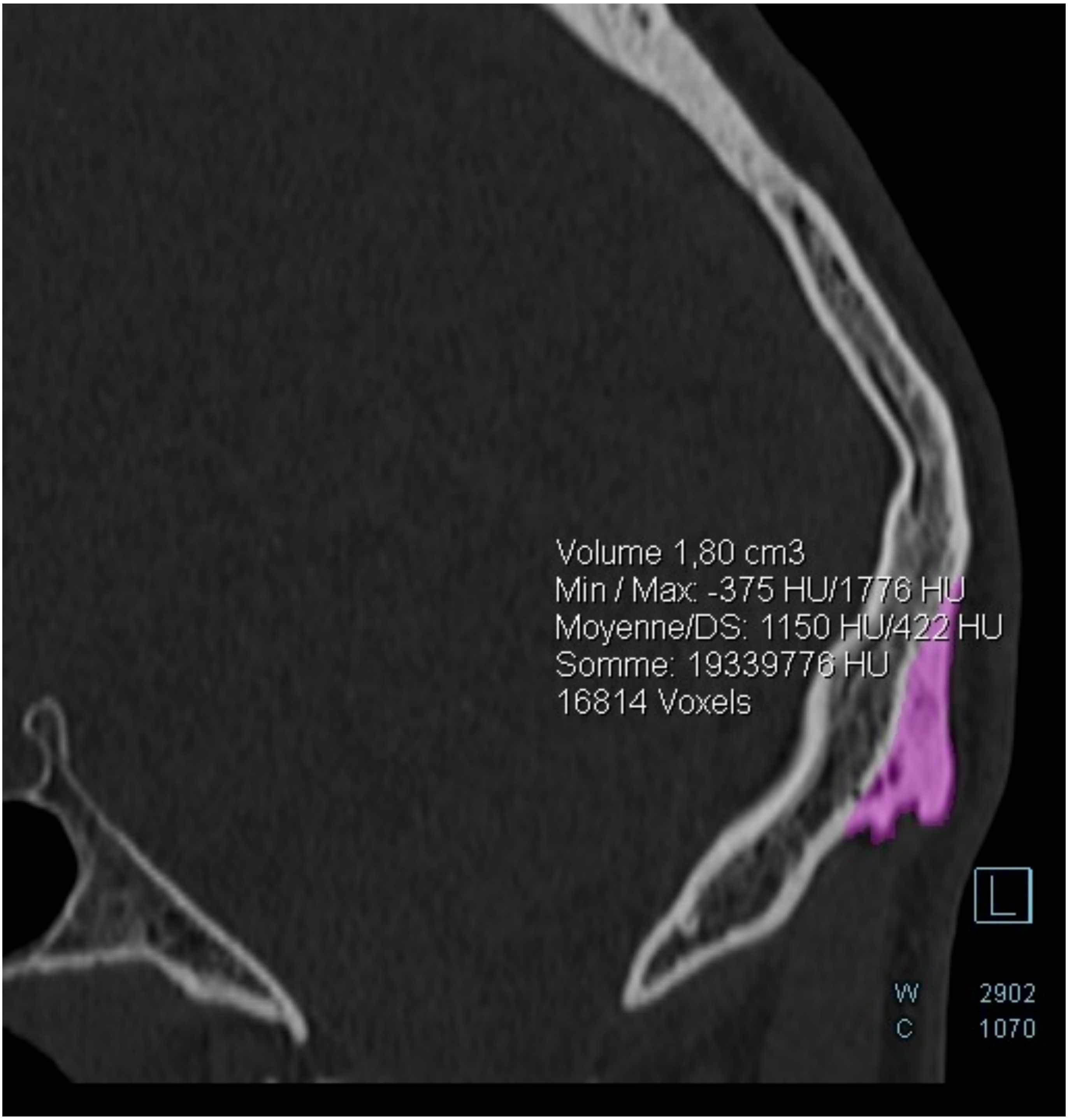

Volume 1,80 cm3
Min / Max: -375 HU/1776 HU
Moyenne/DS: 1150 HU/422 HU
Somme: 19339776 HU
16814 Voxels
L
W 2902
C 1070

11m3

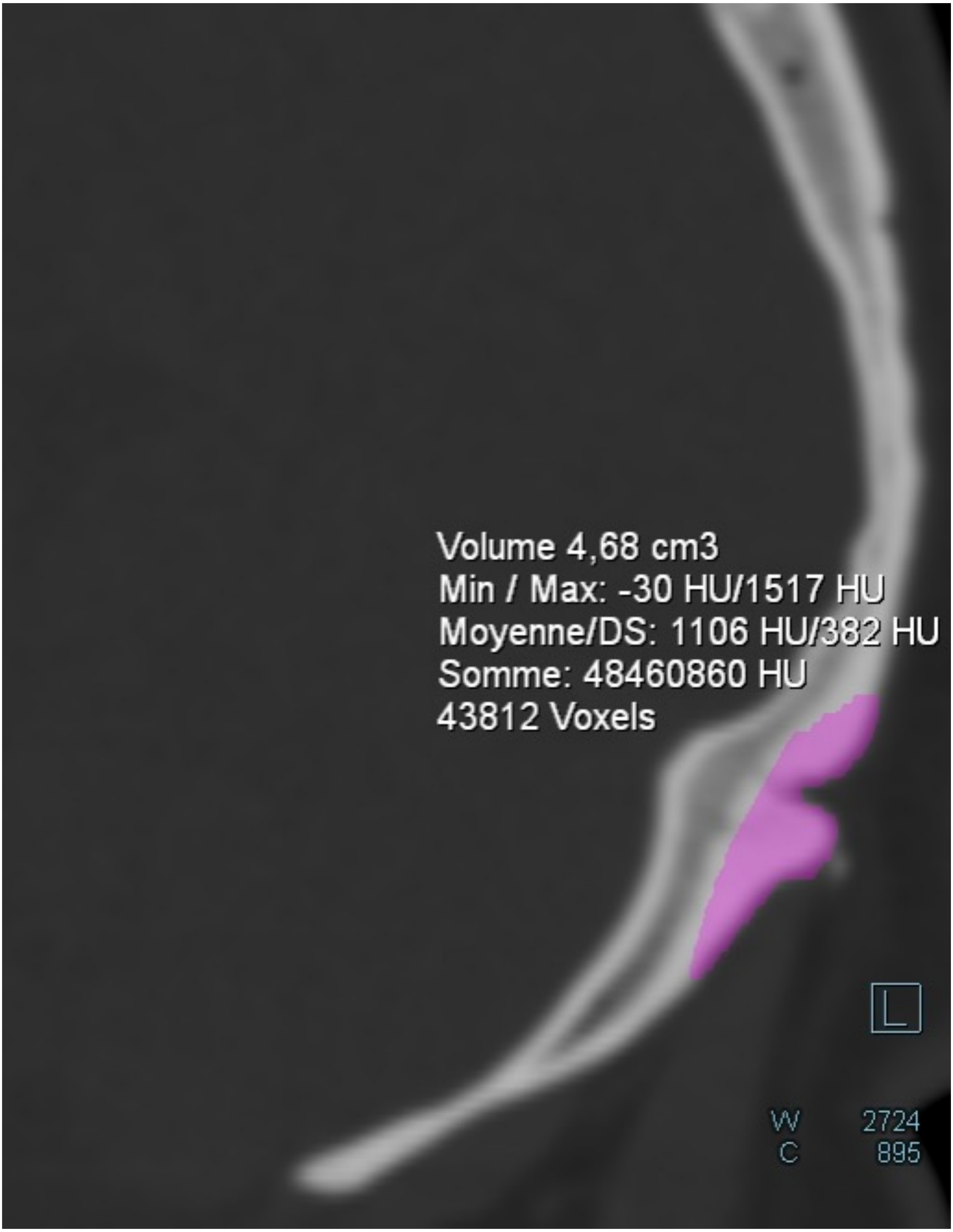
Volume 4,68 cm3
Min / Max: -30 HU/1517 HU
Moyenne/DS: 1106 HU/382 HU
Somme: 48460860 HU
43812 Voxels
L
W 2724
C 895

11m4

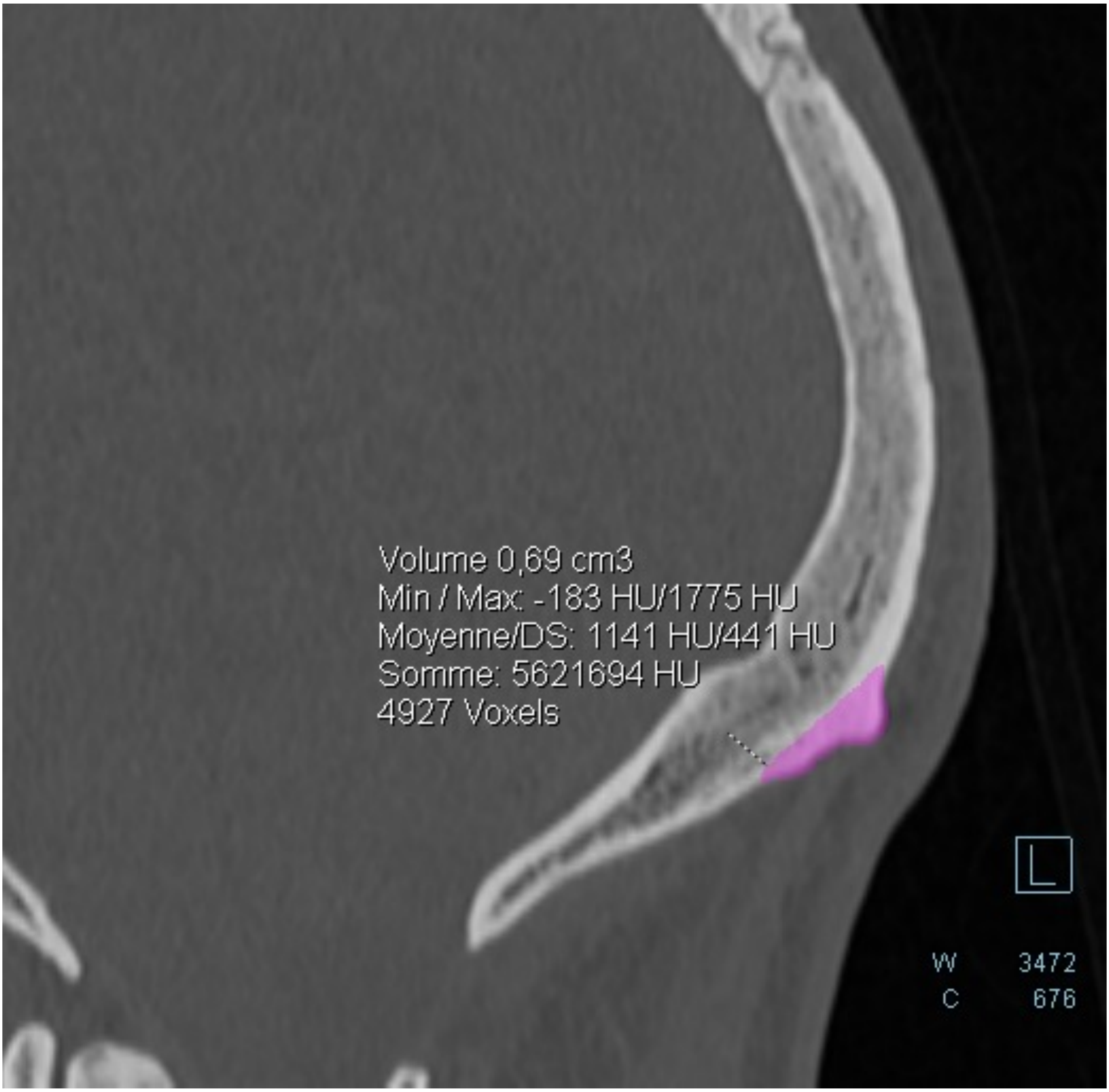
Volume 0,69 cm3
Min / Max: -183 HU/1775 HU
Moyenne/DS: 1141 HU/441 HU
Somme: 5621694 HU
4927 Voxels
L
W 3472
C 676

11m5

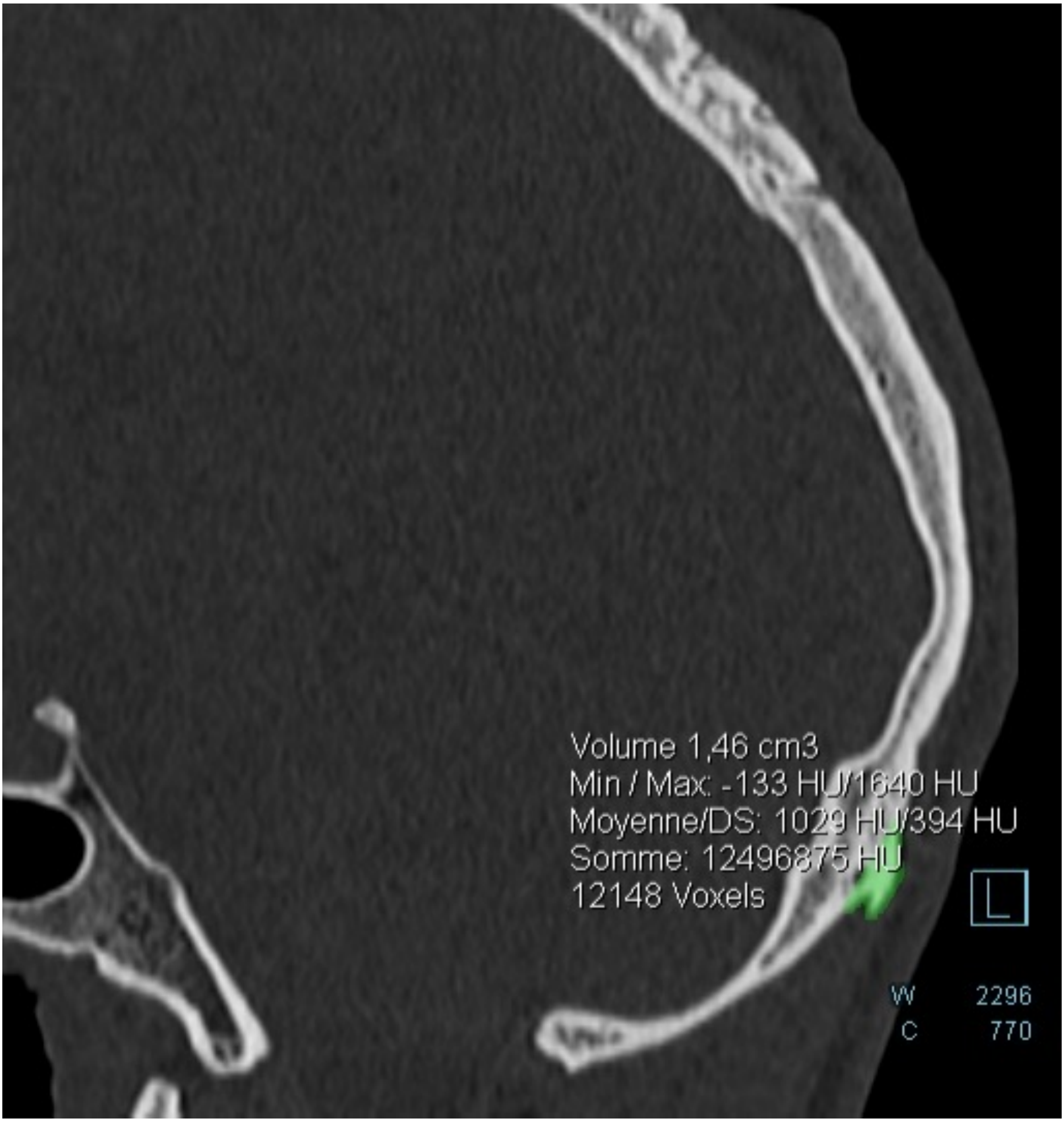
Volume 1,46 cm3
Min / Max: -133 HU/1640 HU
Moyenne/DS: 1029 HU/394 HU
Somme: 12496875 HU
12148 Voxels
L
W 2296
C 770

11m6

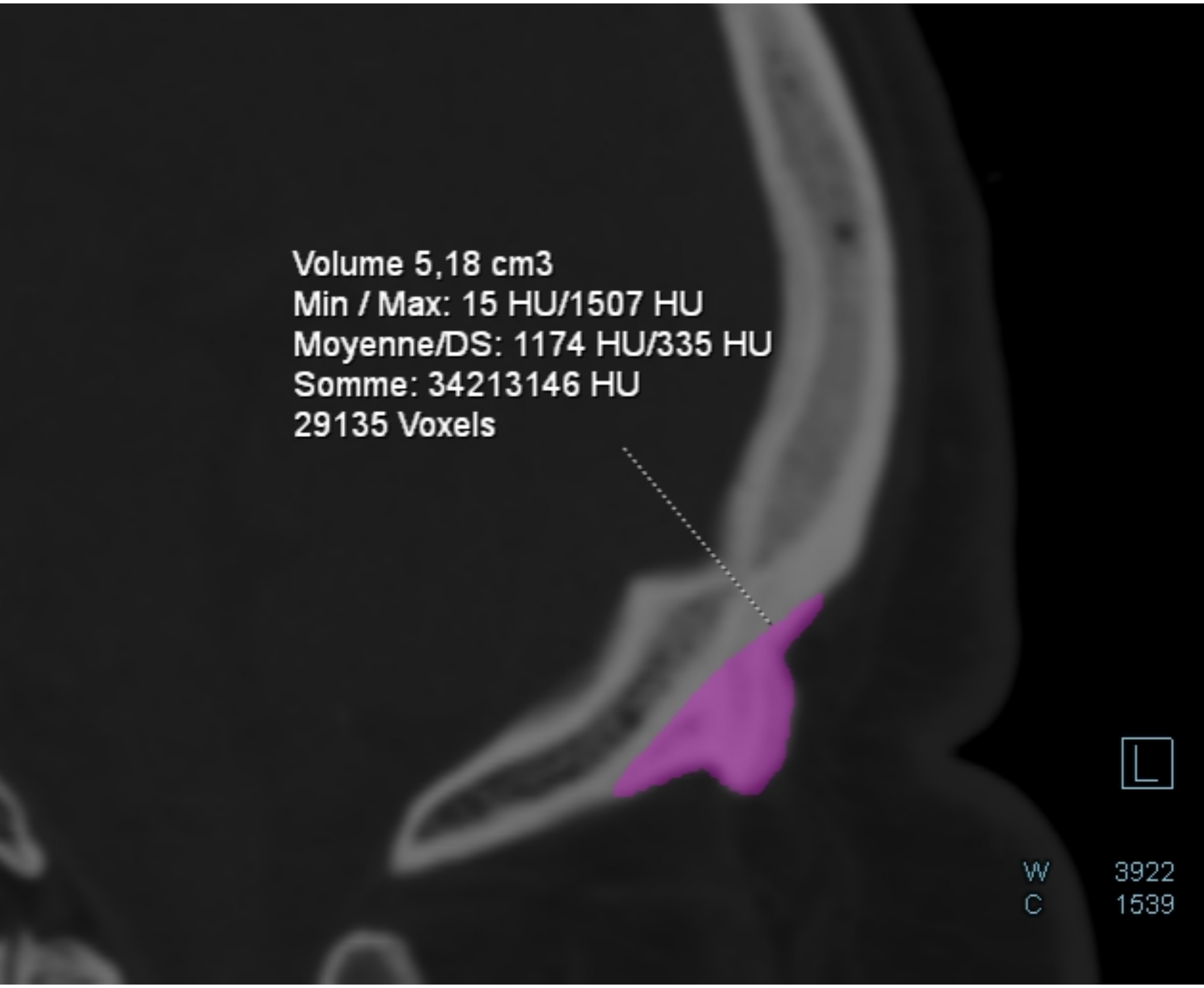
Volume 5,18 cm3
Min / Max: 15 HU/1507 HU
Moyenne/DS: 1174 HU/335 HU
Somme: 34213146 HU
29135 Voxels
L
W 3922
C 1539

11m7

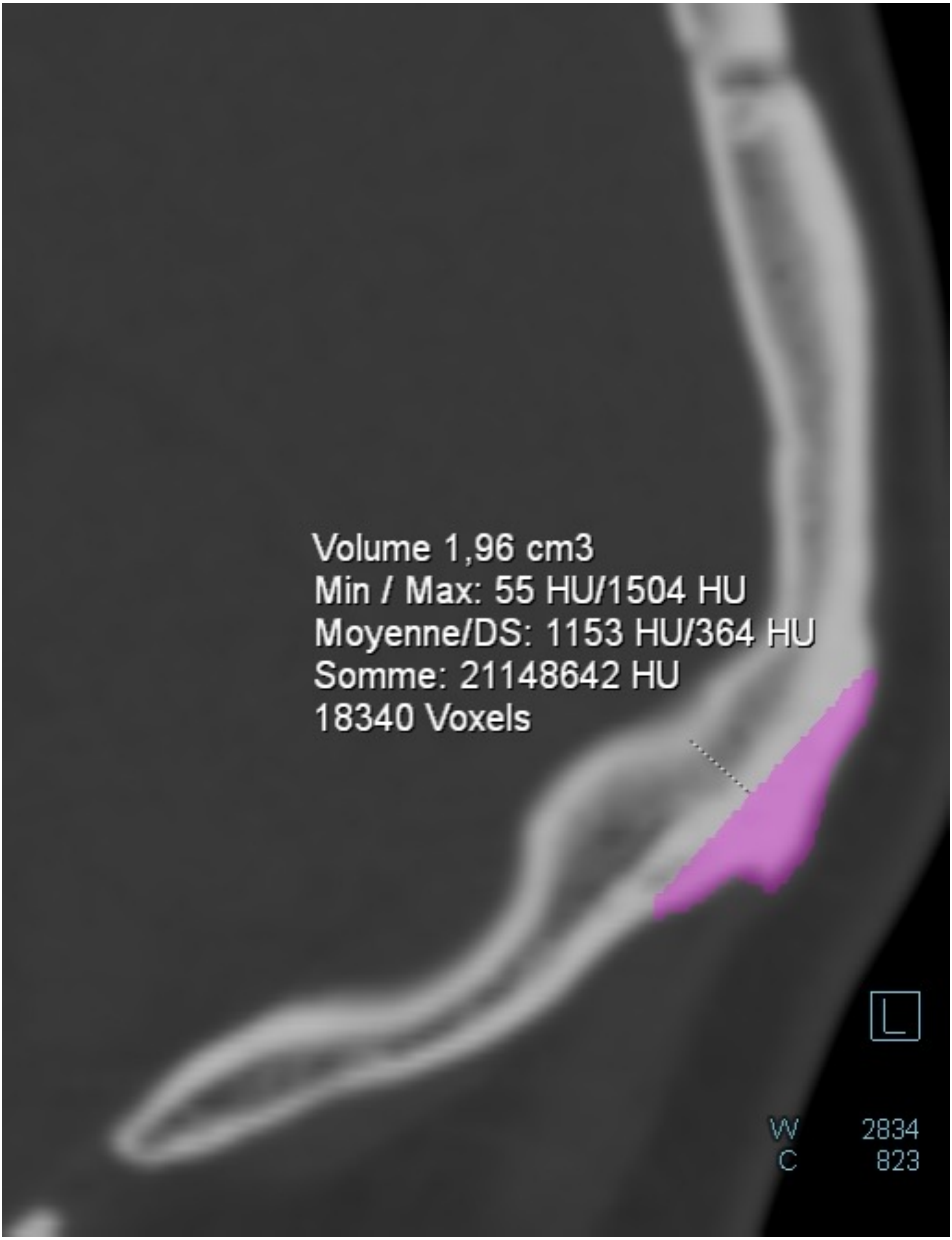
Volume 1,96 cm3
Min / Max: 55 HU/1504 HU
Moyenne/DS: 1153 HU/364 HU
Somme: 21148642 HU
18340 Voxels
L
W 2834
C 823

11m8

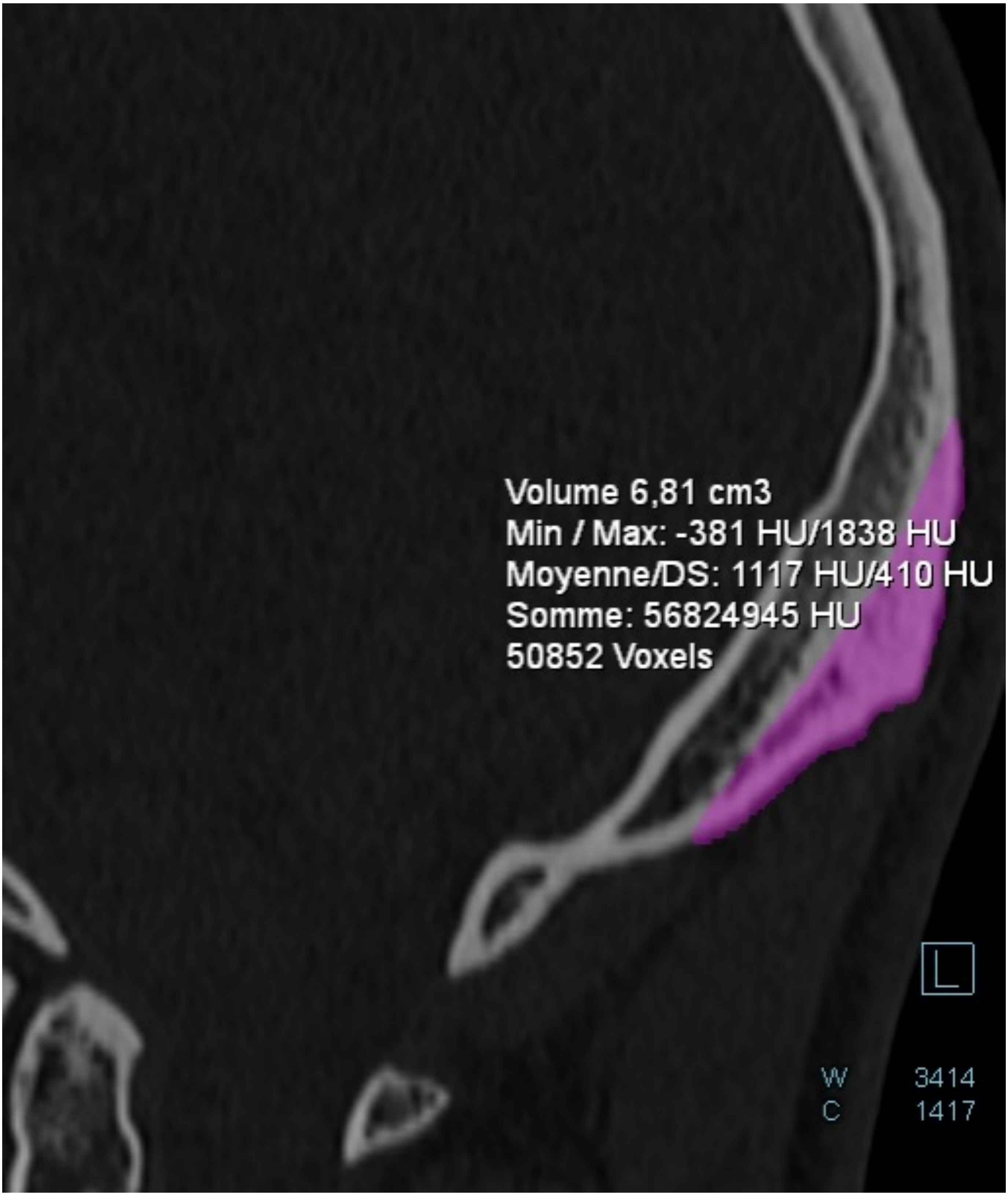
Volume 6,81 cm3
Min / Max: -381 HU/1838 HU
Moyenne/DS: 1117 HU/410 HU
Somme: 56824945 HU
50852 Voxels
L
W 3414
C 1417

11m9

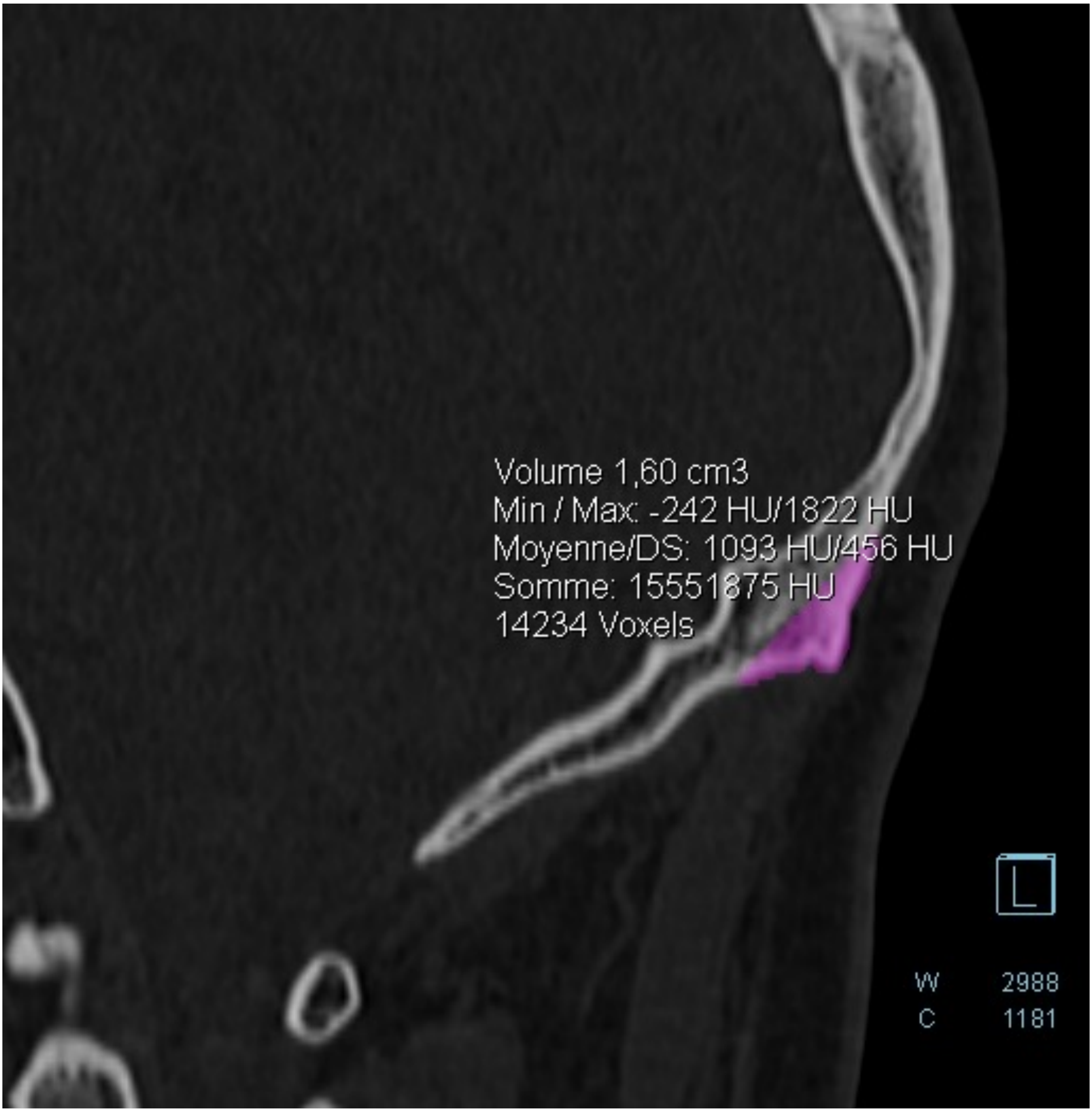
Volume 1,60 cm3
Min / Max: -242 HU/1822 HU
Moyenne/DS: 1093 HU/456 HU
Somme: 15551875 HU
14234 Voxels
L
W 2988
C 1181

11m10

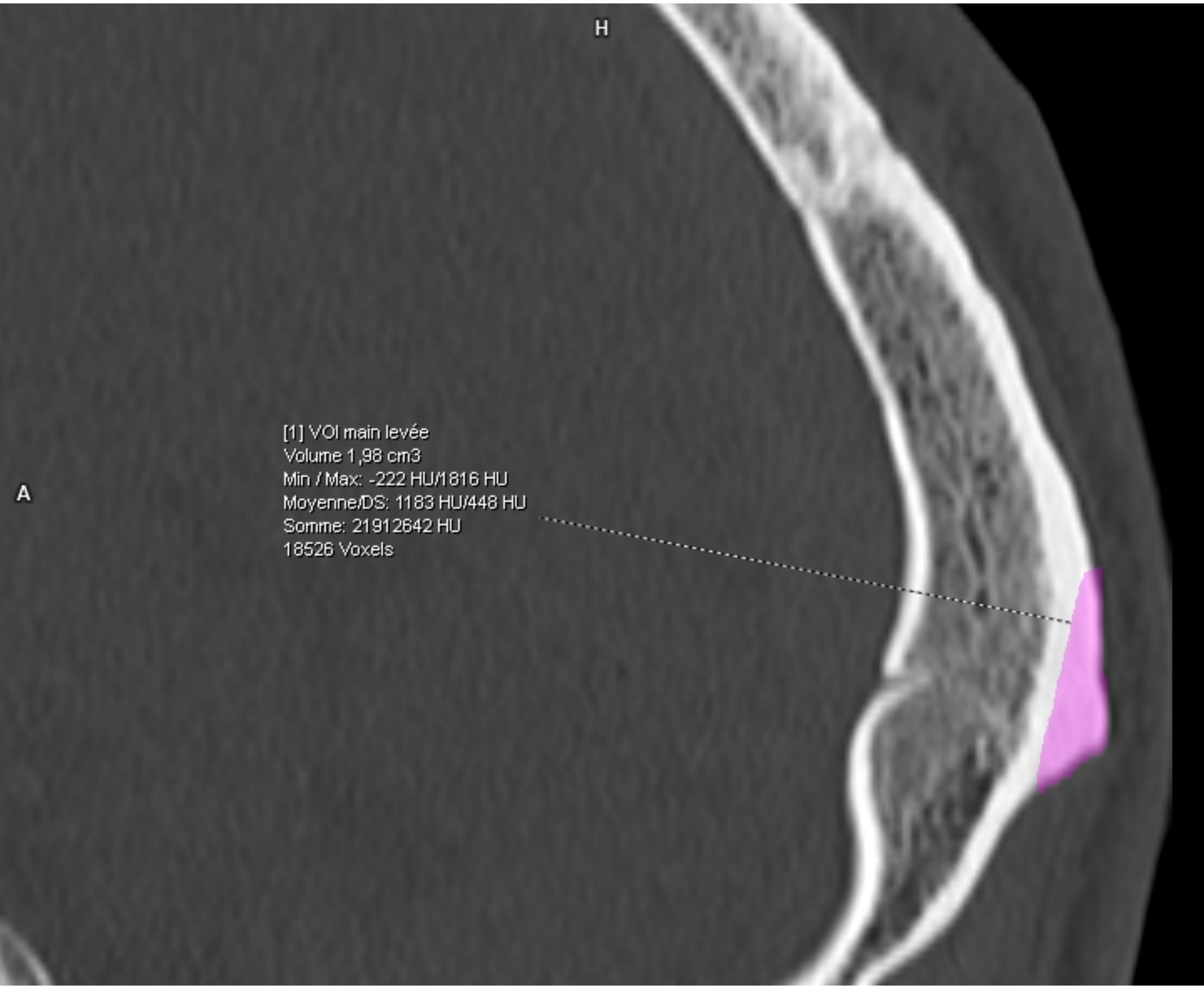
H
A
[1] VOI main levée
Volume 1,98 cm3
Min / Max: -222 HU/1816 HU
Moyenne/DS: 1183 HU/448 HU
Somme: 21912642 HU
18526 Voxels

11m11

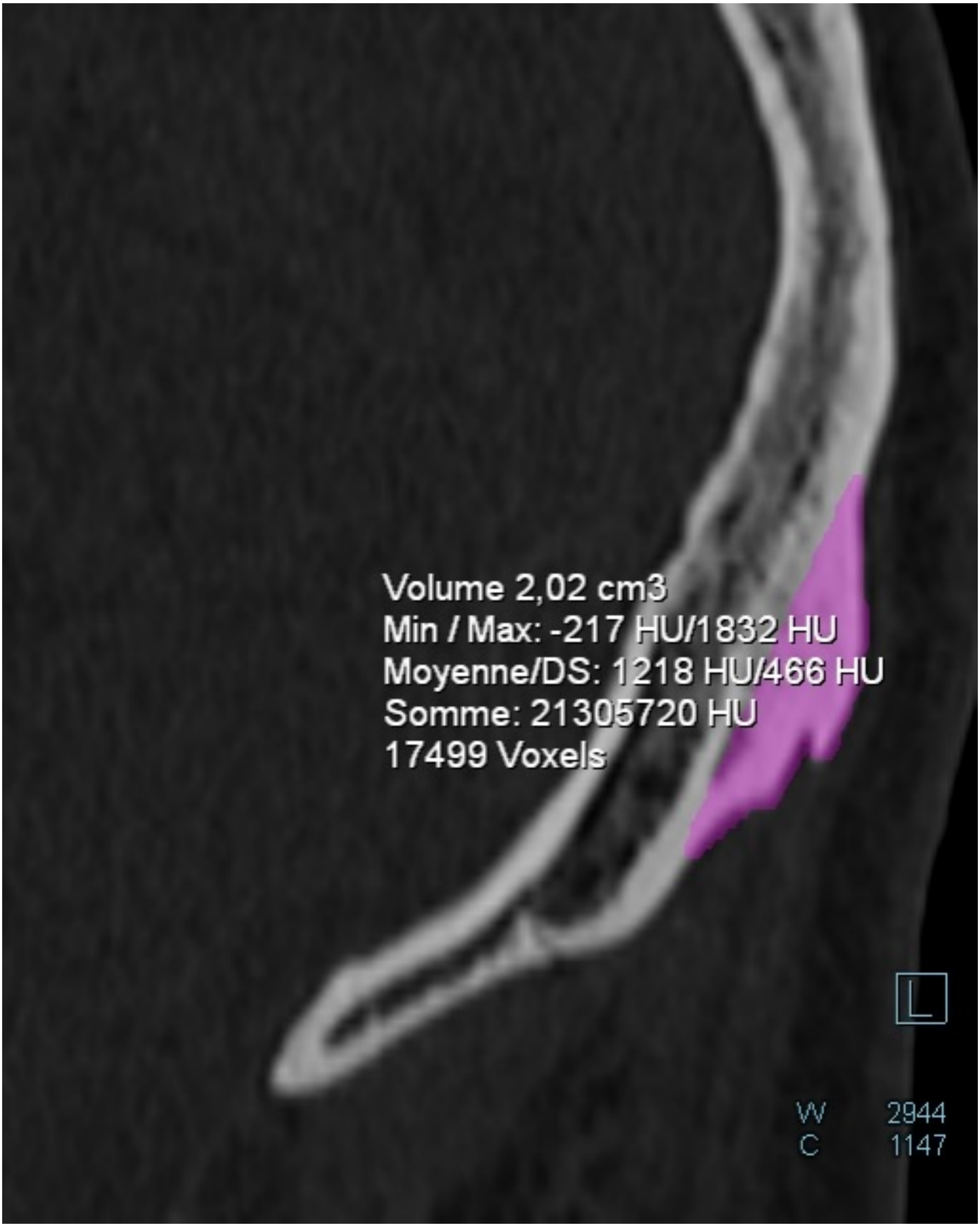

Volume 2,02 cm3
Min / Max: -217 HU/1832 HU
Moyenne/DS: 1218 HU/466 HU
Somme: 21305720 HU
17499 Voxels
L
W 2944
C 1147

11m12

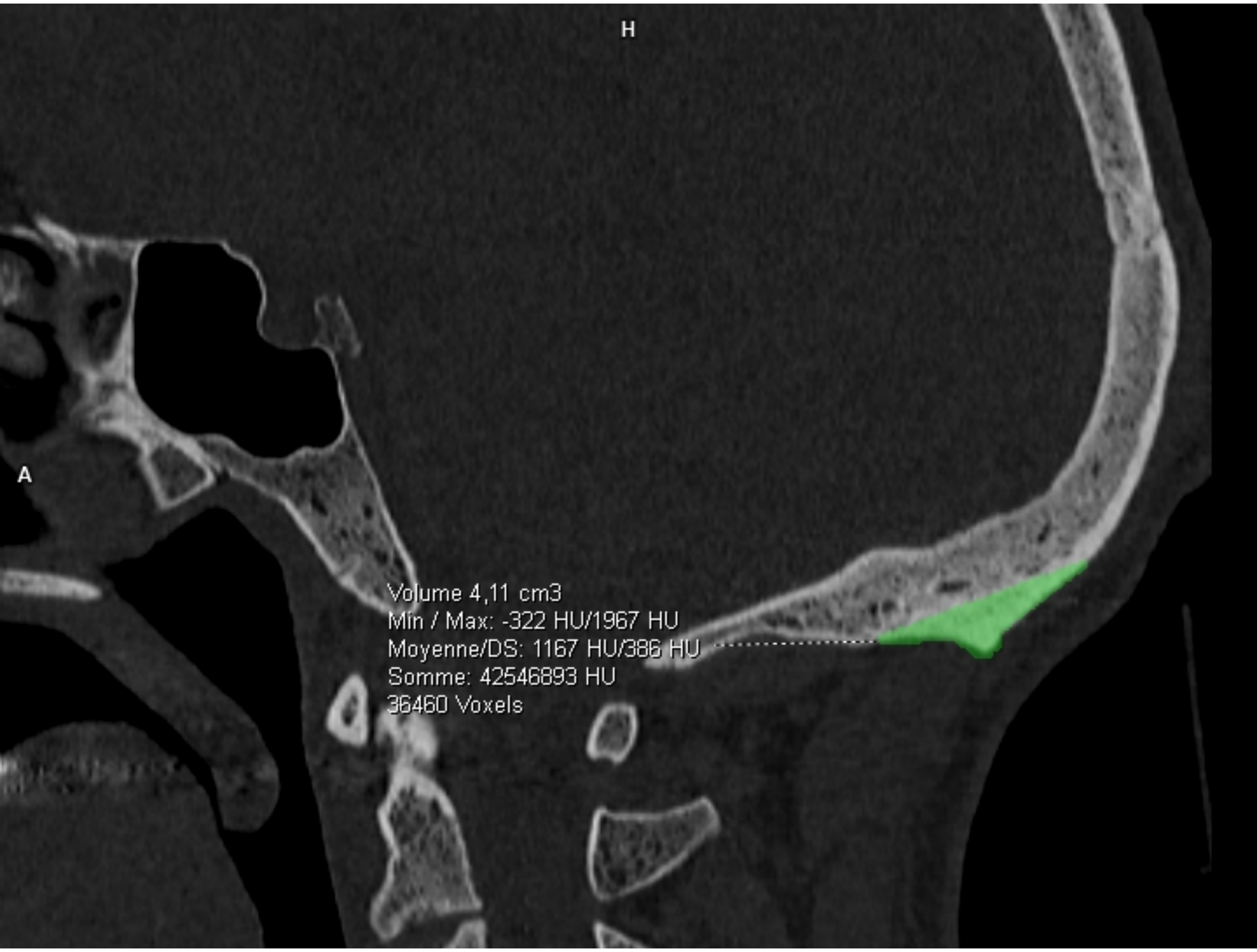
H
A
Volume 4,11 cm3
Min / Max: -322 HU/1967 HU
Moyenne/DS: 1167 HU/386 HU
Somme: 42546893 HU
36460 Voxels

11m13

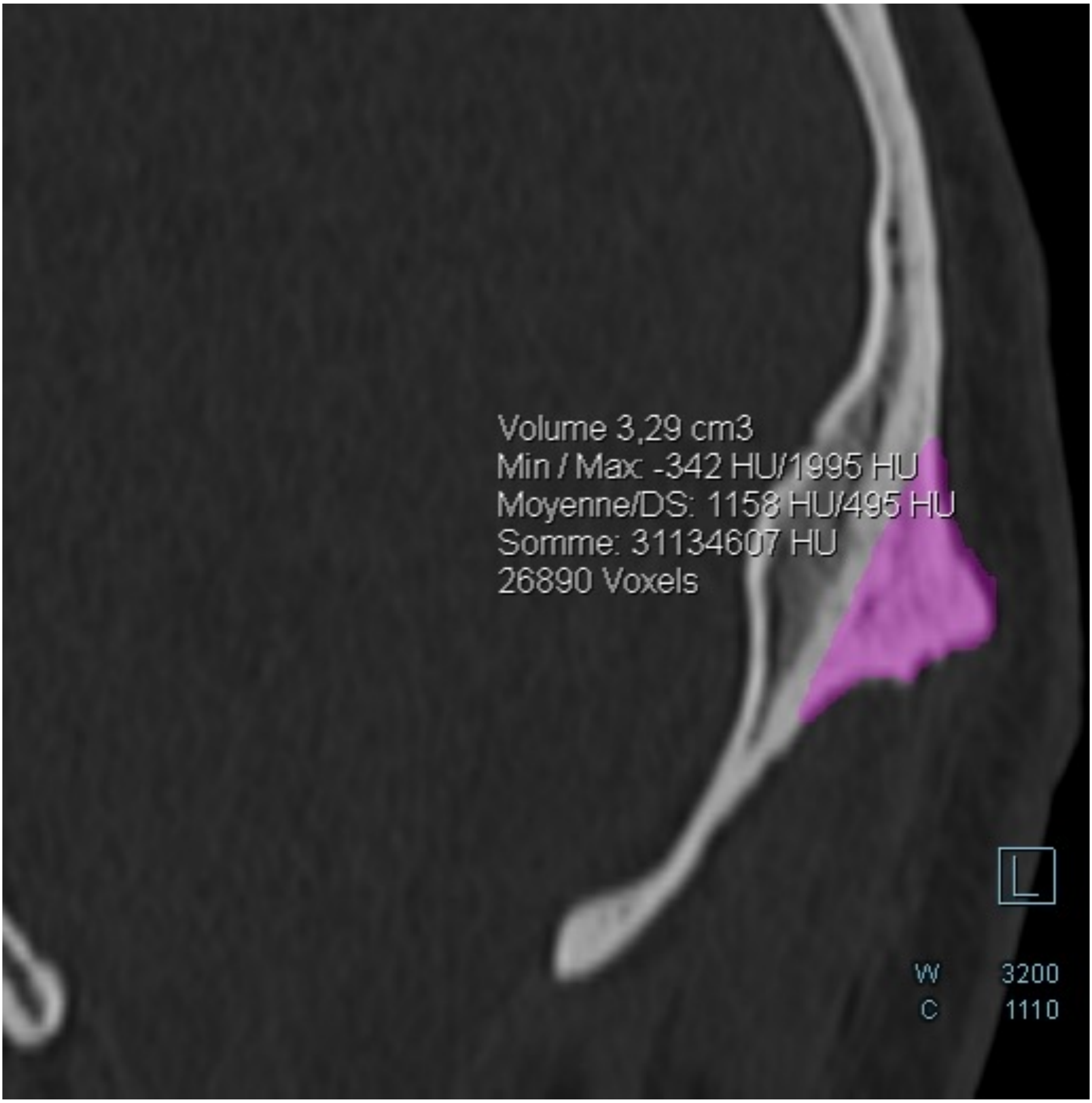
Volume 3,29 cm3
Min / Max: -342 HU/1995 HU
Moyenne/DS: 1158 HU/495 HU
Somme: 31134607 HU
26890 Voxels
L
W 3200
C 1110

11m14

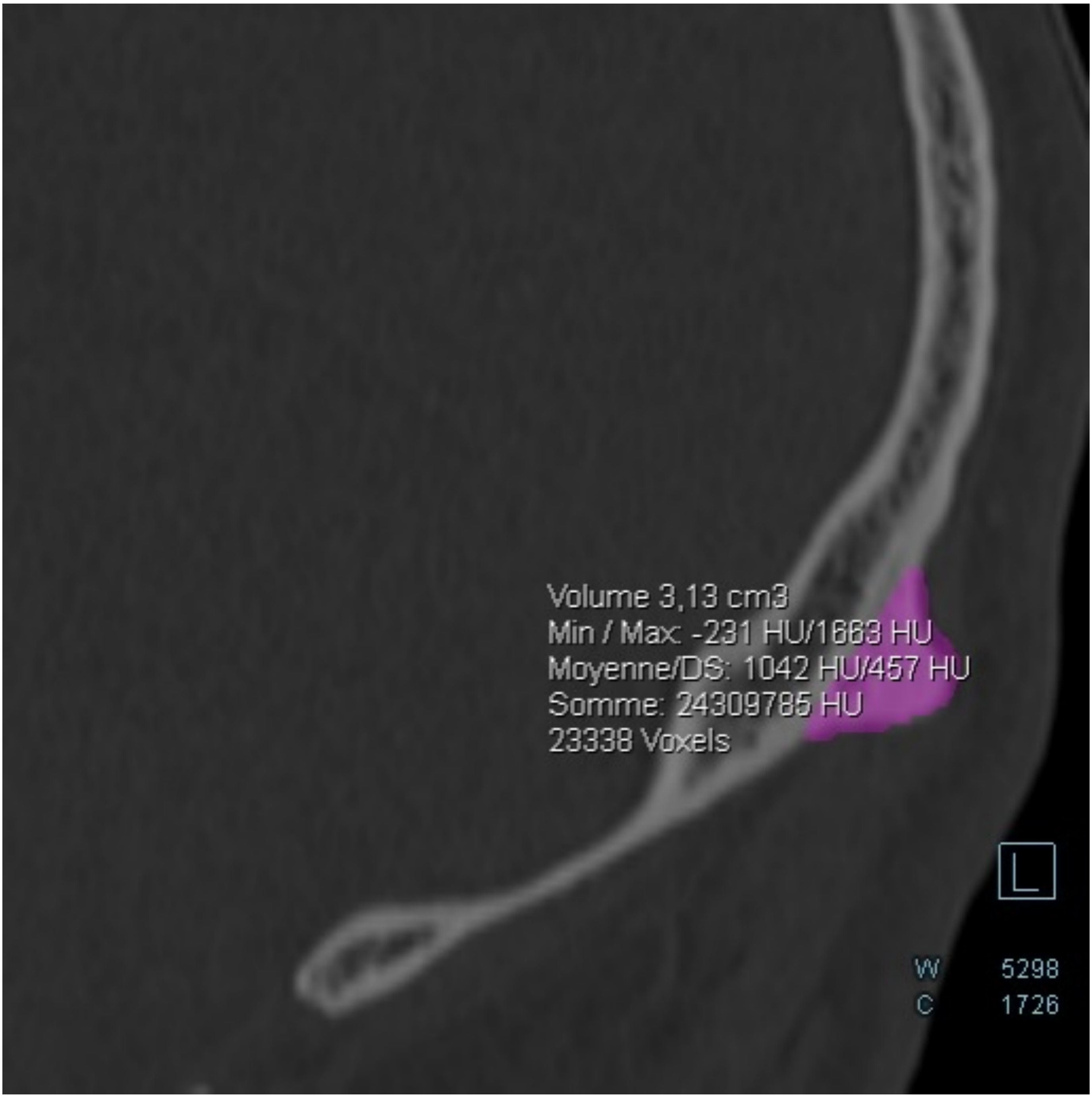
Volume 3,13 cm3
Min / Max: -231 HU/1663 HU
Moyenne/DS: 1042 HU/457 HU
Somme: 24309785 HU
23338 Voxels
L
W 5298
C 1726

11m15

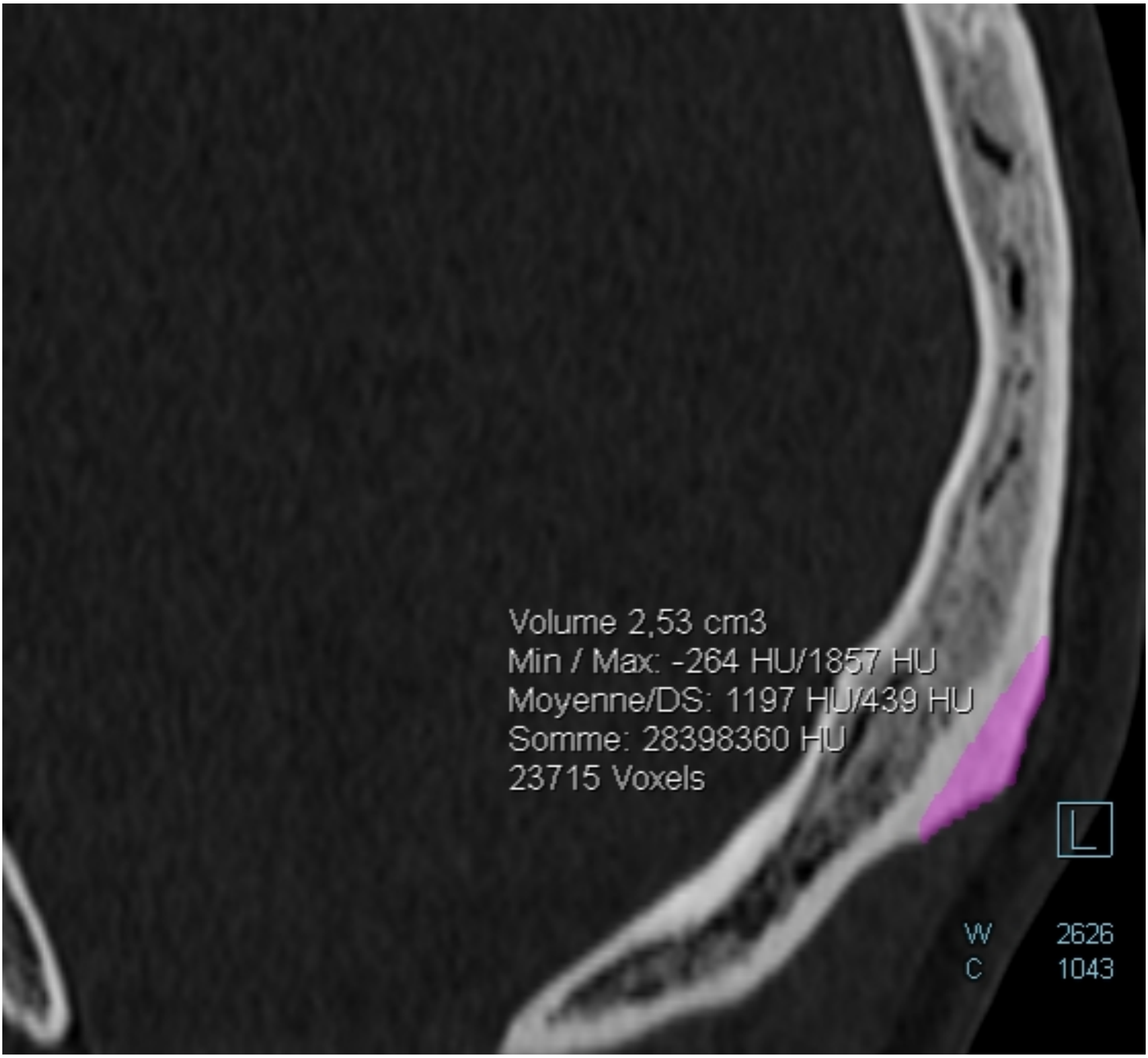
Volume 2,53 cm3
Min / Max: -264 HU/1857 HU
Moyenne/DS: 1197 HU/439 HU
Somme: 28398360 HU
23715 Voxels
L
W 2626
C 1043

11m16

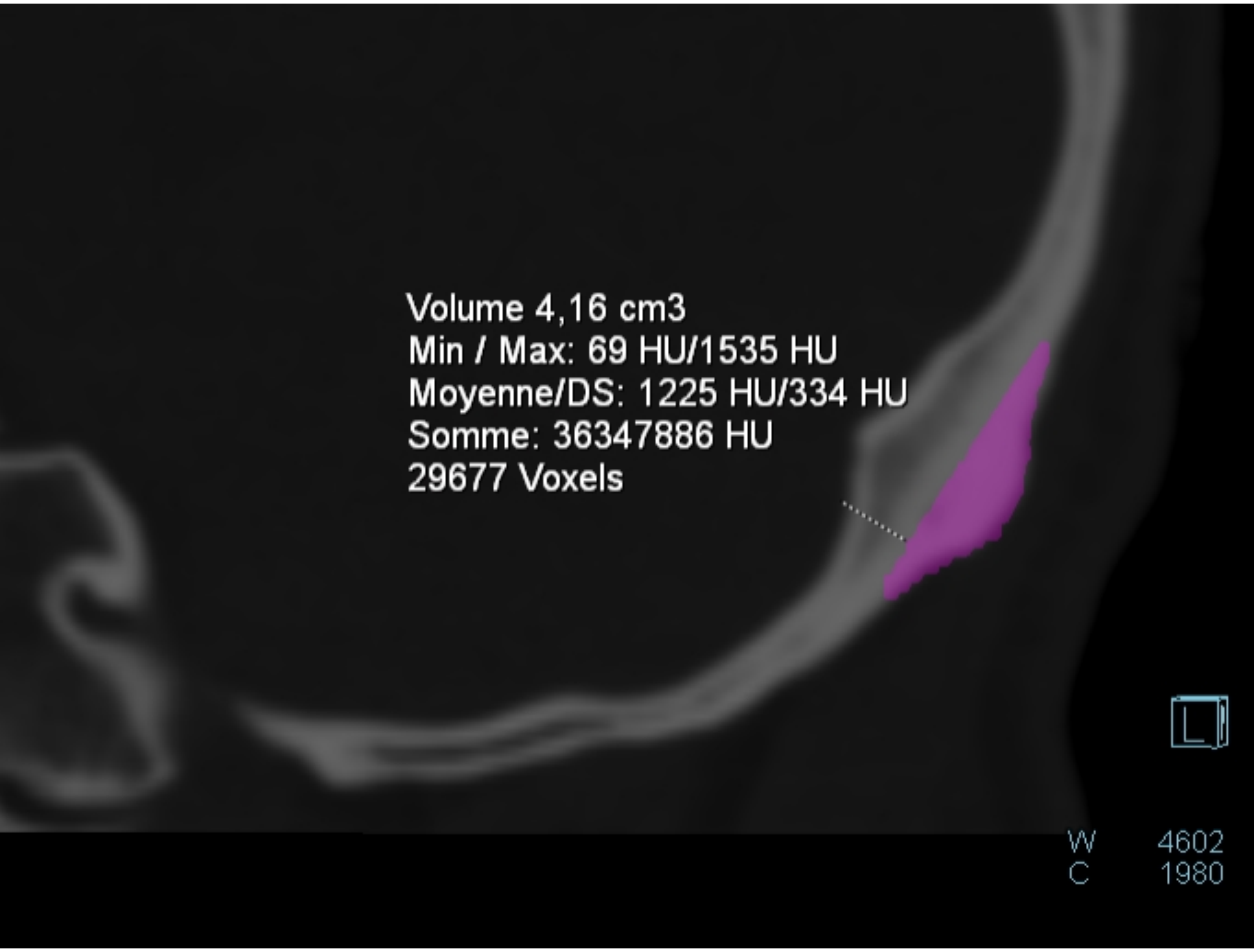
Volume 4,16 cm3
Min / Max: 69 HU/1535 HU
Moyenne/DS: 1225 HU/334 HU
Somme: 36347886 HU
29677 Voxels
L
W 4602
C 1980

11m17

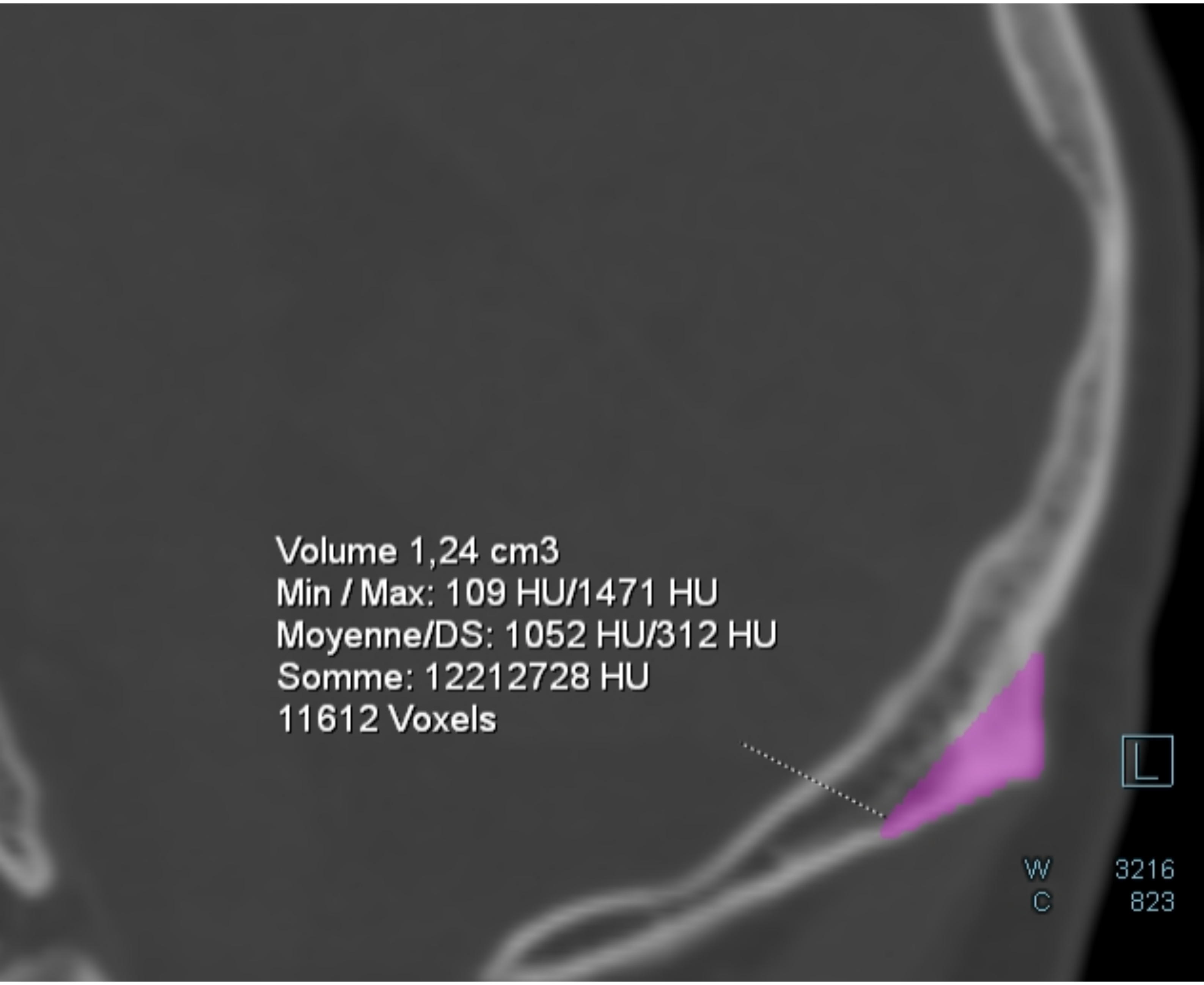
Volume 1,24 cm3
Min / Max: 109 HU/1471 HU
Moyenne/DS: 1052 HU/312 HU
Somme: 12212728 HU
11612 Voxels
L
W 3216
C 823

11m18

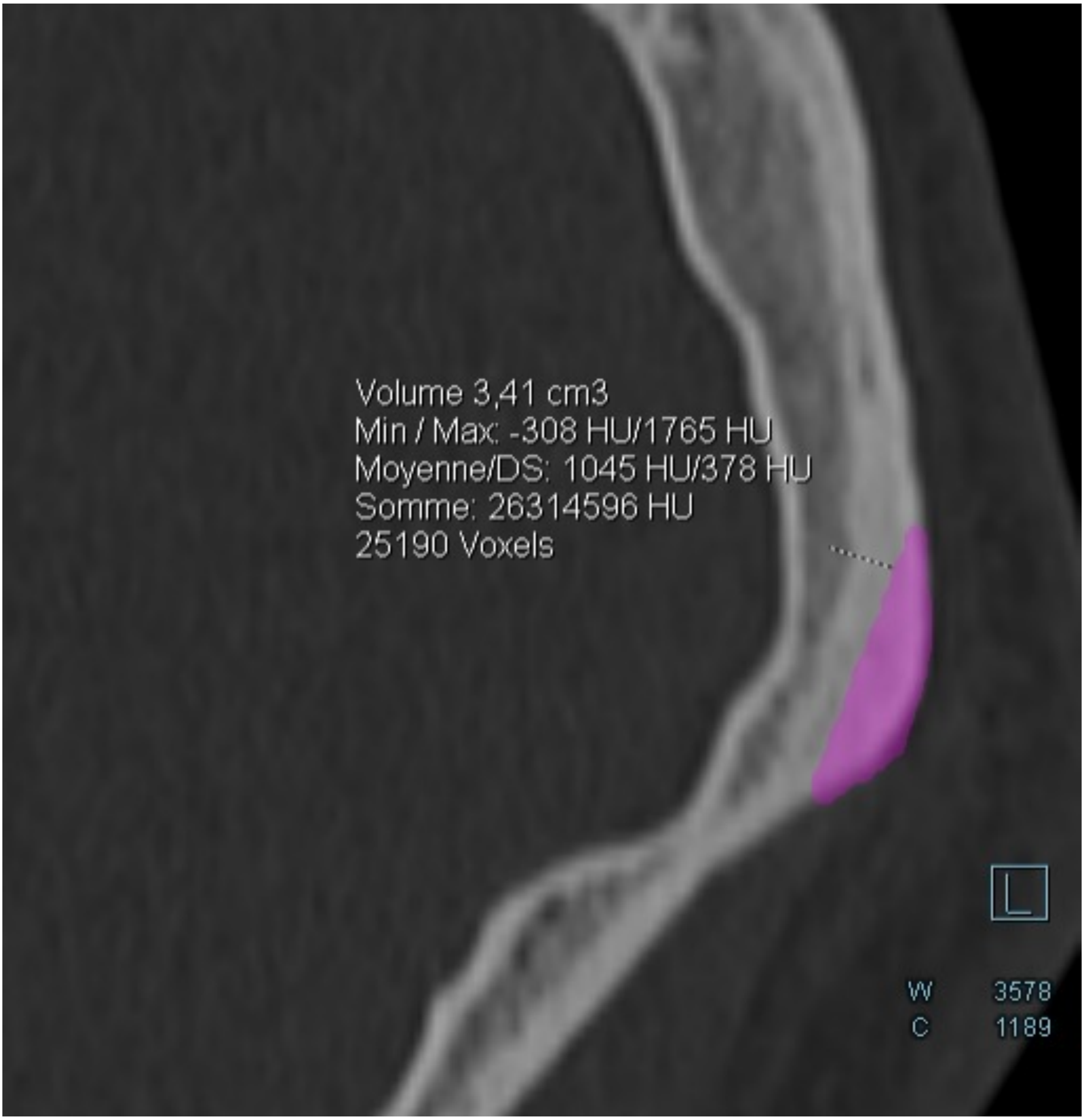

Volume 3,41 cm3
Min / Max: -308 HU/1765 HU
Moyenne/DS: 1045 HU/378 HU
Somme: 26314596 HU
25190 Voxels
L
W 3578
C 1189

11m19

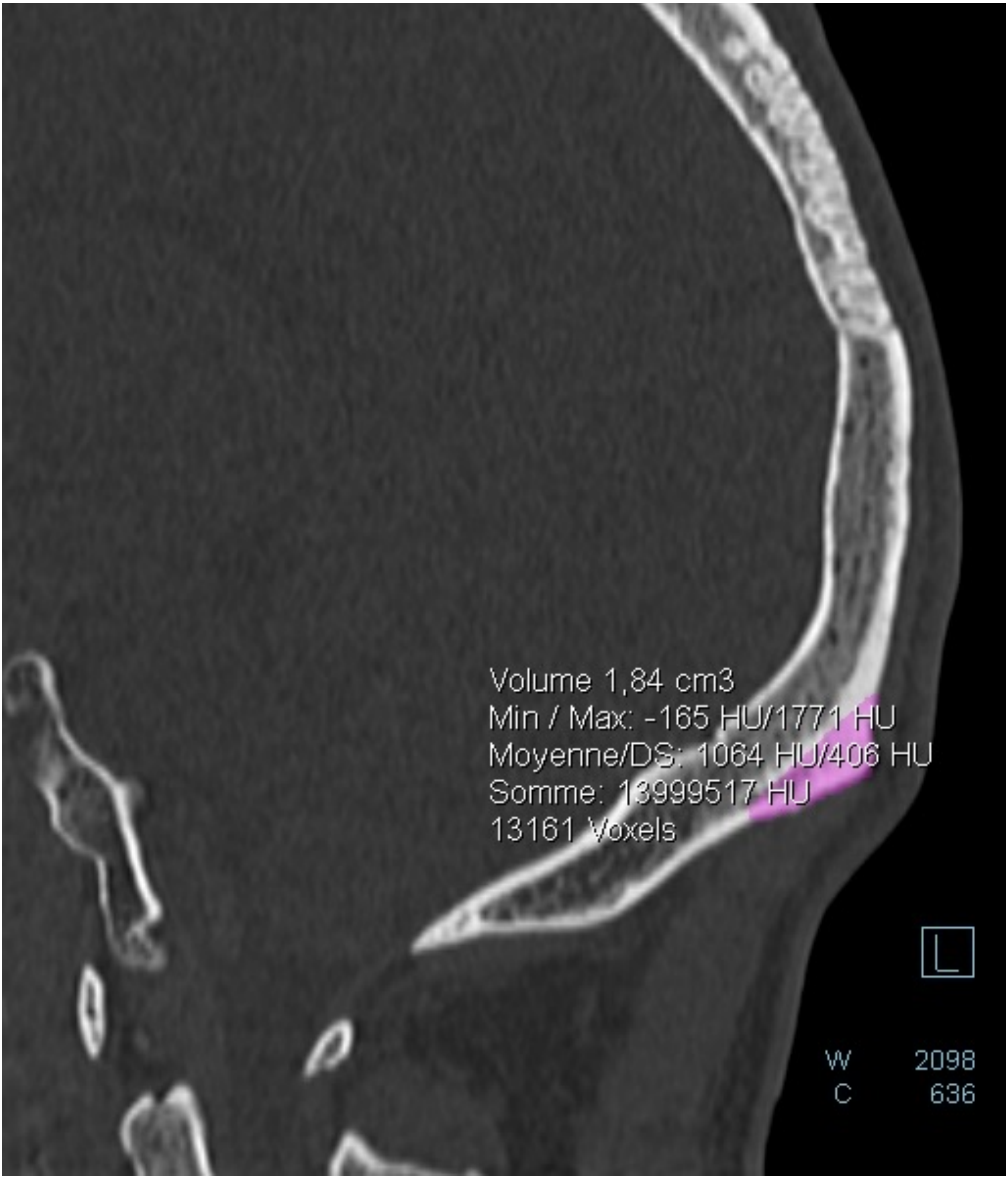
Volume 1,84 cm3
Min / Max: -165 HU/1771 HU
Moyenne/DS: 1064 HU/406 HU
Somme: 13999517 HU
13161 Voxels
L
W 2098
C 636

11m20

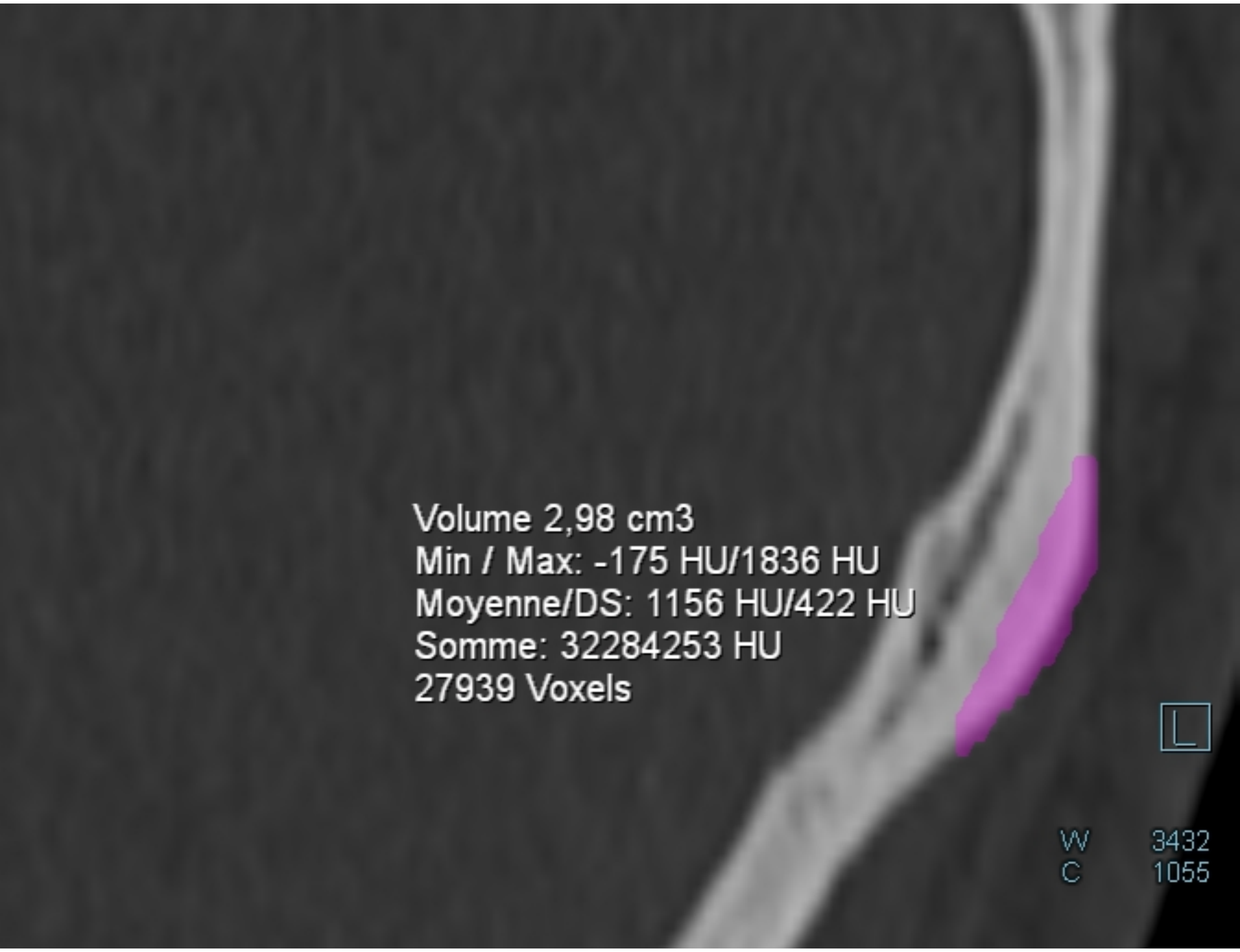

Volume 2,98 cm3
Min / Max: -175 HU/1836 HU
Moyenne/DS: 1156 HU/422 HU
Somme: 32284253 HU
27939 Voxels
L
W 3432
C 1055

11m21

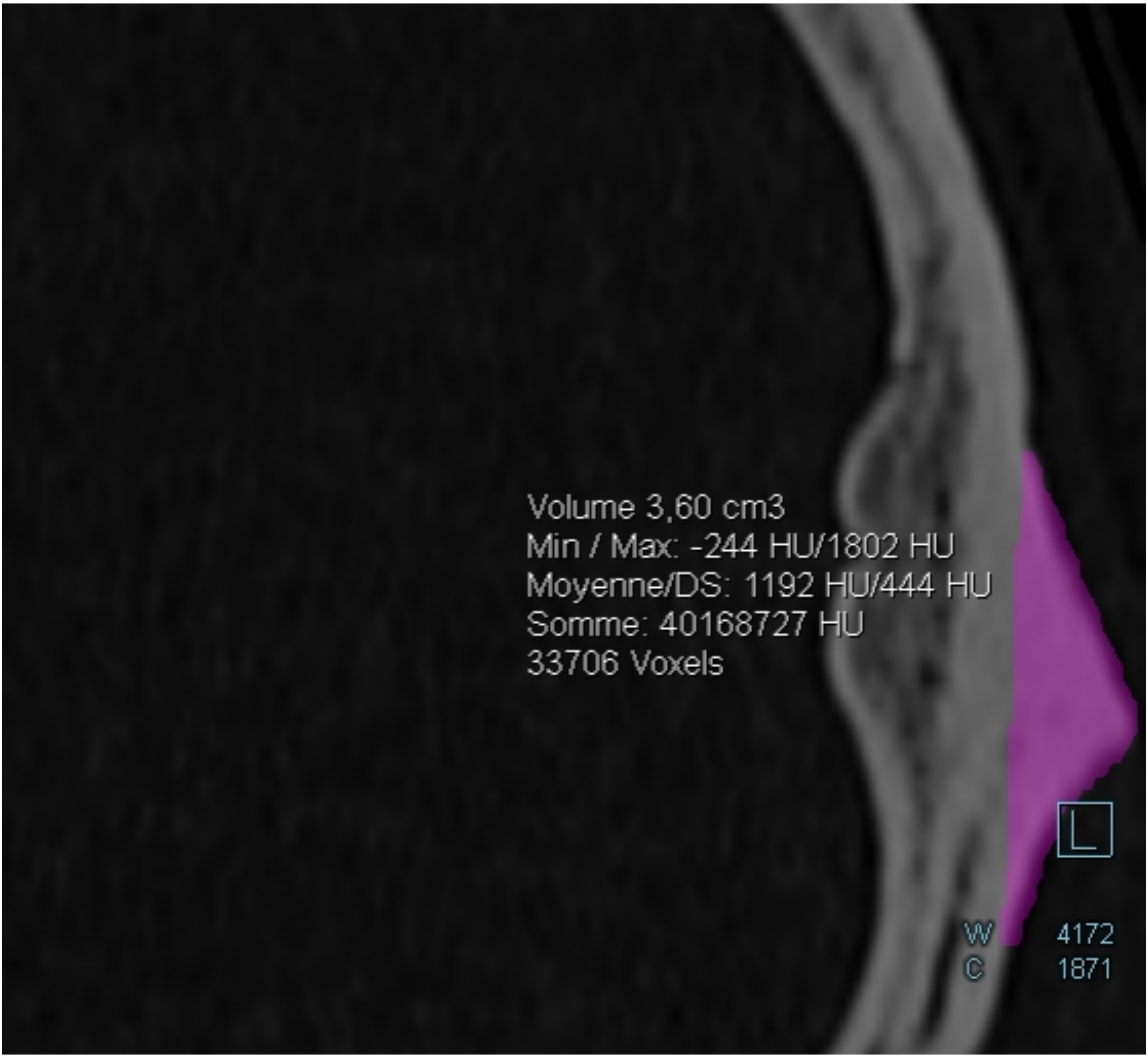

Volume 3,60 cm3
Min / Max: -244 HU/1802 HU
Moyenne/DS: 1192 HU/444 HU
Somme: 40168727 HU
33706 Voxels
L
W 4172
C 1871

11m22

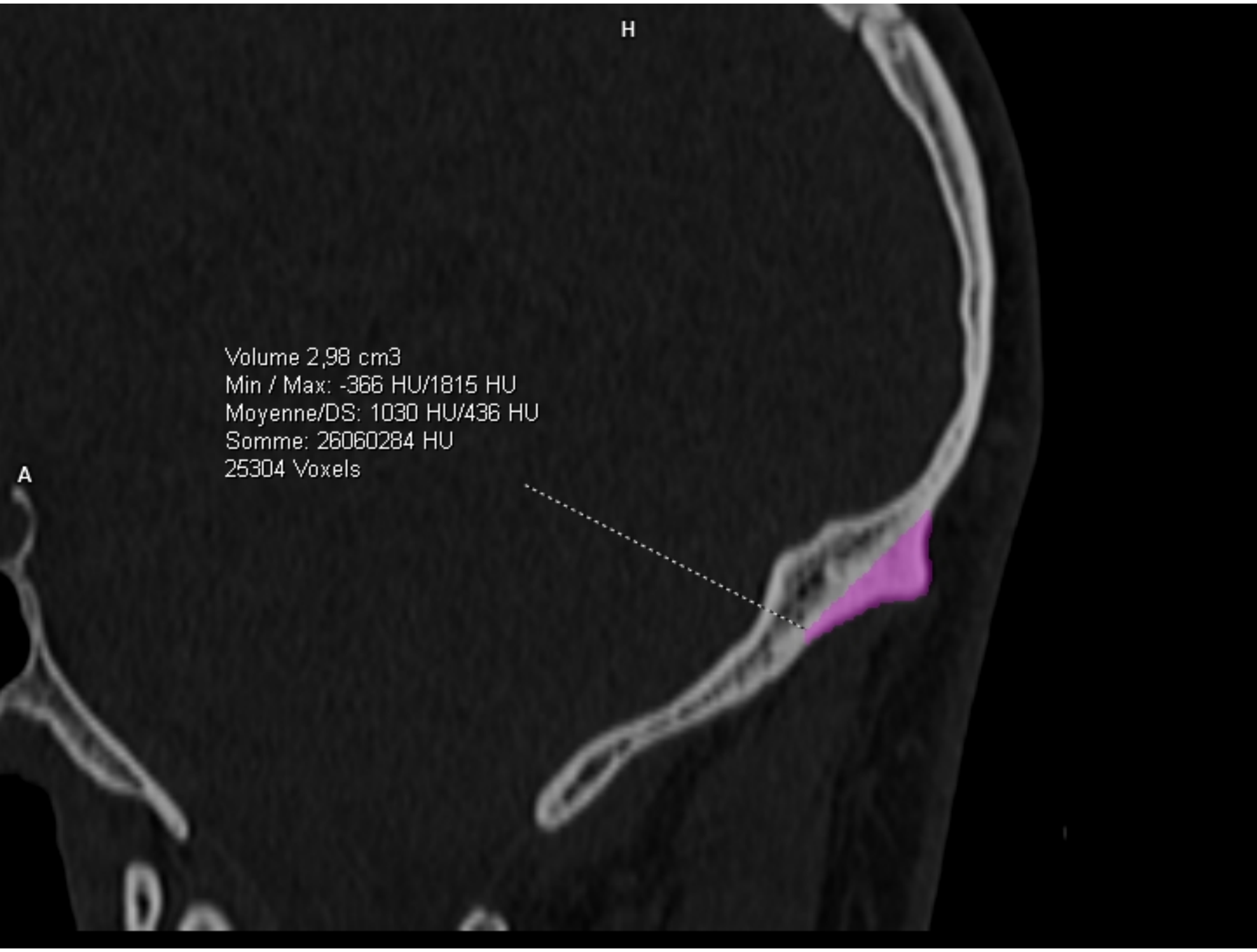
H
A
Volume 2,98 cm3
Min / Max: -366 HU/1815 HU
Moyenne/DS: 1030 HU/436 HU
Somme: 26060284 HU
25304 Voxels

11m23

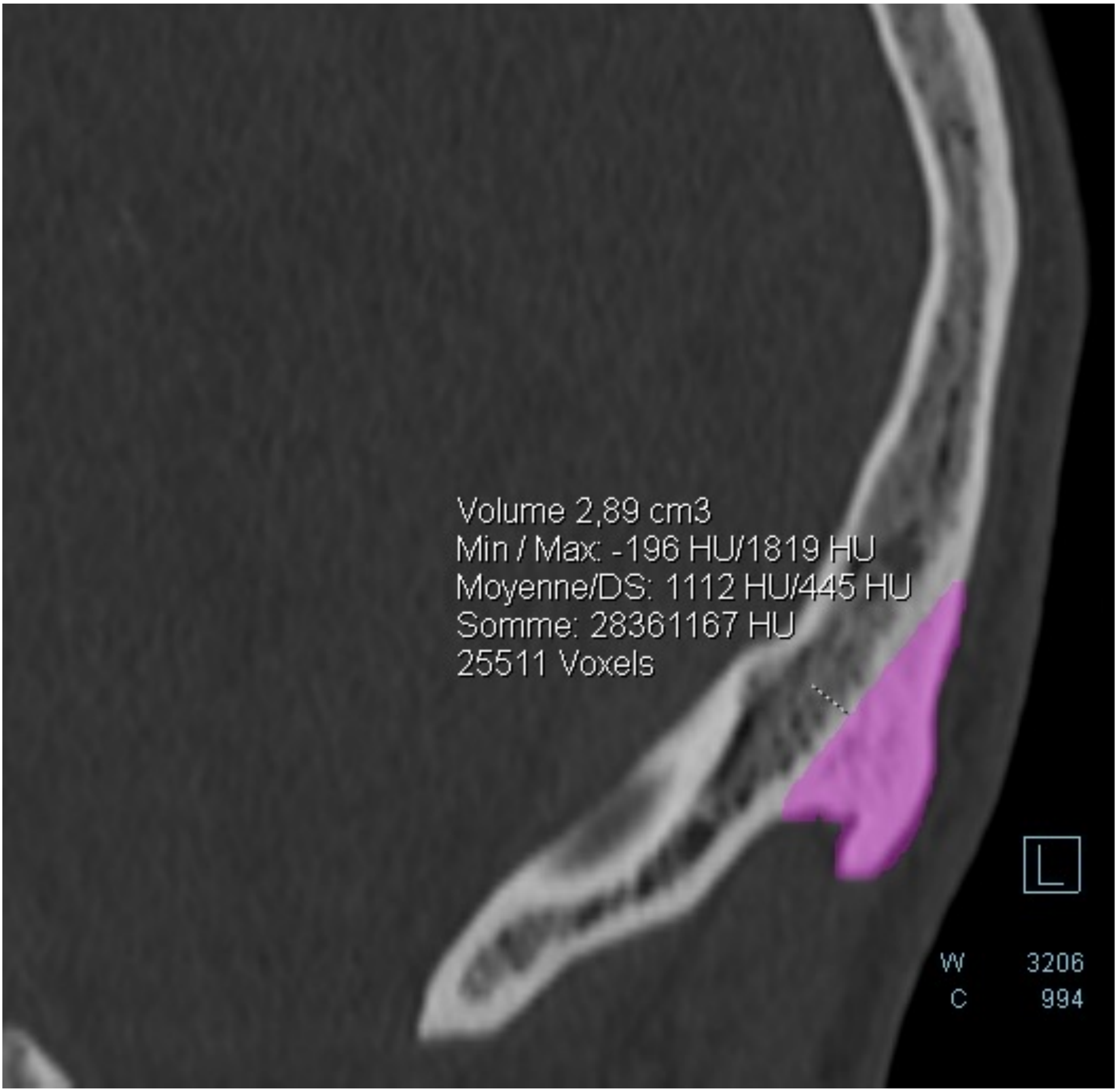
Volume 2,89 cm3
Min / Max: -196 HU/1819 HU
Moyenne/DS: 1112 HU/445 HU
Somme: 28361167 HU
25511 Voxels
L
W 3206
C 994

11m24

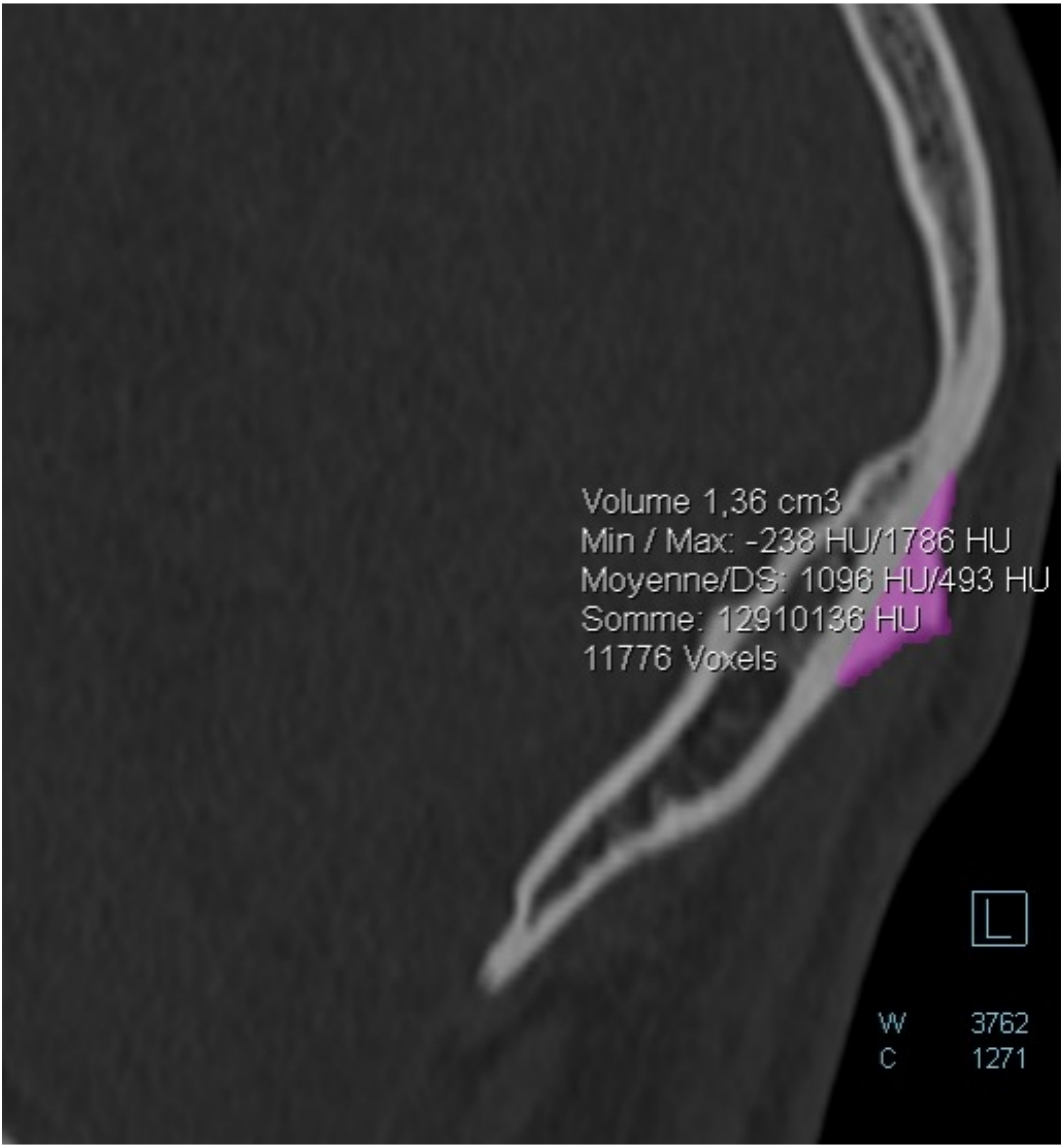

Volume 1,36 cm3
Min / Max: -238 HU/1786 HU
Moyenne/DS: 1096 HU/493 HU
Somme: 12910136 HU
11776 Voxels
L
W 3762
C 1271

11m25

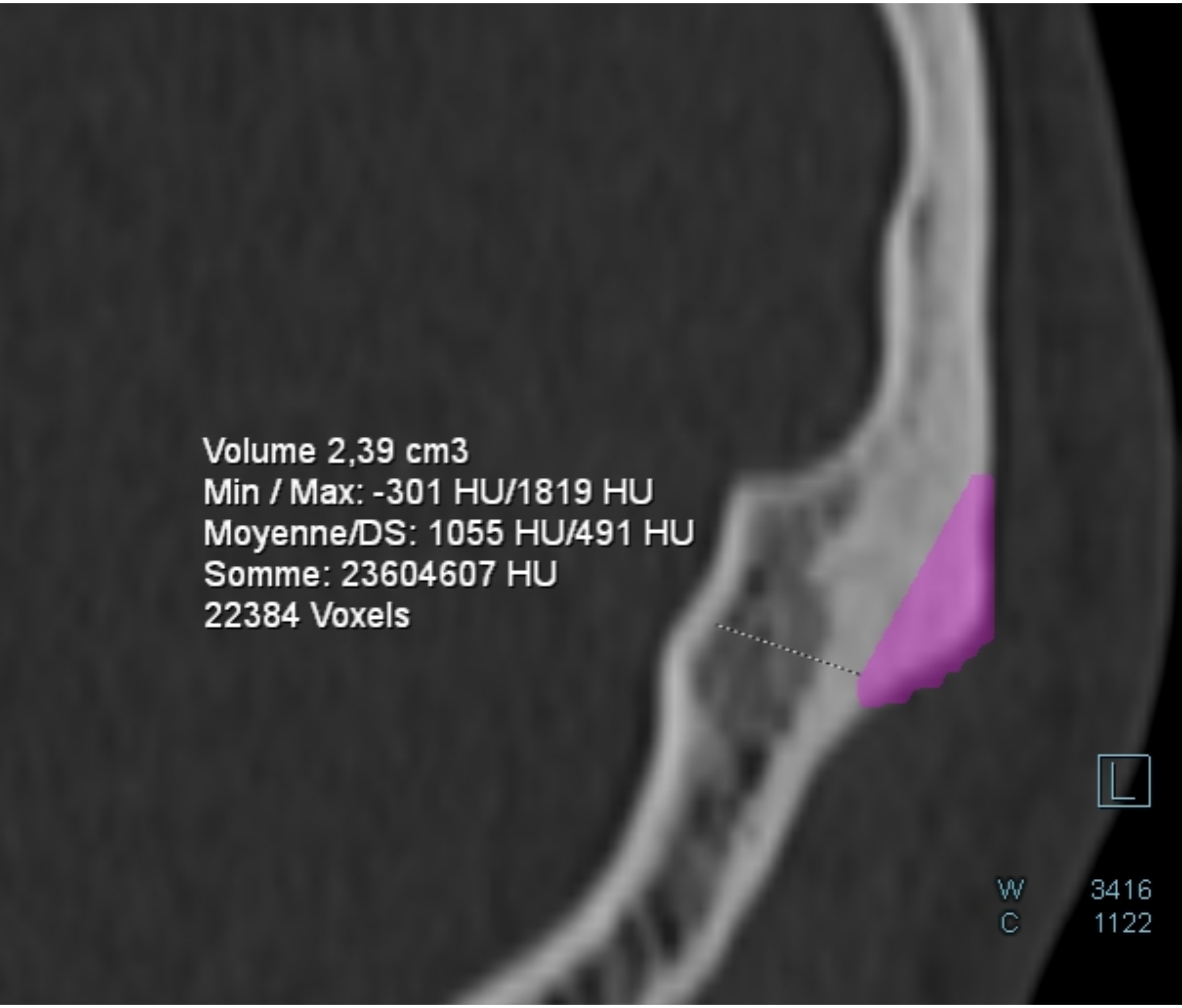
Volume 2,39 cm3
Min / Max: -301 HU/1819 HU
Moyenne/DS: 1055 HU/491 HU
Somme: 23604607 HU
22384 Voxels
L
W 3416
C 1122

11m26

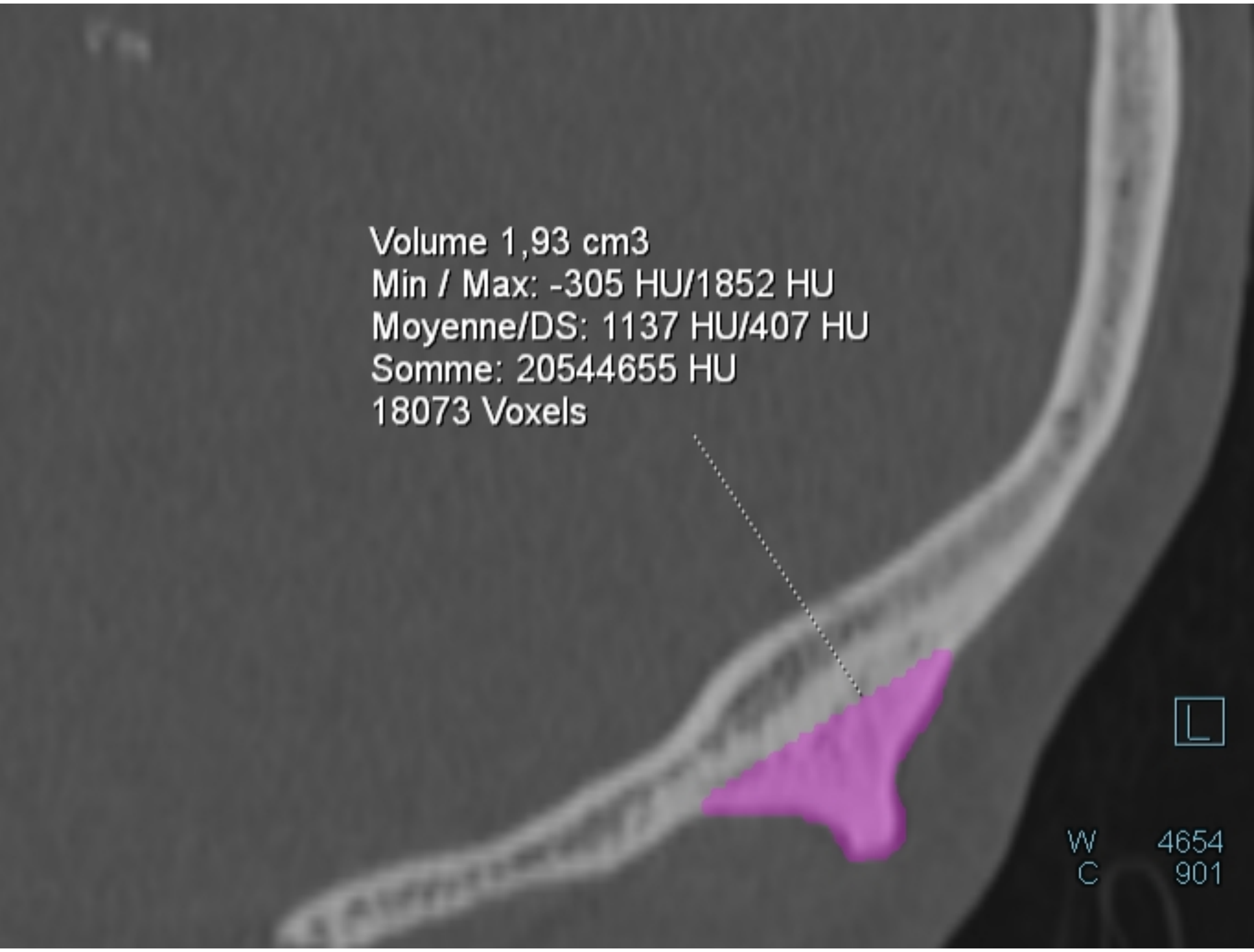
Volume 1,93 cm3
Min / Max: -305 HU/1852 HU
Moyenne/DS: 1137 HU/407 HU
Somme: 20544655 HU
18073 Voxels
L
W 4654
C 901

11m27

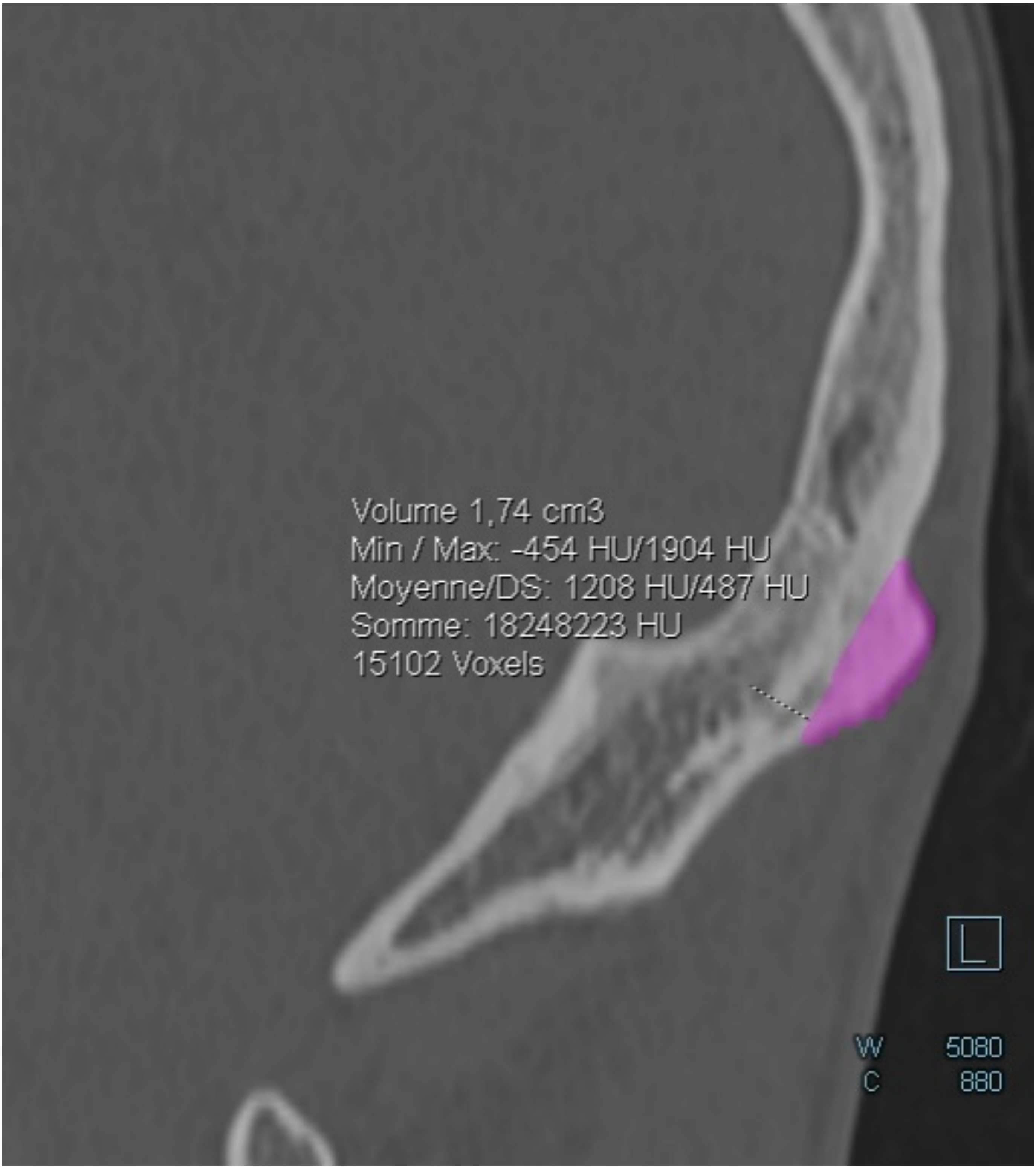
Volume 1,74 cm3
Min / Max: -454 HU/1904 HU
Moyenne/DS: 1208 HU/487 HU
Somme: 18248223 HU
15102 Voxels
L
W 5080
C 880

11m28

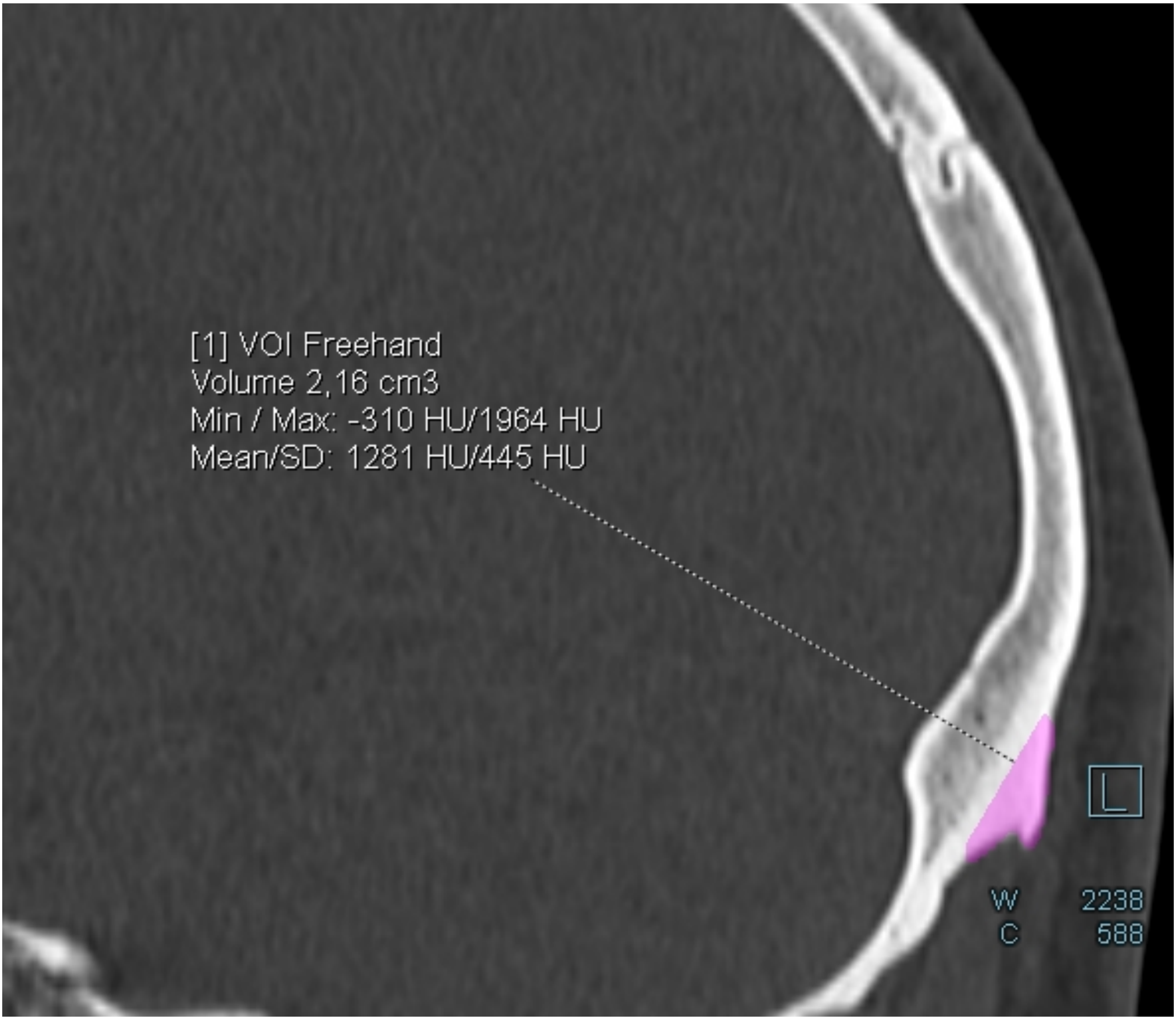
[1] VOI Freehand
Volume 2,16 cm3
Min / Max: -310 HU/1964 HU
Mean/SD: 1281 HU/445 HU
L
W 2238
C 588

11m29

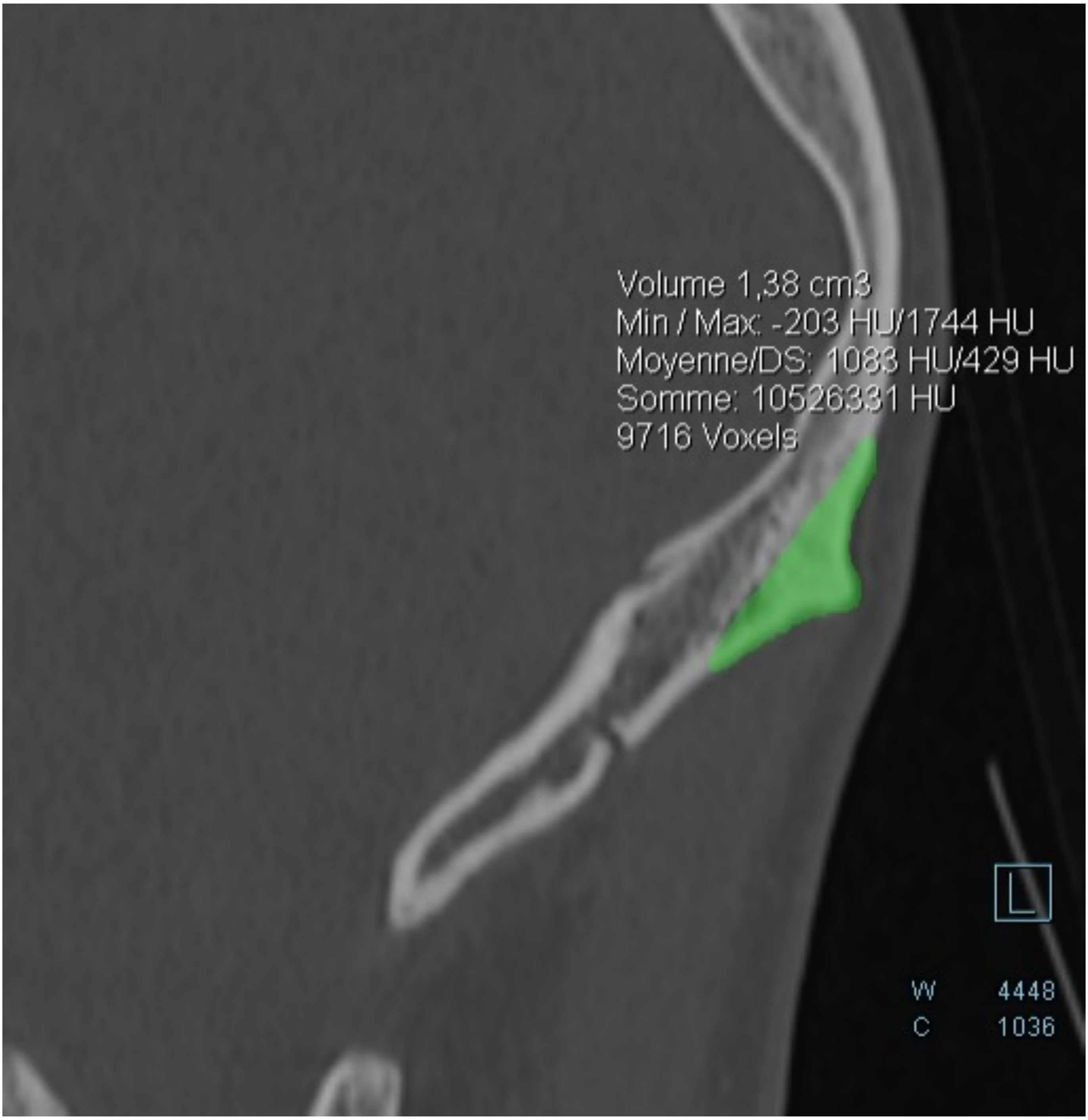
Volume 1,38 cm3
Min / Max: -203 HU/1744 HU
Moyenne/DS: 1083 HU/429 HU
Somme: 10526331 HU
9716 Voxels
L
W 4448
C 1036

11m30

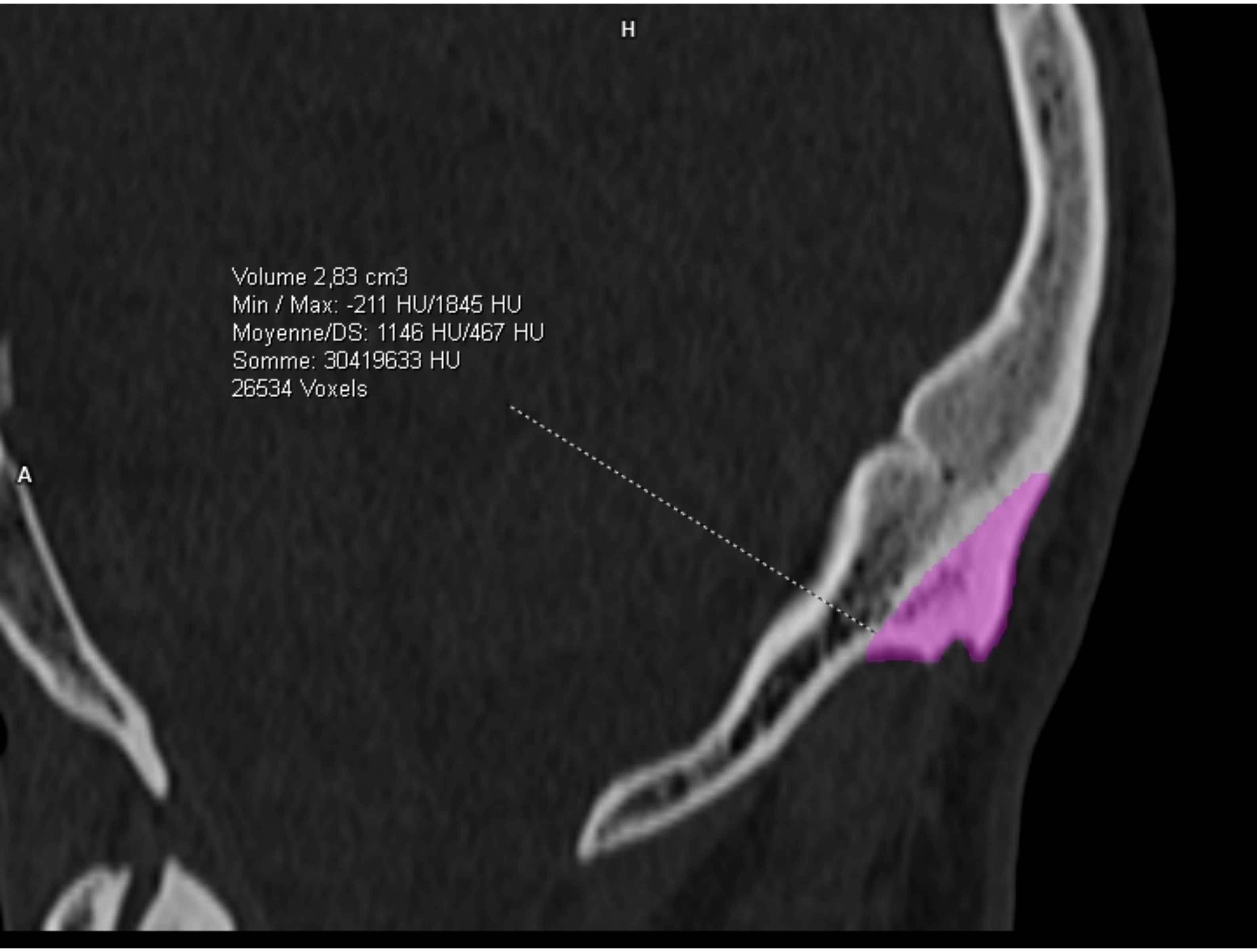
H
A
Volume 2,83 cm3
Min / Max: -211 HU/1845 HU
Moyenne/DS: 1146 HU/467 HU
Somme: 30419633 HU
26534 Voxels

11m31

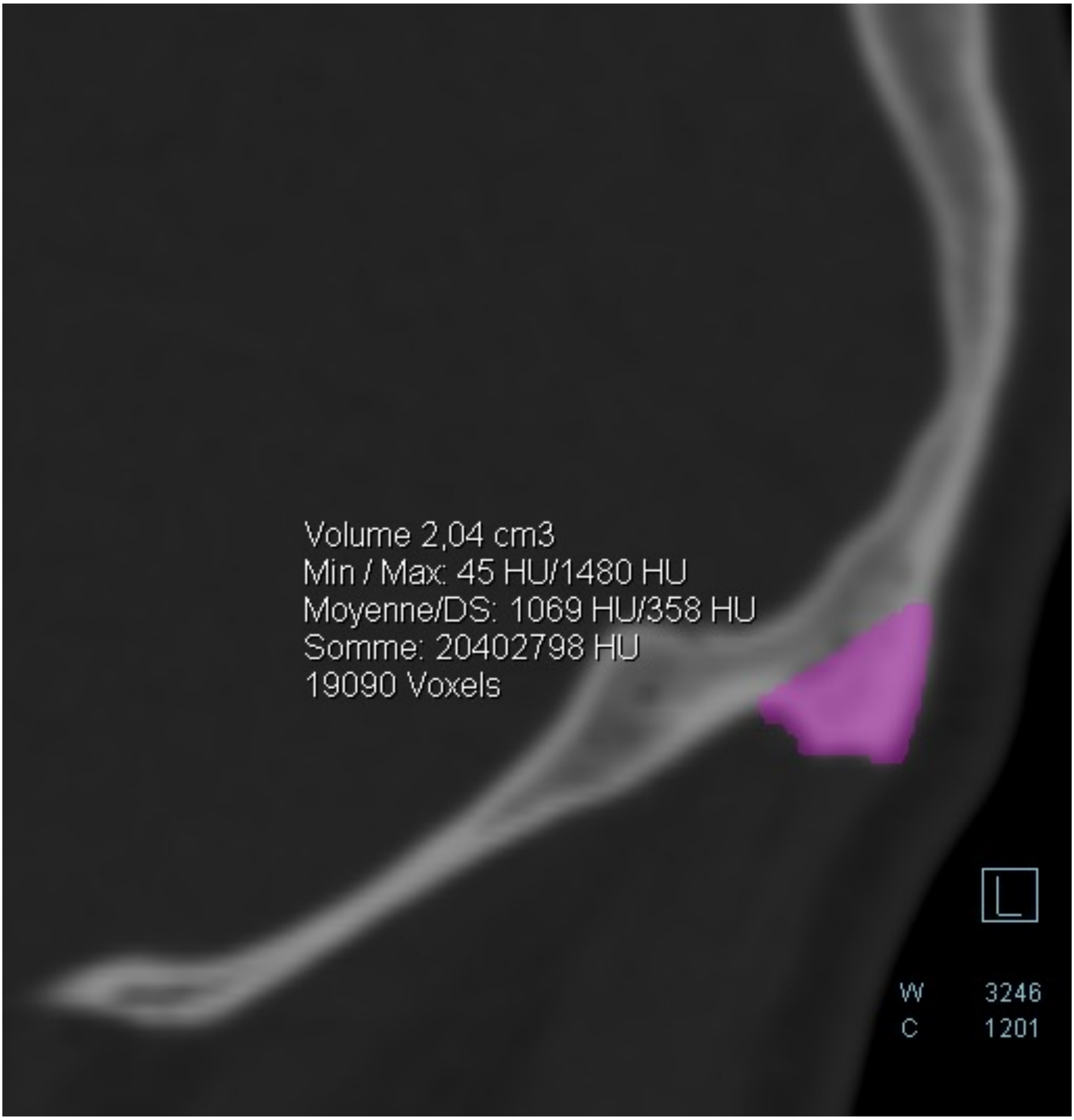
Volume 2,04 cm3
Min / Max: 45 HU/1480 HU
Moyenne/DS: 1069 HU/358 HU
Somme: 20402798 HU
19090 Voxels
L
W 3246
C 1201

11m32

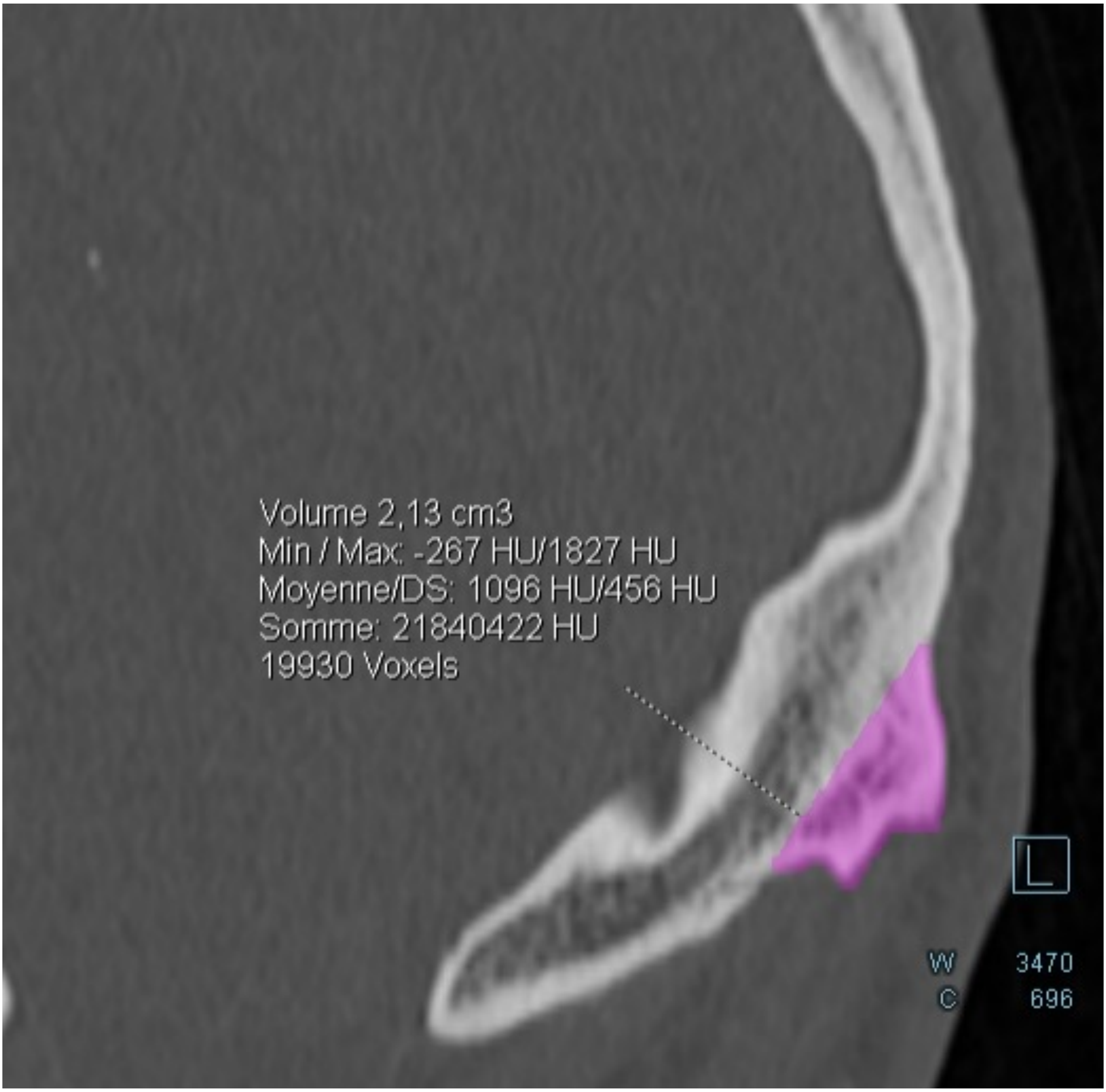
Volume 2,13 cm3
Min / Max: -267 HU/1827 HU
Moyenne/DS: 1096 HU/456 HU
Somme: 21840422 HU
19930 Voxels
L
W 3470
C 696

11m33

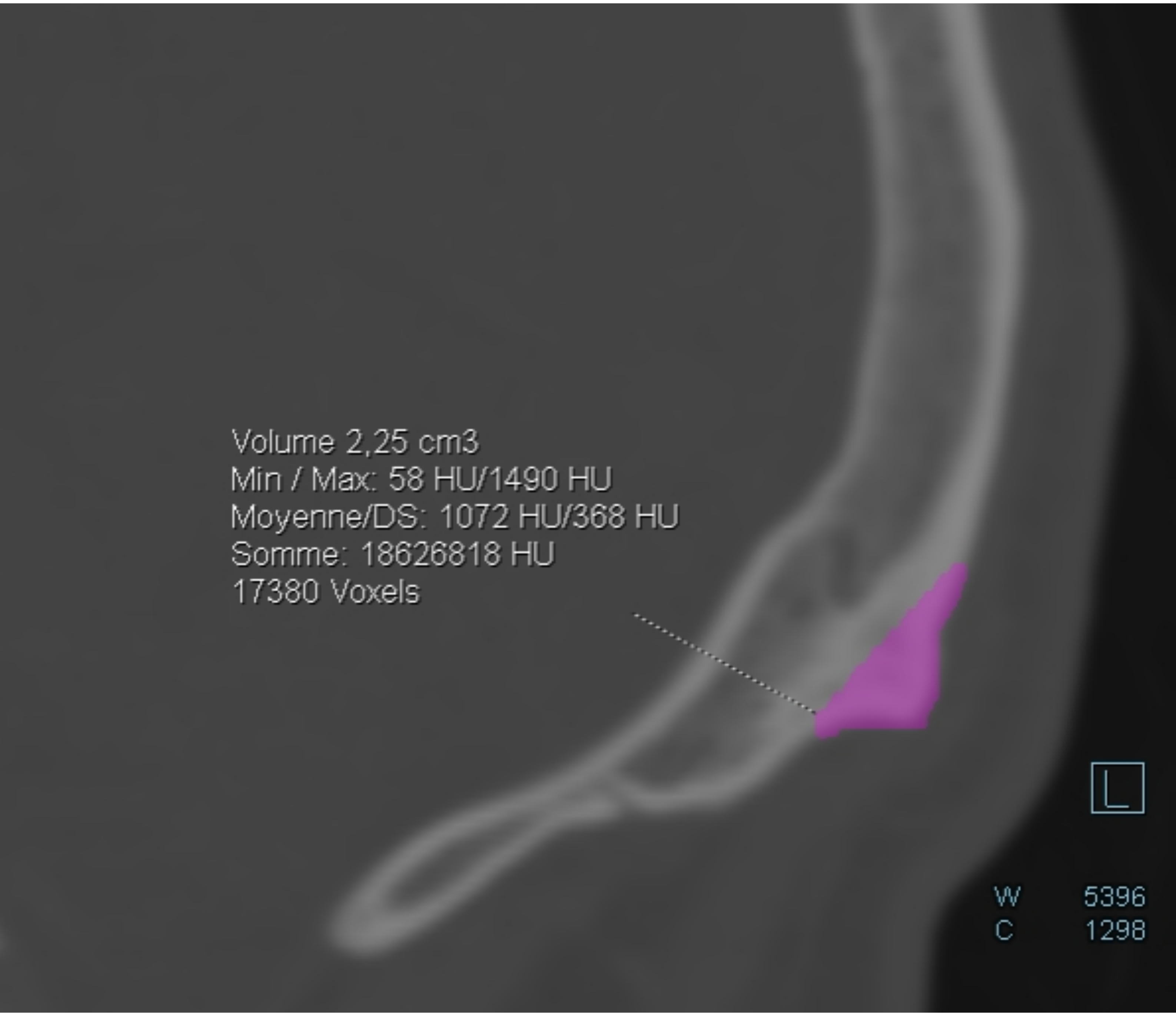
Volume 2,25 cm3
Min / Max: 58 HU/1490 HU
Moyenne/DS: 1072 HU/368 HU
Somme: 18626818 HU
17380 Voxels
L
W 5396
C 1298

11m34

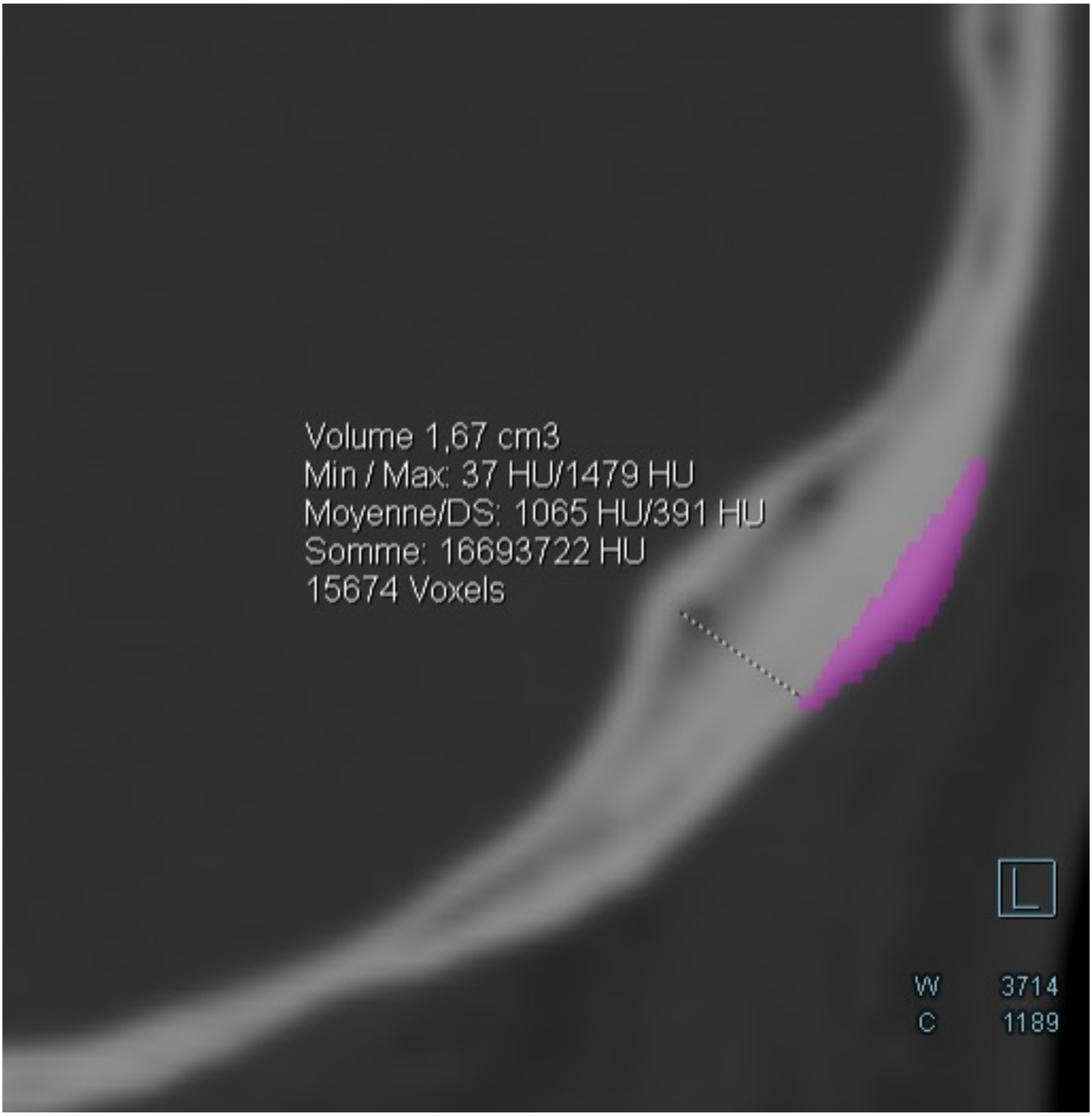
Volume 1,67 cm3
Min / Max: 37 HU/1479 HU
Moyenne/DS: 1065 HU/391 HU
Somme: 16693722 HU
15674 Voxels
L
W 3714
C 1189

11m35

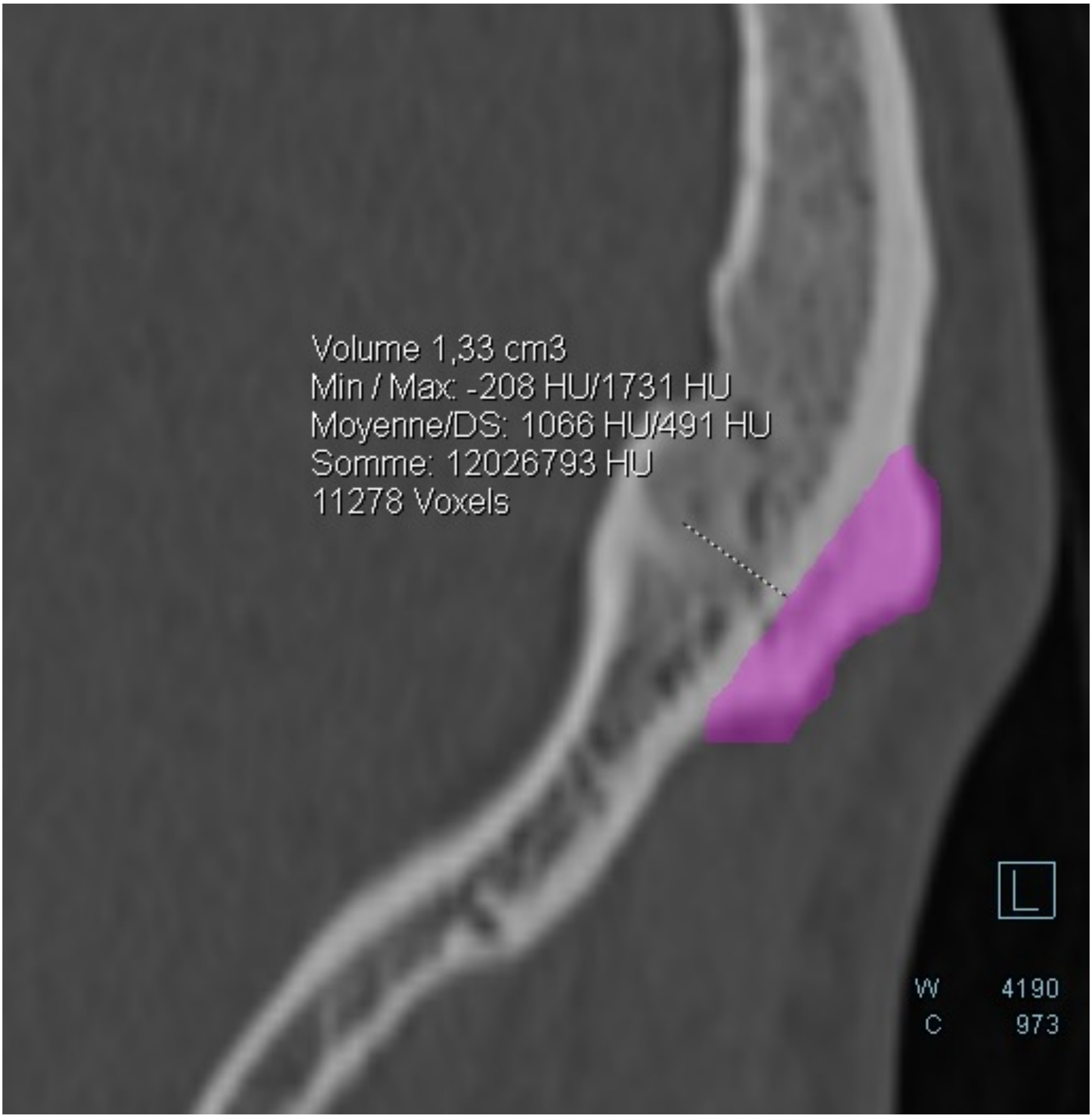

Volume 1,33 cm3
Min / Max: -208 HU/1731 HU
Moyenne/DS: 1066 HU/491 HU
Somme: 12026793 HU
11278 Voxels
L
W 4190
C 973

11m36

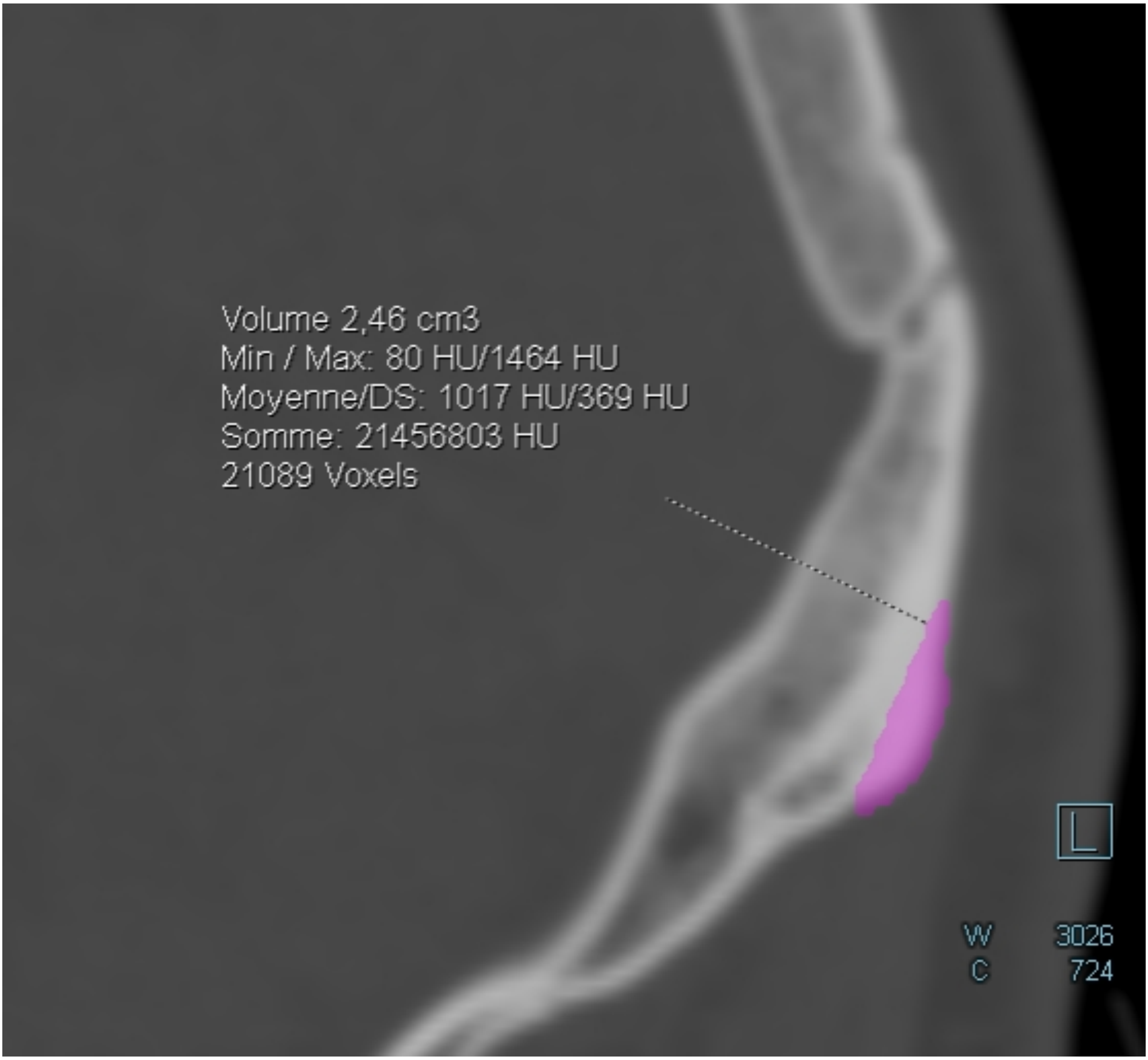
Volume 2,46 cm3
Min / Max: 80 HU/1464 HU
Moyenne/DS: 1017 HU/369 HU
Somme: 21456803 HU
21089 Voxels
L
W 3026
C 724

11m37

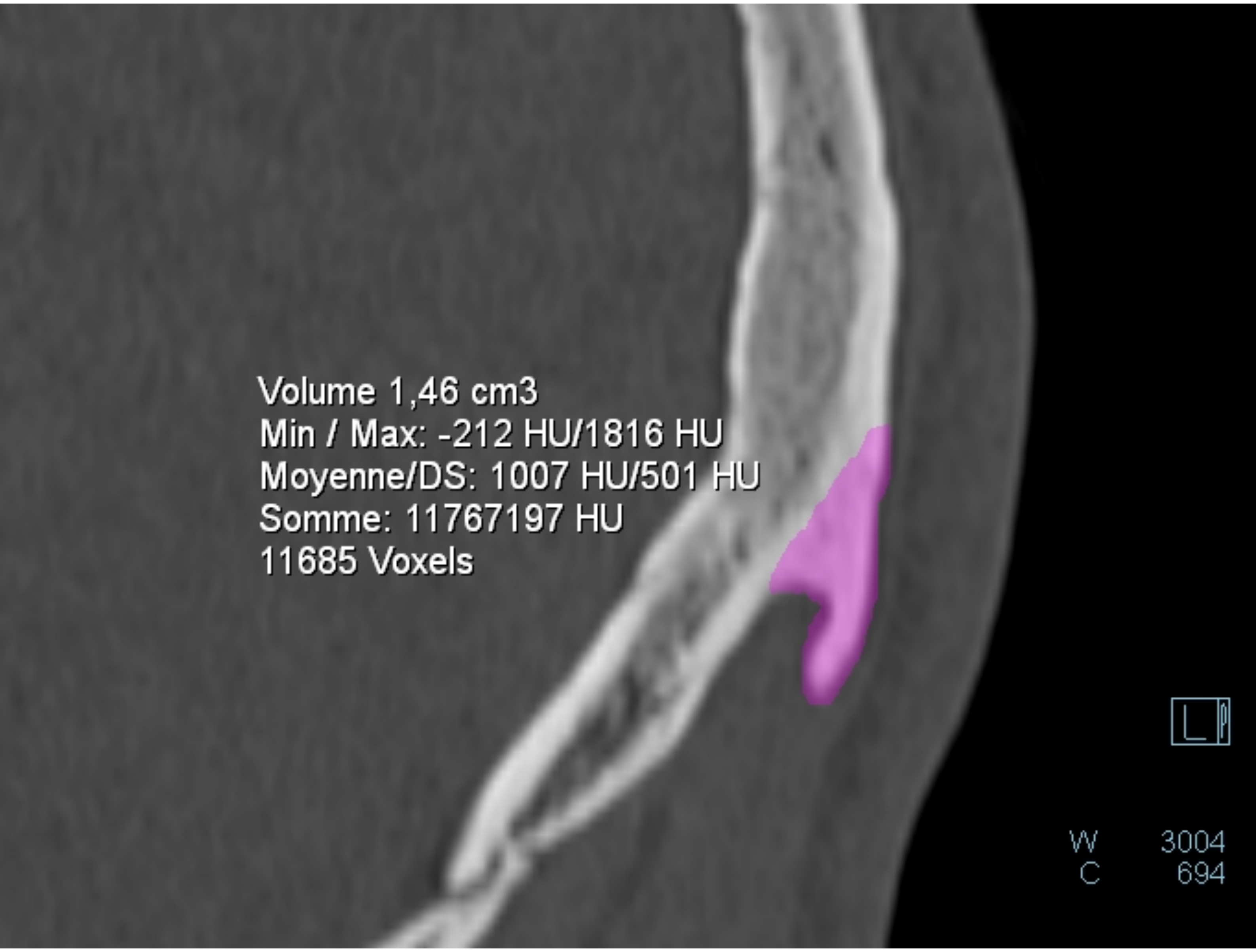
Volume 1,46 cm3
Min / Max: -212 HU/1816 HU
Moyenne/DS: 1007 HU/501 HU
Somme: 11767197 HU
11685 Voxels
W 3004
C 694

11m38

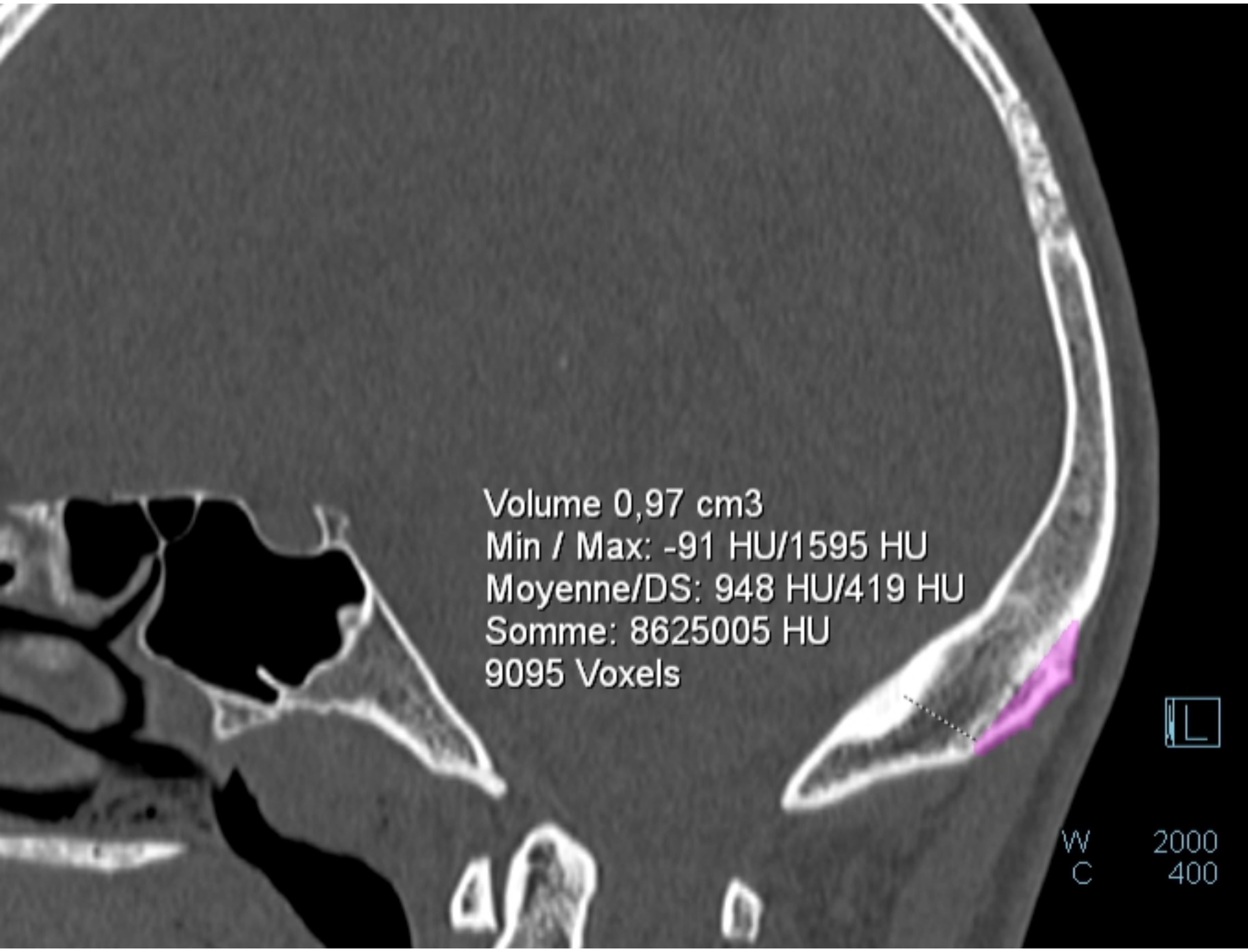
Volume 0,97 cm3
Min / Max: -91 HU/1595 HU
Moyenne/DS: 948 HU/419 HU
Somme: 8625005 HU
9095 Voxels
L
W 2000
C 400

11m39

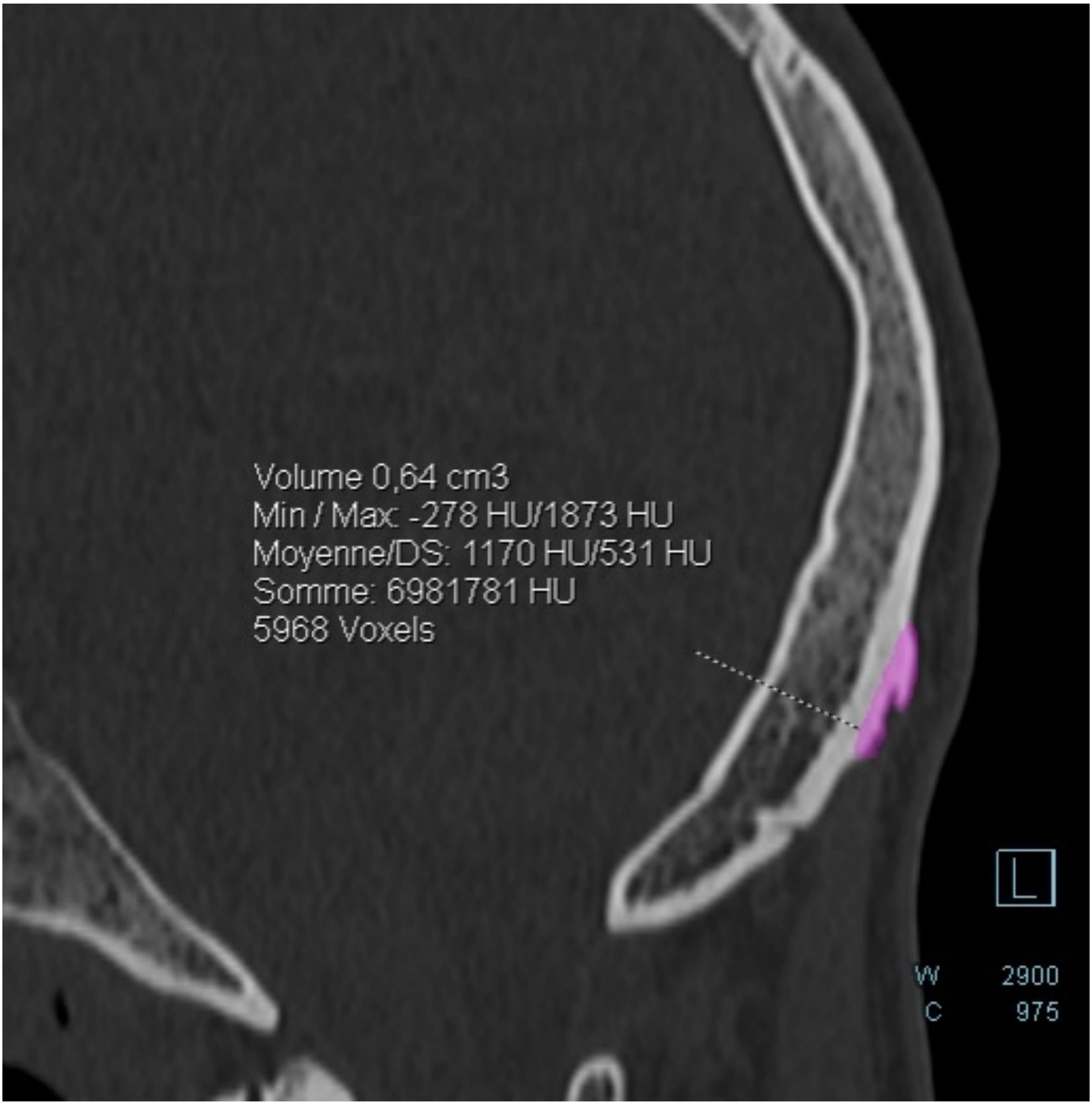
Volume 0,64 cm3
Min / Max: -278 HU/1873 HU
Moyenne/DS: 1170 HU/531 HU
Somme: 6981781 HU
5968 Voxels
L
W 2900
C 975

11m40b

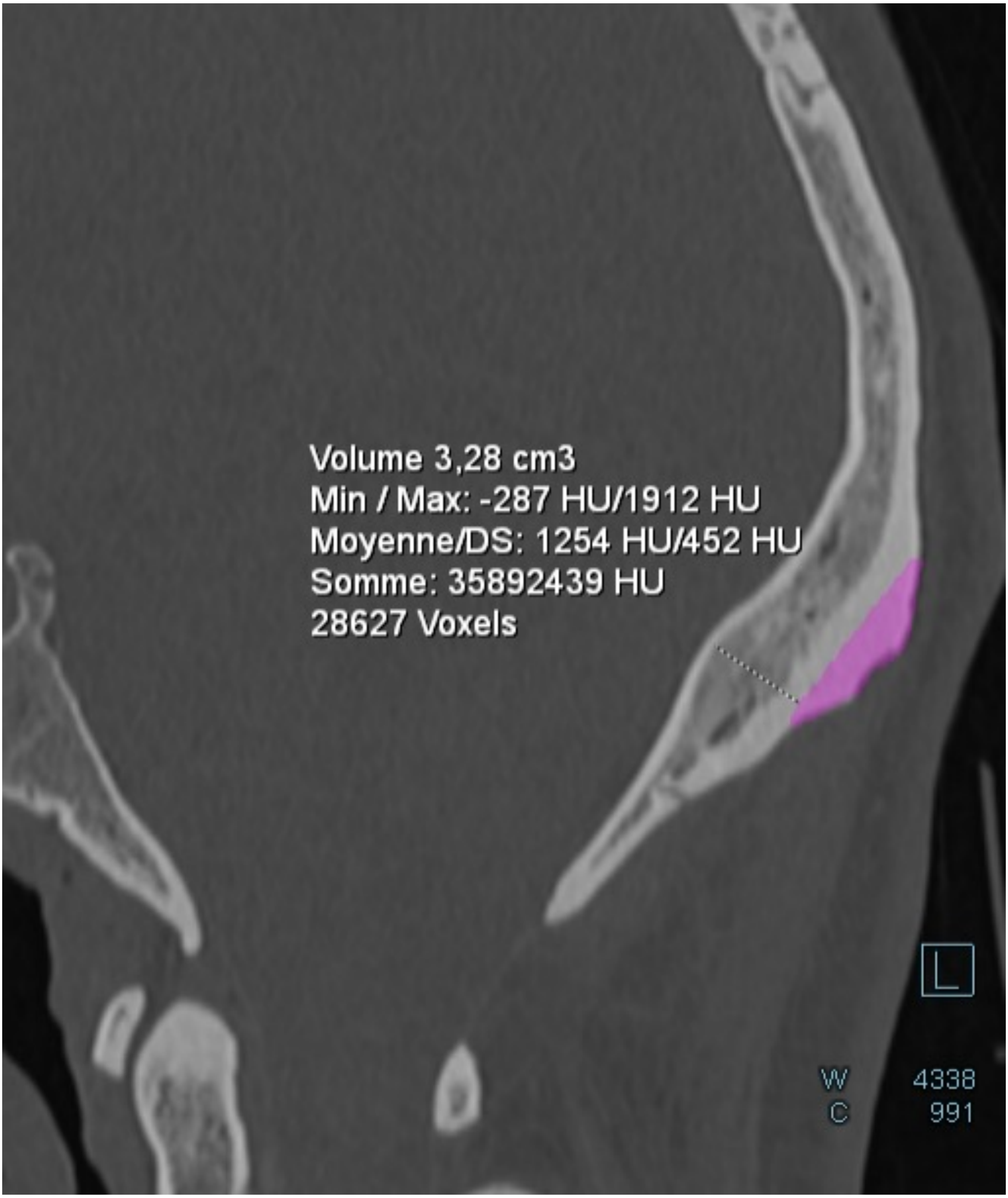
Volume 3,28 cm3
Min / Max: -287 HU/1912 HU
Moyenne/DS: 1254 HU/452 HU
Somme: 35892439 HU
28627 Voxels
L
W 4338
C 991

11m41

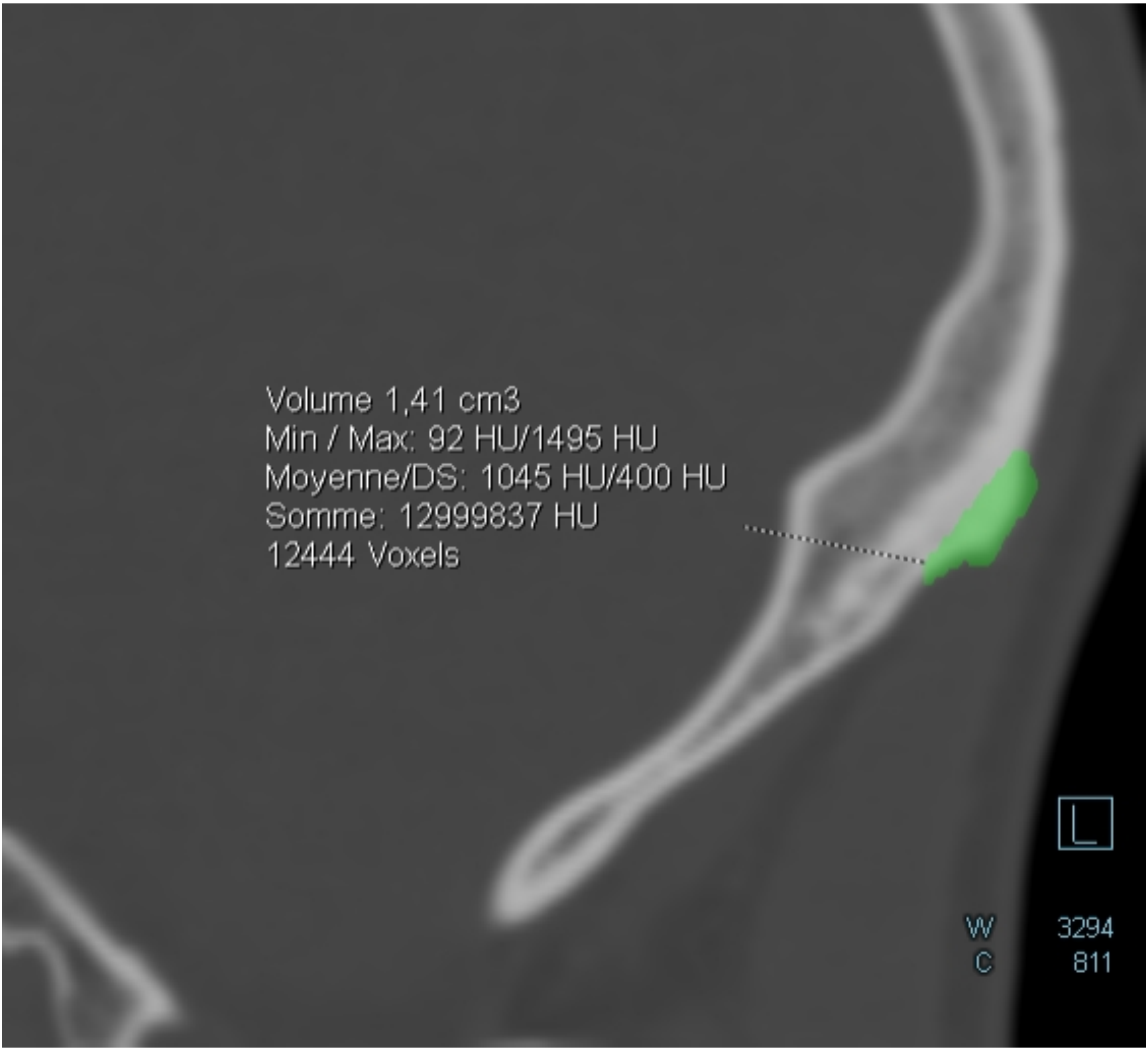
Volume 1,41 cm3
Min / Max: 92 HU/1495 HU
Moyenne/DS: 1045 HU/400 HU
Somme: 12999837 HU
12444 Voxels
L
W 3294
C 811

11m42

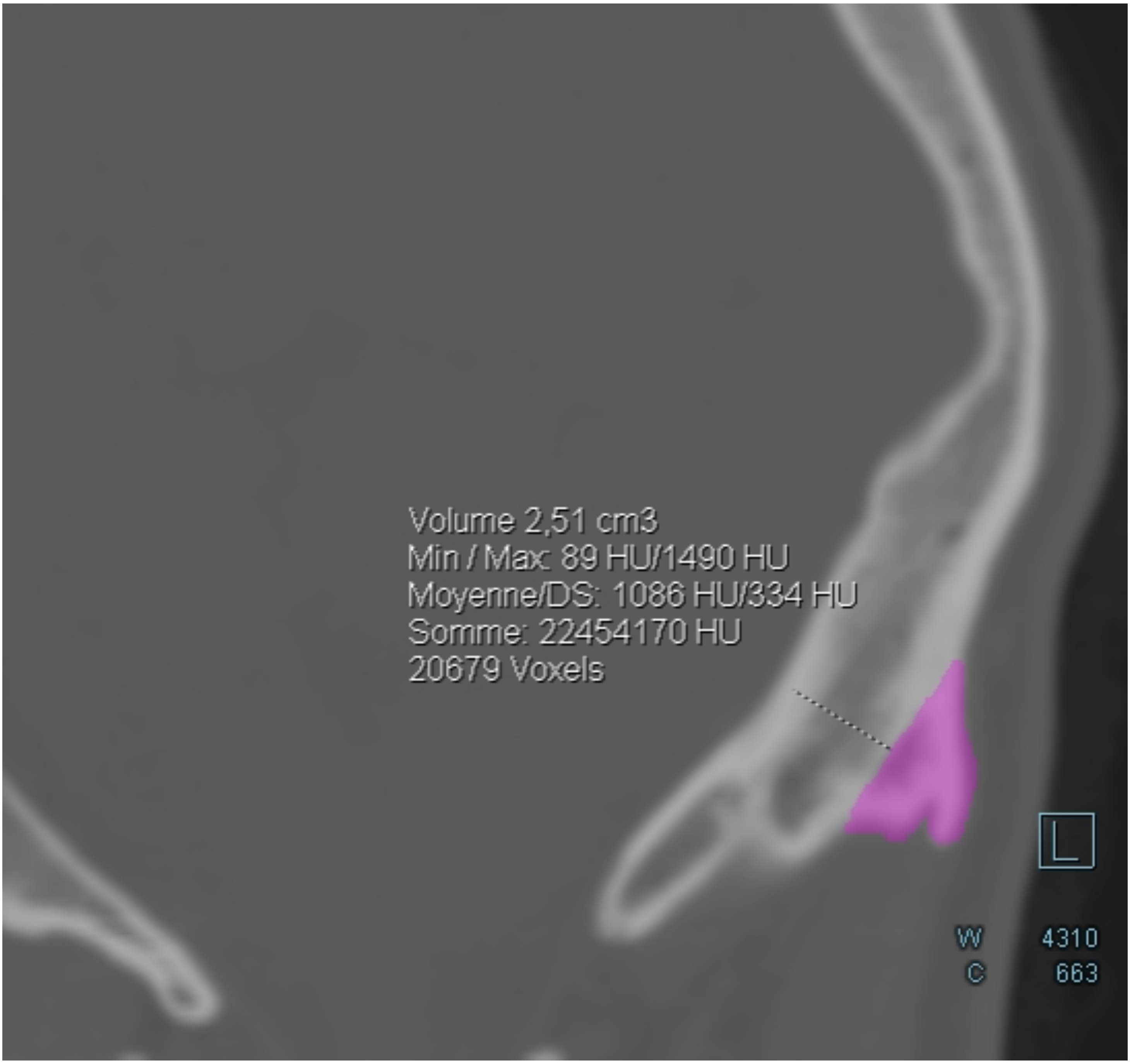
Volume 2,51 cm3
Min / Max: 89 HU/1490 HU
Moyenne/DS: 1086 HU/334 HU
Somme: 22454170 HU
20679 Voxels
L
W 4310
C 663

11m43

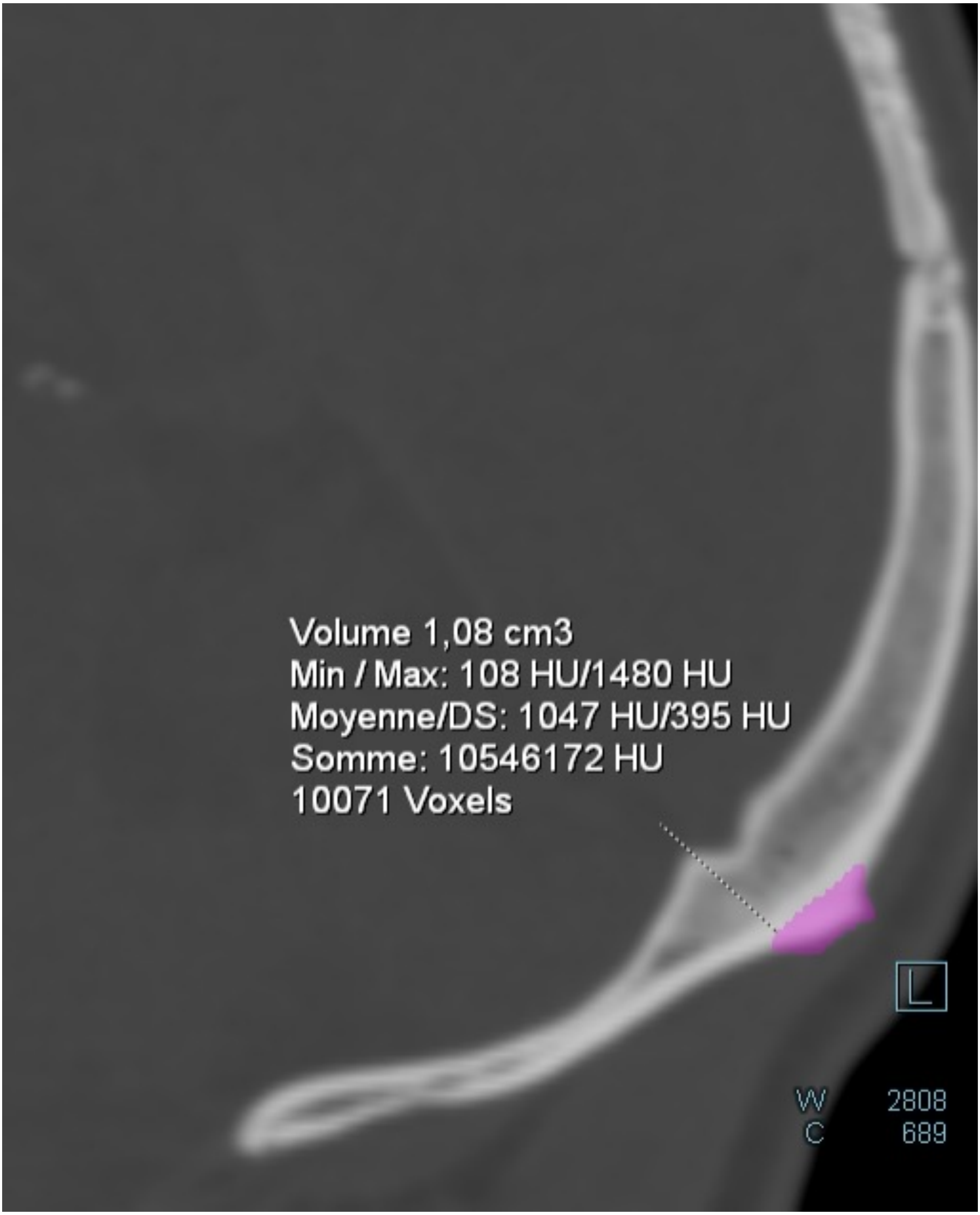
Volume 1,08 cm3
Min / Max: 108 HU/1480 HU
Moyenne/DS: 1047 HU/395 HU
Somme: 10546172 HU
10071 Voxels
L
W 2808
C 689

11m44

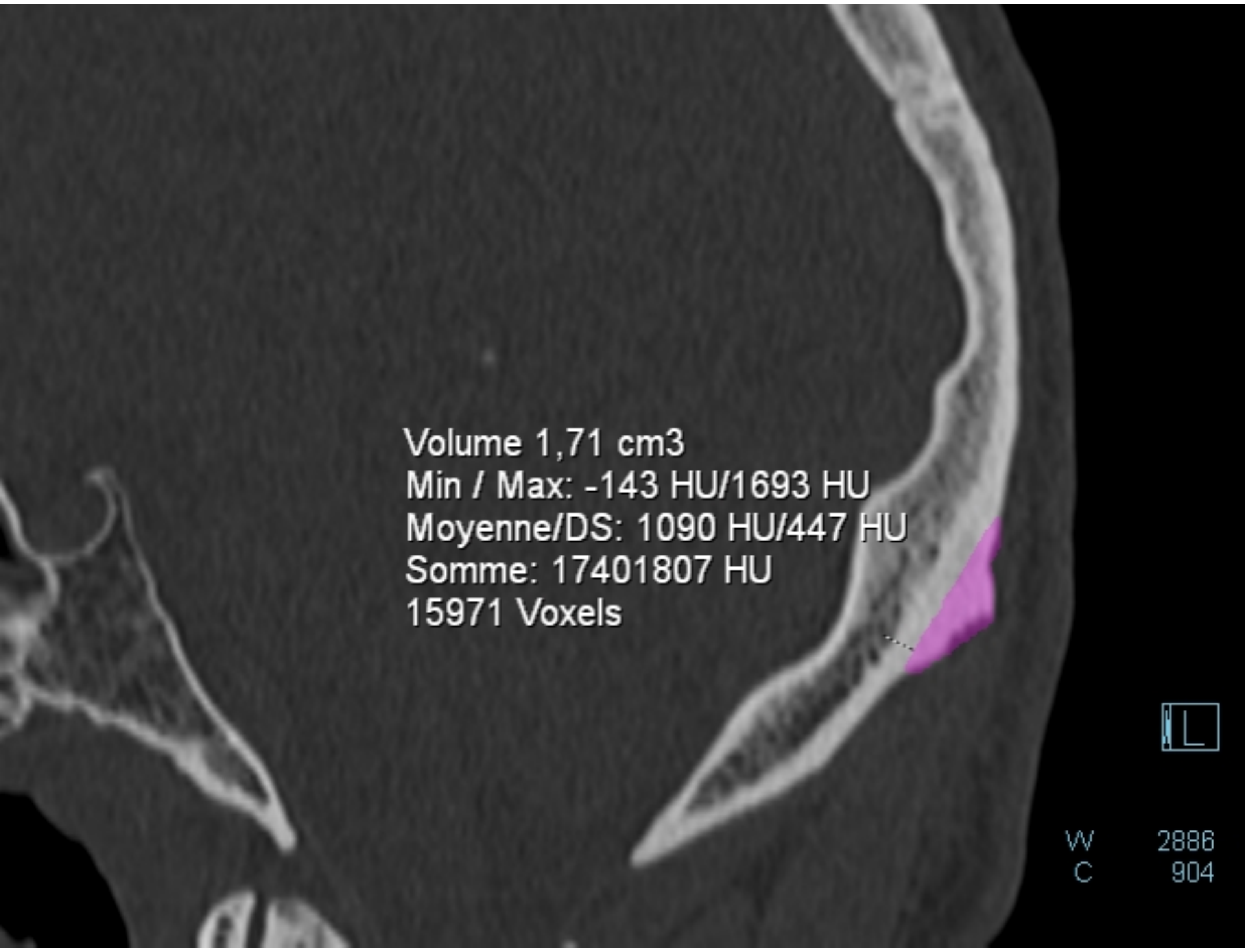
Volume 1,71 cm3
Min / Max: -143 HU/1693 HU
Moyenne/DS: 1090 HU/447 HU
Somme: 17401807 HU
15971 Voxels
L
W 2886
C 904

11m45

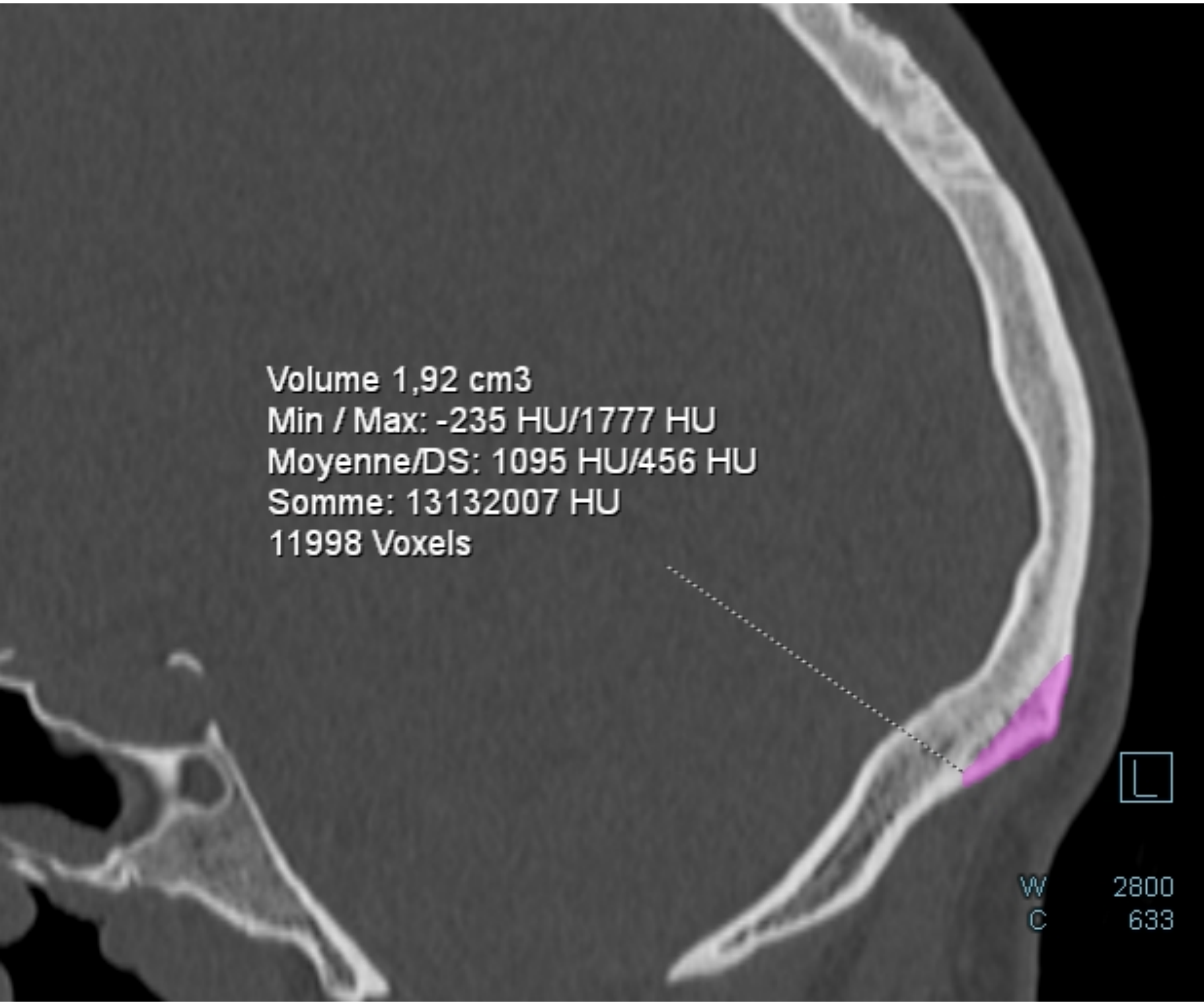
Volume 1,92 cm3
Min / Max: -235 HU/1777 HU
Moyenne/DS: 1095 HU/456 HU
Somme: 13132007 HU
11998 Voxels
L
W 2800
C 633

11m46

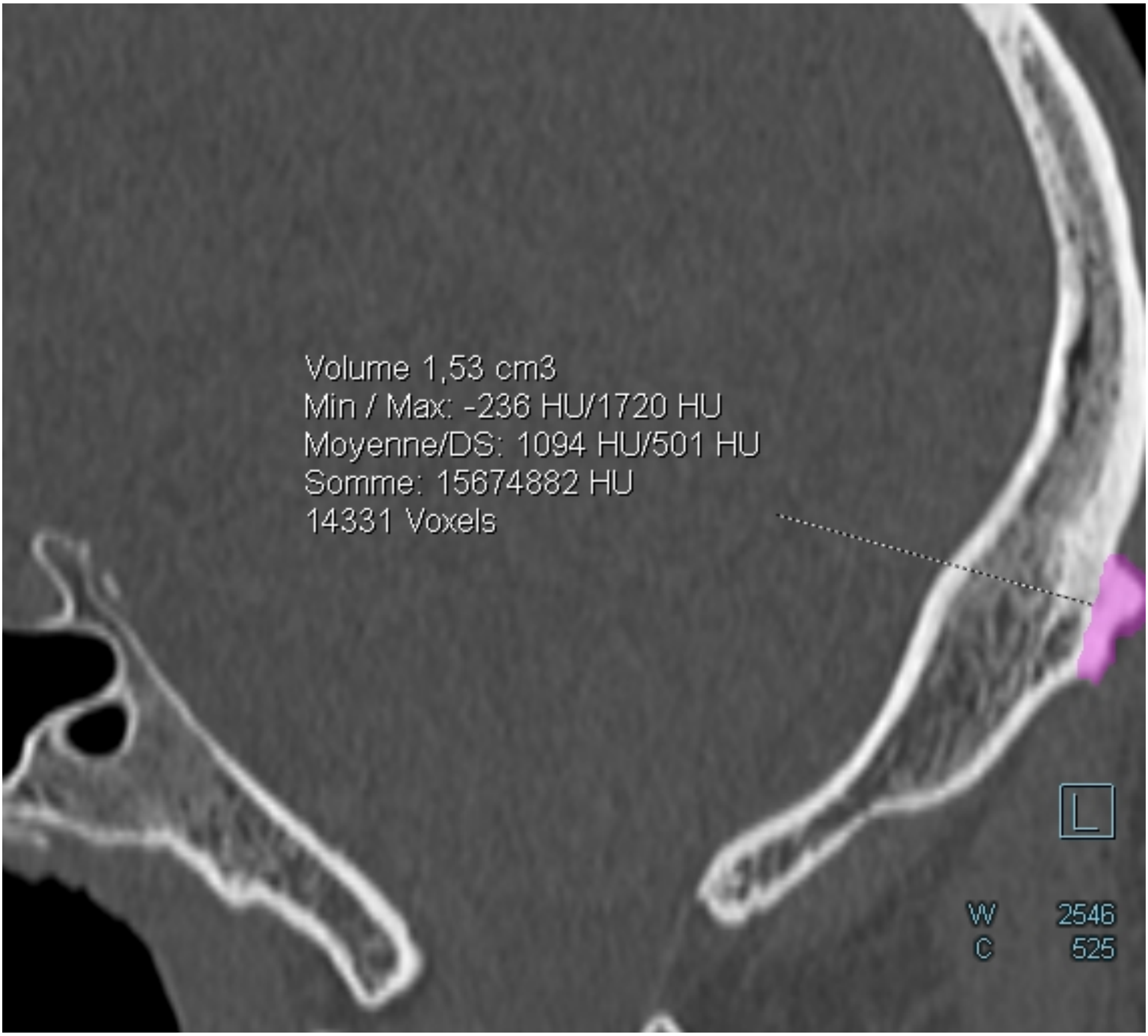
Volume 1,53 cm3
Min / Max: -236 HU/1720 HU
Moyenne/DS: 1094 HU/501 HU
Somme: 15674882 HU
14331 Voxels
L
W 2546
C 525

11m47

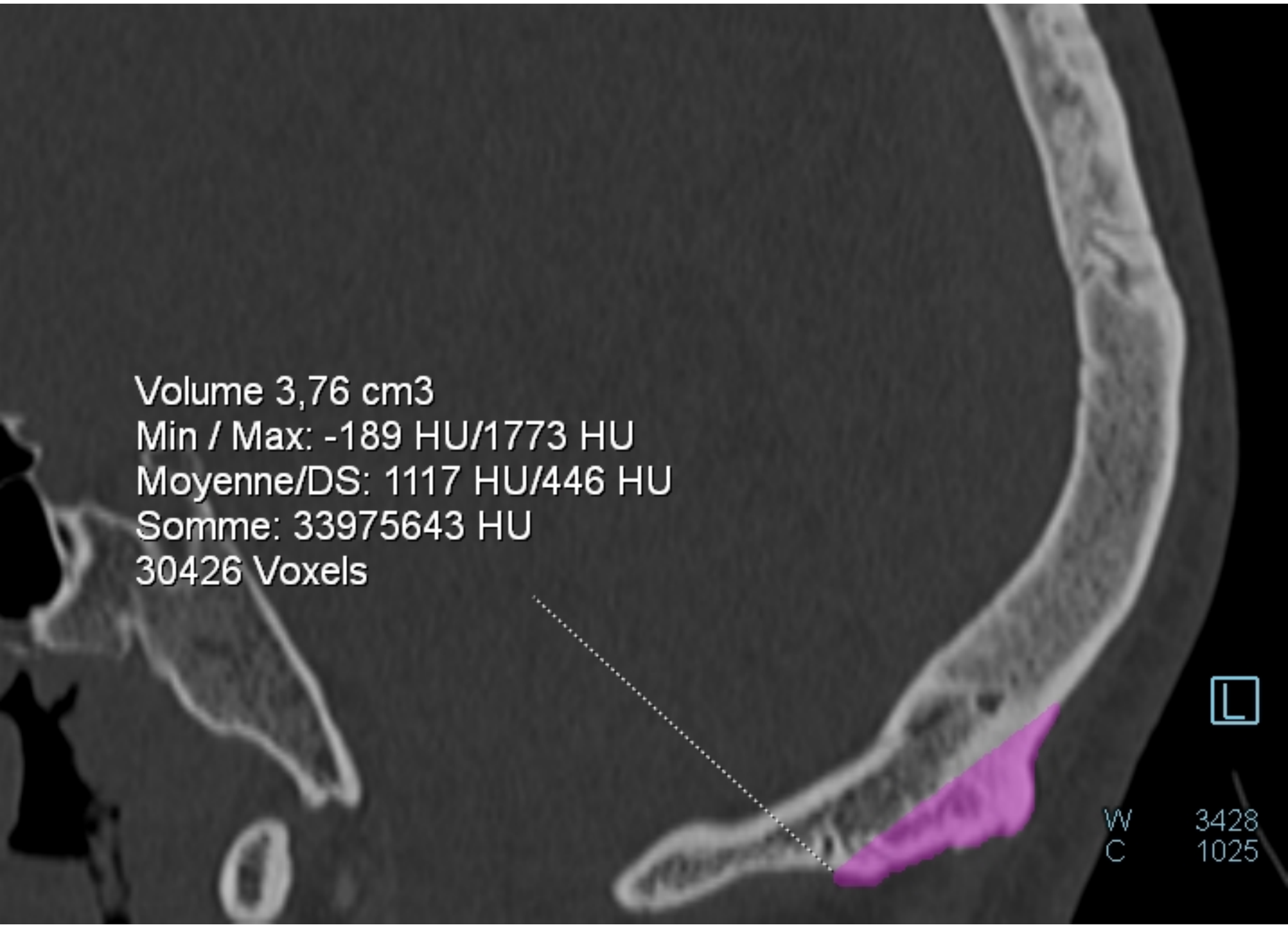

Volume 3,76 cm3
Min / Max: -189 HU/1773 HU
Moyenne/DS: 1117 HU/446 HU
Somme: 33975643 HU
30426 Voxels
L
W 3428
C 1025

11m48

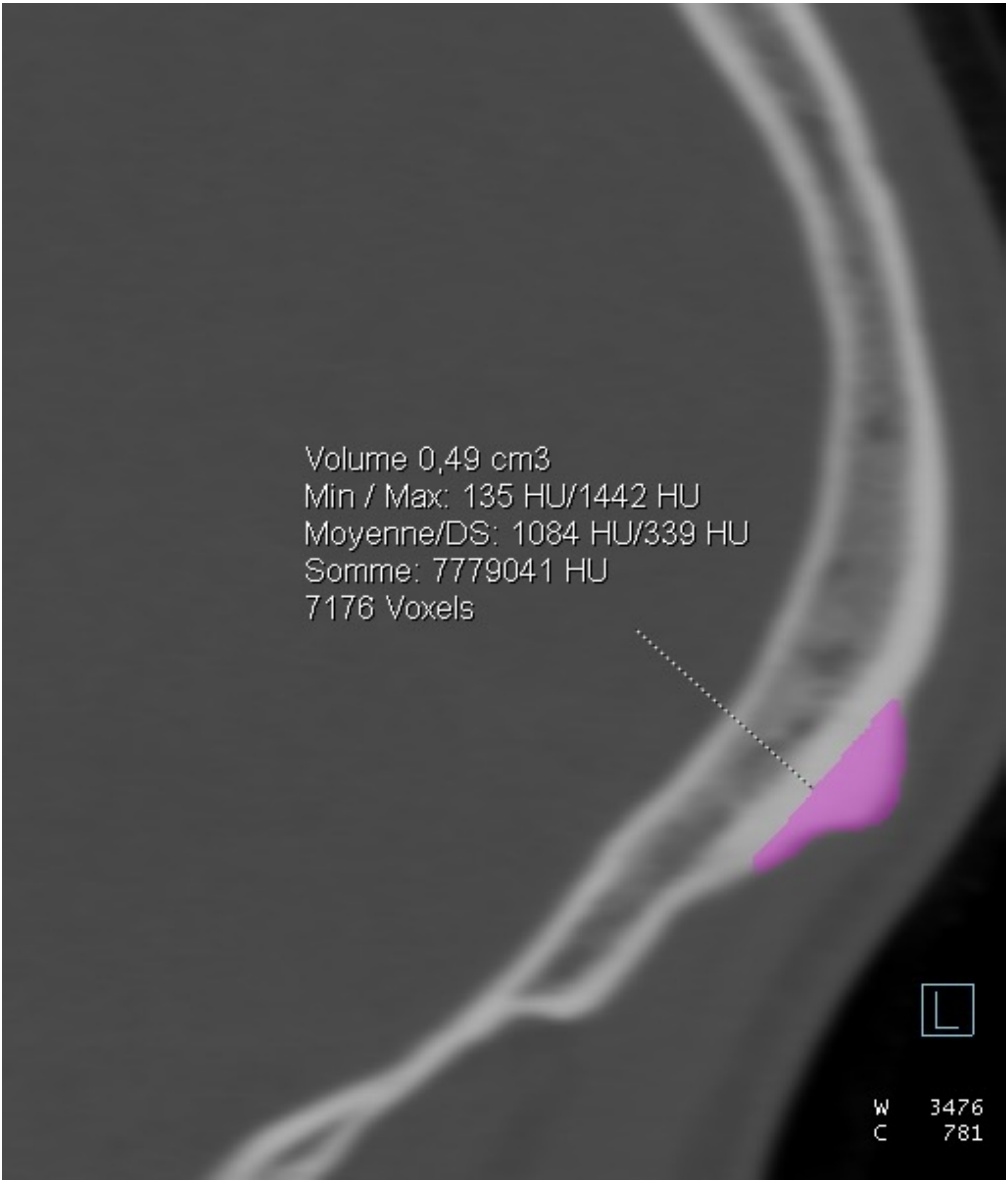
Volume 0,49 cm3
Min / Max: 135 HU/1442 HU
Moyenne/DS: 1084 HU/339 HU
Somme: 7779041 HU
7176 Voxels
L
W 3476
C 781

11m49

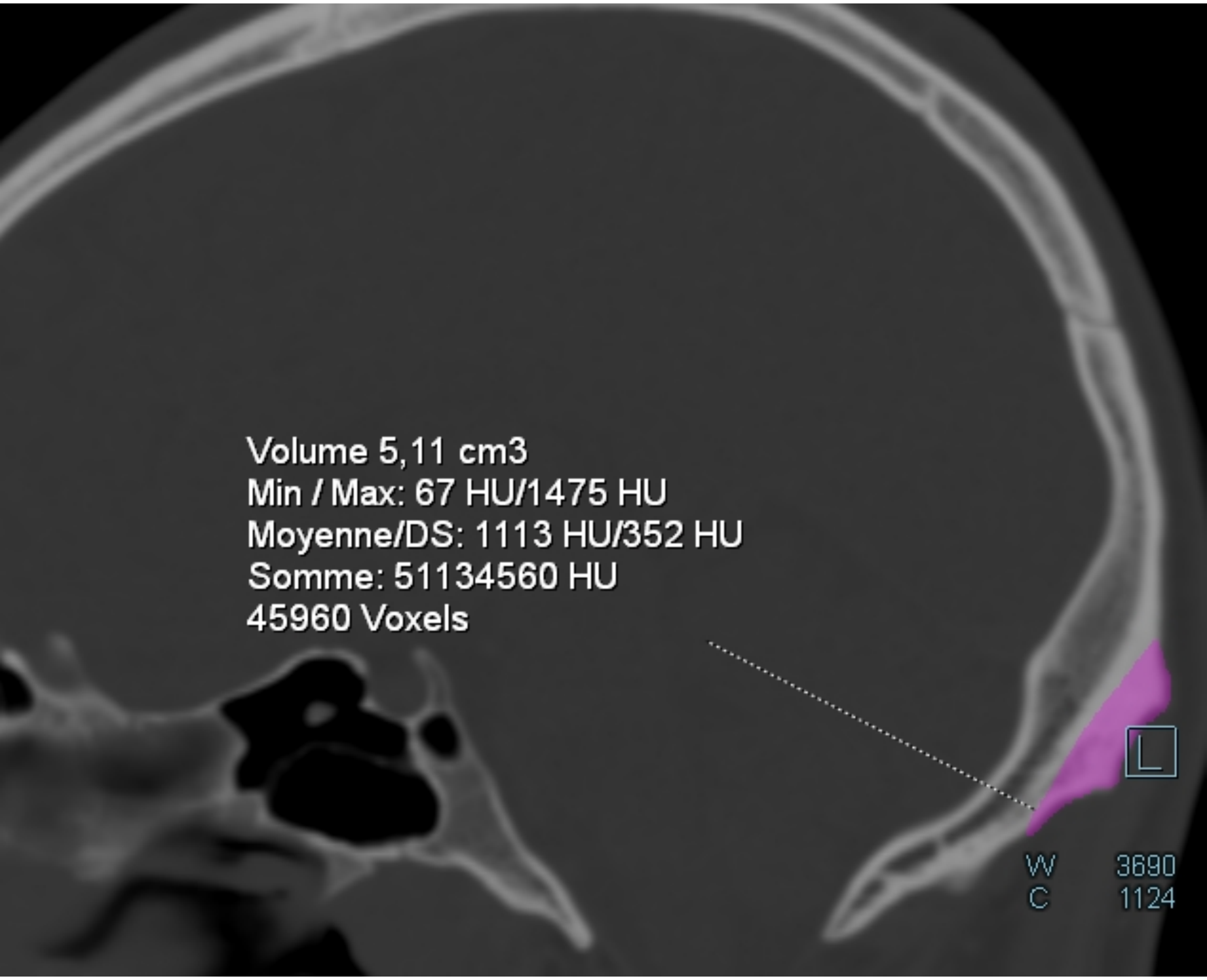
Volume 5,11 cm3
Min / Max: 67 HU/1475 HU
Moyenne/DS: 1113 HU/352 HU
Somme: 51134560 HU
45960 Voxels
L
W 3690
C 1124

11m50

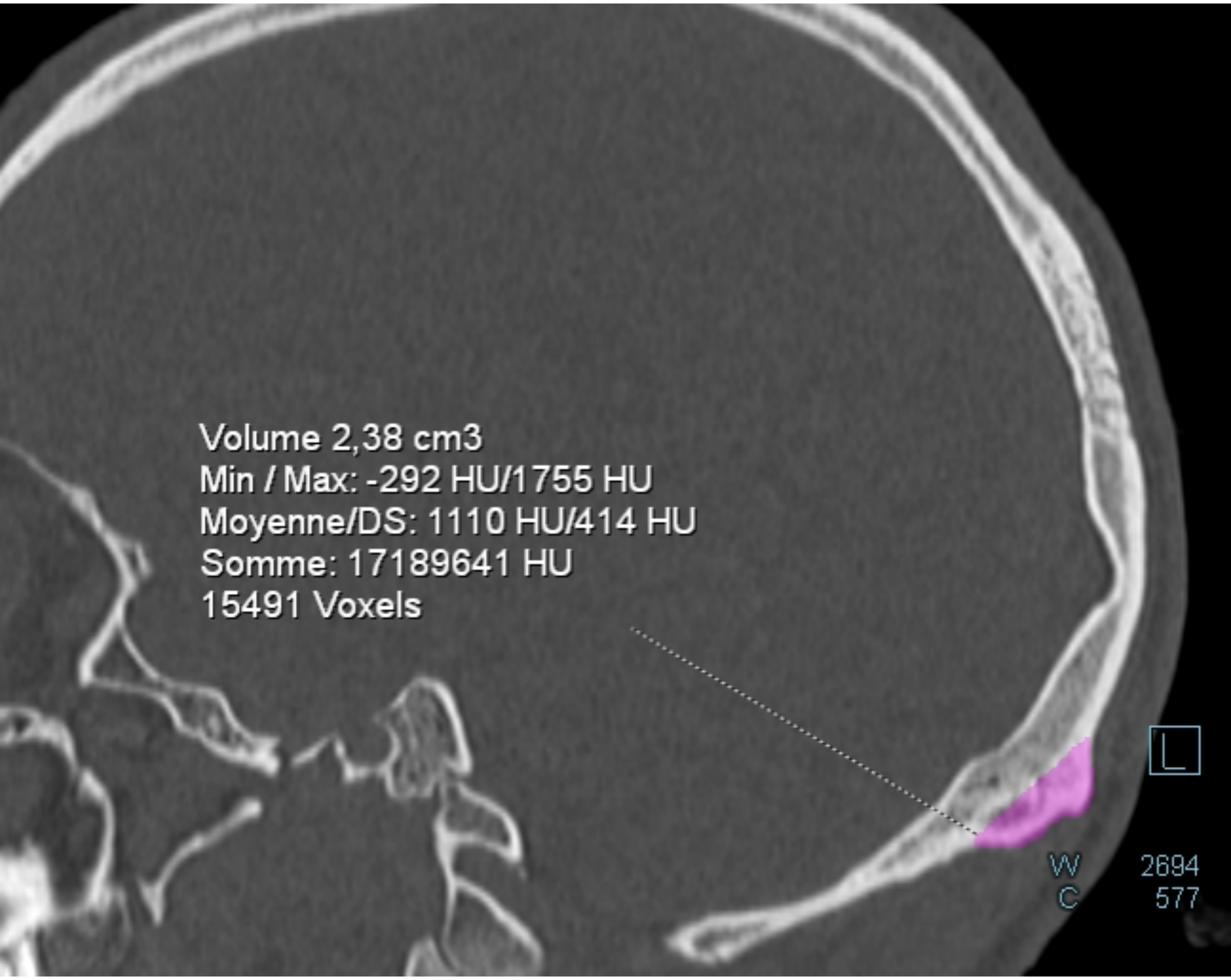

Volume 2,38 cm3
Min / Max: -292 HU/1755 HU
Moyenne/DS: 1110 HU/414 HU
Somme: 17189641 HU
15491 Voxels
L
W 2694
C 577

11m51

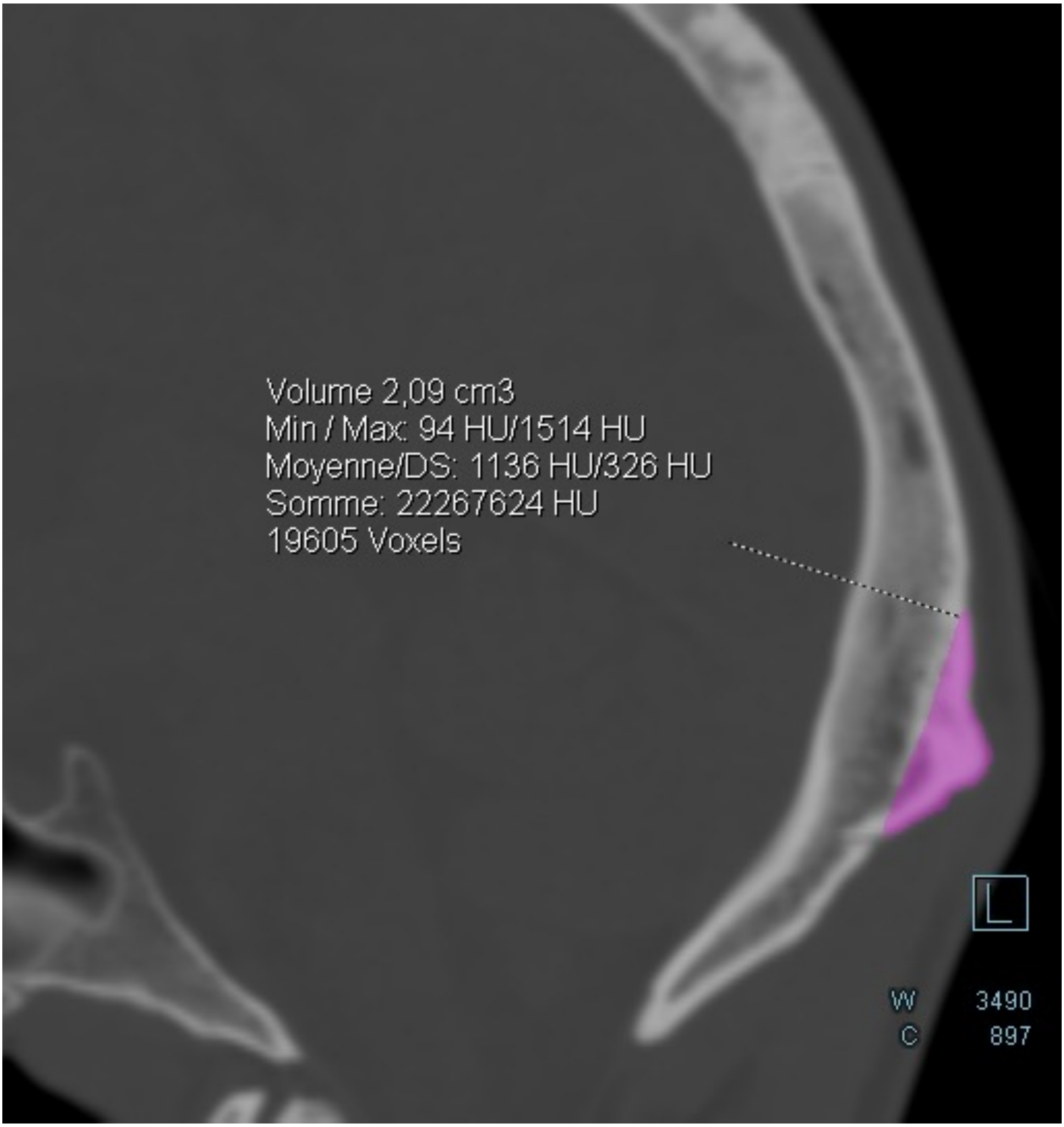
Volume 2,09 cm3
Min / Max: 94 HU/1514 HU
Moyenne/DS: 1136 HU/326 HU
Somme: 22267624 HU
19605 Voxels
L
W 3490
C 897

11m52

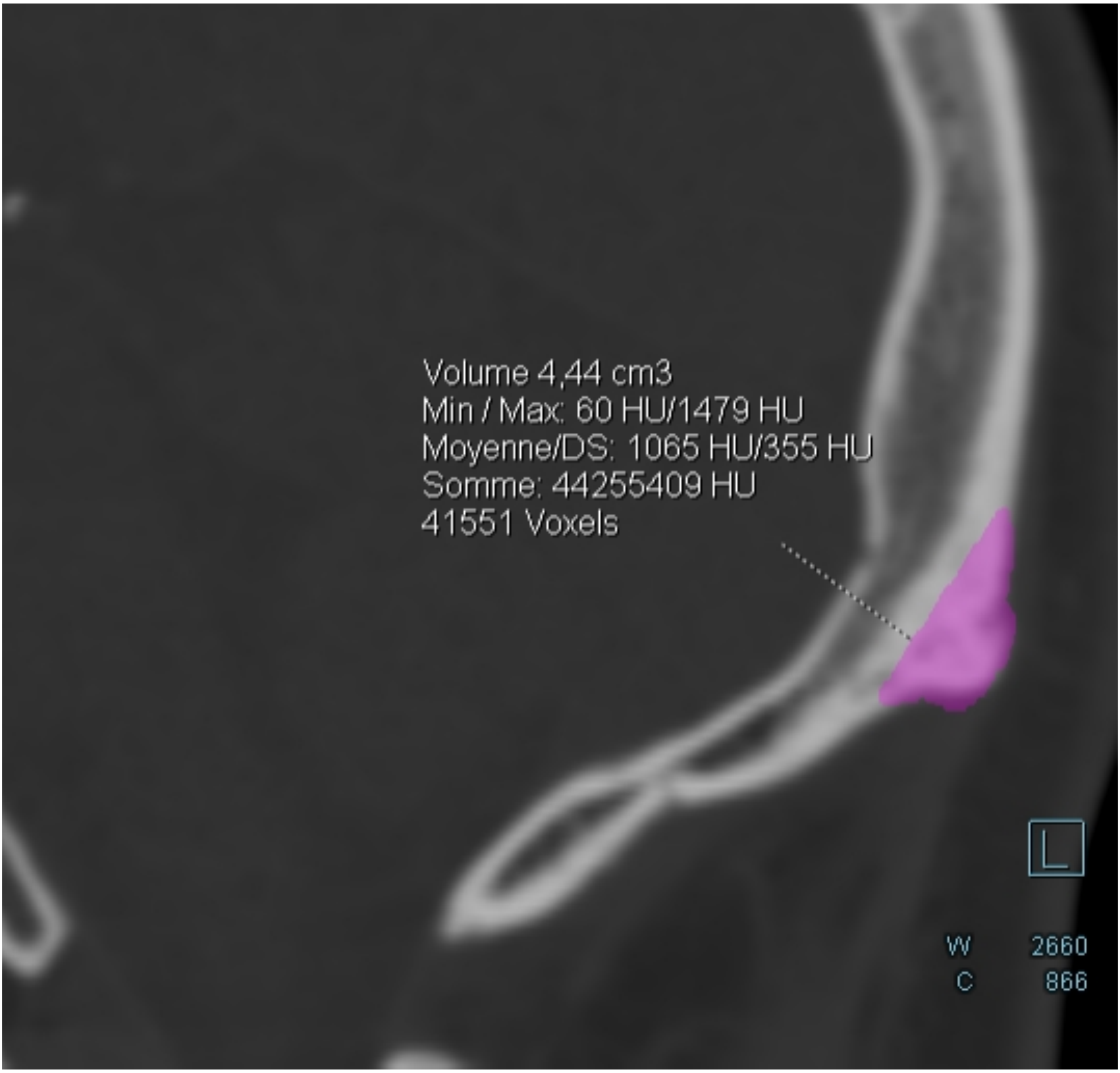
Volume 4,44 cm3
Min / Max: 60 HU/1479 HU
Moyenne/DS: 1065 HU/355 HU
Somme: 44255409 HU
41551 Voxels
L
W 2660
C 866

11m53

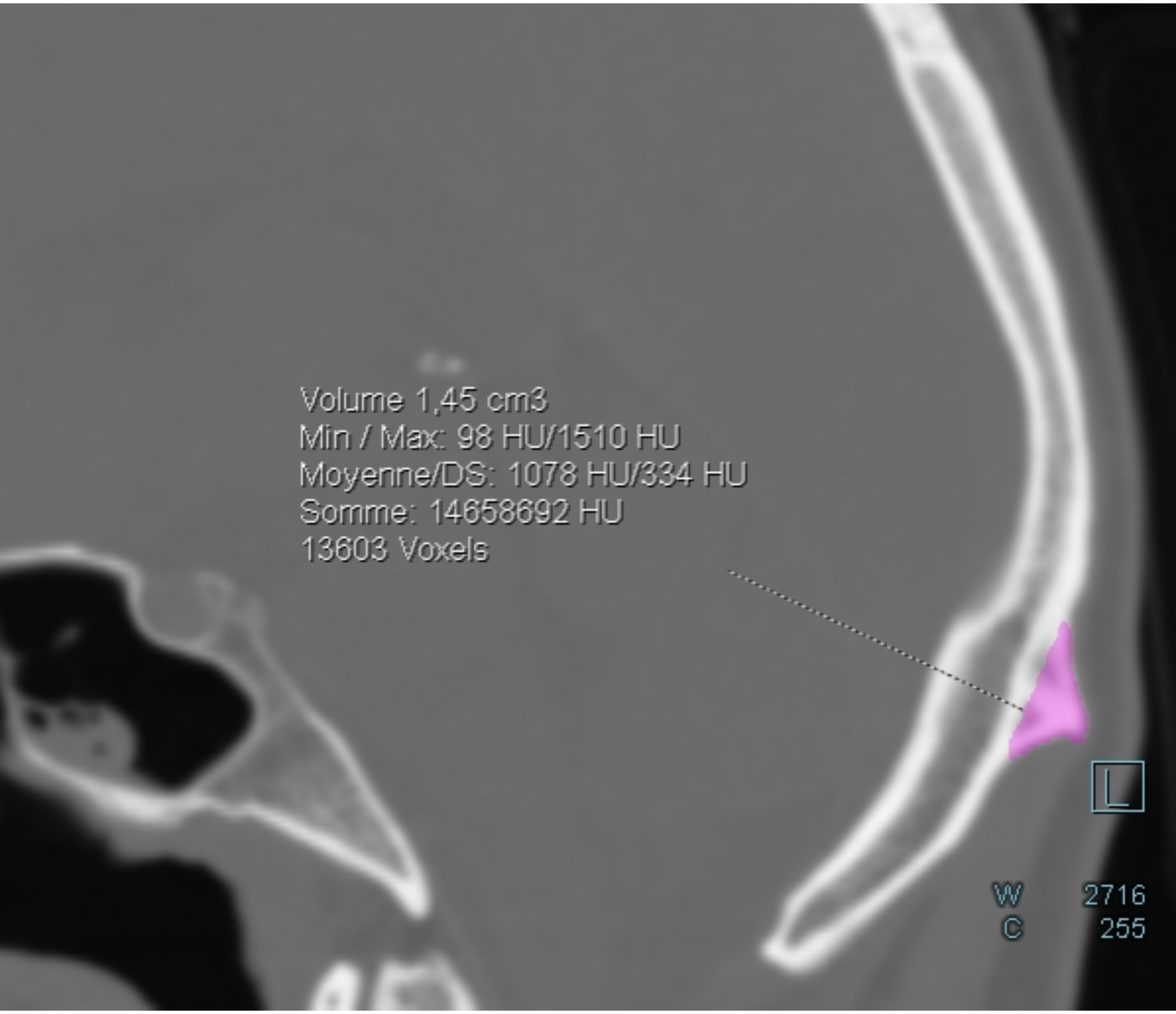
Volume 1,45 cm3
Min / Max: 98 HU/1510 HU
Moyenne/DS: 1078 HU/334 HU
Somme: 14658692 HU
13603 Voxels
L
W 2716
C 255

11m54

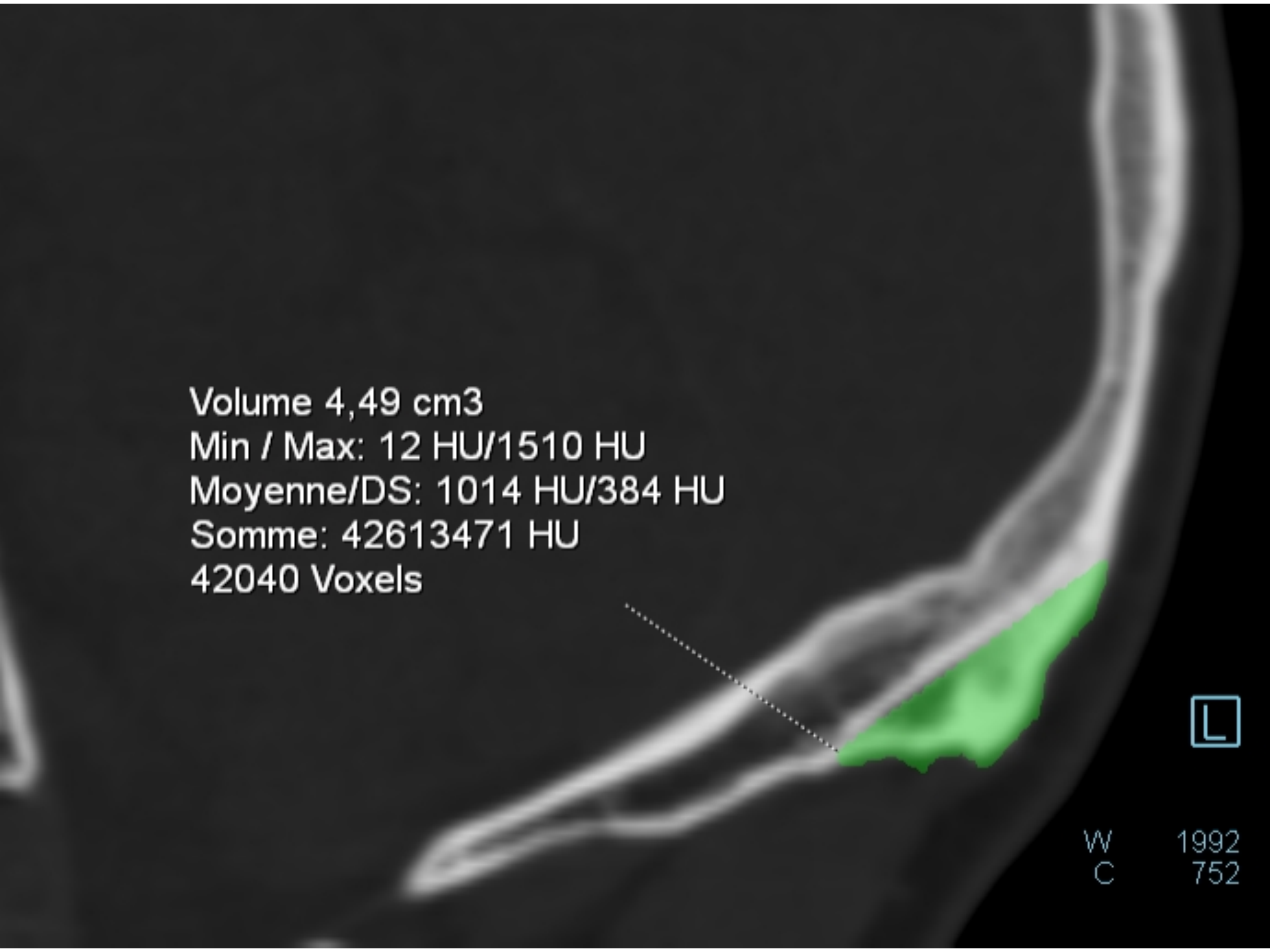
Volume 4,49 cm3
Min / Max: 12 HU/1510 HU
Moyenne/DS: 1014 HU/384 HU
Somme: 42613471 HU
42040 Voxels
L
W 1992
C 752

11m55

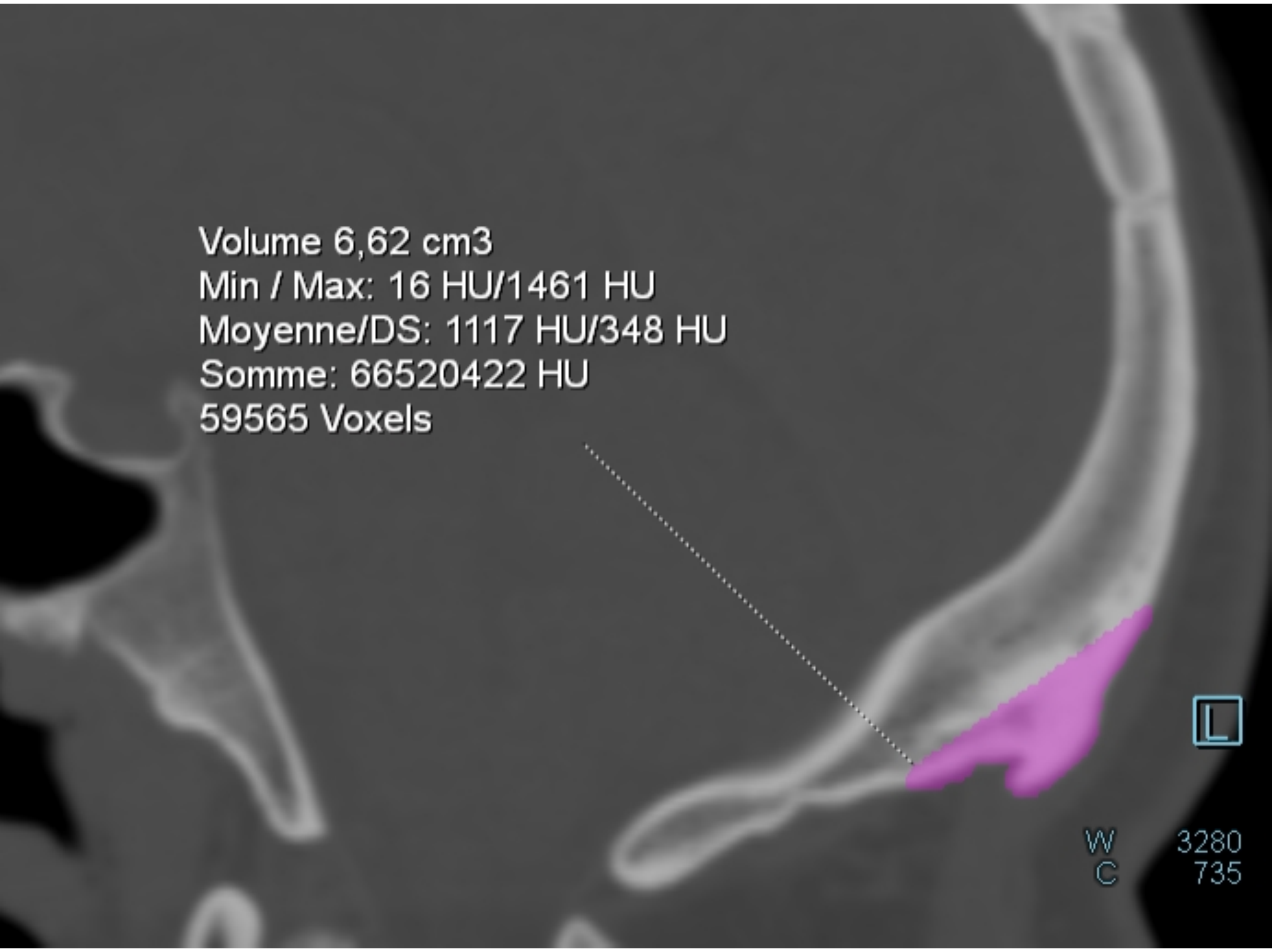
Volume 6,62 cm3
Min / Max: 16 HU/1461 HU
Moyenne/DS: 1117 HU/348 HU
Somme: 66520422 HU
59565 Voxels
L
W 3280
C 735

11m56

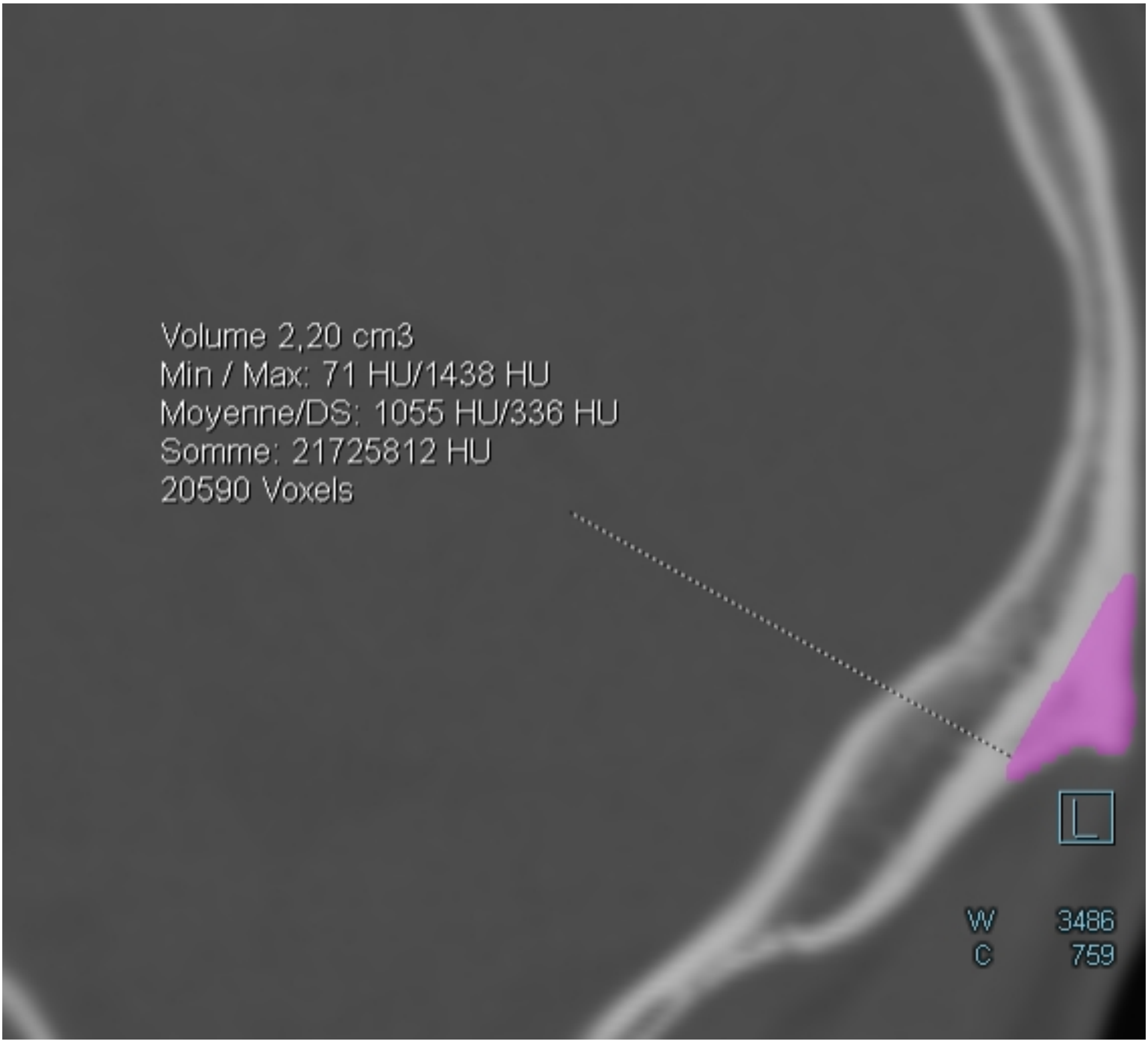
Volume 2,20 cm3
Min / Max: 71 HU/1438 HU
Moyenne/DS: 1055 HU/336 HU
Somme: 21725812 HU
20590 Voxels
L
W 3486
C 759

11m57

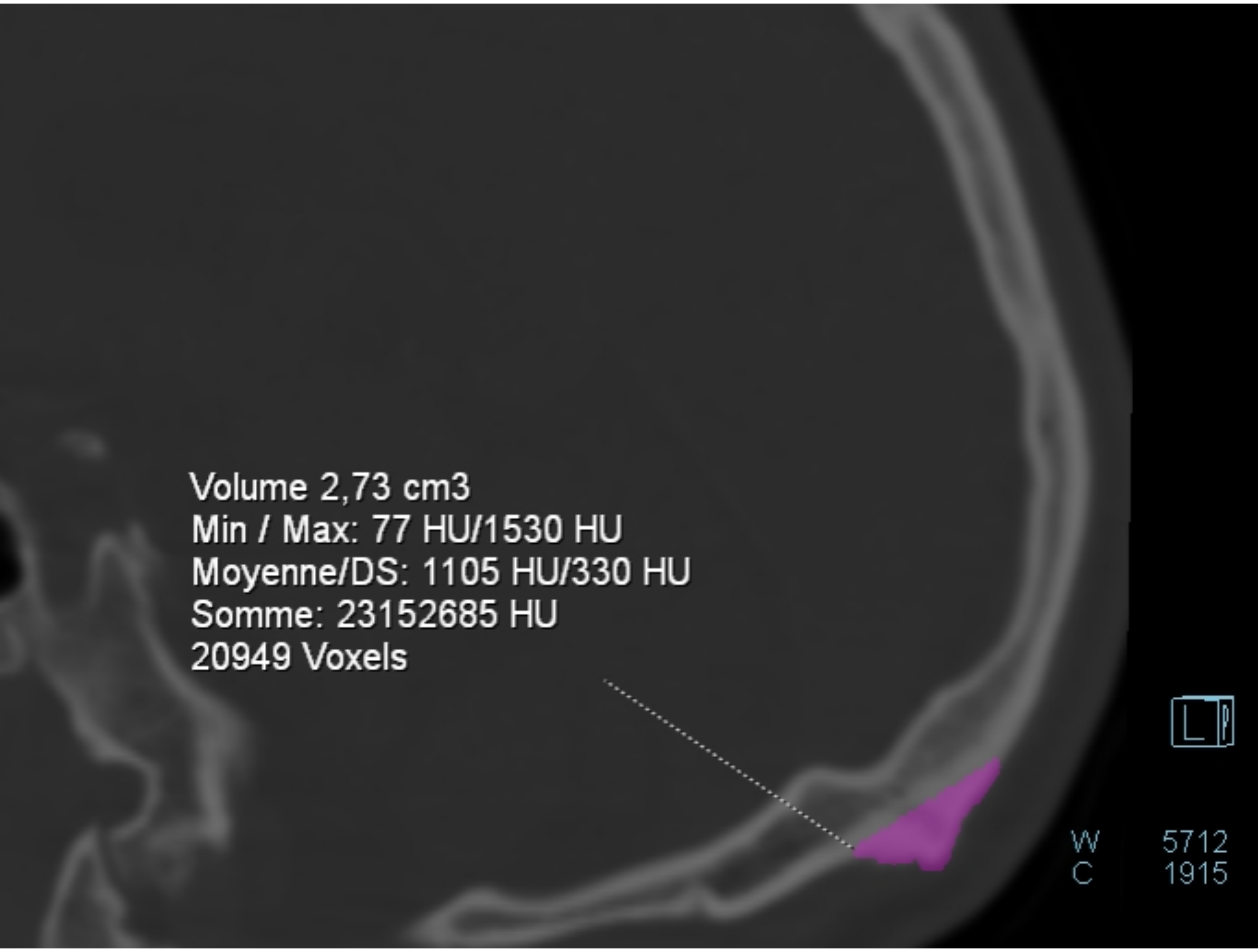
Volume 2,73 cm3
Min / Max: 77 HU/1530 HU
Moyenne/DS: 1105 HU/330 HU
Somme: 23152685 HU
20949 Voxels
L
W 5712
C 1915

11m58

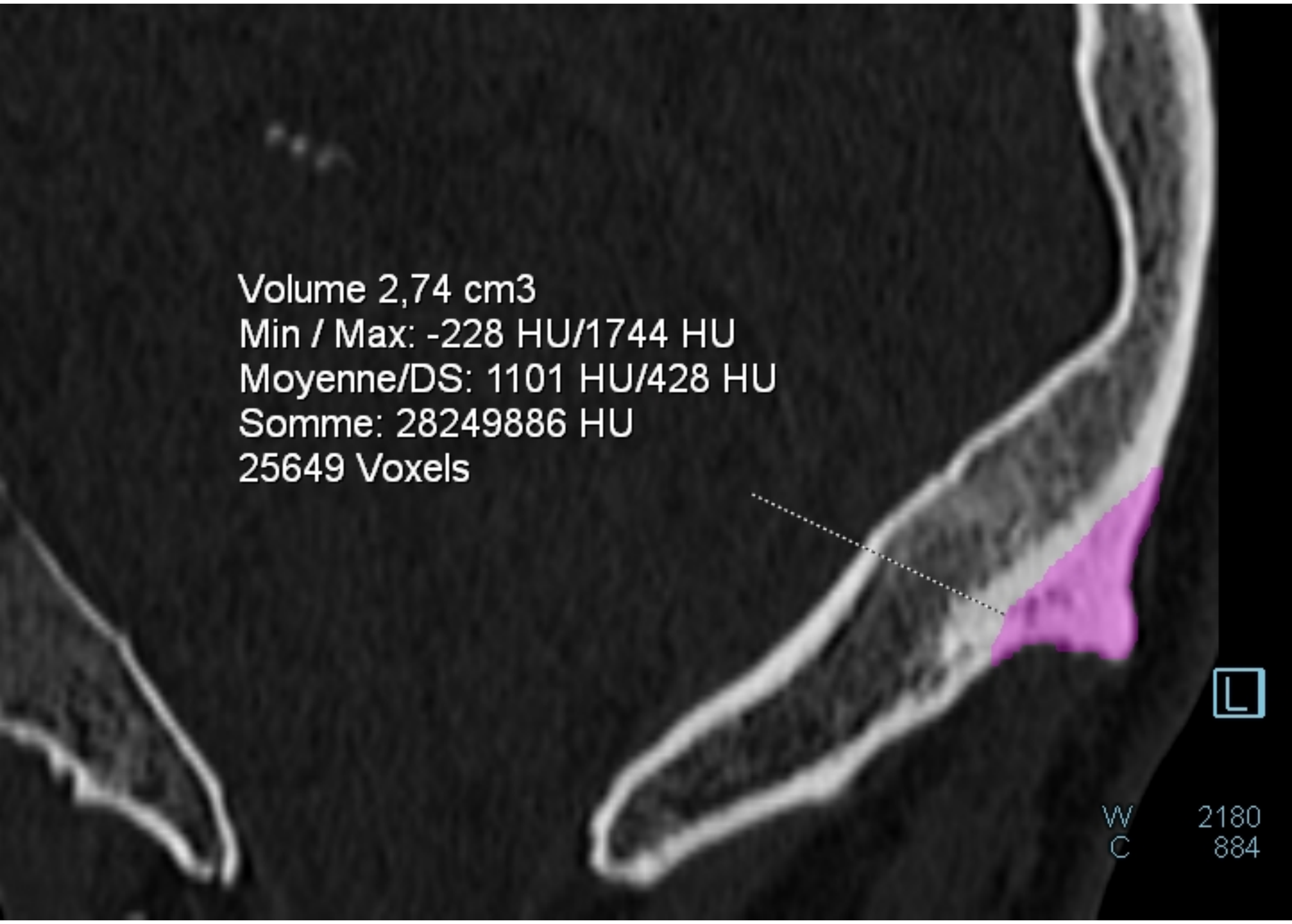
Volume 2,74 cm3
Min / Max: -228 HU/1744 HU
Moyenne/DS: 1101 HU/428 HU
Somme: 28249886 HU
25649 Voxels
L
W 2180
C 884

11m59

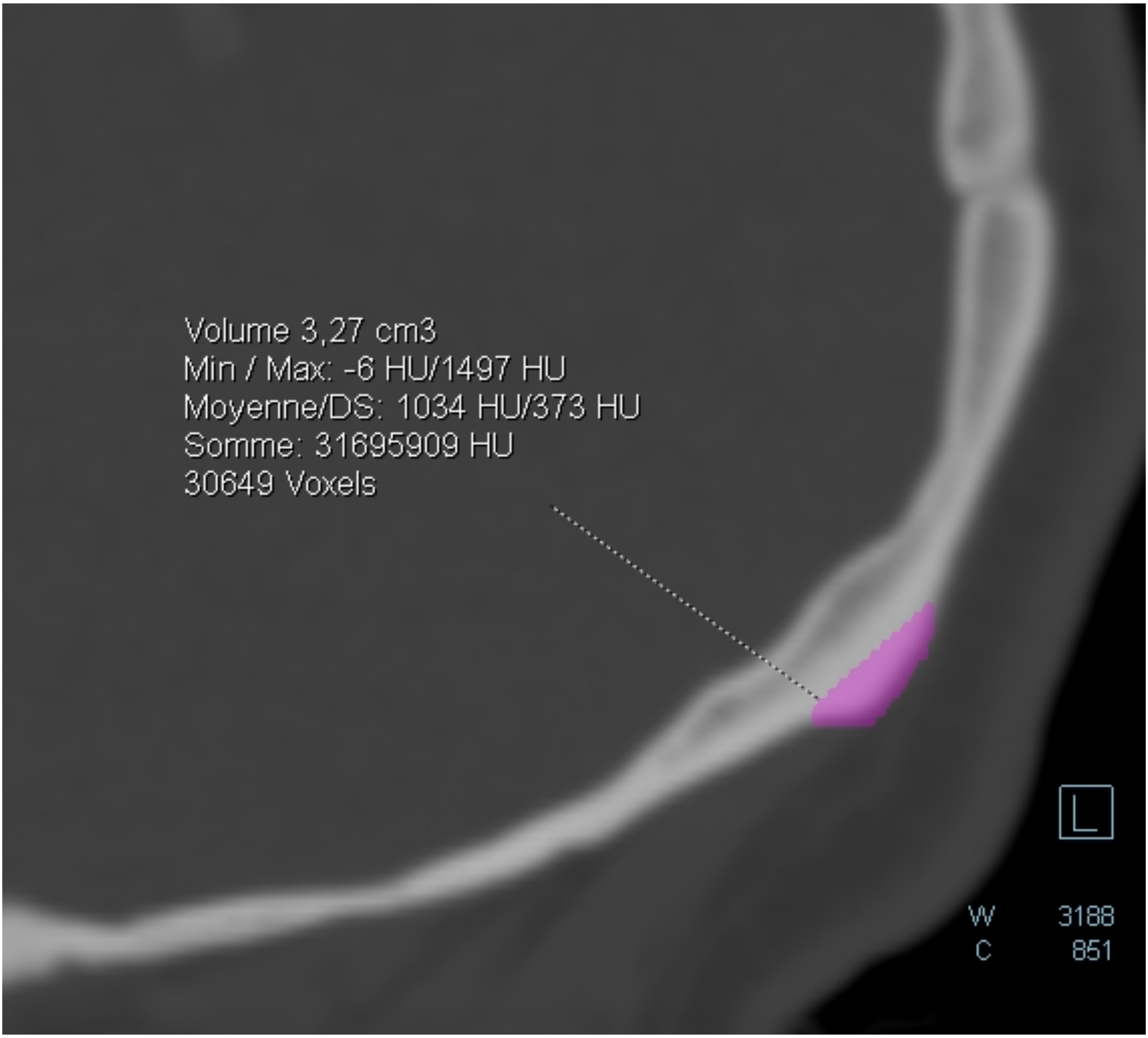

Volume 3,27 cm3
Min / Max: -6 HU/1497 HU
Moyenne/DS: 1034 HU/373 HU
Somme: 31695909 HU
30649 Voxels
L
W 3188
C 851

11m60

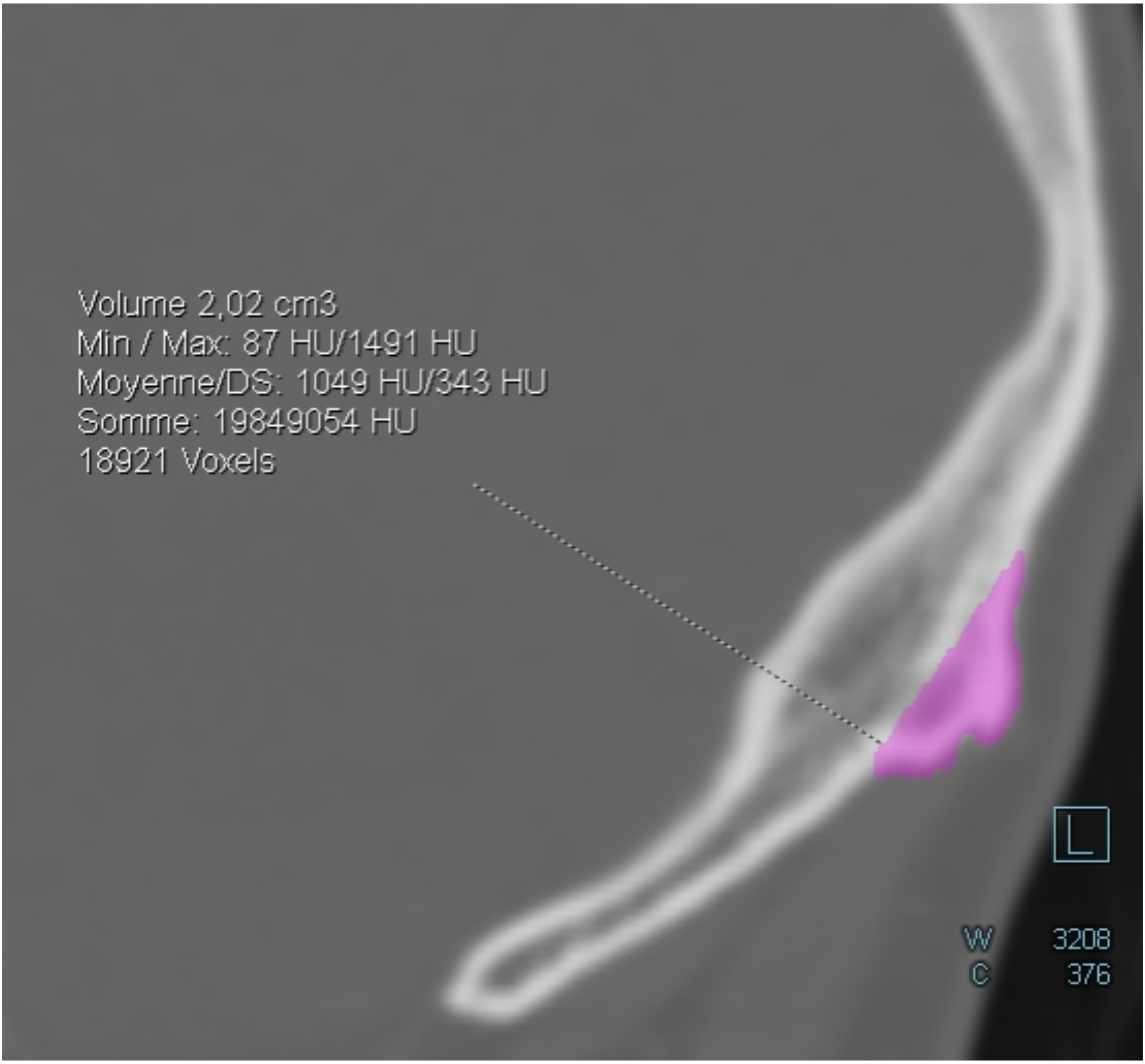

Volume 2,02 cm3
Min / Max: 87 HU/1491 HU
Moyenne/DS: 1049 HU/343 HU
Somme: 19849054 HU
18921 Voxels
L
W 3208
C 376

11m61

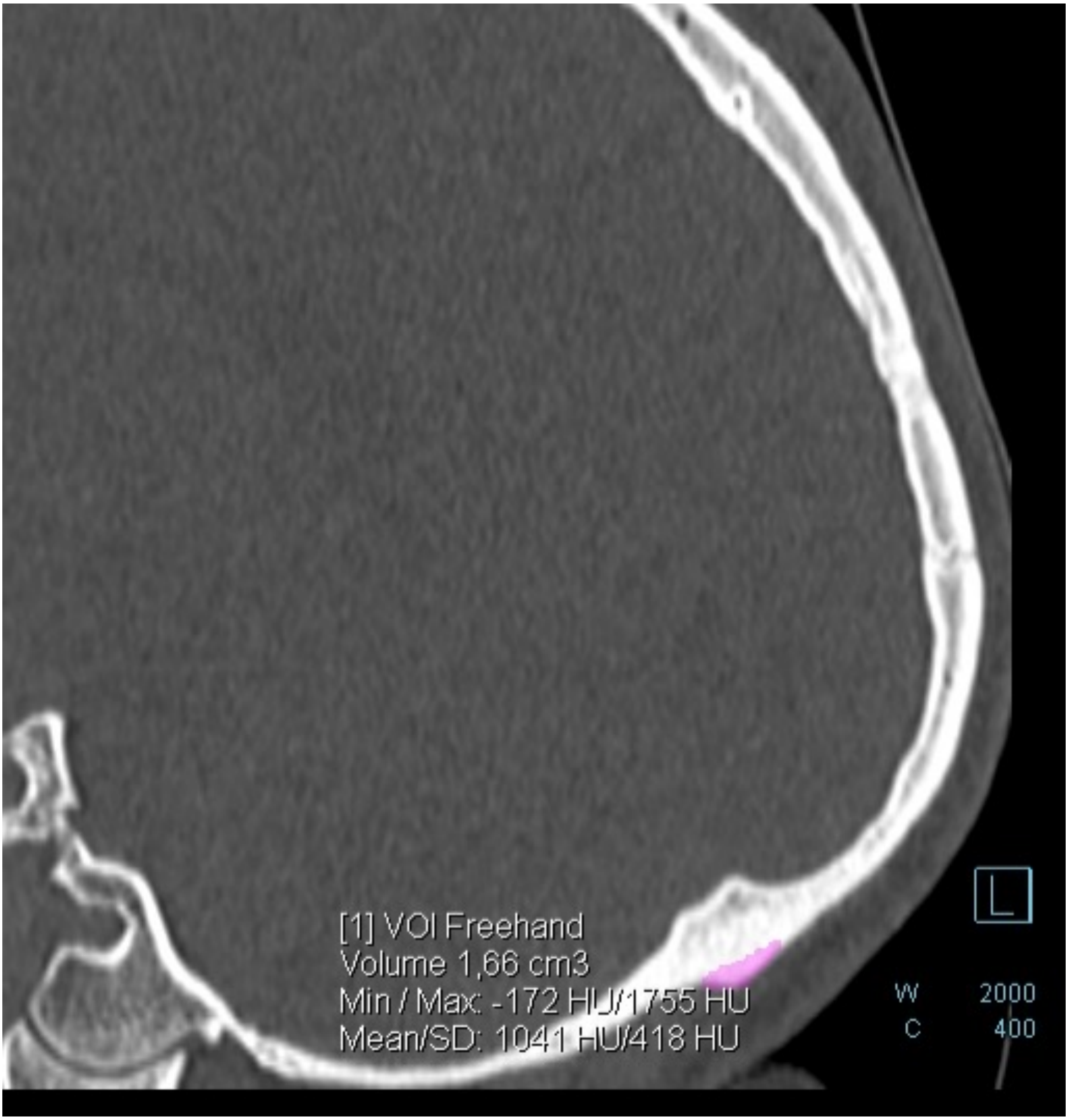
[1] VOI Freehand
Volume 1,66 cm3
Min / Max: -172 HU/1755 HU
Mean/SD: 1041 HU/418 HU
L
W 2000
C 400

11m62

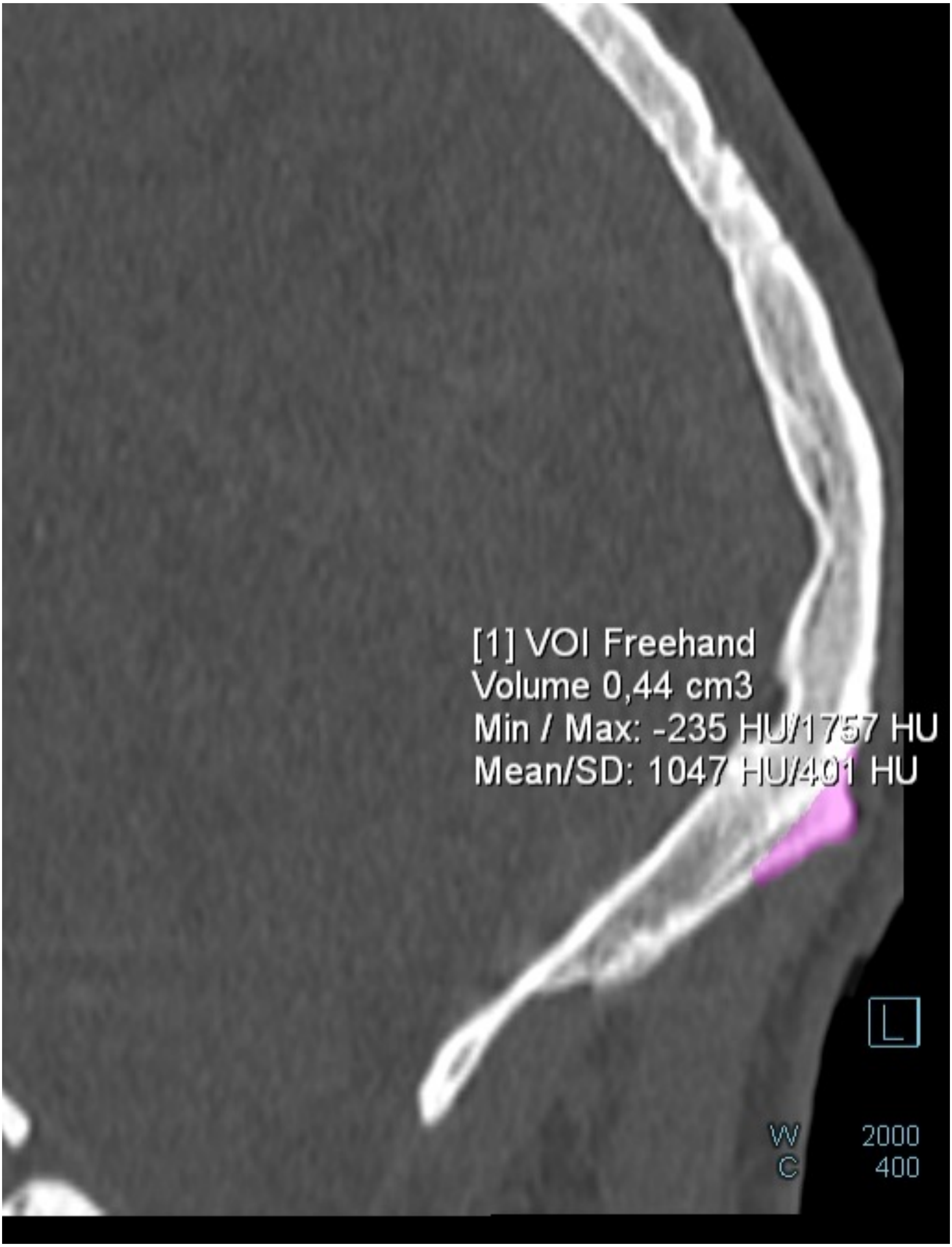
[1] VOI Freehand
Volume 0,44 cm3
Min / Max: -235 HU/1757 HU
Mean/SD: 1047 HU/401 HU
L
W 2000
C 400

11m63

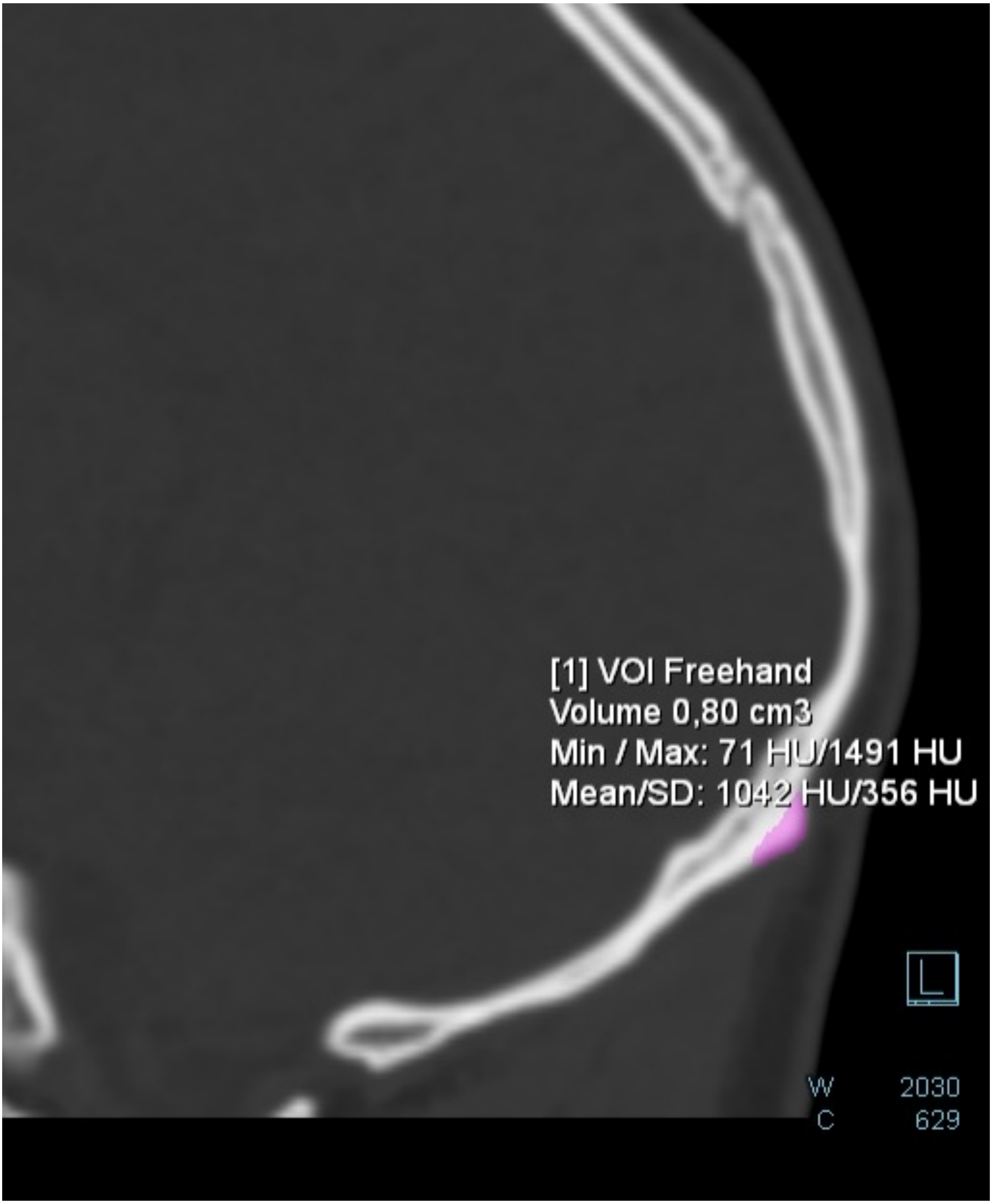
[1] VOI Freehand
Volume 0,80 cm3
Min / Max: 71 HU/1491 HU
Mean/SD: 1042 HU/356 HU
L
W 2030
C 629

11m64

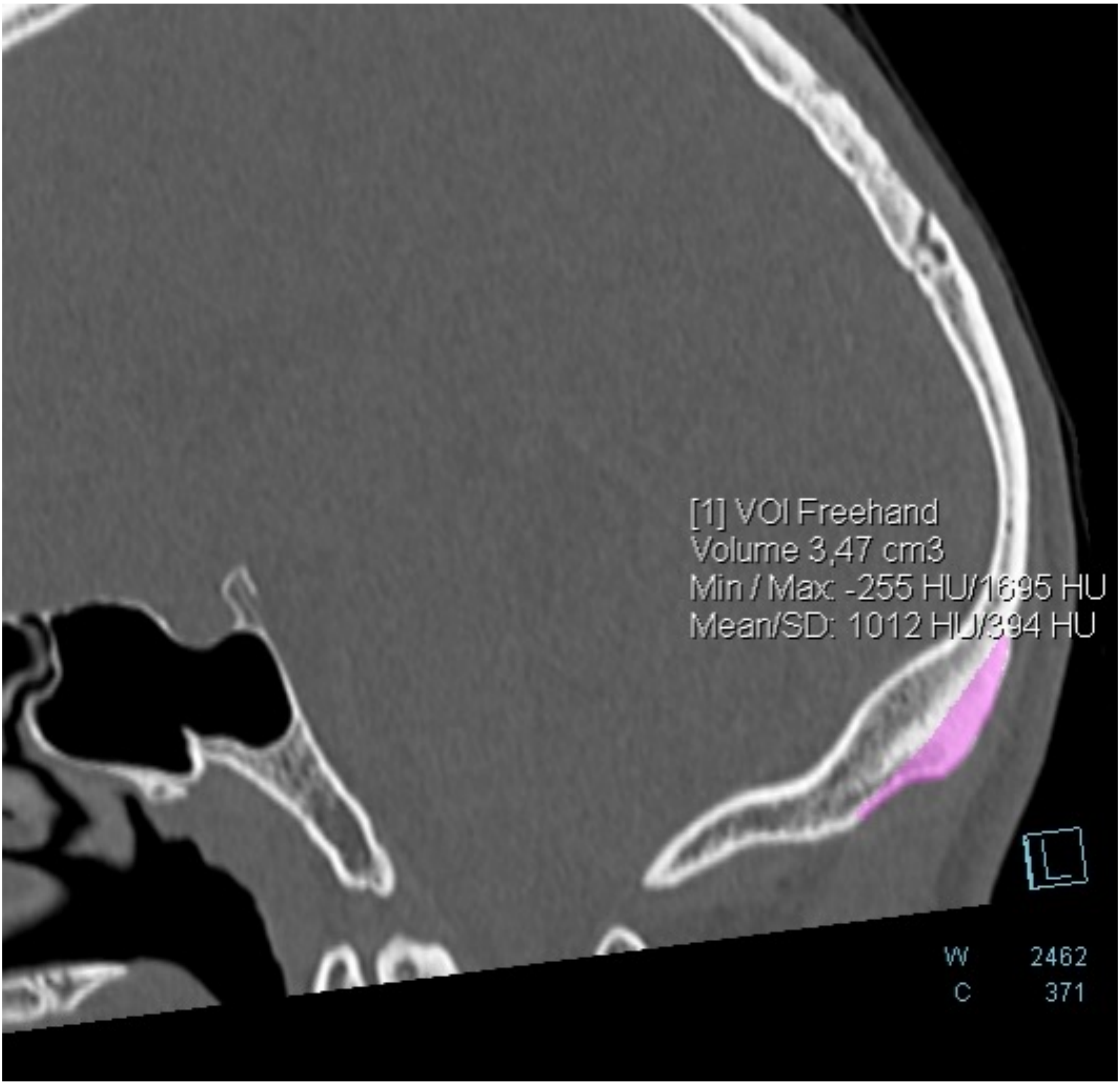
[1] VOI Freehand
Volume 3,47 cm3
Min / Max: -255 HU/1695 HU
Mean/SD: 1012 HU/394 HU
W 2462
C 371

11m65

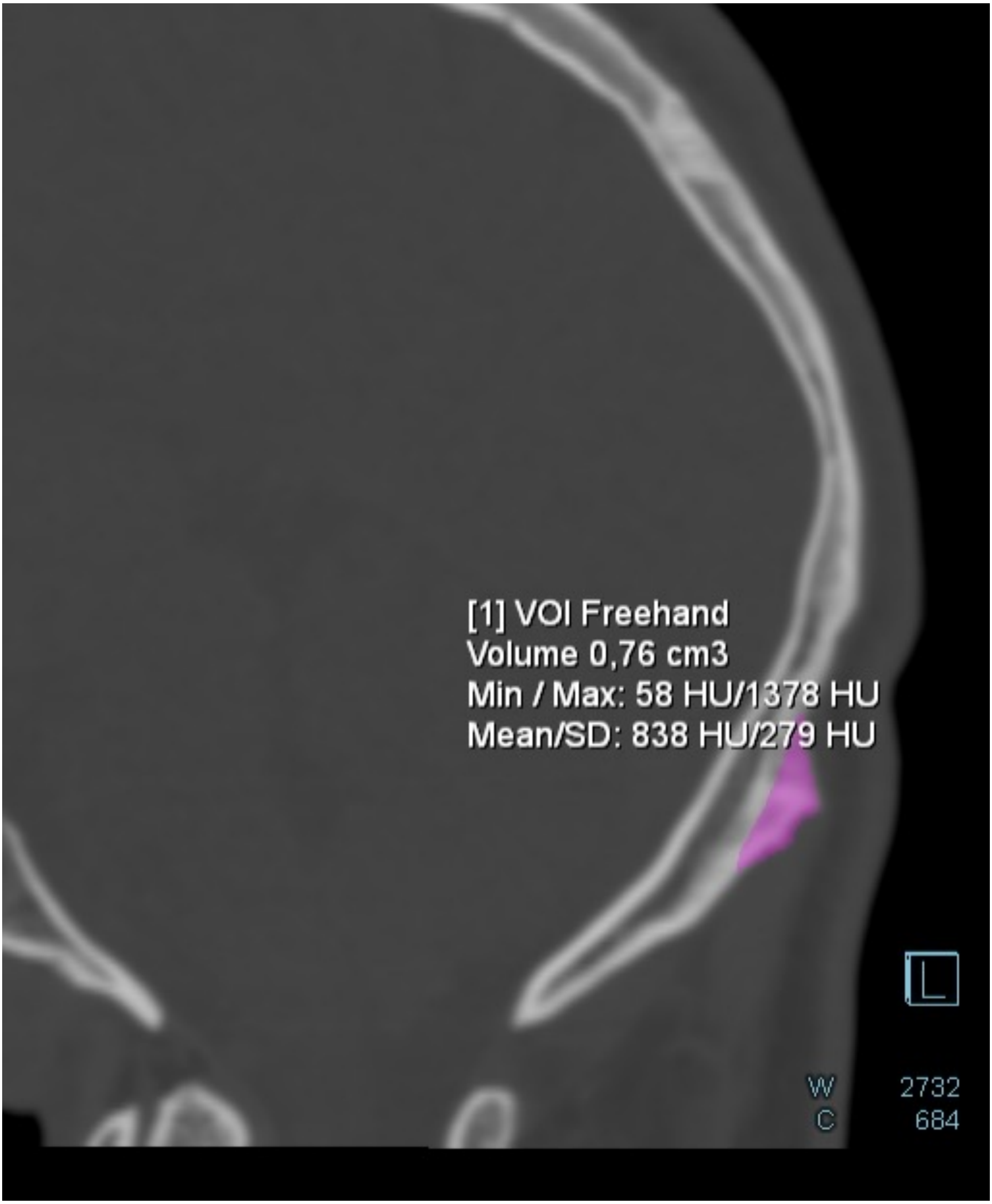
[1] VOI Freehand
Volume 0,76 cm3
Min / Max: 58 HU/1378 HU
Mean/SD: 838 HU/279 HU
L
W 2732
C 684

11m66

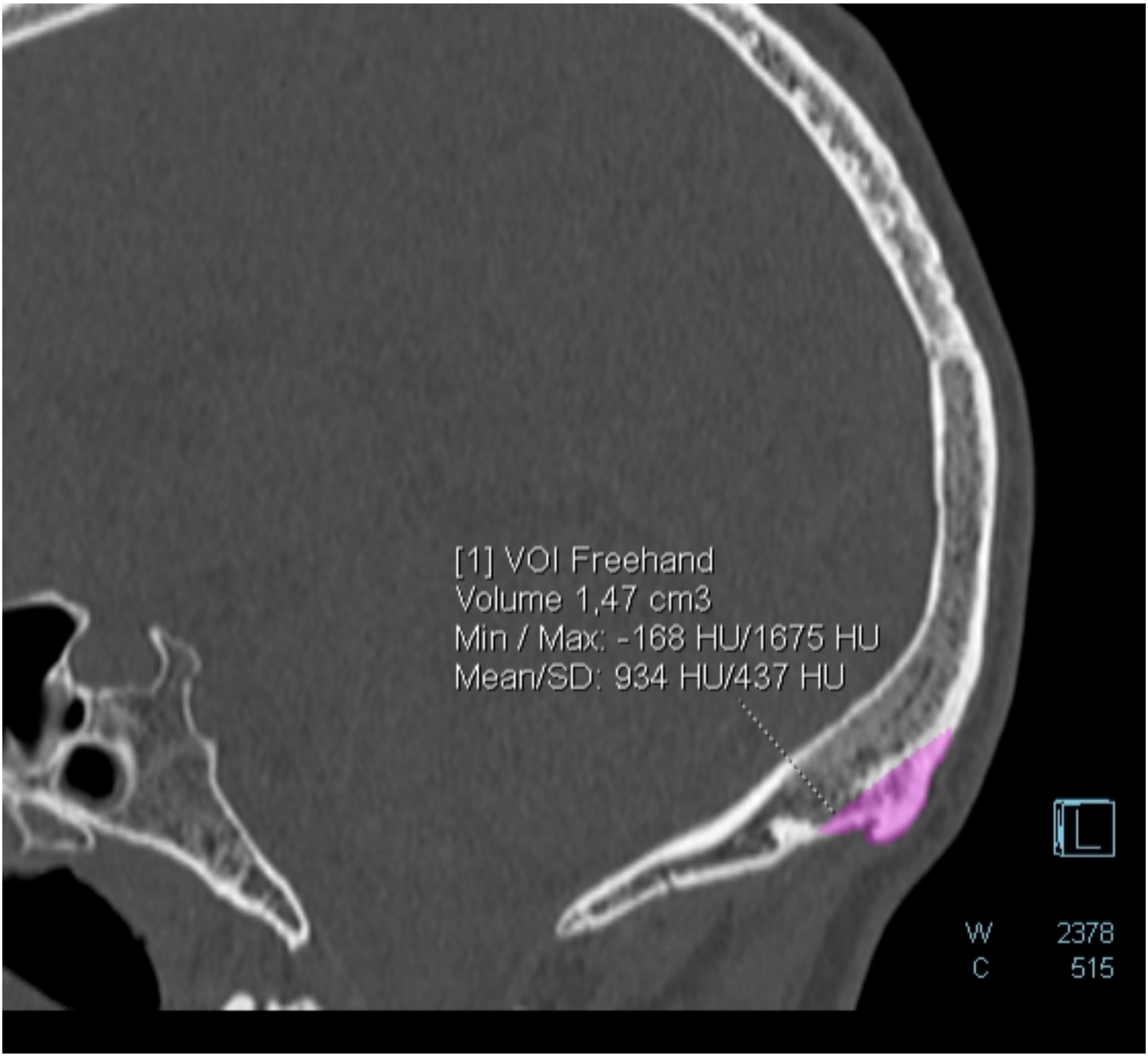

[1] VOI Freehand
Volume 1,47 cm3
Min / Max: -168 HU/1675 HU
Mean/SD: 934 HU/437 HU
L
W 2378
C 515

11m67

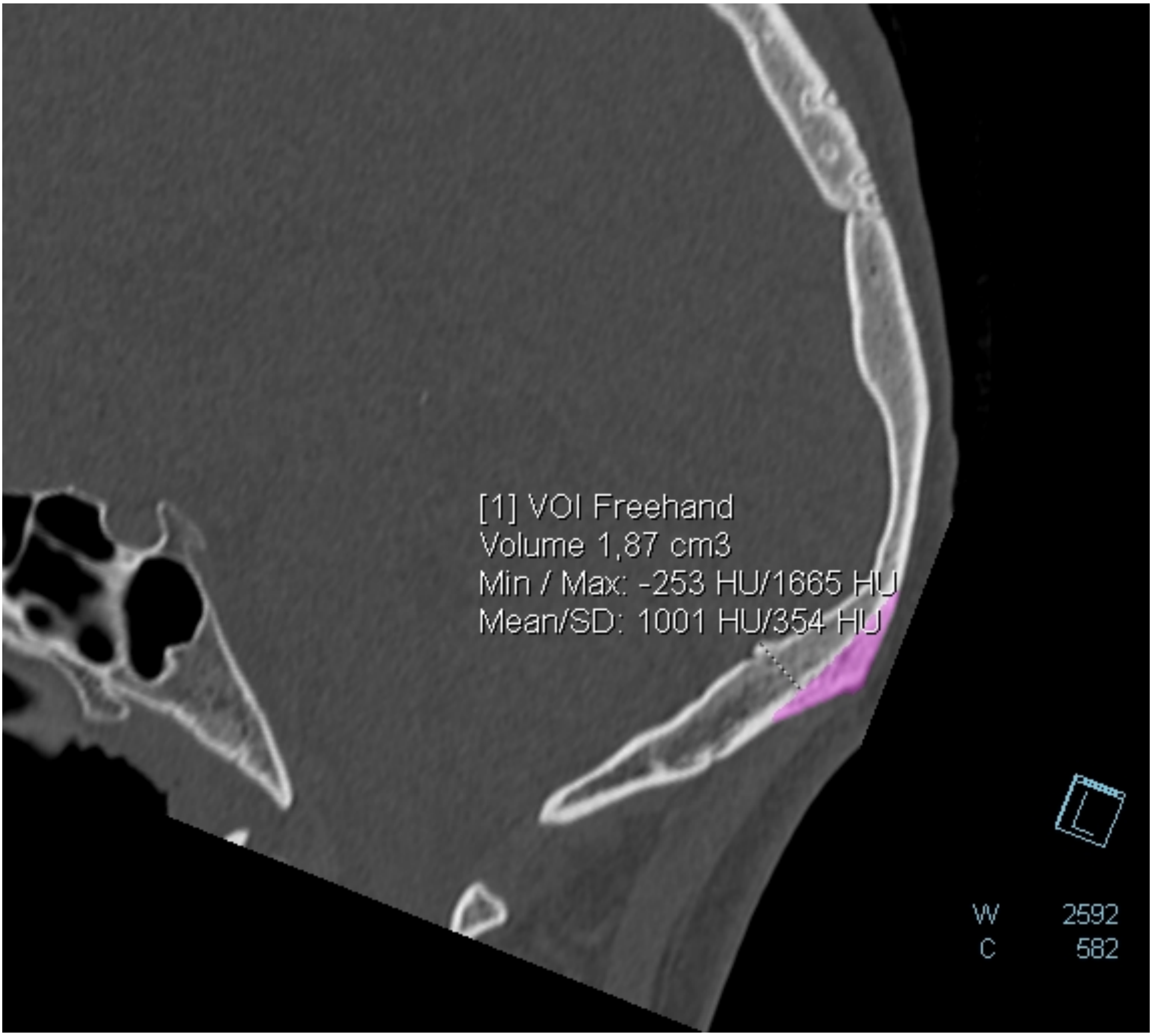

[1] VOI Freehand
Volume 1,87 cm3
Min / Max: -253 HU/1665 HU
Mean/SD: 1001 HU/354 HU
W 2592
C 582

11m68

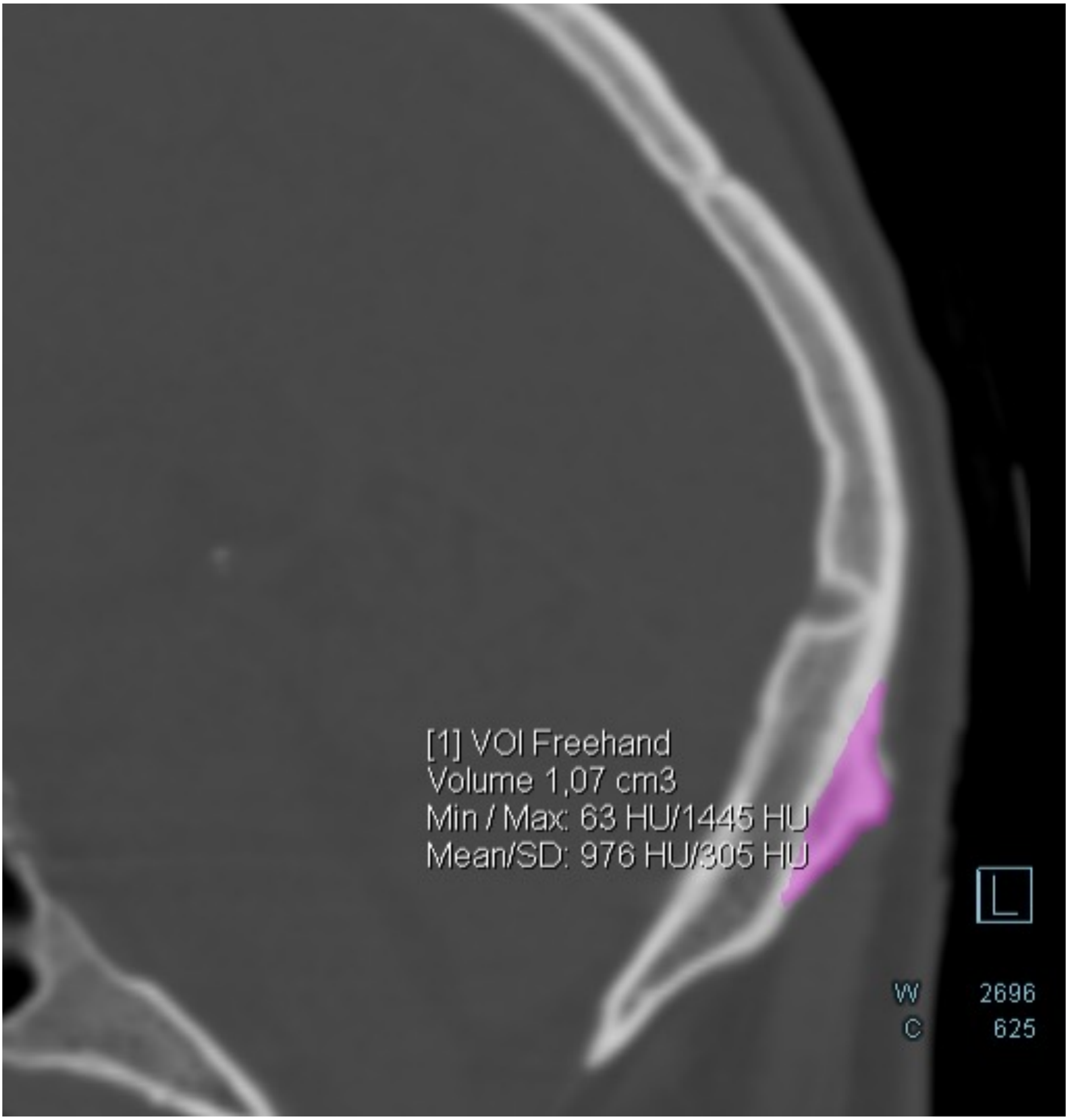

[1] VOI Freehand
Volume 1,07 cm3
Min / Max: 63 HU/1445 HU
Mean/SD: 976 HU/305 HU
L
W 2696
C 625

11m69

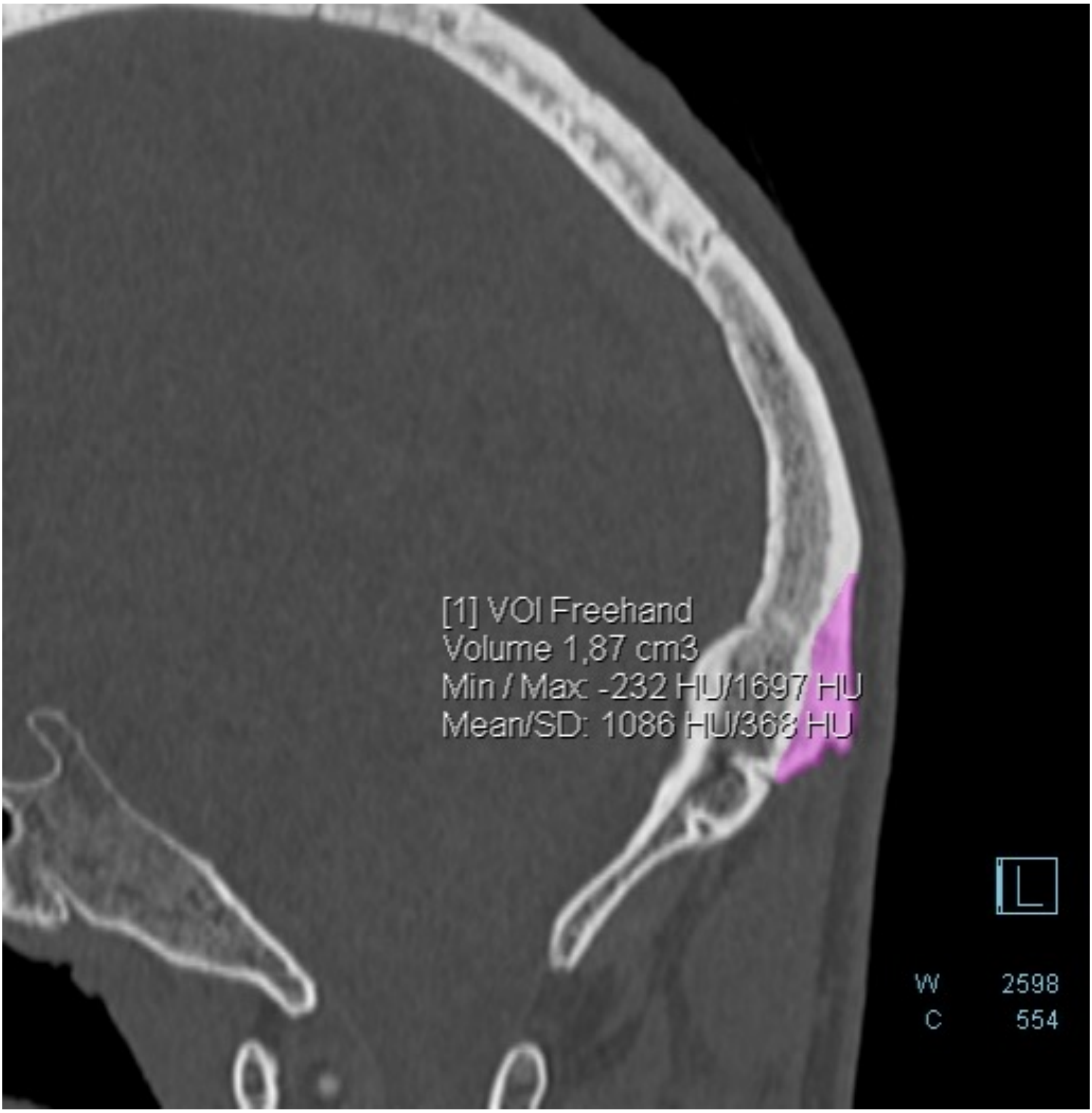
[1] VOI Freehand
Volume 1,87 cm3
Min / Max: -232 HU/1697 HU
Mean/SD: 1086 HU/368 HU
L
W 2598
C 554

11m70

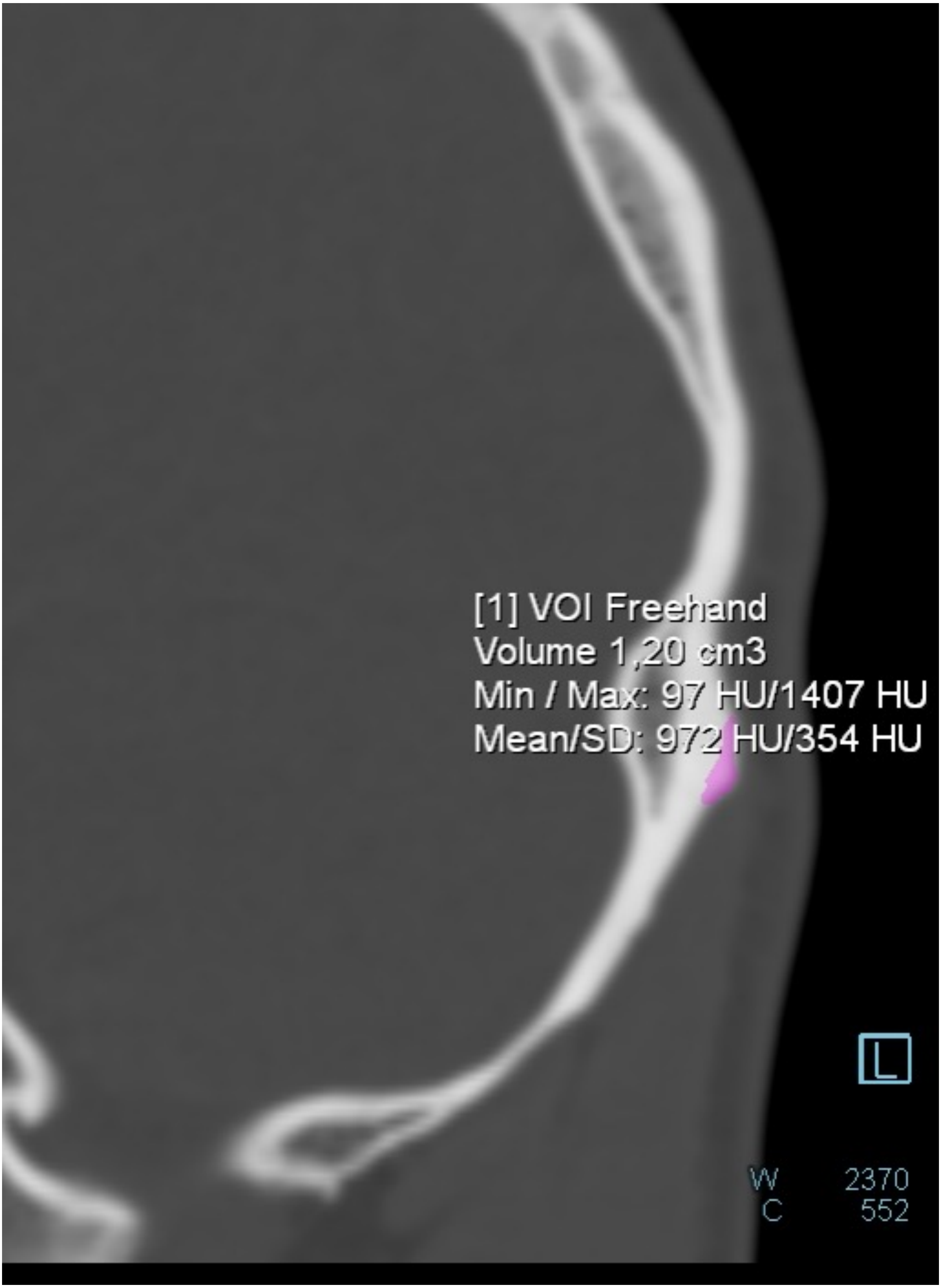
[1] VOI Freehand
Volume 1,20 cm3
Min / Max: 97 HU/1407 HU
Mean/SD: 972 HU/354 HU
L
W 2370
C 552

11m71

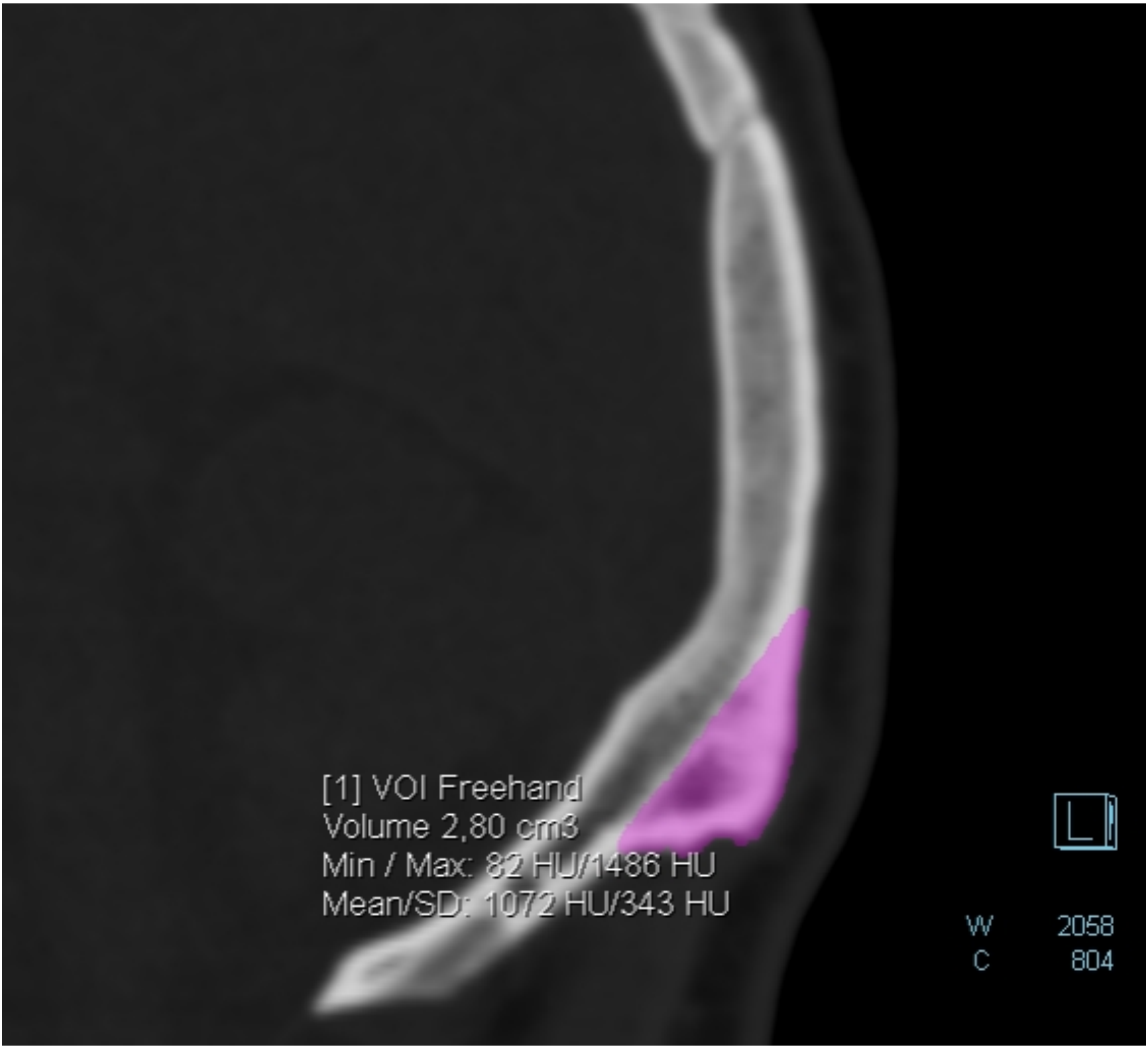
[1] VOI Freehand
Volume 2,80 cm3
Min / Max: 82 HU/1486 HU
Mean/SD: 1072 HU/343 HU
L
W 2058
C 804

11m72

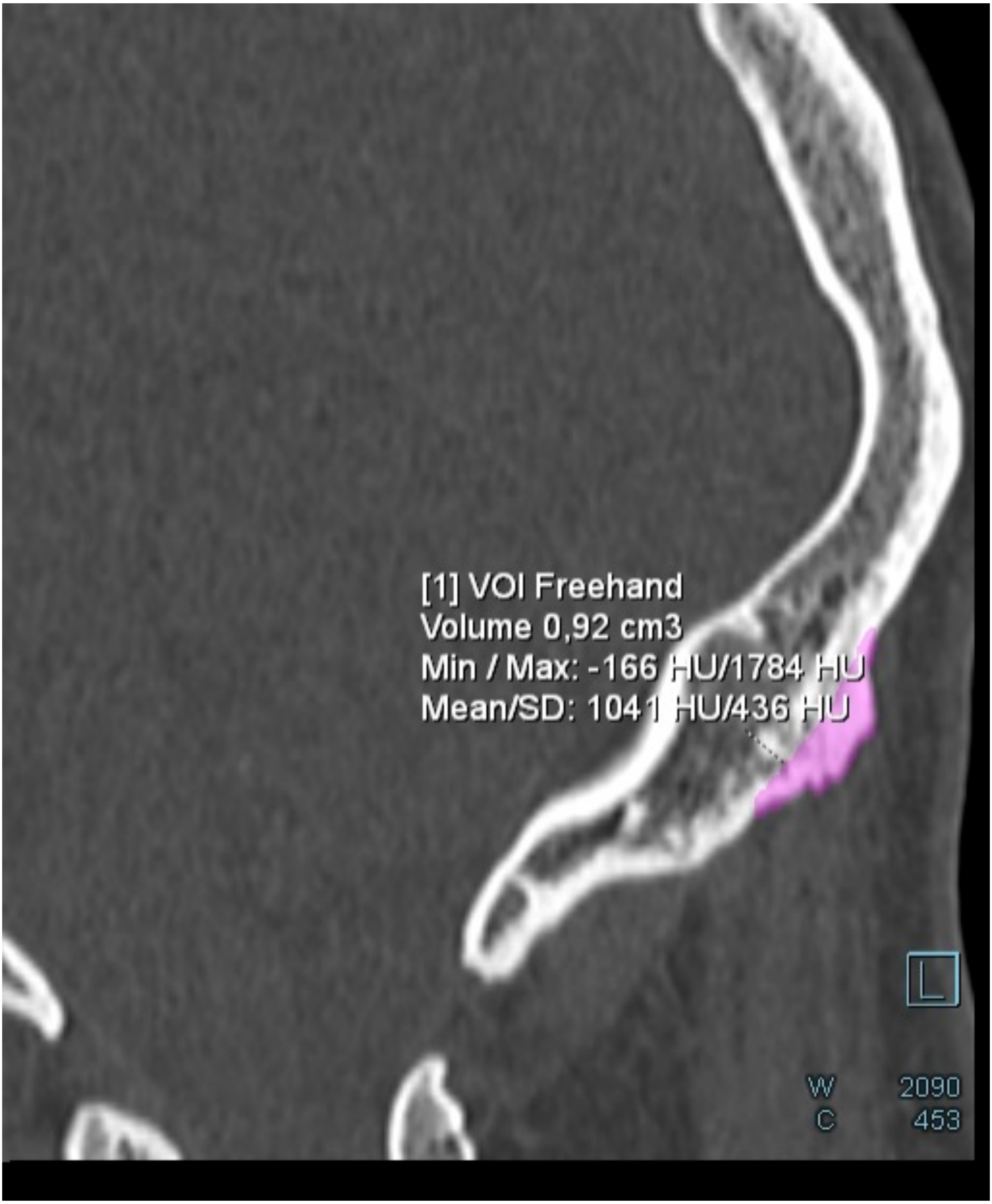
[1] VOI Freehand
Volume 0,92 cm3
Min / Max: -166 HU/1784 HU
Mean/SD: 1041 HU/436 HU
L
W 2090
C 453

11m73a

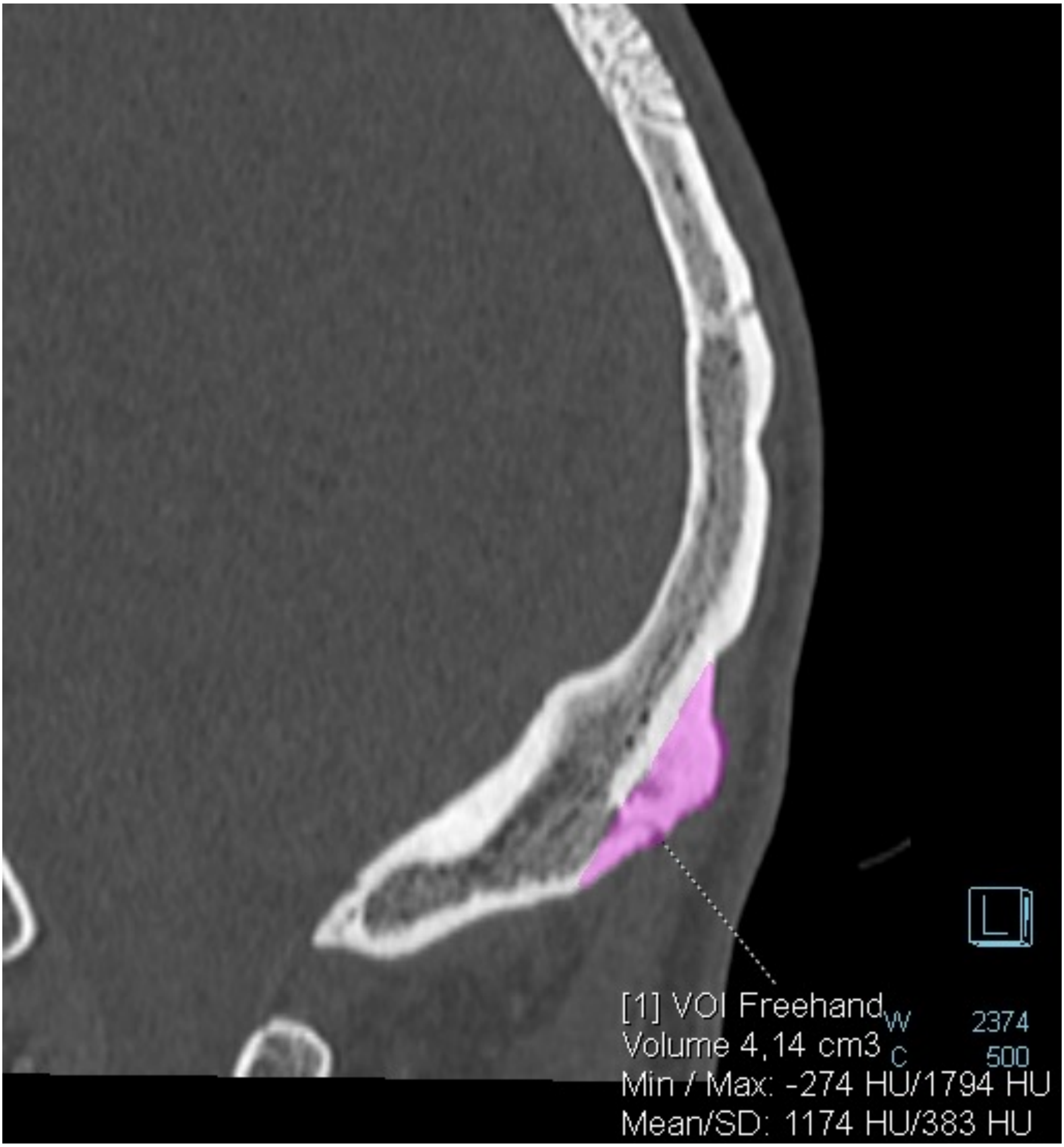

[1] VOI Freehand
Volume 4,14 cm3
Min / Max: -274 HU/1794 HU
Mean/SD: 1174 HU/383 HU
W 2374
C 500
L

11m74

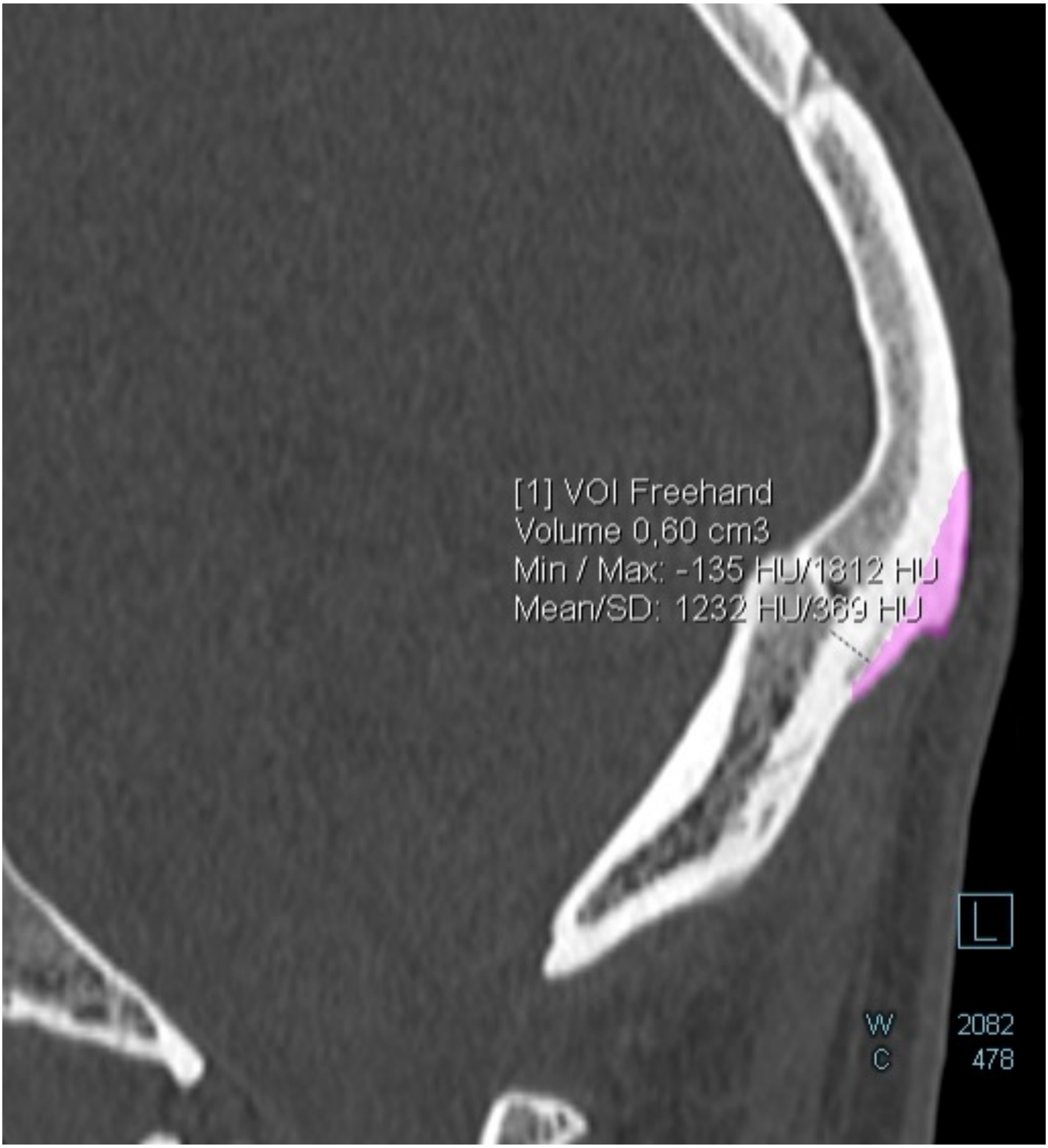
[1] VOI Freehand
Volume 0,60 cm3
Min / Max: -135 HU/1812 HU
Mean/SD: 1232 HU/369 HU
L
W 2082
C 478

11m75

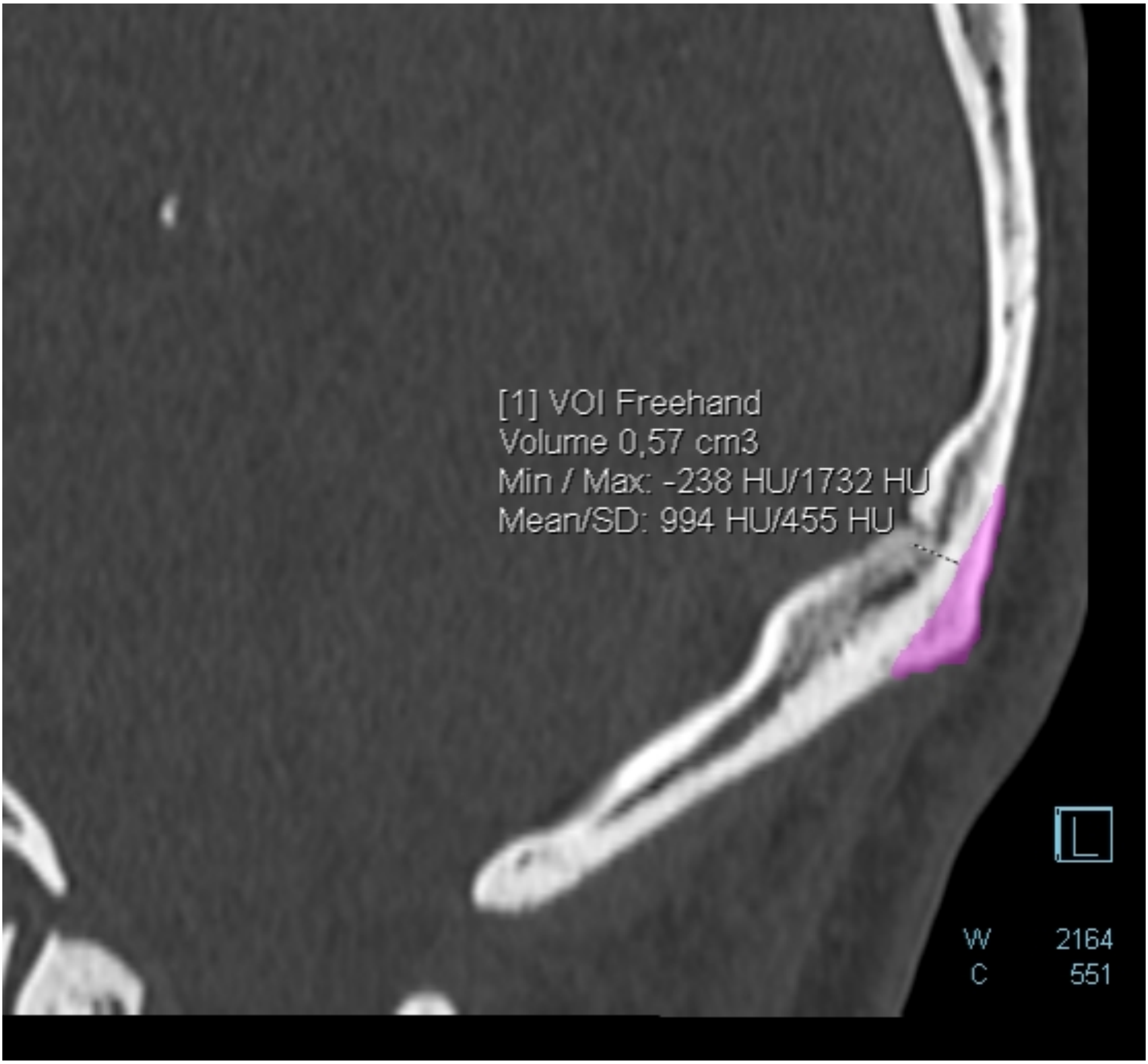

[1] VOI Freehand
Volume 0,57 cm3
Min / Max: -238 HU/1732 HU
Mean/SD: 994 HU/455 HU
L
W 2164
C 551

11m76a

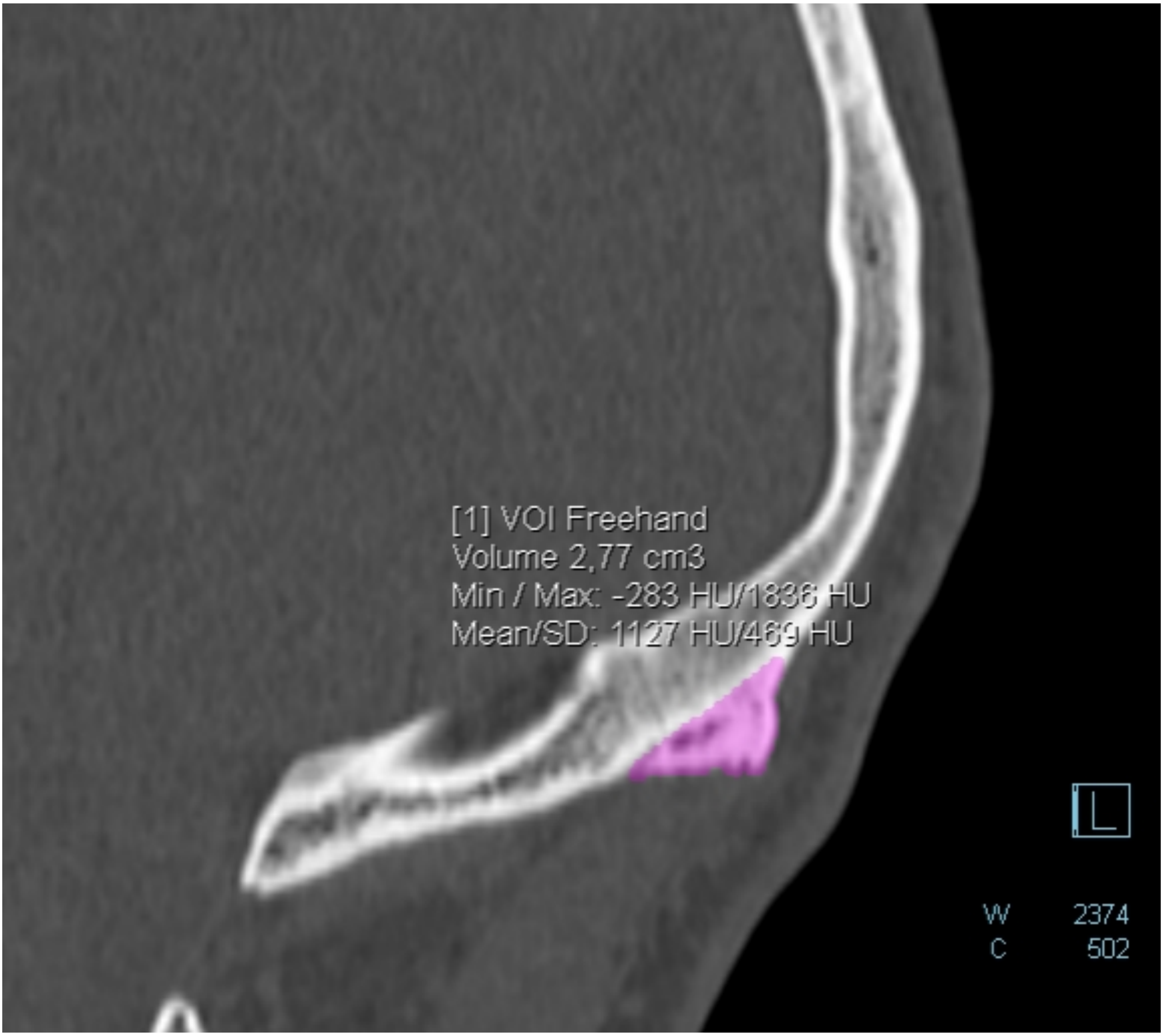

[1] VOI Freehand
Volume 2,77 cm3
Min / Max: -283 HU/1836 HU
Mean/SD: 1127 HU/469 HU
L
W 2374
C 502

11m77

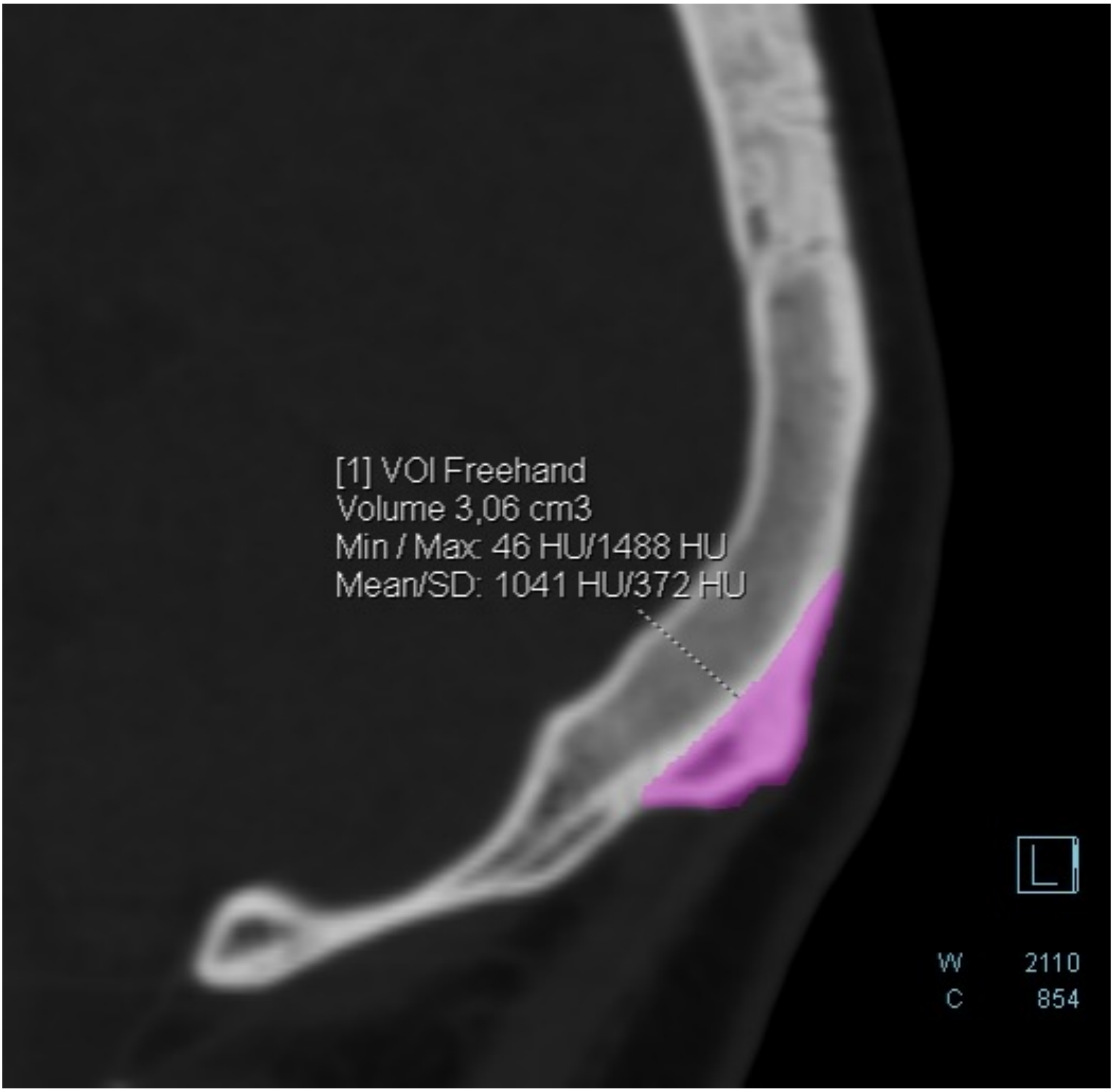

[1] VOI Freehand
Volume 3,06 cm3
Min / Max: 46 HU/1488 HU
Mean/SD: 1041 HU/372 HU
L
W 2110
C 854

19f1

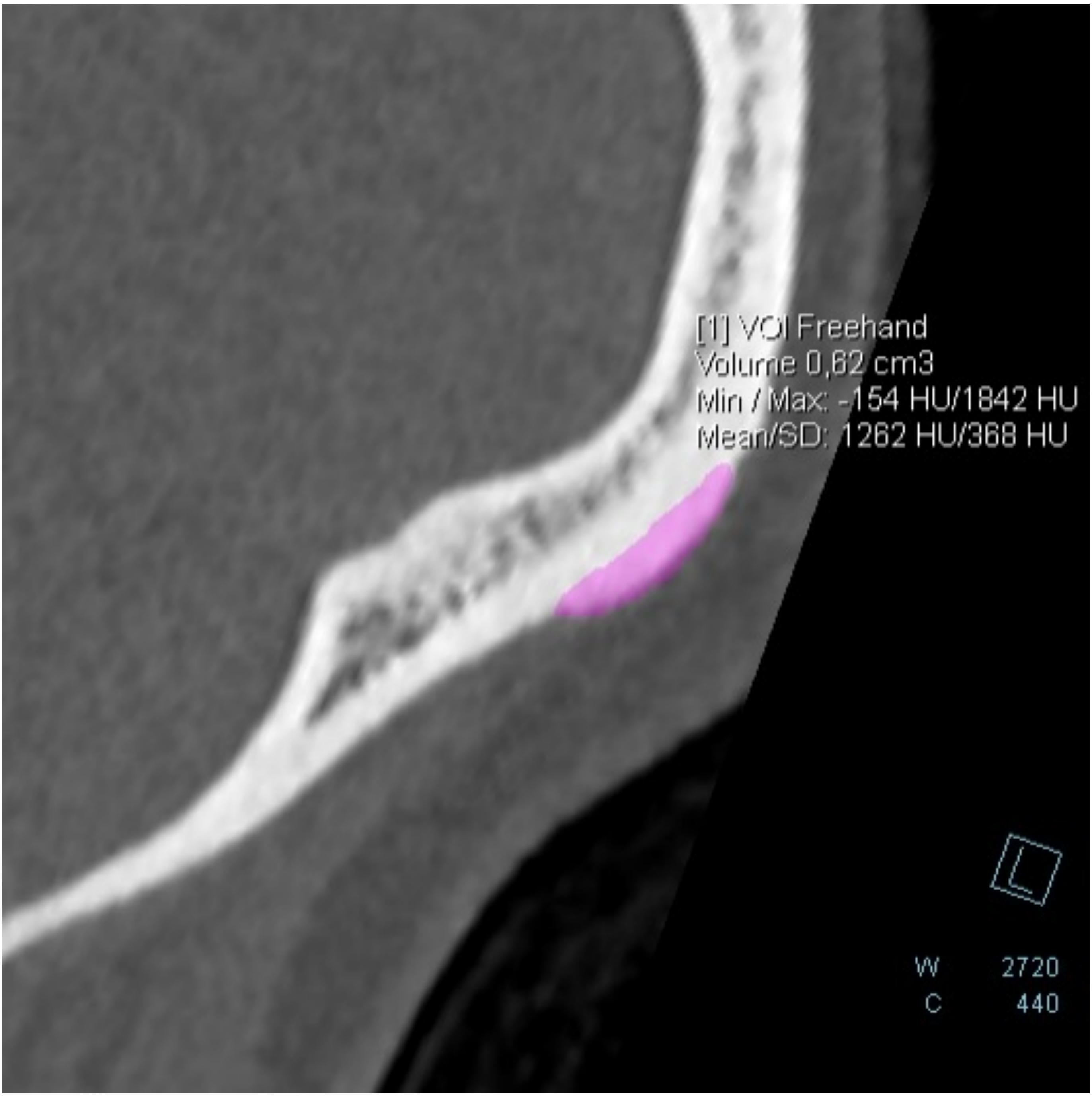
[1] VOI Freehand
Volume 0,62 cm3
Min / Max: -154 HU/1842 HU
Mean/SD: 1262 HU/368 HU
W 2720
C 440

19f2

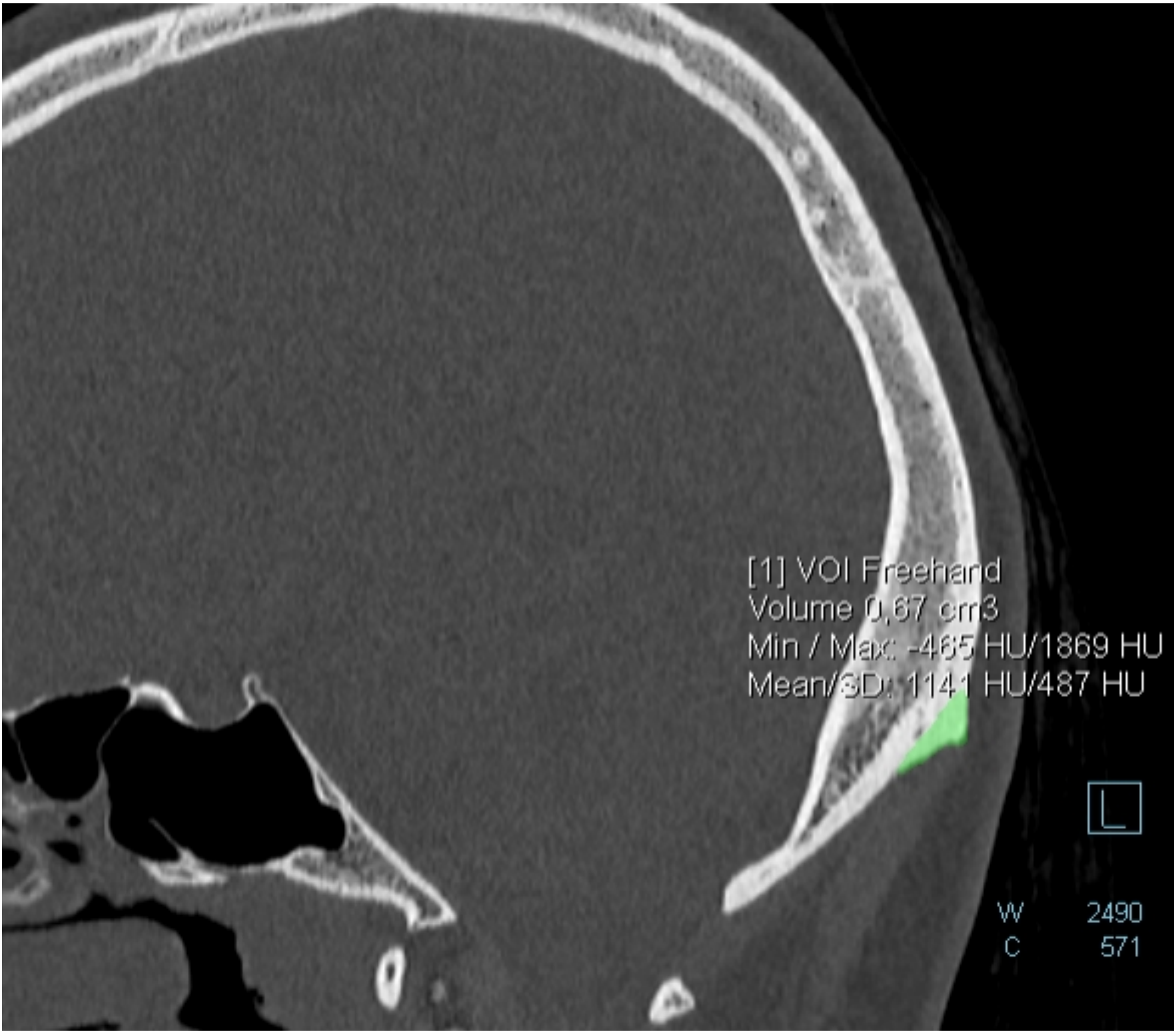

[1] VOI Freehand
Volume 0,67 cm3
Min / Max: -465 HU/1869 HU
Mean/SD: 1141 HU/487 HU
L
W 2490
C 571

19f3

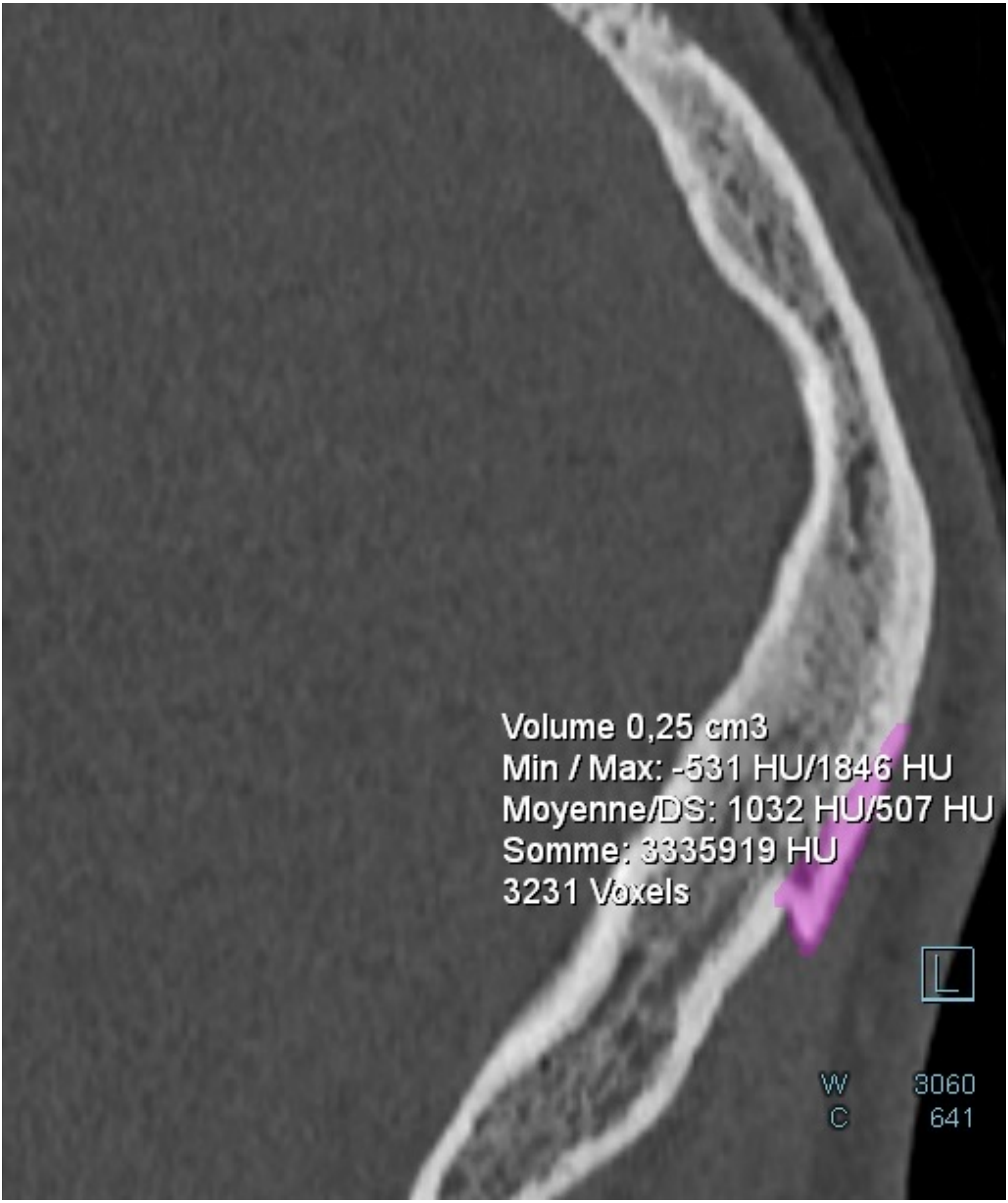
Volume 0,25 cm3
Min / Max: -531 HU/1846 HU
Moyenne/DS: 1032 HU/507 HU
Somme: 3335919 HU
3231 Voxels
L
W 3060
C 641

19f4

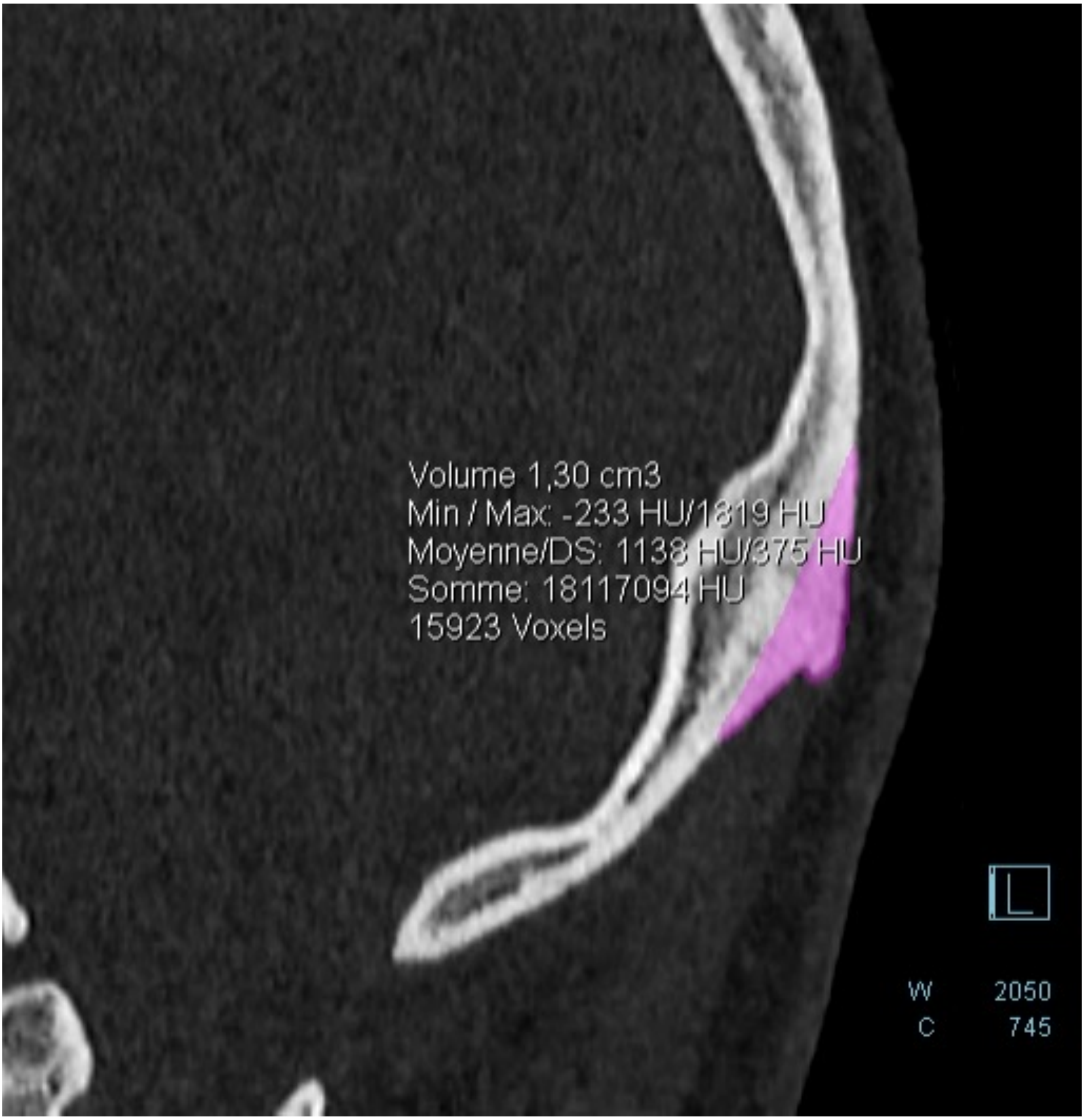

Volume 1,30 cm3
Min / Max: -233 HU/1819 HU
Moyenne/DS: 1138 HU/375 HU
Somme: 18117094 HU
15923 Voxels
L
W 2050
C 745

19f5

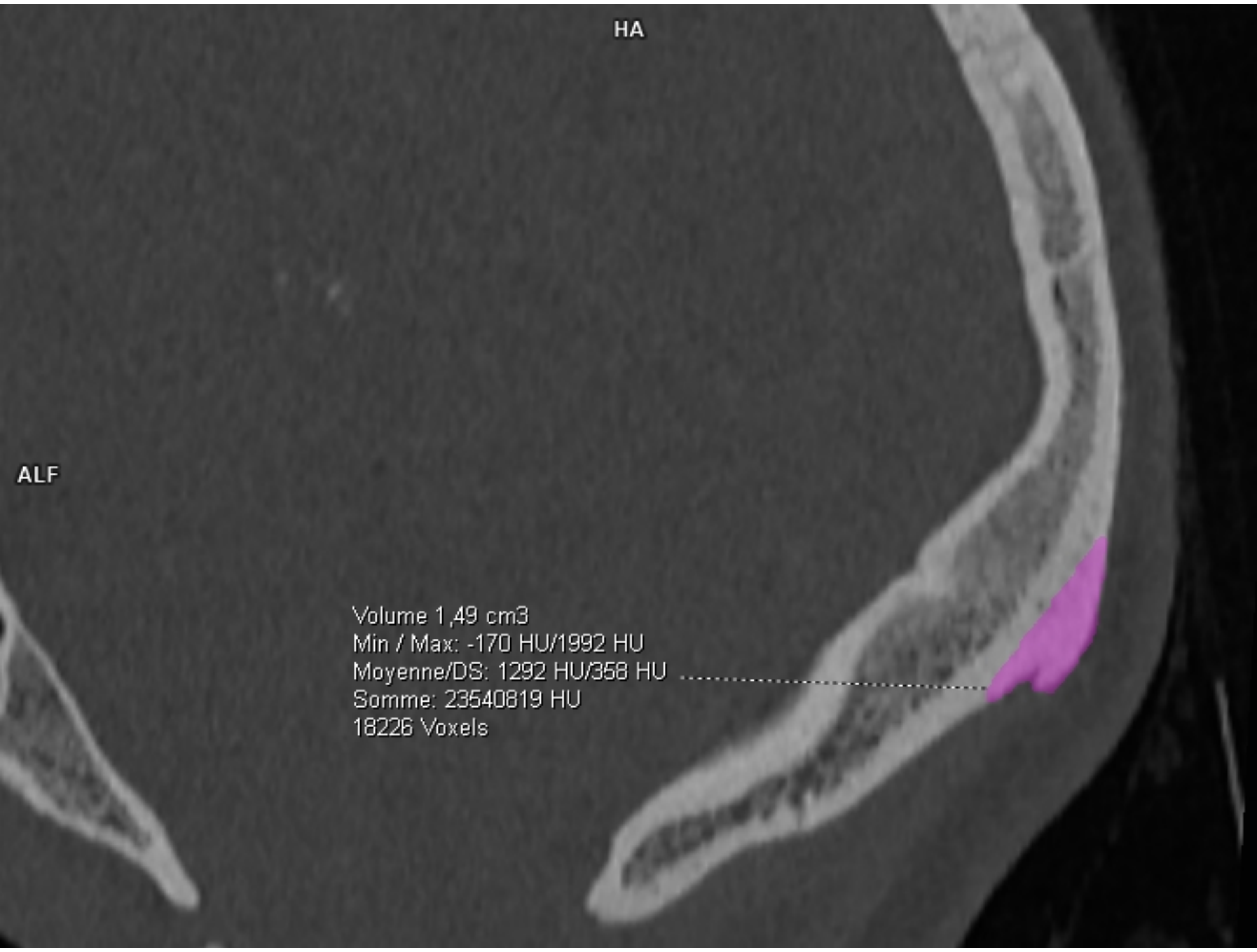
HA
ALF
Volume 1,49 cm3
Min / Max: -170 HU/1992 HU
Moyenne/DS: 1292 HU/358 HU
Somme: 23540819 HU
18226 Voxels

19f6

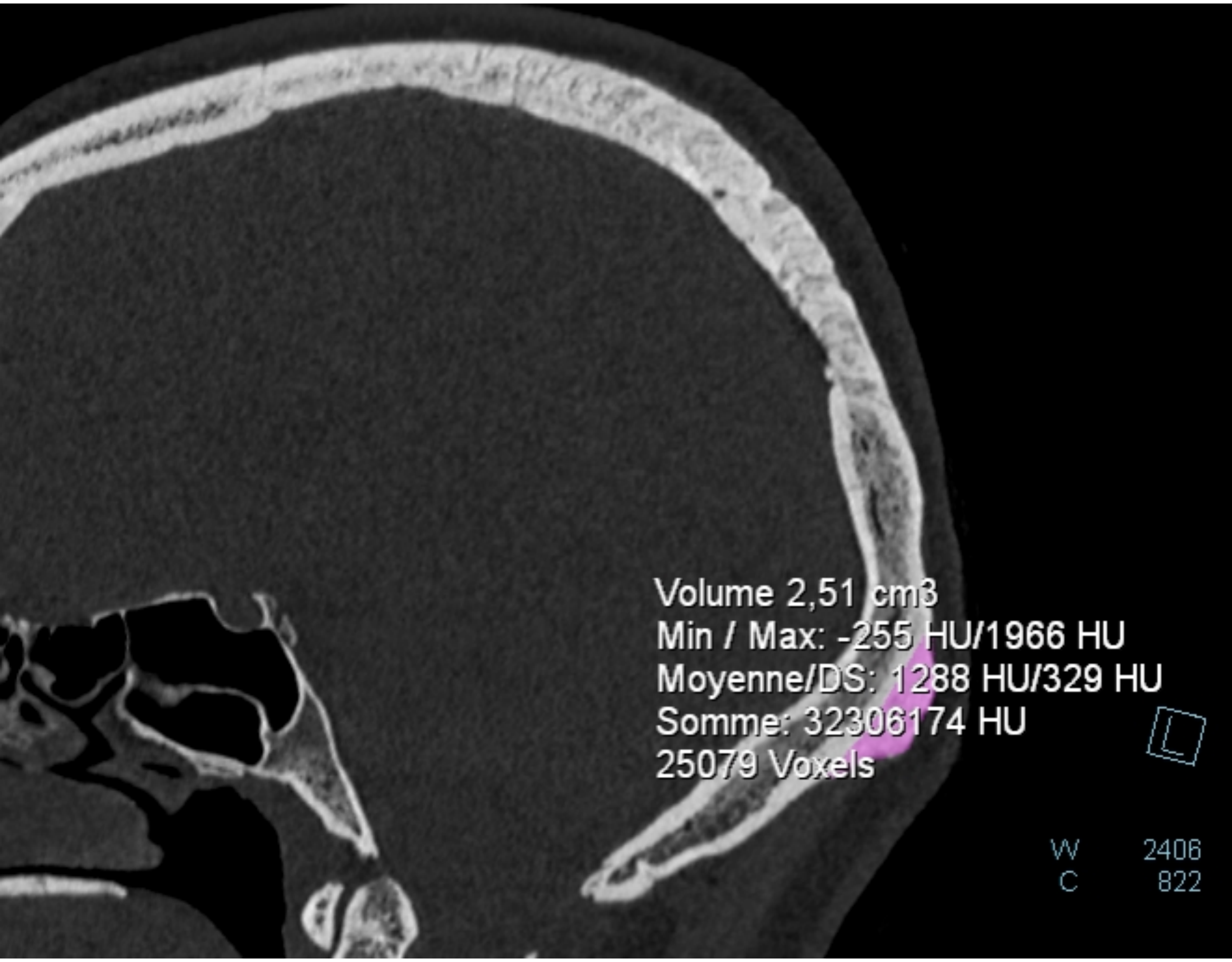

Volume 2,51 cm3
Min / Max: -255 HU/1966 HU
Moyenne/DS: 1288 HU/329 HU
Somme: 32306174 HU
25079 Voxels
W 2406
C 822

19f7

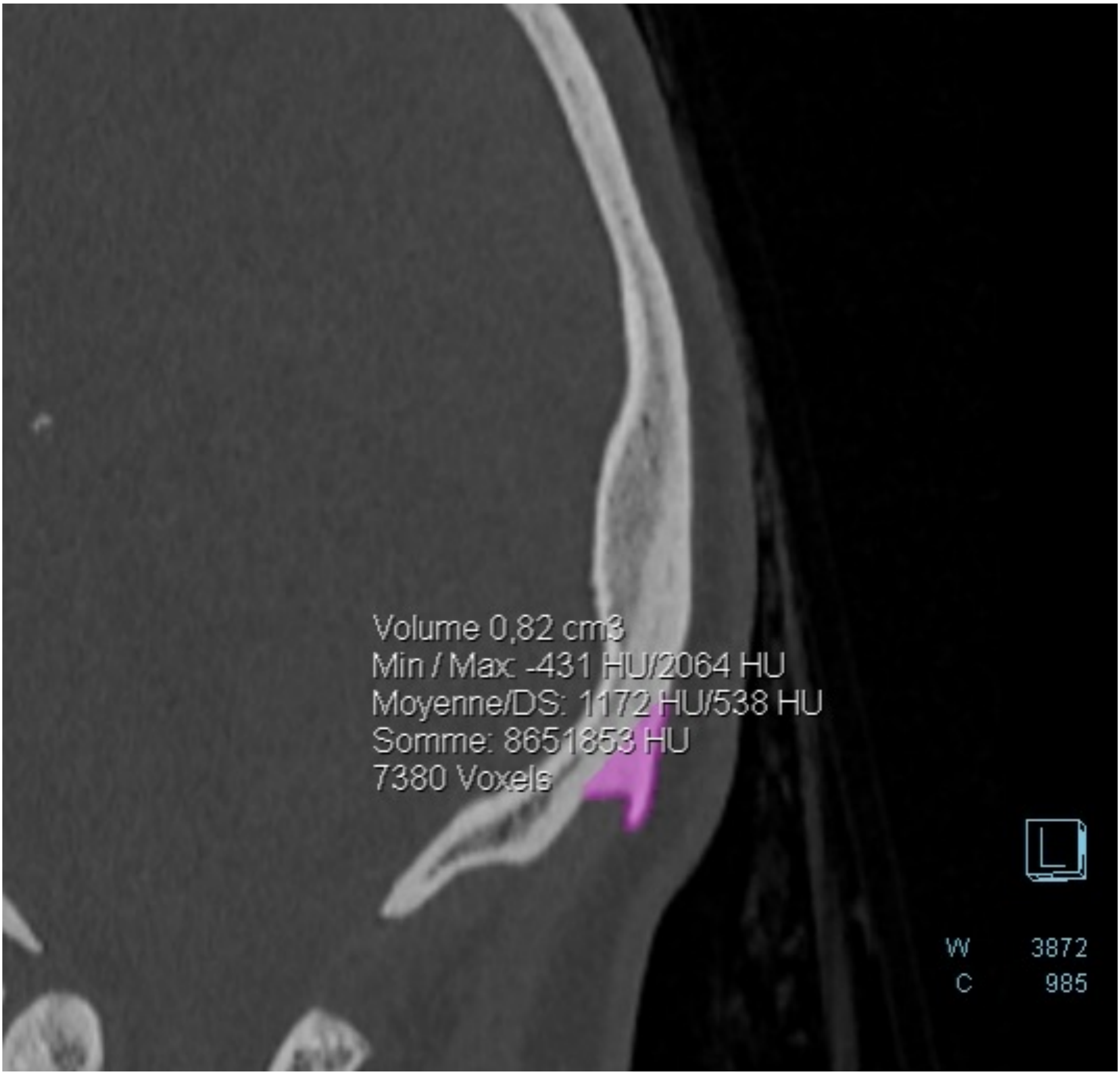
Volume 0,82 cm3
Min / Max: -431 HU/2064 HU
Moyenne/DS: 1172 HU/538 HU
Somme: 8651853 HU
7380 Voxels
W 3872
C 985

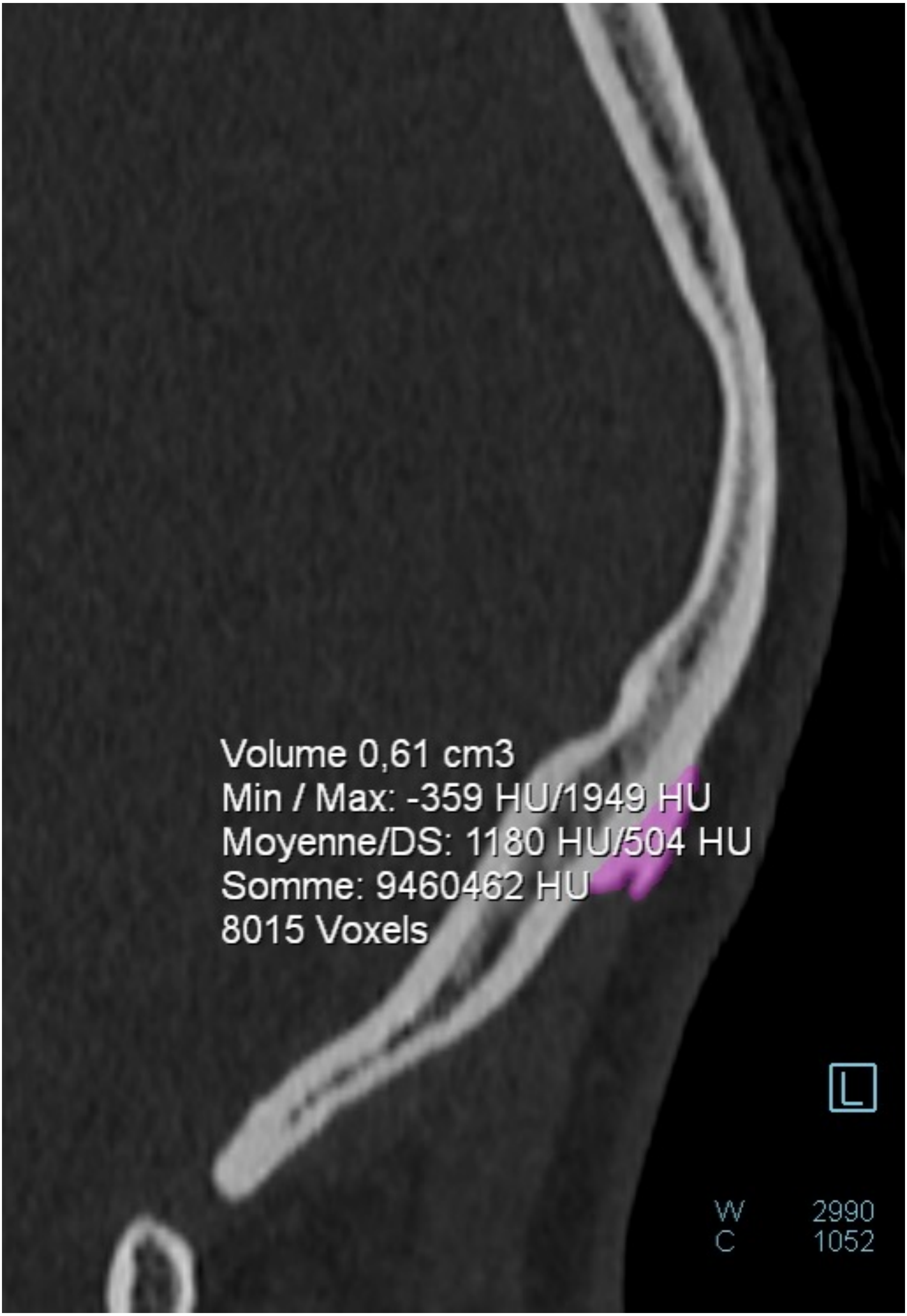
Volume 0,61 cm3
Min / Max: -359 HU/1949 HU
Moyenne/DS: 1180 HU/504 HU
Somme: 9460462 HU
8015 Voxels
L
W 2990
C 1052

19f9

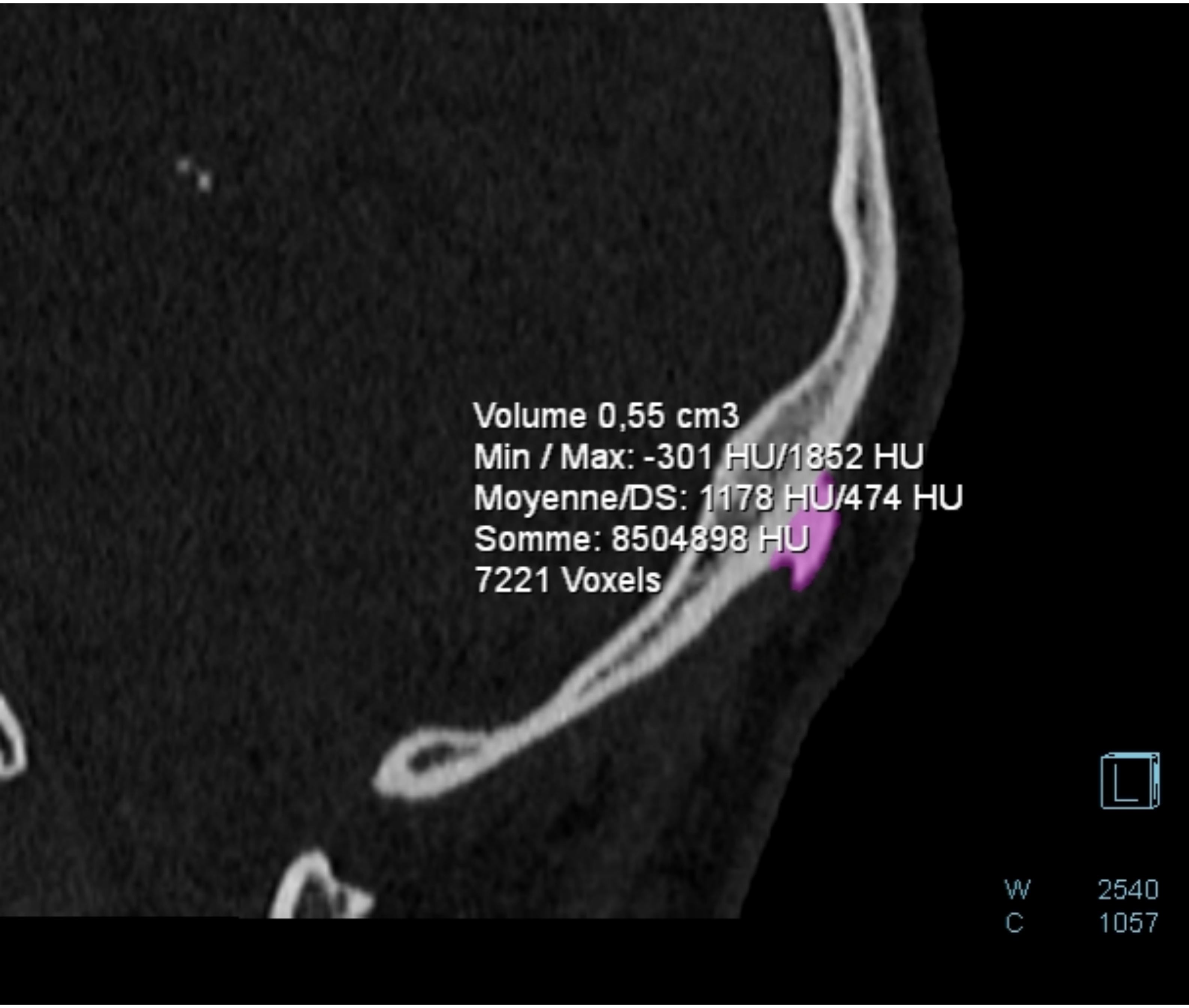

Volume 0,55 cm3
Min / Max: -301 HU/1852 HU
Moyenne/DS: 1178 HU/474 HU
Somme: 8504898 HU
7221 Voxels
W 2540
C 1057

19f10

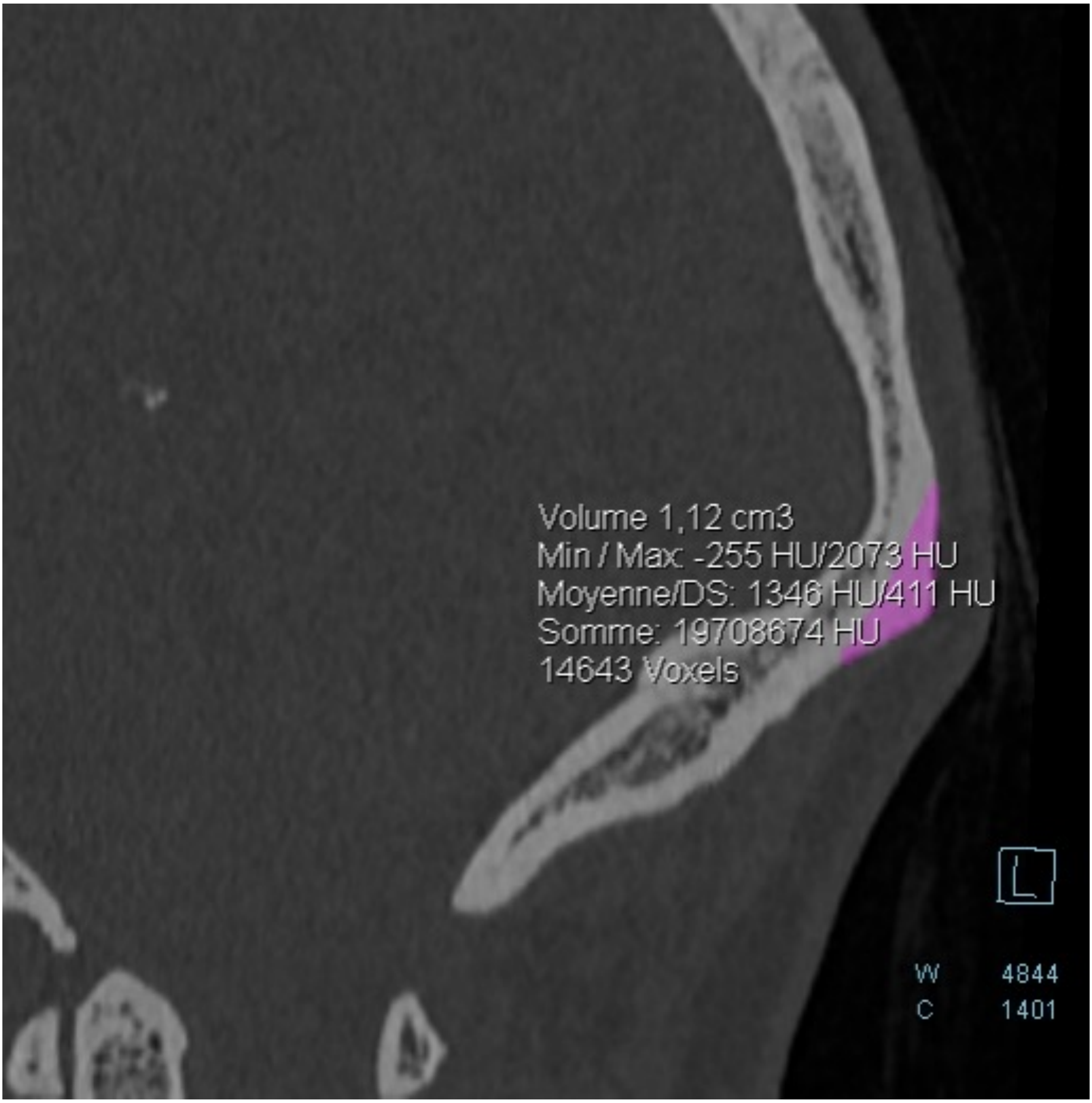

Volume 1,12 cm3
Min / Max: -255 HU/2073 HU
Moyenne/DS: 1346 HU/411 HU
Somme: 19708674 HU
14643 Voxels
L
W 4844
C 1401

19f11

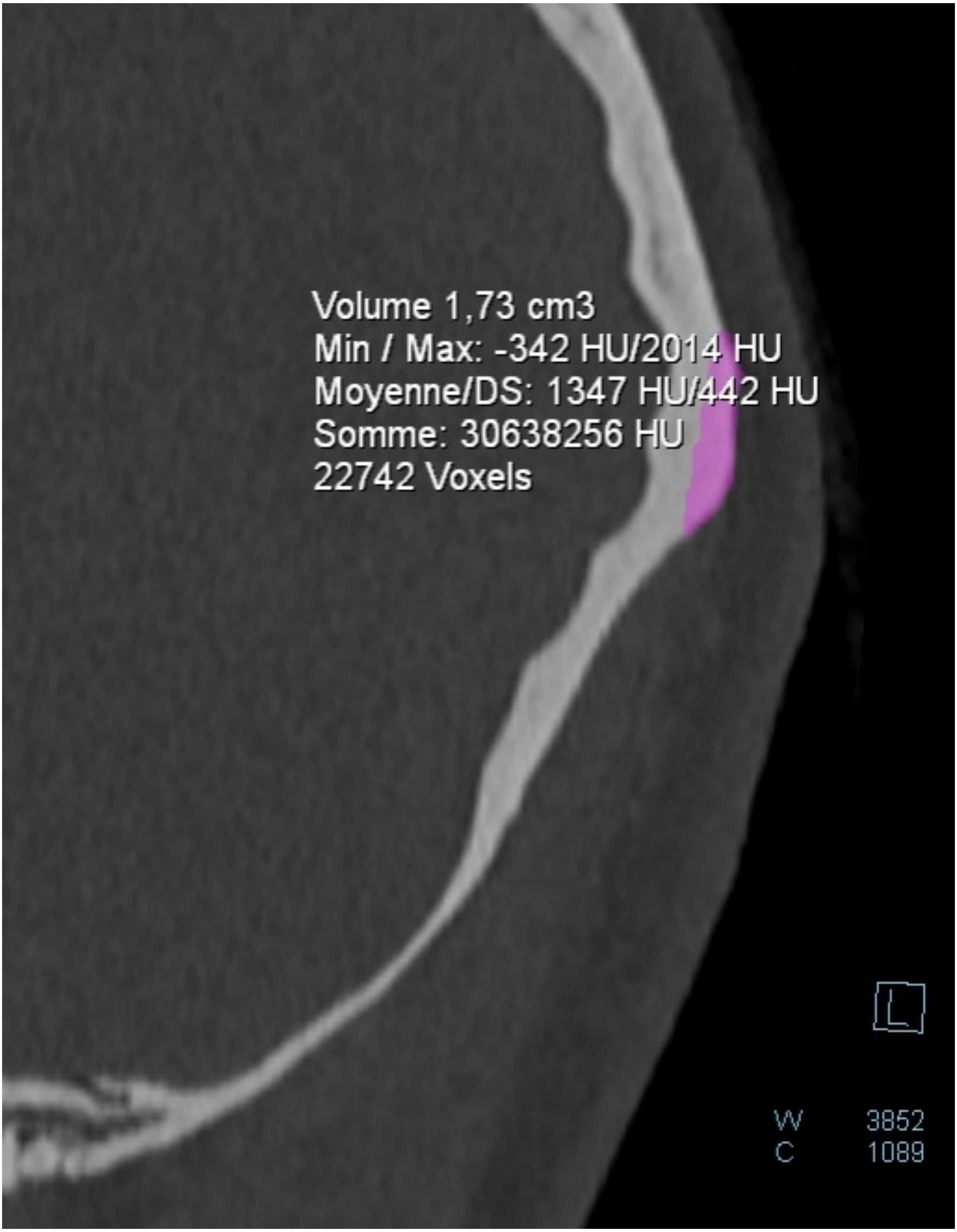
Volume 1,73 cm3
Min / Max: -342 HU/2014 HU
Moyenne/DS: 1347 HU/442 HU
Somme: 30638256 HU
22742 Voxels
L
W 3852
C 1089

19f12

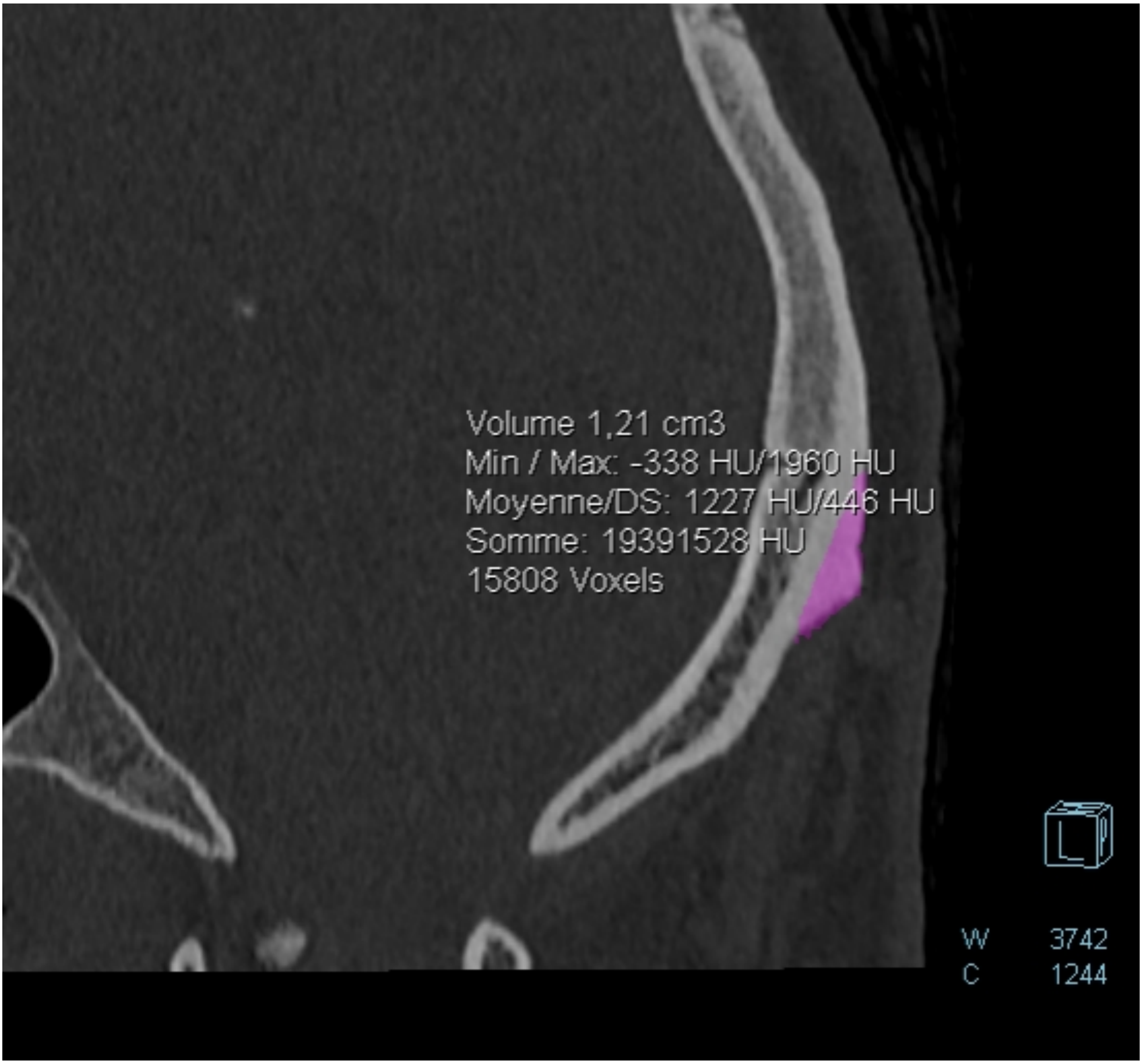
Volume 1,21 cm3
Min / Max: -338 HU/1960 HU
Moyenne/DS: 1227 HU/446 HU
Somme: 19391528 HU
15808 Voxels
L
W 3742
C 1244

19f13

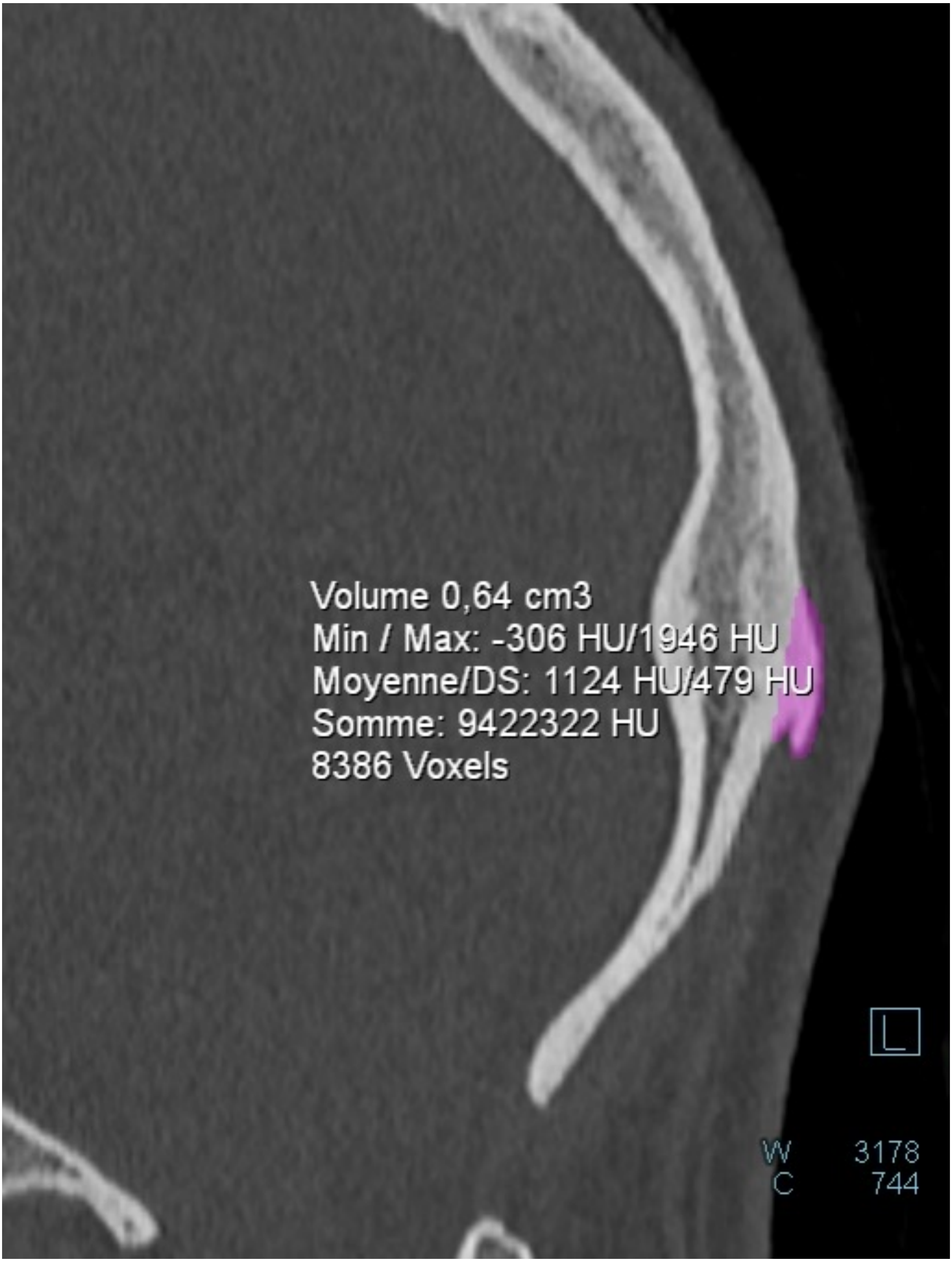
Volume 0,64 cm3
Min / Max: -306 HU/1946 HU
Moyenne/DS: 1124 HU/479 HU
Somme: 9422322 HU
8386 Voxels
L
W 3178
C 744

19f14

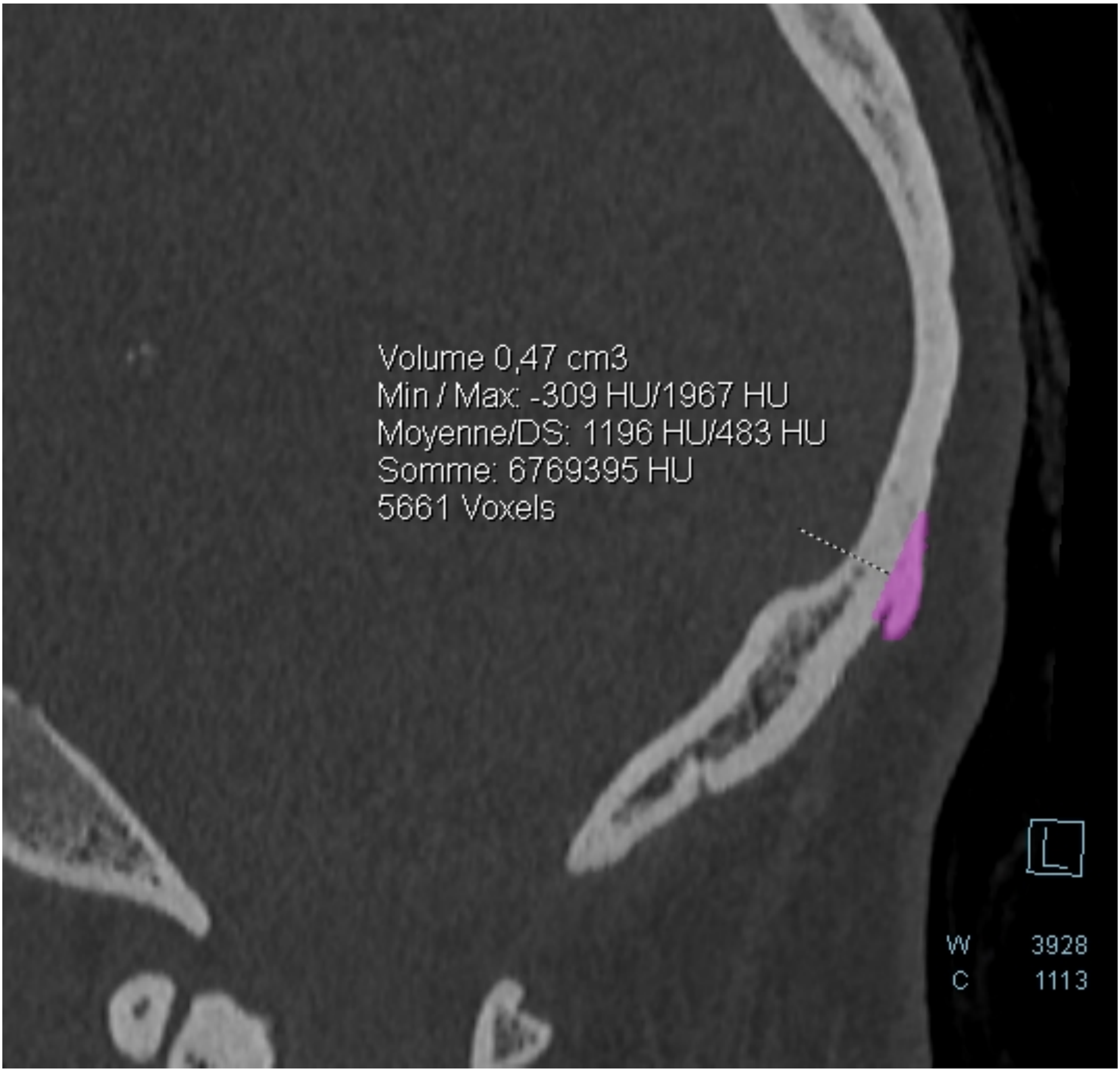
Volume 0,47 cm3
Min / Max: -309 HU/1967 HU
Moyenne/DS: 1196 HU/483 HU
Somme: 6769395 HU
5661 Voxels
L
W 3928
C 1113

19f15

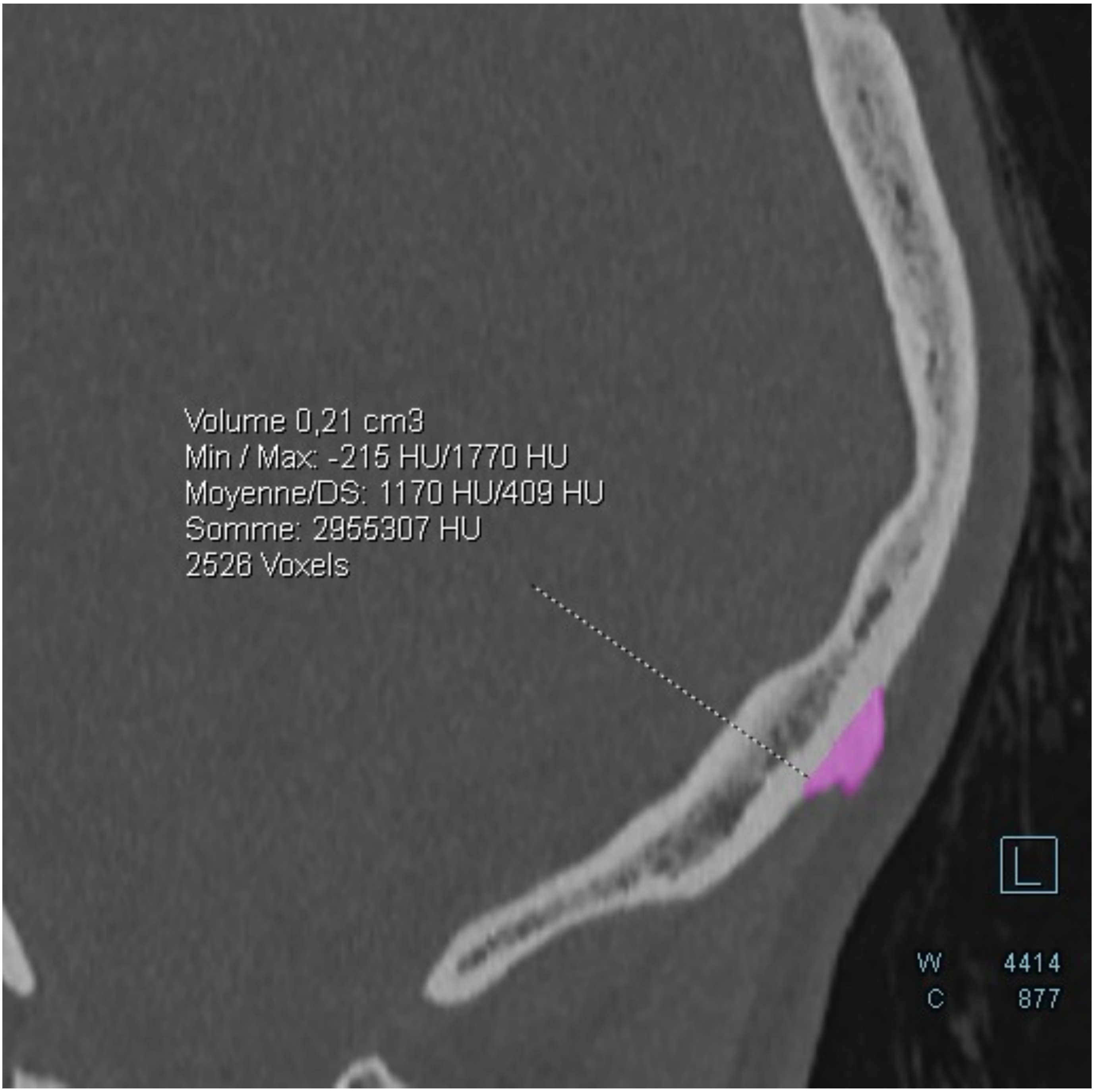
Volume 0,21 cm3
Min / Max: -215 HU/1770 HU
Moyenne/DS: 1170 HU/409 HU
Somme: 2955307 HU
2526 Voxels
L
W 4414
C 877

19f16

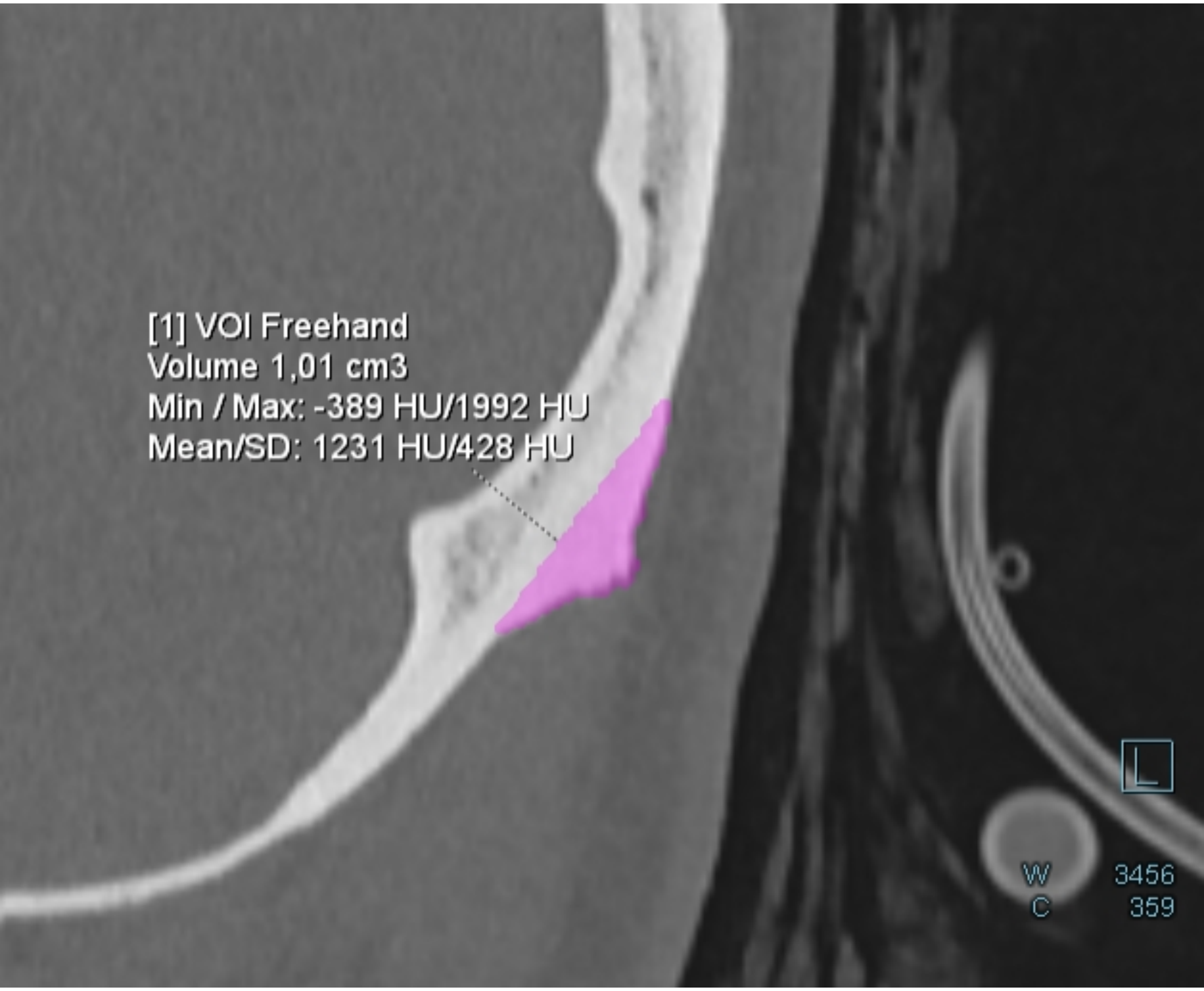
[1] VOI Freehand
Volume 1,01 cm3
Min / Max: -389 HU/1992 HU
Mean/SD: 1231 HU/428 HU
L
W 3456
C 359

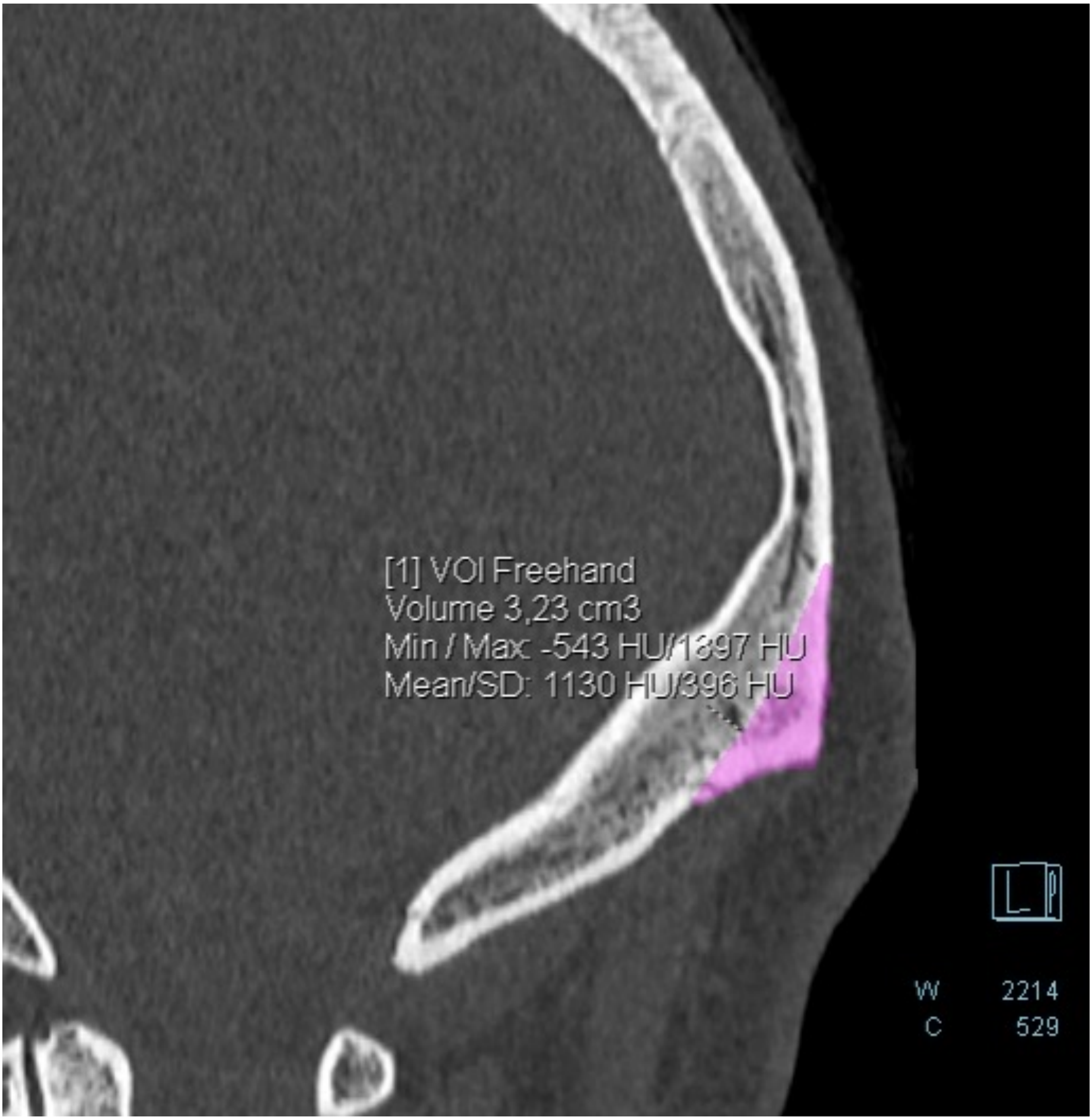
[1] VOI Freehand
Volume 3,23 cm3
Min / Max: -543 HU/1397 HU
Mean/SD: 1130 HU/396 HU
W 2214
C 529

19f18

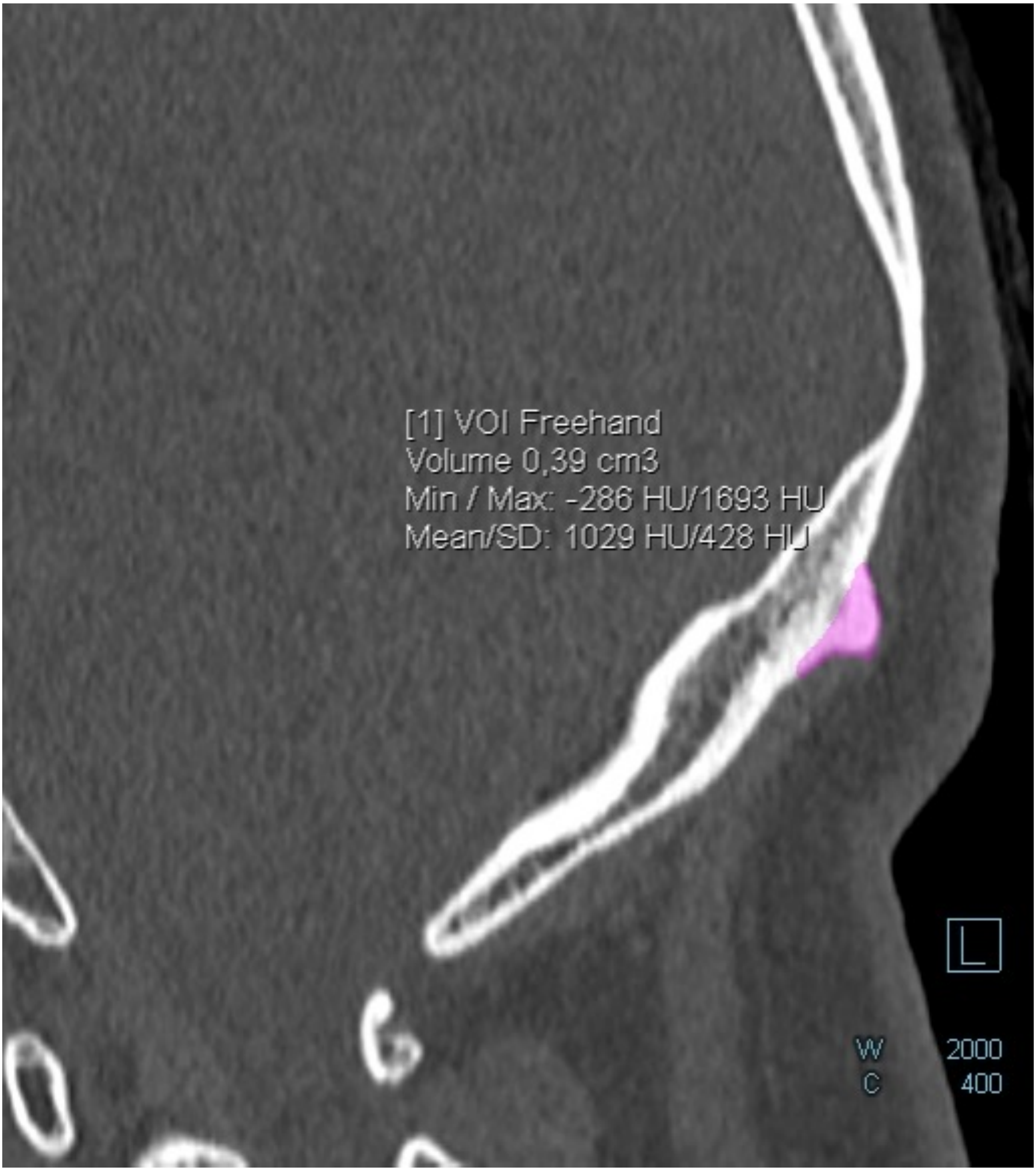
[1] VOI Freehand
Volume 0,39 cm3
Min / Max: -286 HU/1693 HU
Mean/SD: 1029 HU/428 HU
L
W 2000
C 400

19m1

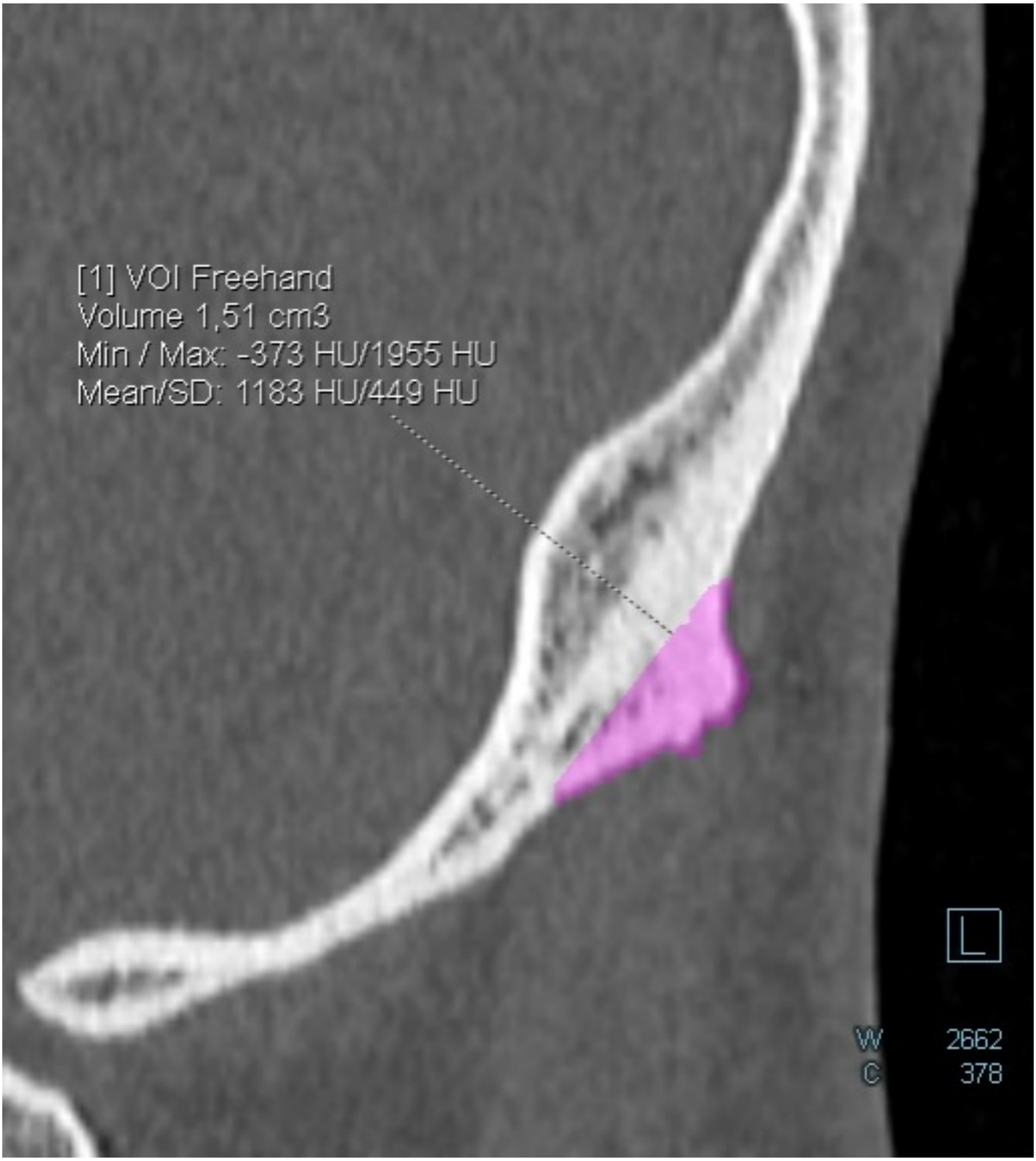
[1] VOI Freehand
Volume 1,51 cm3
Min / Max: -373 HU/1955 HU
Mean/SD: 1183 HU/449 HU
L
W 2662
C 378

19m2

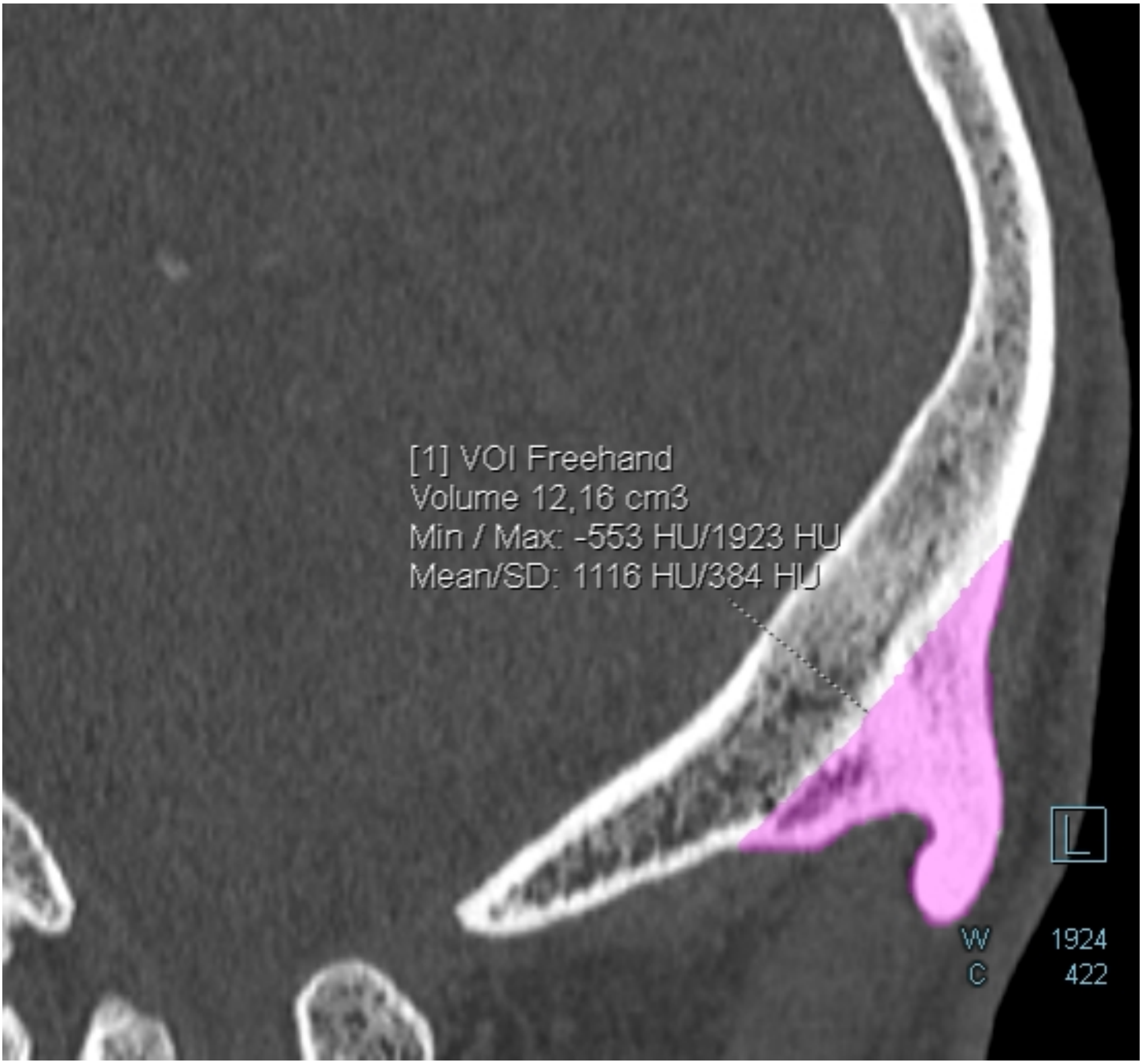
[1] VOI Freehand
Volume 12,16 cm3
Min / Max: -553 HU/1923 HU
Mean/SD: 1116 HU/384 HU
L
W 1924
C 422

19m3

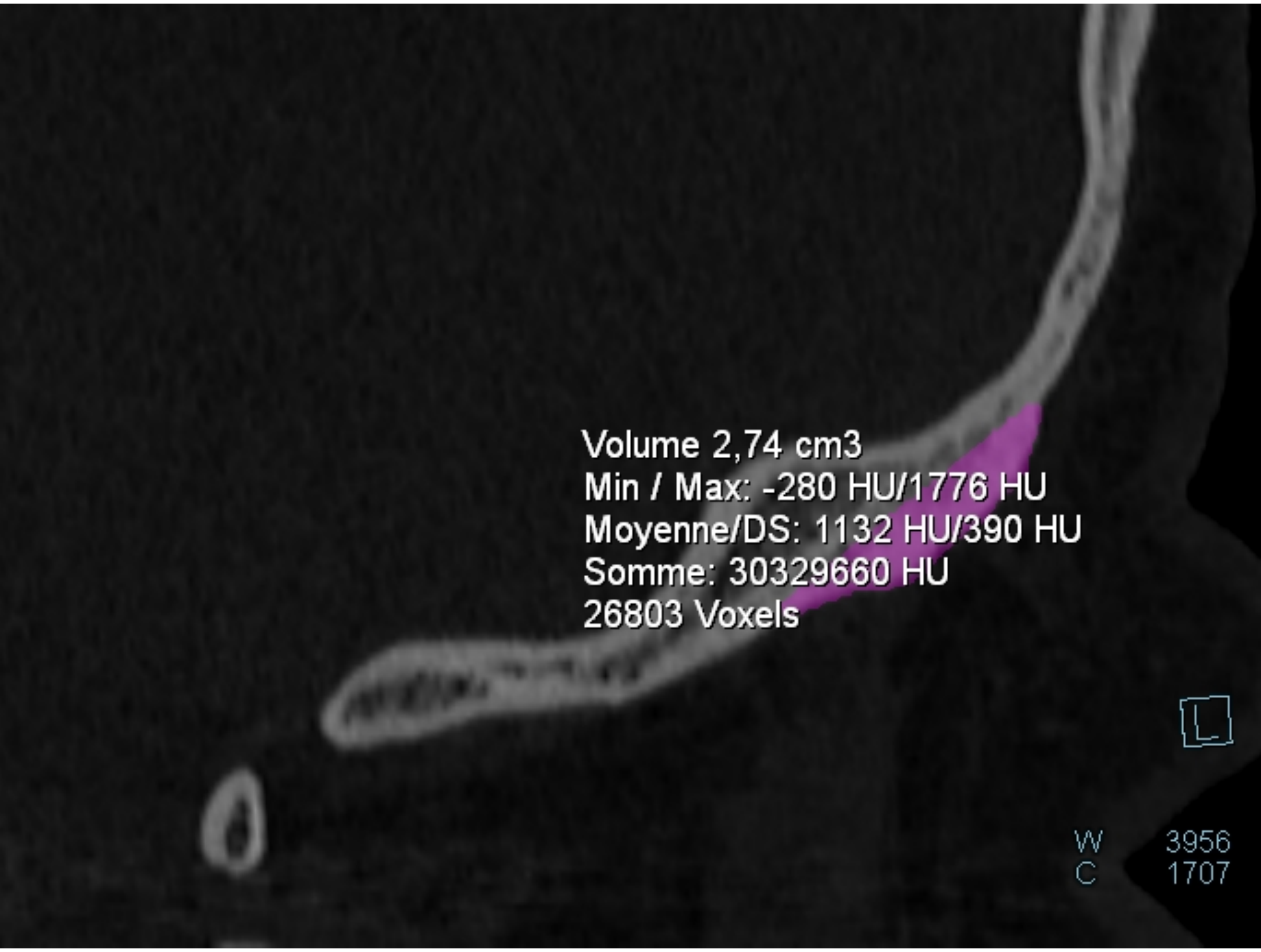

Volume 2,74 cm3
Min / Max: -280 HU/1776 HU
Moyenne/DS: 1132 HU/390 HU
Somme: 30329660 HU
26803 Voxels
W 3956
C 1707

19m4

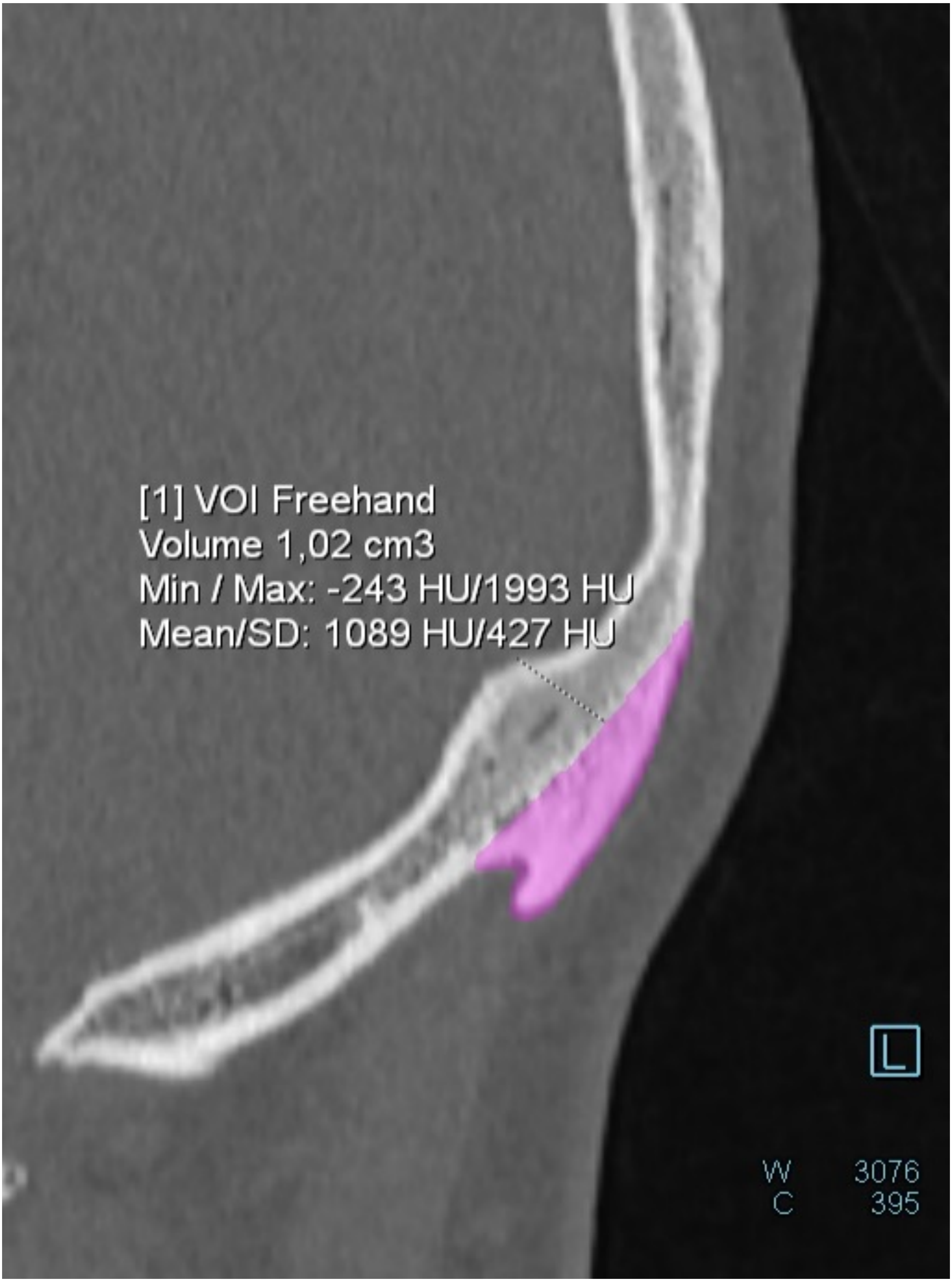
[1] VOI Freehand
Volume 1,02 cm3
Min / Max: -243 HU/1993 HU
Mean/SD: 1089 HU/427 HU
L
W 3076
C 395

19m5

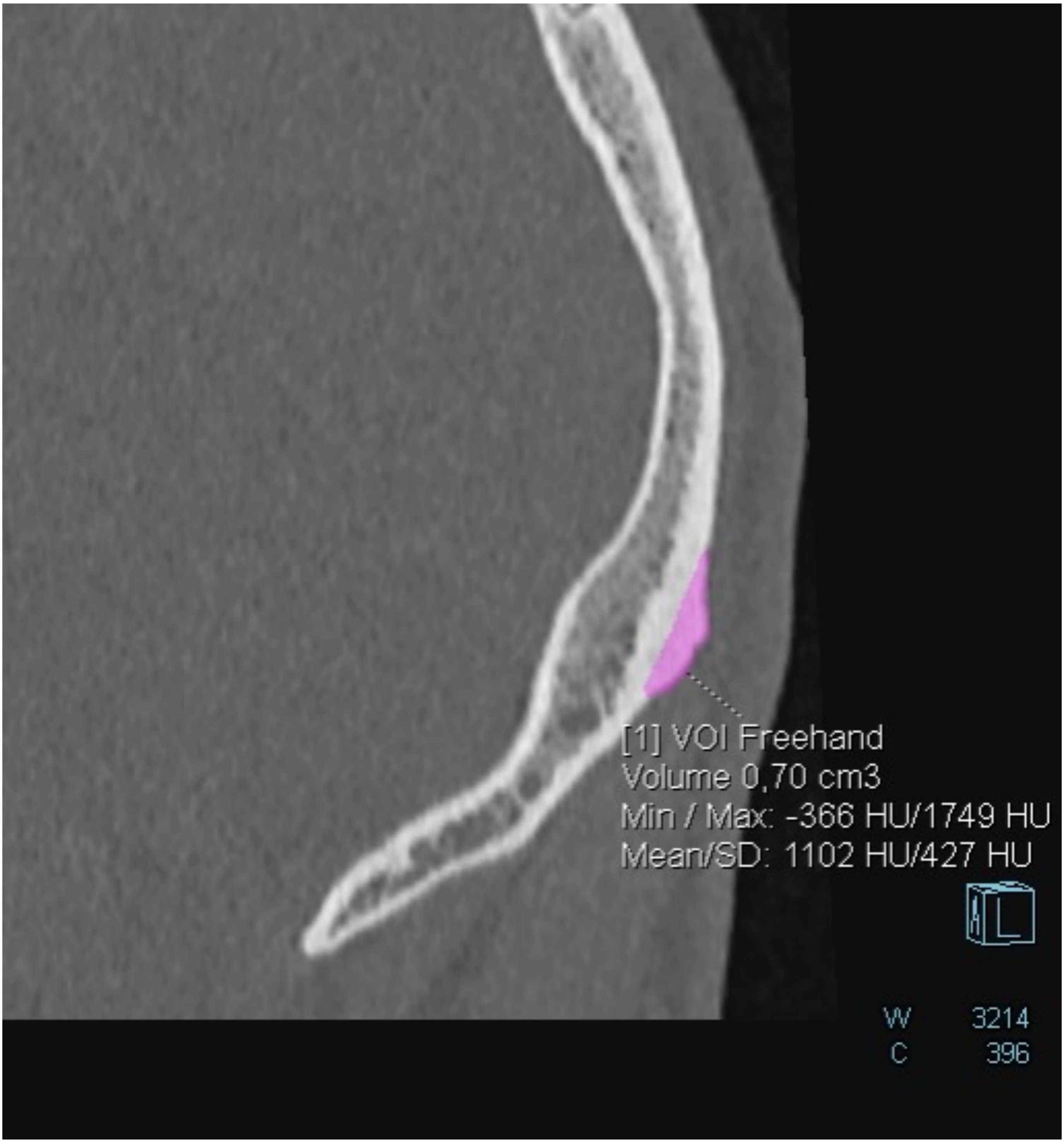
[1] VOI Freehand
Volume 0,70 cm3
Min / Max: -366 HU/1749 HU
Mean/SD: 1102 HU/427 HU
W 3214
C 396

19m6

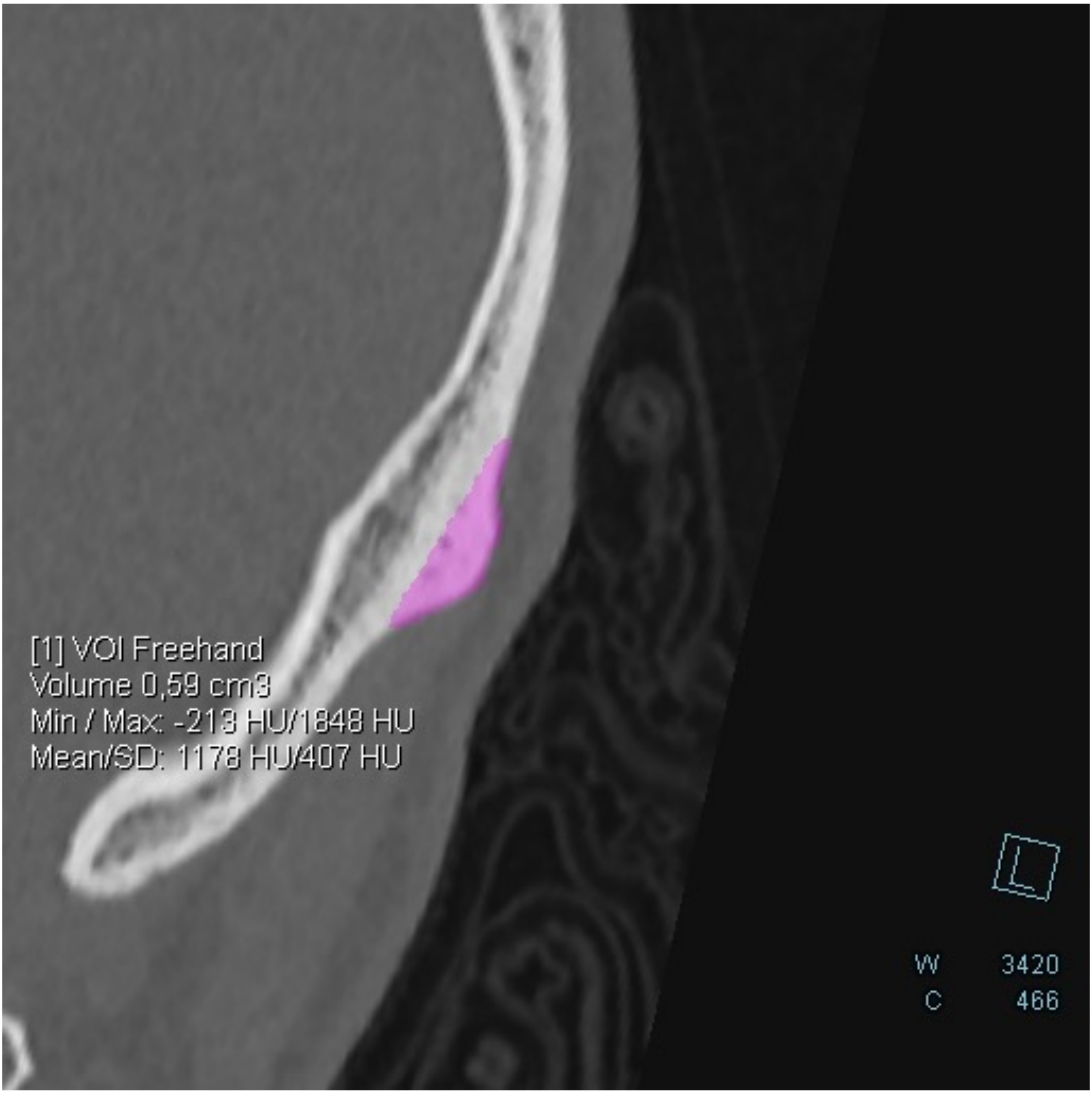
[1] VOI Freehand
Volume 0,59 cm3
Min / Max: -213 HU/1848 HU
Mean/SD: 1178 HU/407 HU
W 3420
C 466

19m7

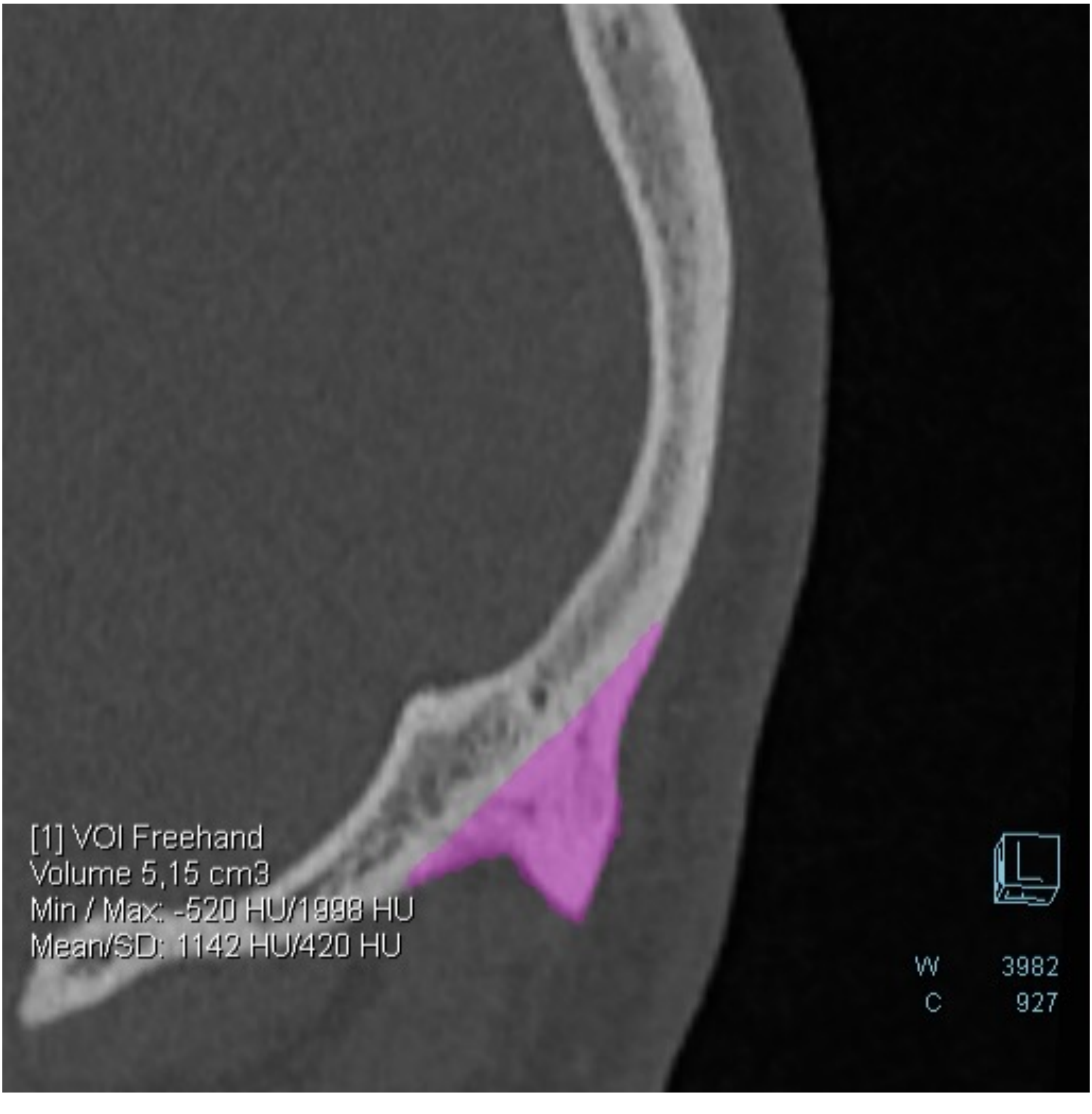
[1] VOI Freehand
Volume 5,15 cm3
Min / Max: -520 HU/1998 HU
Mean/SD: 1142 HU/420 HU
W 3982
C 927

19m8

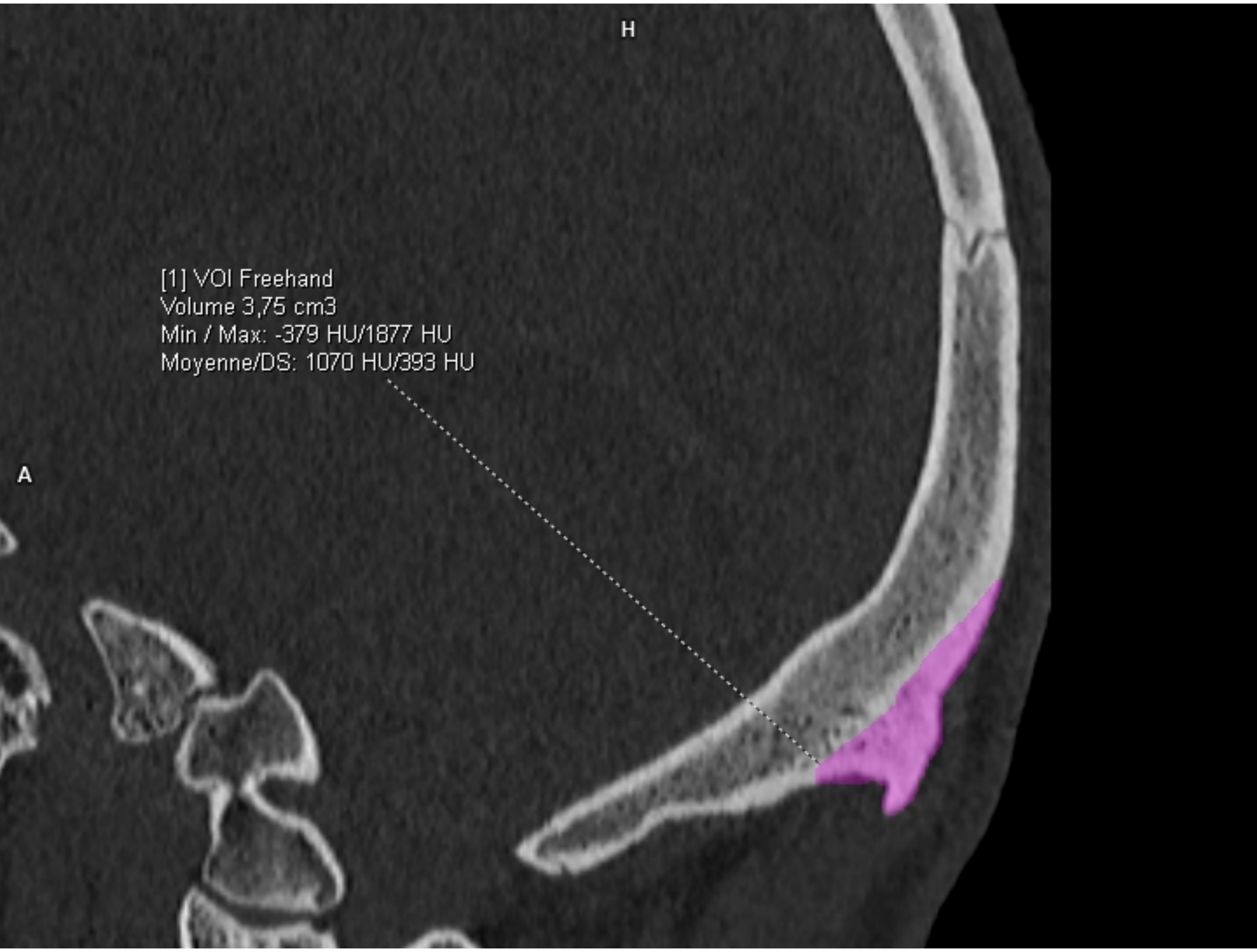
H
A
[1] VOI Freehand
Volume 3,75 cm3
Min / Max: -379 HU/1877 HU
Moyenne/DS: 1070 HU/393 HU

19m9

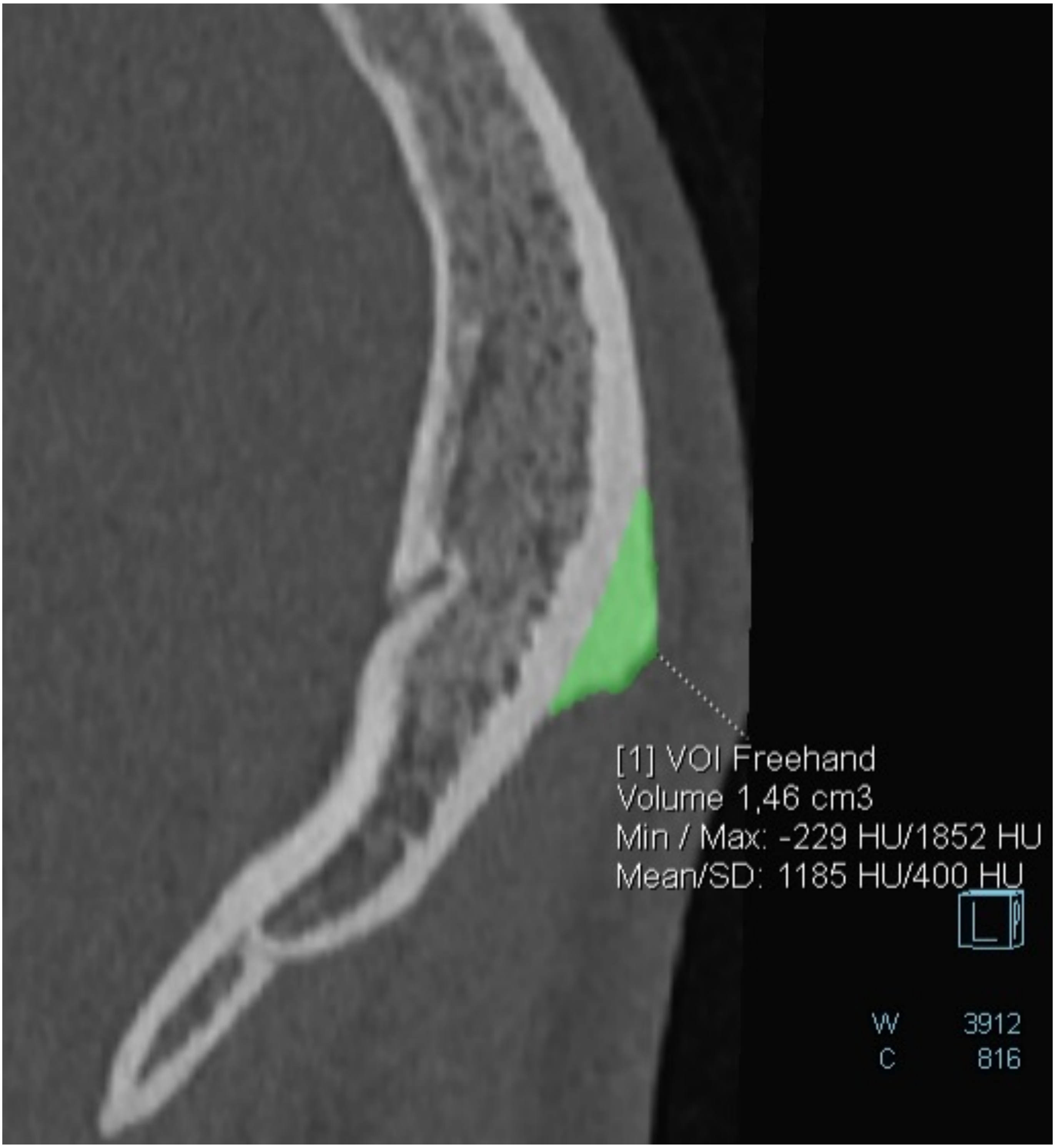
[1] VOI Freehand
Volume 1,46 cm3
Min / Max: -229 HU/1852 HU
Mean/SD: 1185 HU/400 HU
L
W 3912
C 816

19m10

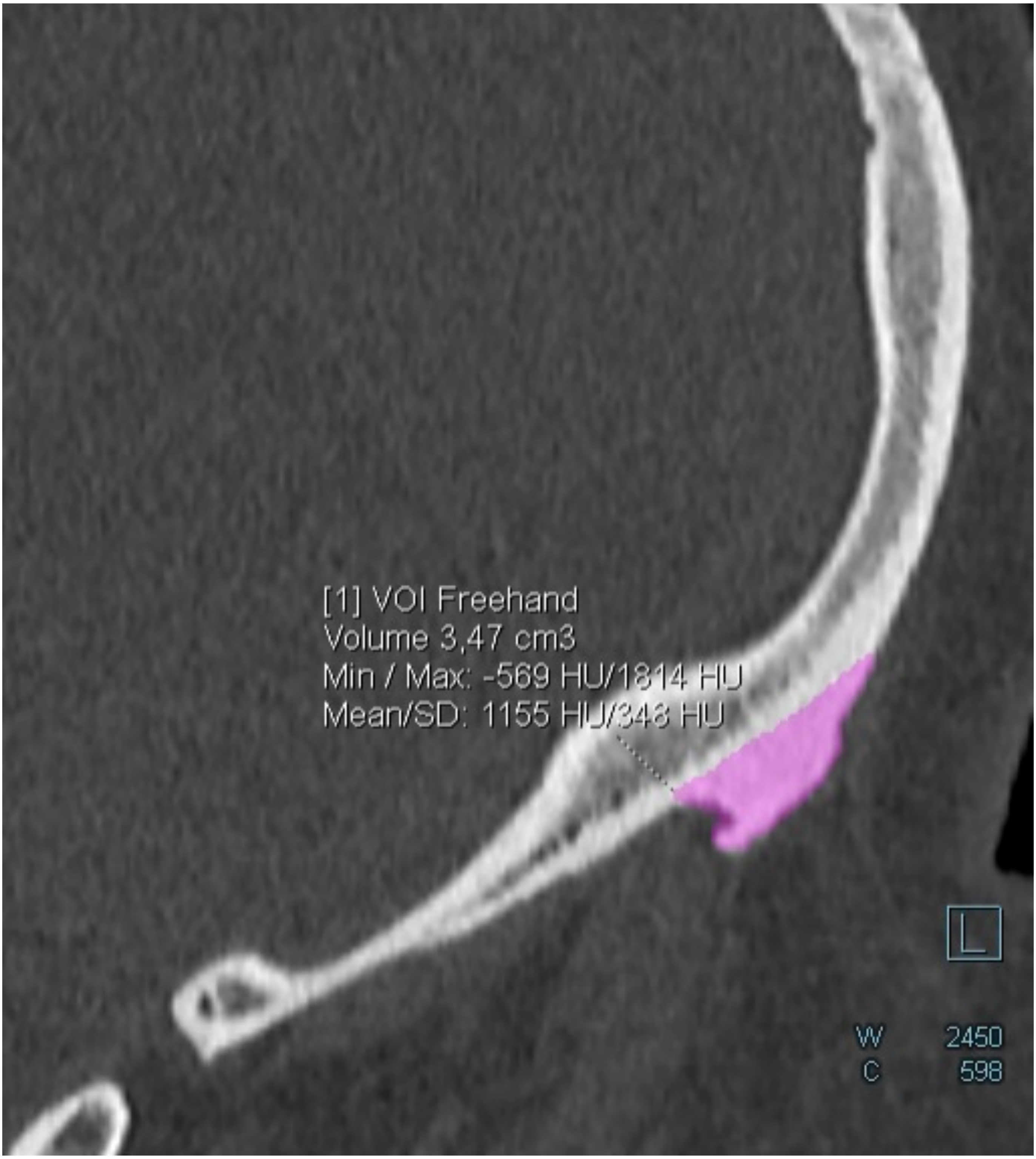
[1] VOI Freehand
Volume 3,47 cm3
Min / Max: -569 HU/1814 HU
Mean/SD: 1155 HU/343 HU
L
W 2450
C 598

19m11

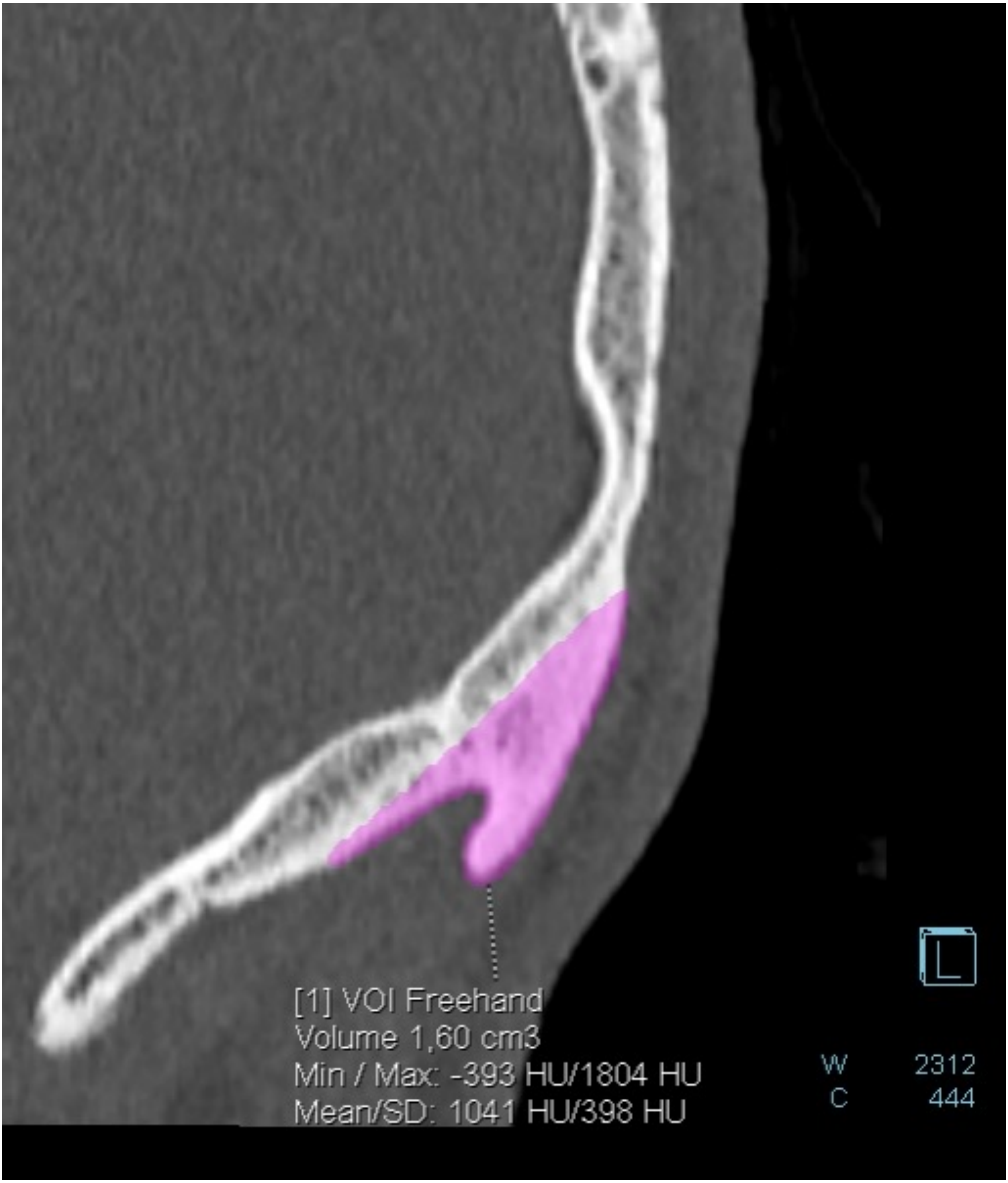
[1] VOI Freehand
Volume 1,60 cm3
Min / Max: -393 HU/1804 HU
Mean/SD: 1041 HU/398 HU
L
W 2312
C 444

19m12

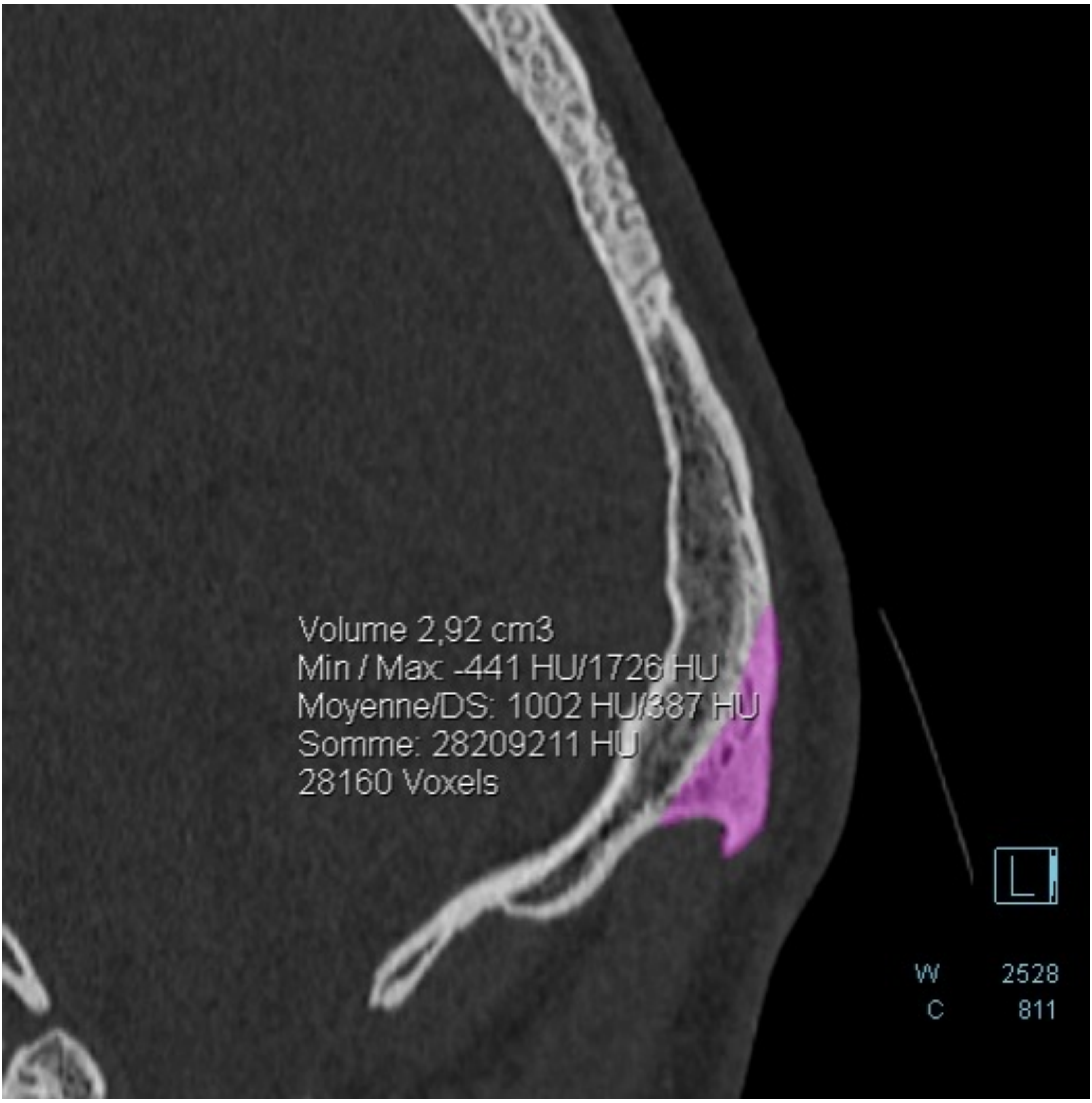
Volume 2,92 cm3
Min / Max: -441 HU/1726 HU
Moyenne/DS: 1002 HU/387 HU
Somme: 28209211 HU
28160 Voxels
L
W 2528
C 811

19m13

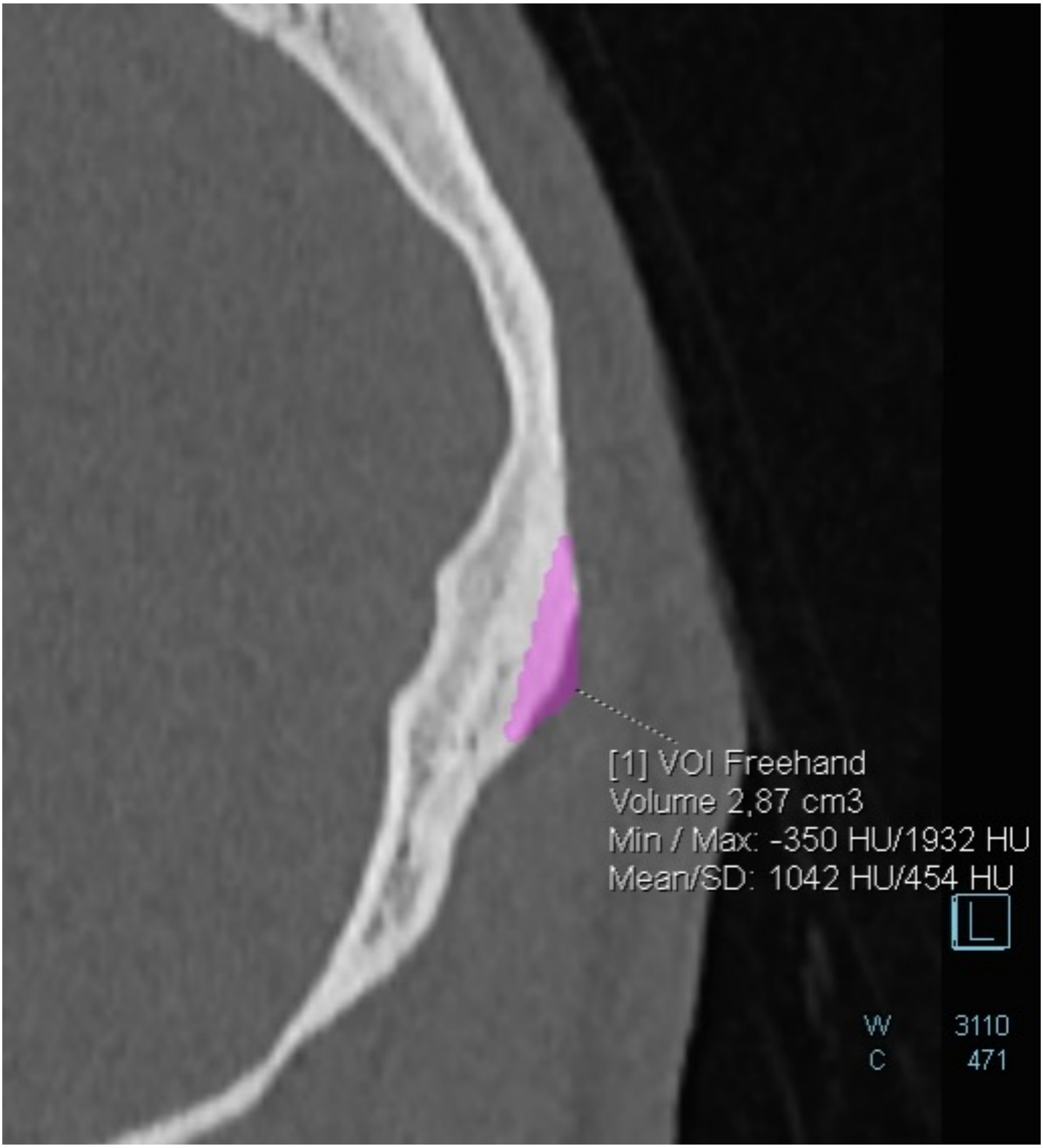
[1] VOI Freehand
Volume 2,87 cm3
Min / Max: -350 HU/1932 HU
Mean/SD: 1042 HU/454 HU
L
W 3110
C 471

19m14

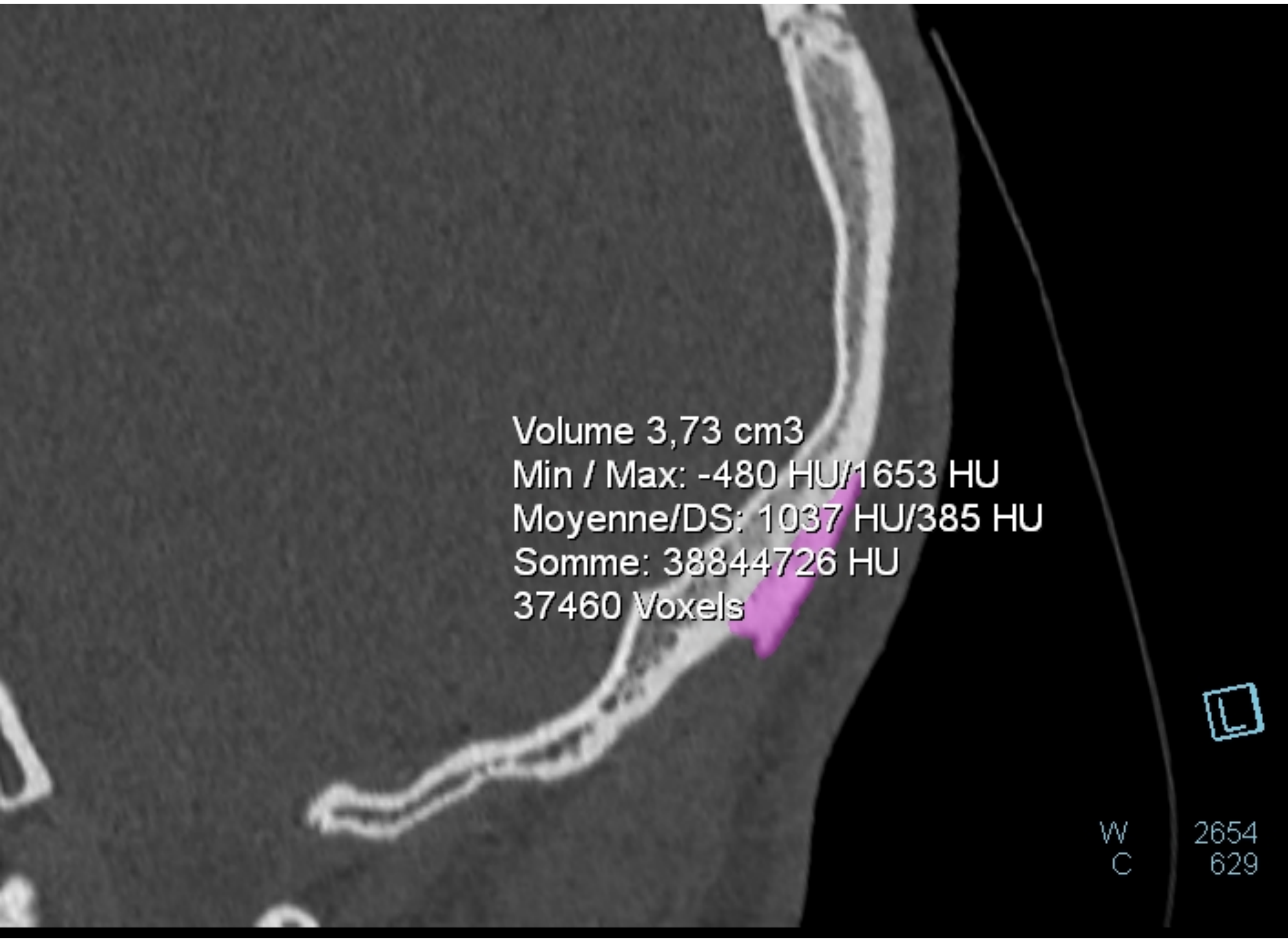
Volume 3,73 cm3
Min / Max: -480 HU/1653 HU
Moyenne/DS: 1037 HU/385 HU
Somme: 38844726 HU
37460 Voxels
W 2654
C 629

19m15

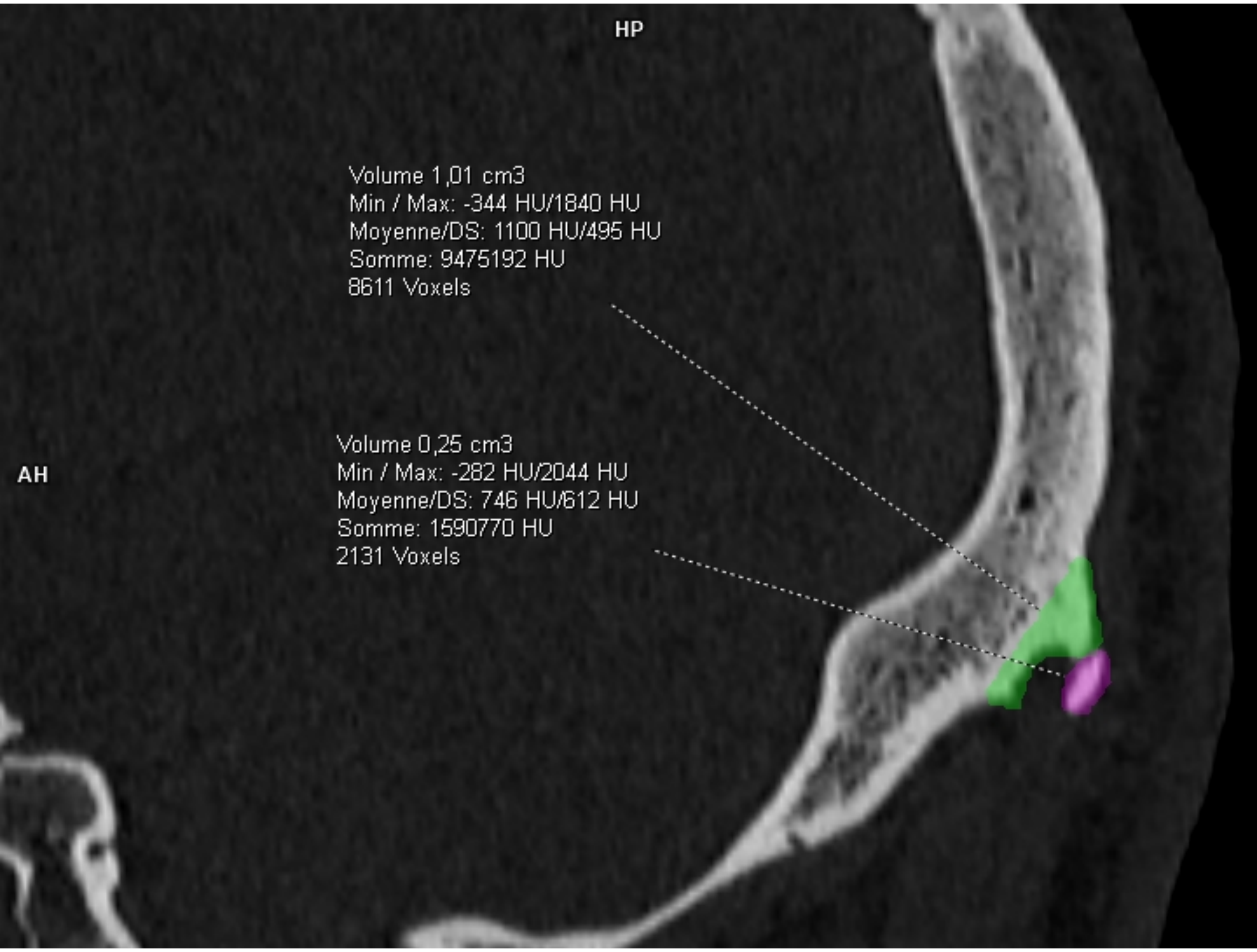
HP
AH
Volume 1,01 cm3
Min / Max: -344 HU/1840 HU
Moyenne/DS: 1100 HU/495 HU
Somme: 9475192 HU
8611 Voxels
Volume 0,25 cm3
Min / Max: -282 HU/2044 HU
Moyenne/DS: 746 HU/612 HU
Somme: 1590770 HU
2131 Voxels

19m16

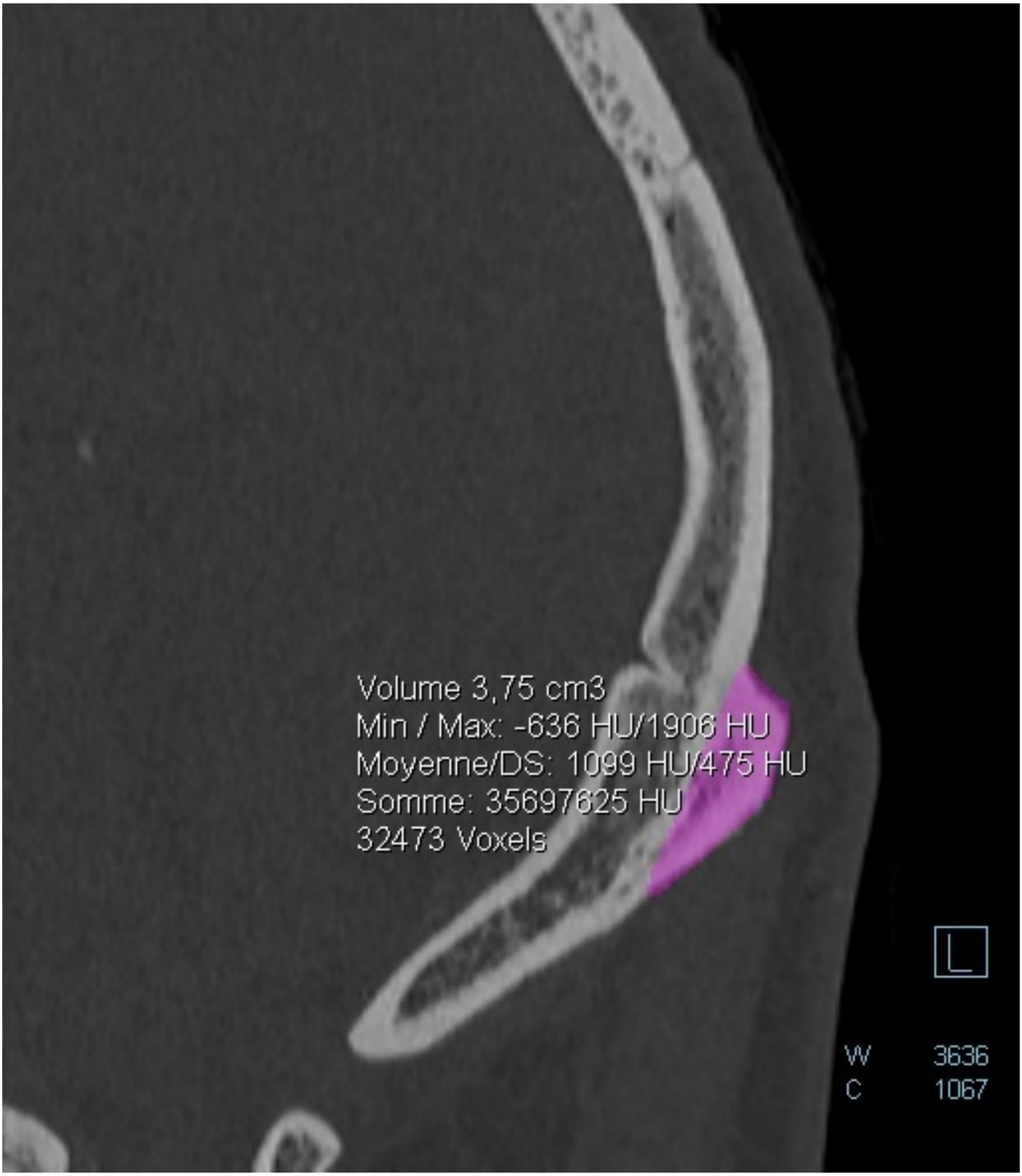
Volume 3,75 cm3
Min / Max: -636 HU/1906 HU
Moyenne/DS: 1099 HU/475 HU
Somme: 35697625 HU
32473 Voxels
L
W 3636
C 1067

19m17

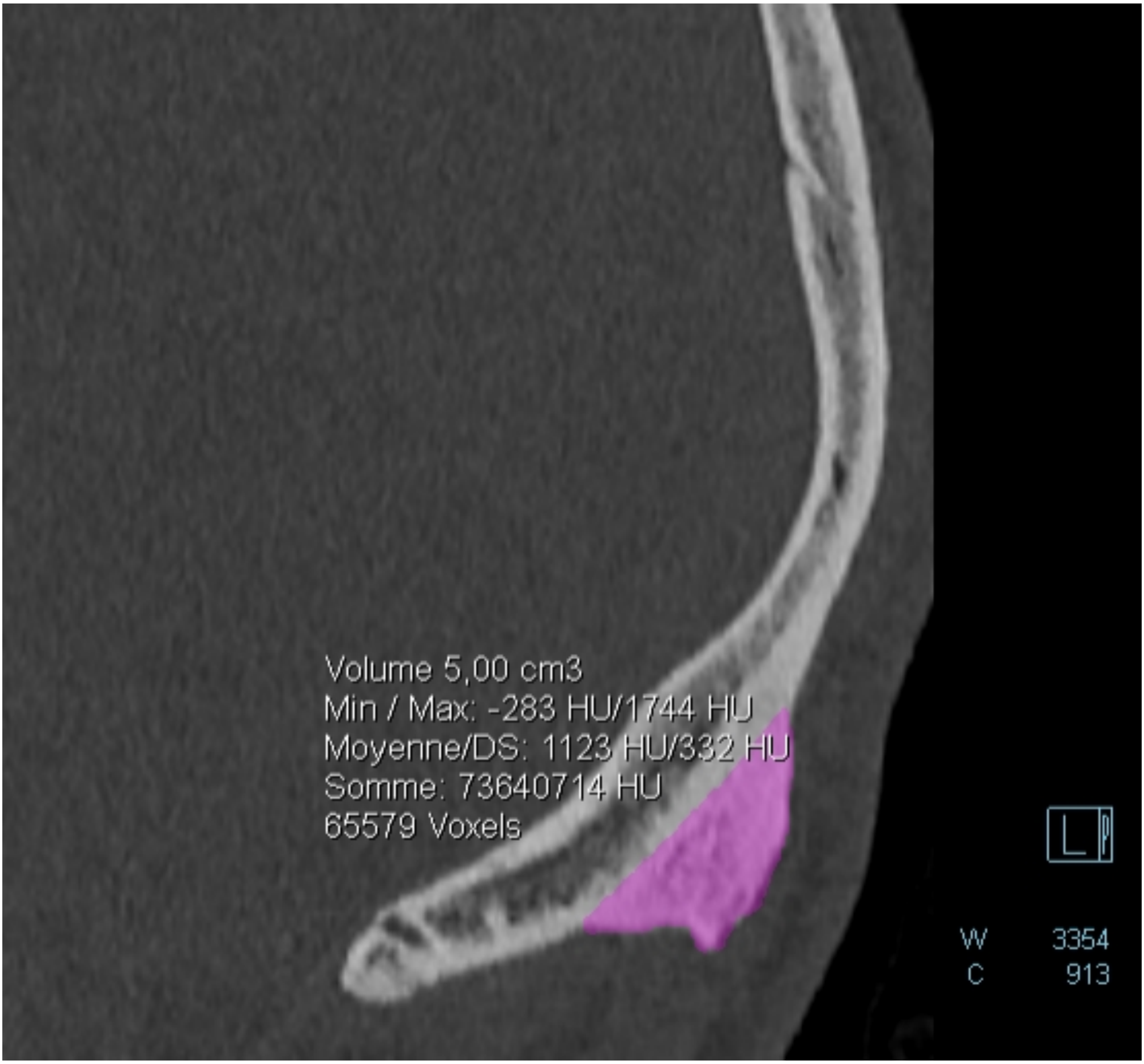
Volume 5,00 cm3
Min / Max: -283 HU/1744 HU
Moyenne/DS: 1123 HU/332 HU
Somme: 73640714 HU
65579 Voxels
L
W 3354
C 913

19m18

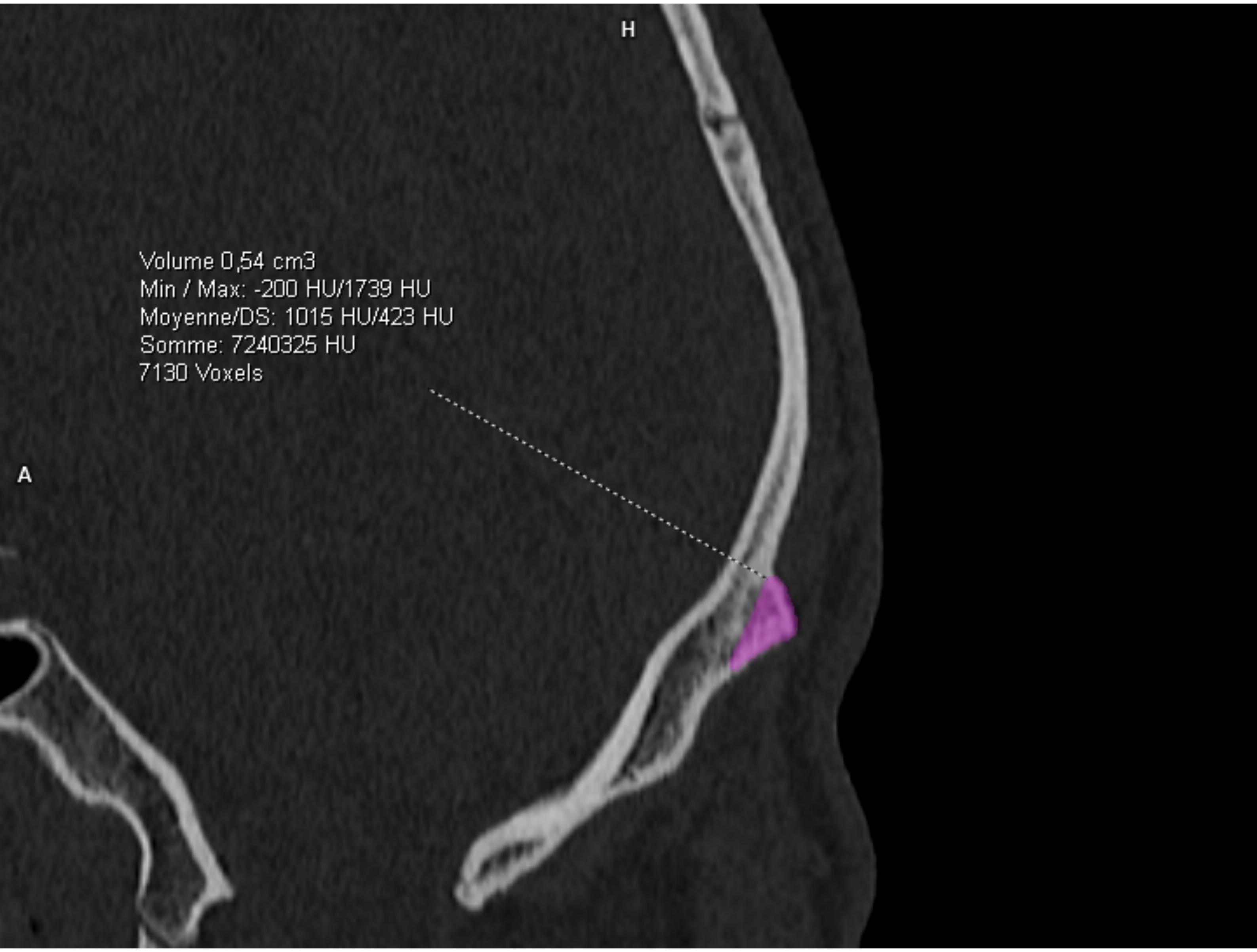
H
Volume 0,54 cm3
Min / Max: -200 HU/1739 HU
Moyenne/DS: 1015 HU/423 HU
Somme: 7240325 HU
7130 Voxels
A

19m19

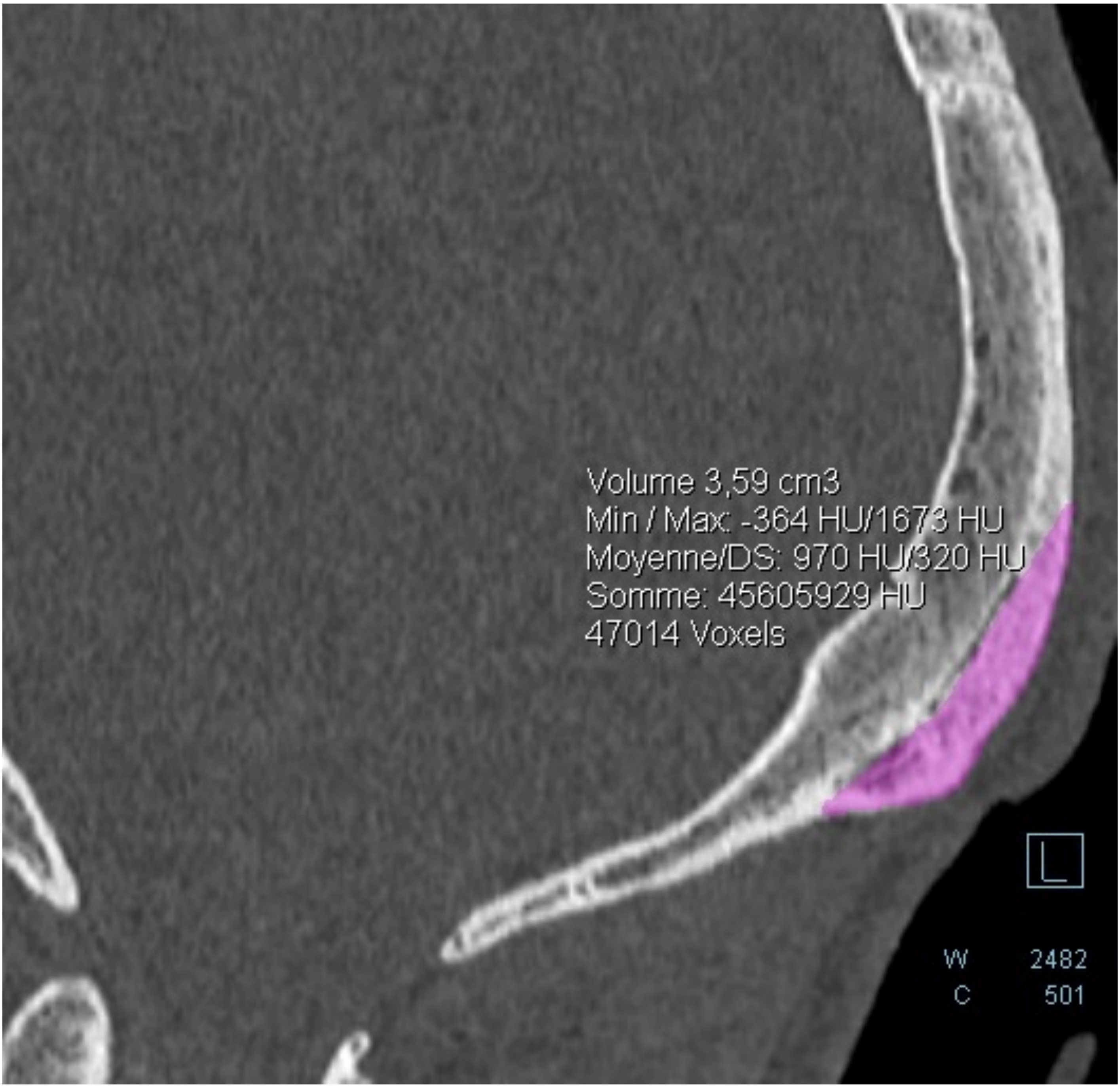
Volume 3,59 cm3
Min / Max: -364 HU/1673 HU
Moyenne/DS: 970 HU/320 HU
Somme: 45605929 HU
47014 Voxels
L
W 2482
C 501

19m20

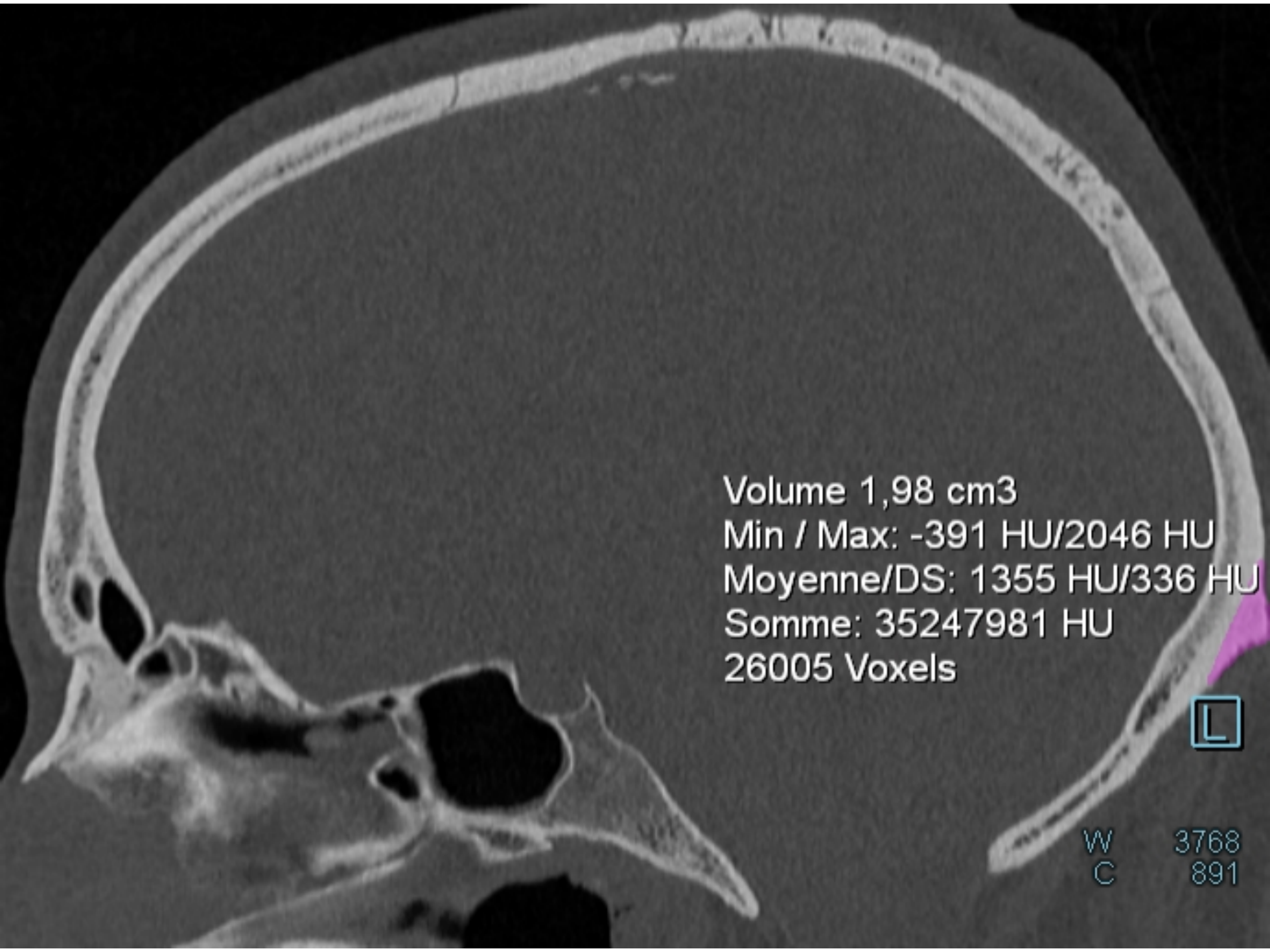
Volume 1,98 cm3
Min / Max: -391 HU/2046 HU
Moyenne/DS: 1355 HU/336 HU
Somme: 35247981 HU
26005 Voxels
L
W 3768
C 891

19m21

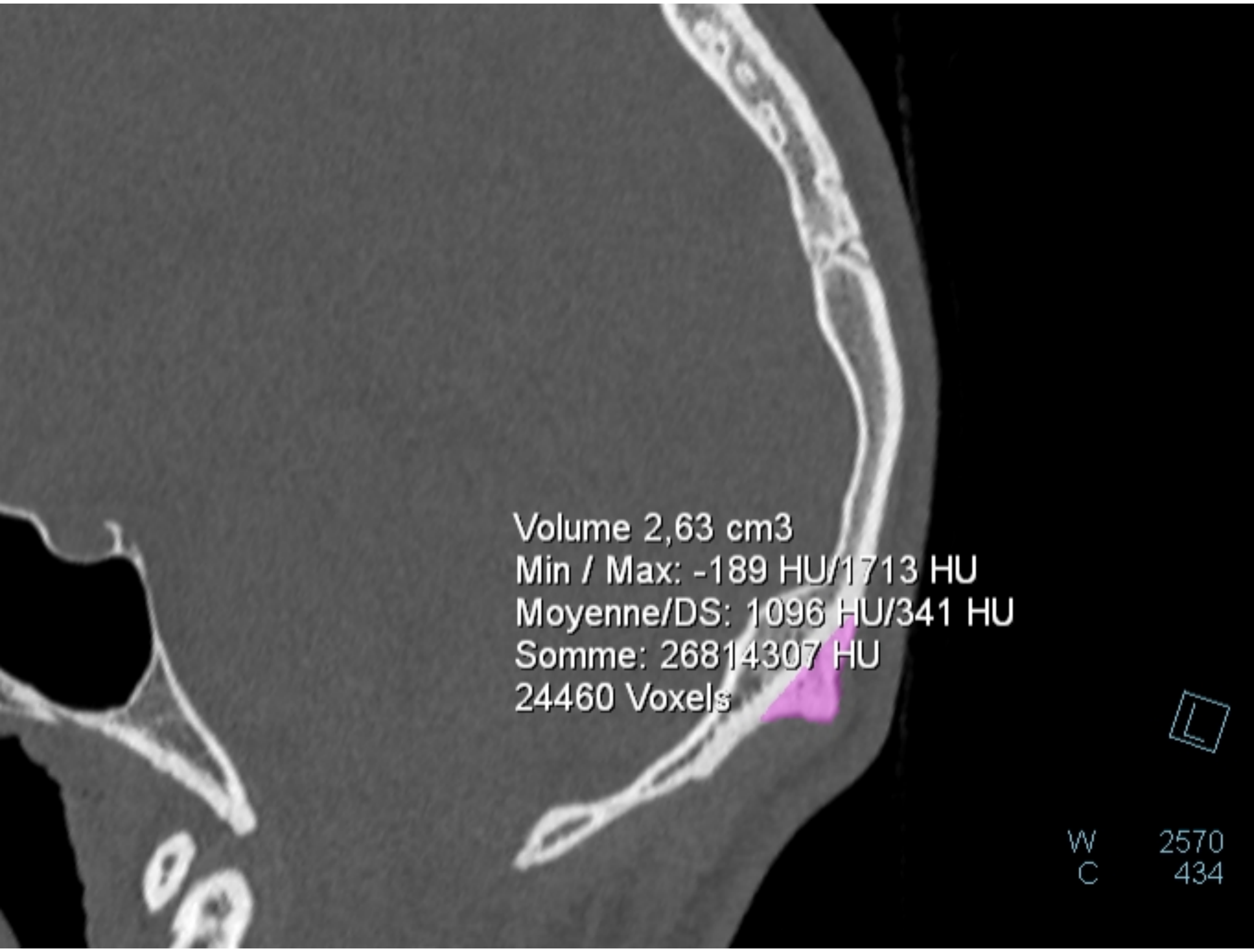
Volume 2,63 cm3
Min / Max: -189 HU/1713 HU
Moyenne/DS: 1096 HU/341 HU
Somme: 26814307 HU
24460 Voxels
W 2570
C 434

19m22

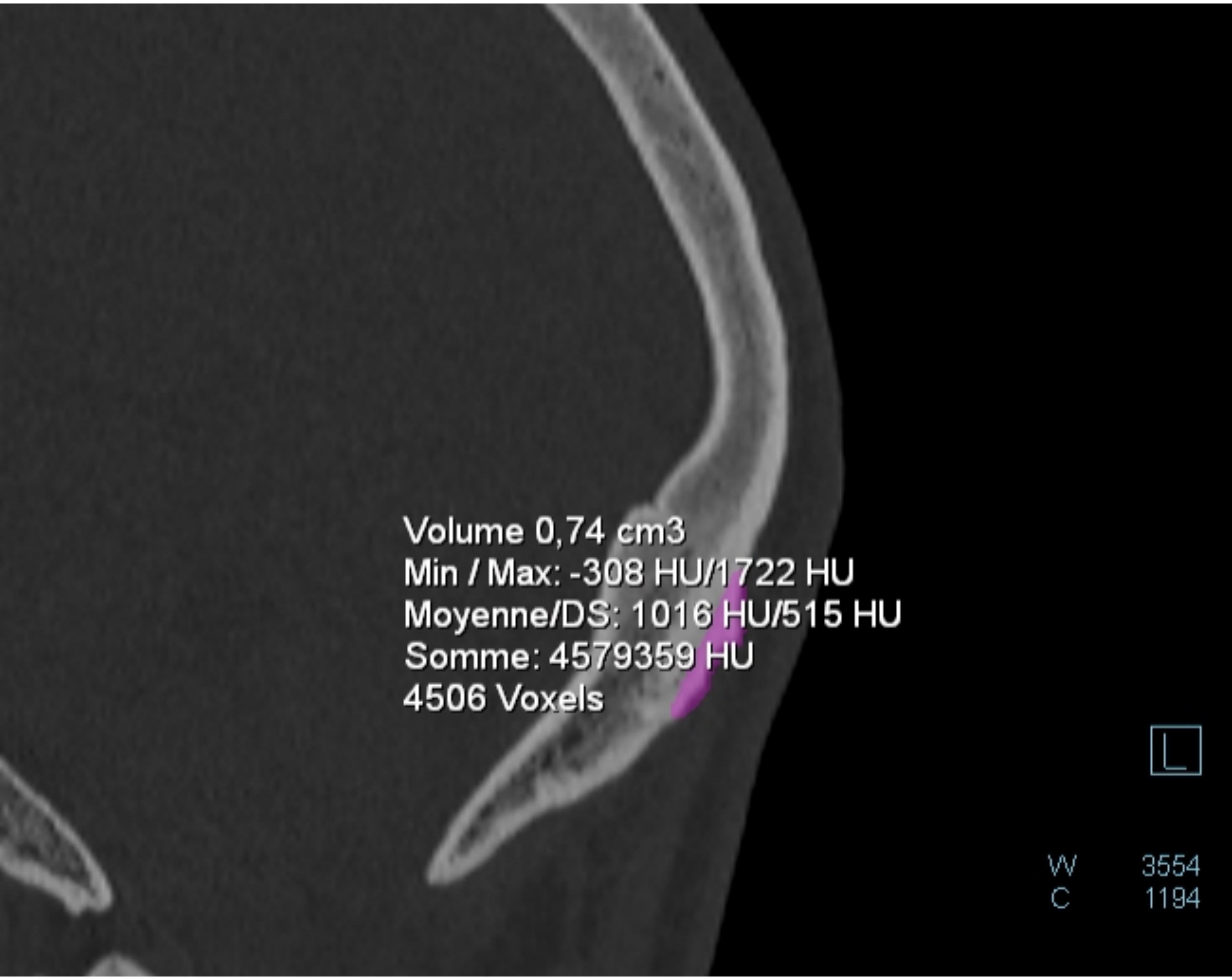
Volume 0,74 cm3
Min / Max: -308 HU/1722 HU
Moyenne/DS: 1016 HU/515 HU
Somme: 4579359 HU
4506 Voxels
L
W 3554
C 1194

19m23

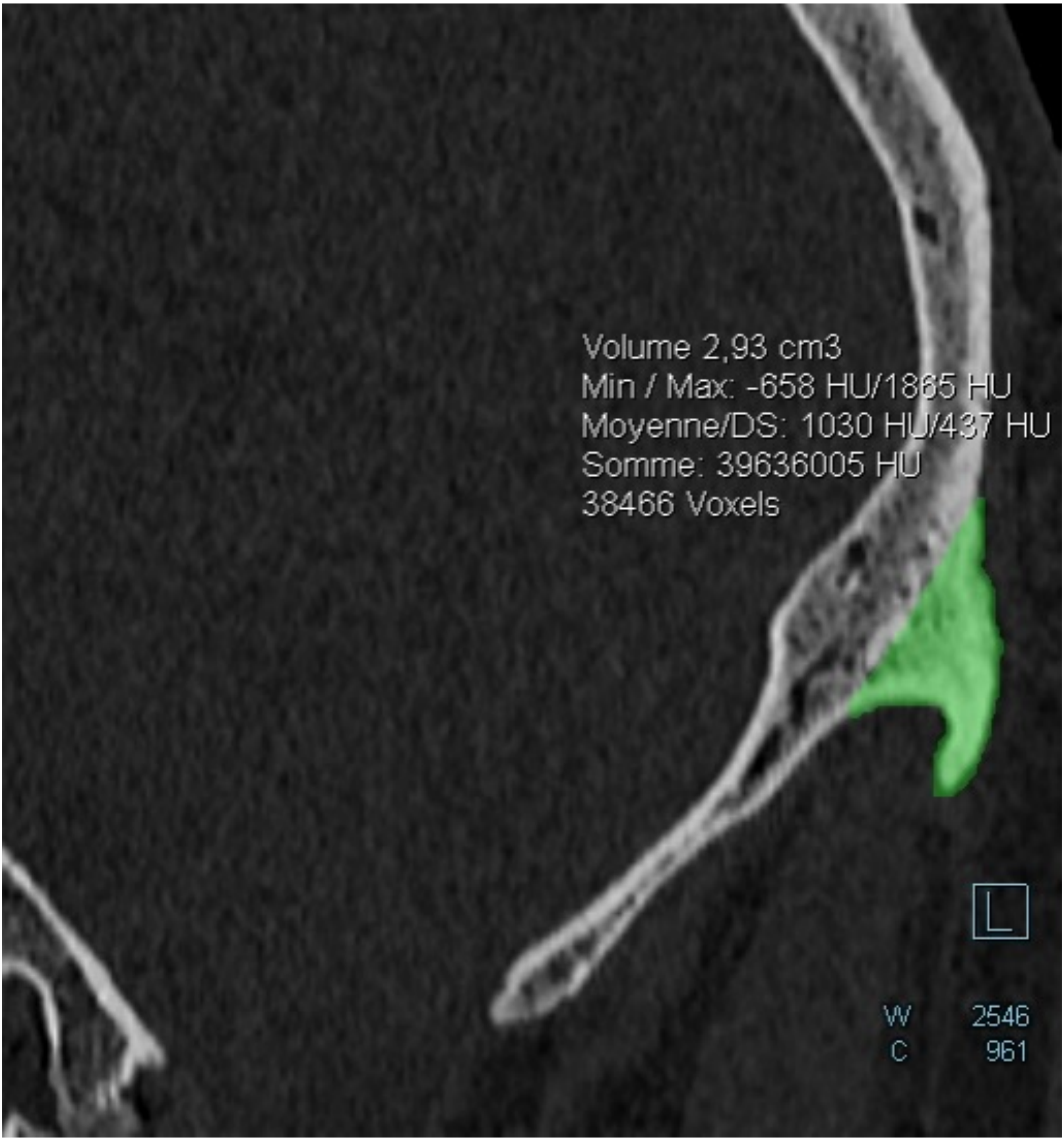
Volume 2,93 cm3
Min / Max: -658 HU/1865 HU
Moyenne/DS: 1030 HU/437 HU
Somme: 39636005 HU
38466 Voxels
L
W 2546
C 961

19m24

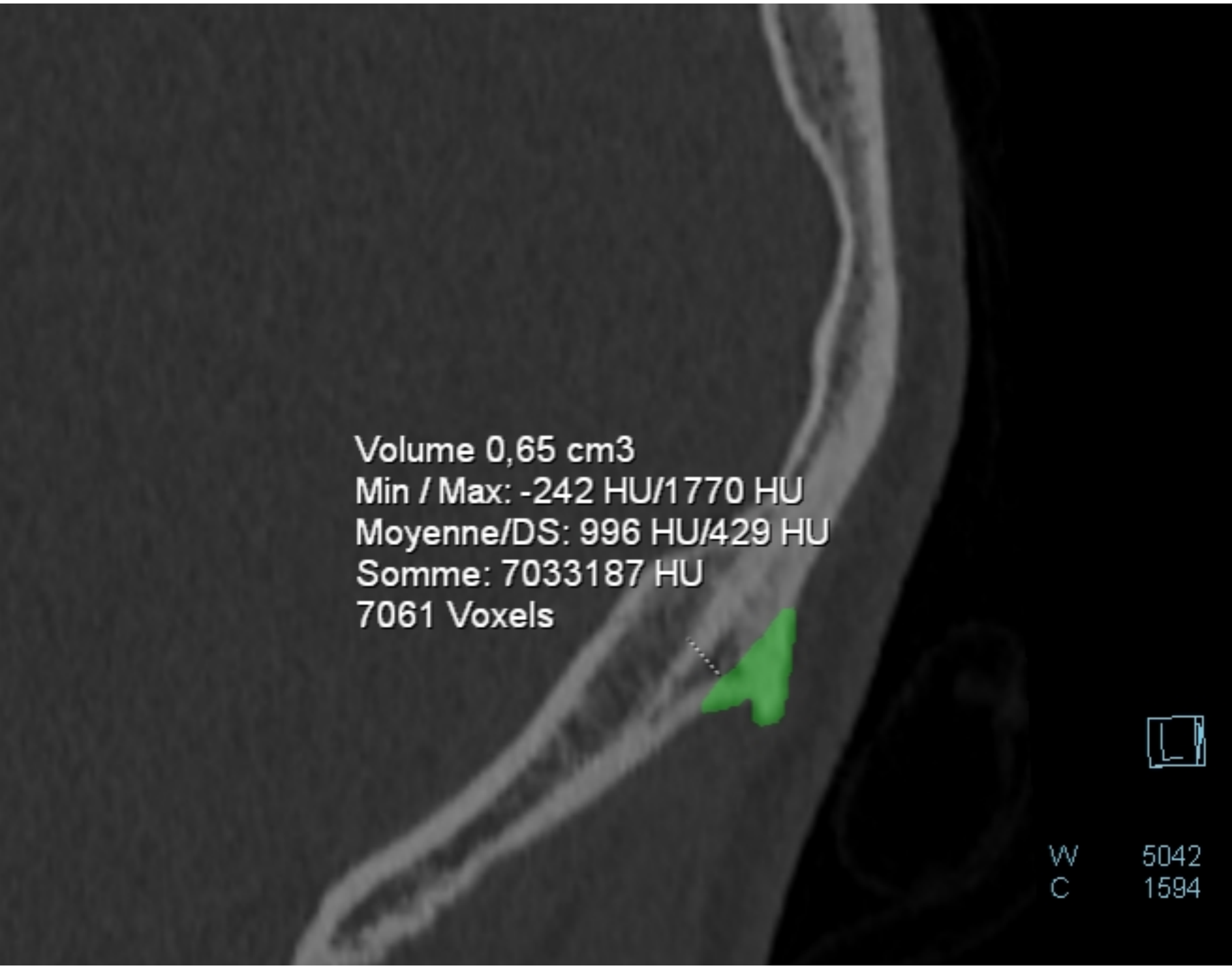

Volume 0,65 cm3
Min / Max: -242 HU/1770 HU
Moyenne/DS: 996 HU/429 HU
Somme: 7033187 HU
7061 Voxels
W 5042
C 1594

19m25

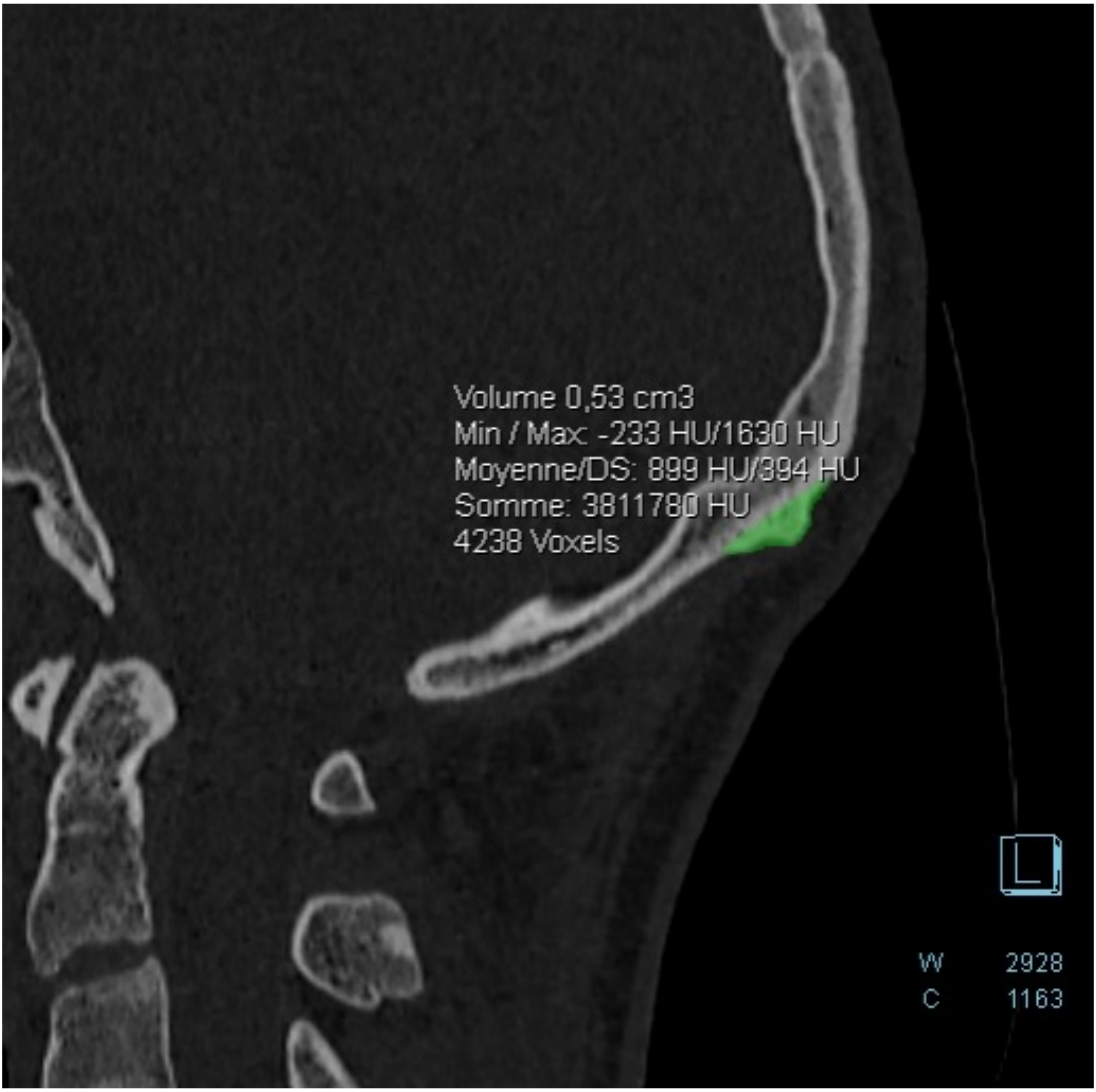

Volume 0,53 cm3
Min / Max: -233 HU/1630 HU
Moyenne/DS: 899 HU/394 HU
Somme: 3811780 HU
4238 Voxels
W 2928
C 1163

19m26

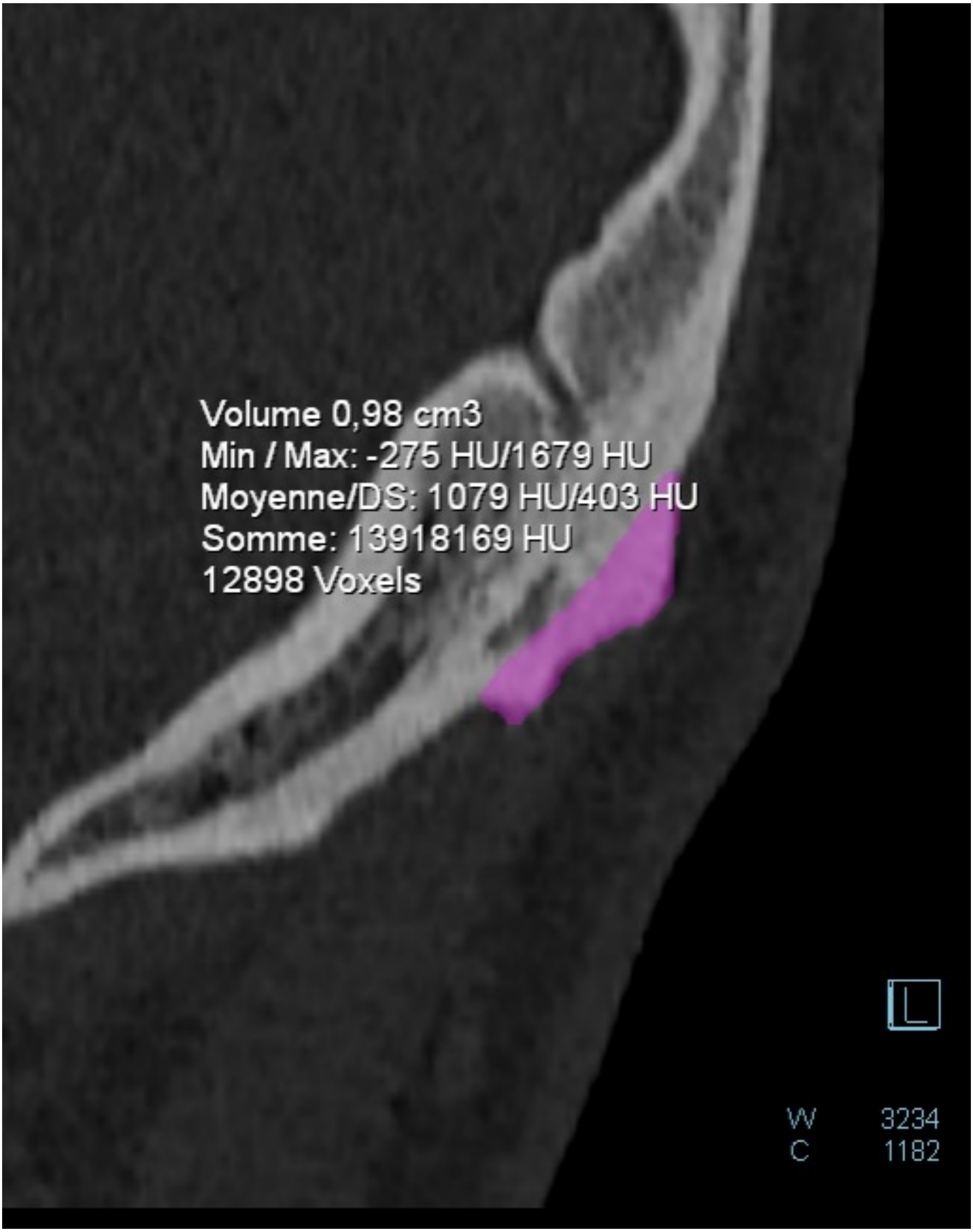
Volume 0,98 cm3
Min / Max: -275 HU/1679 HU
Moyenne/DS: 1079 HU/403 HU
Somme: 13918169 HU
12898 Voxels
L
W 3234
C 1182

19m27

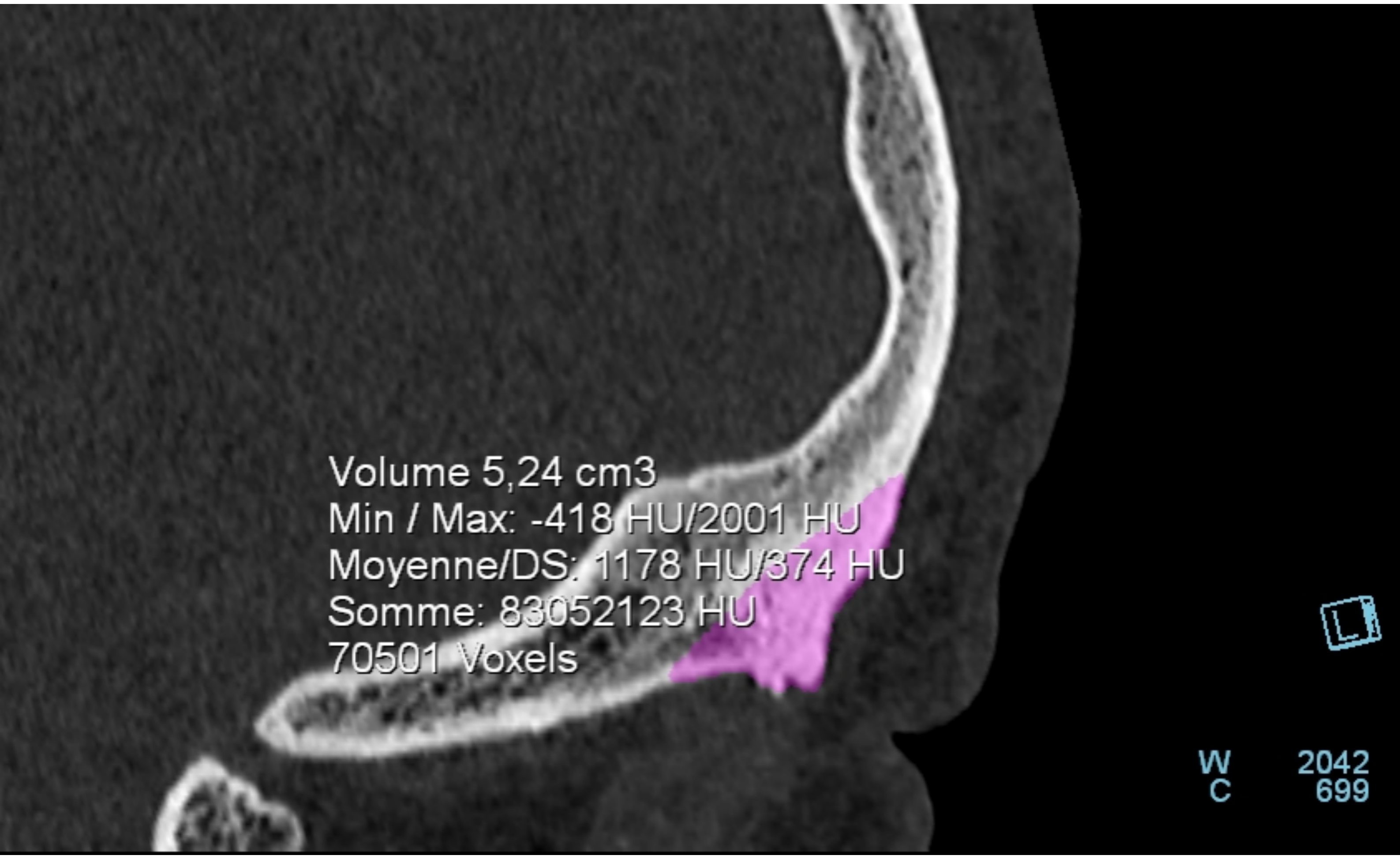
Volume 5,24 cm3
Min / Max: -418 HU/2001 HU
Moyenne/DS: 1178 HU/374 HU
Somme: 83052123 HU
70501 Voxels
W 2042
C 699

19m28

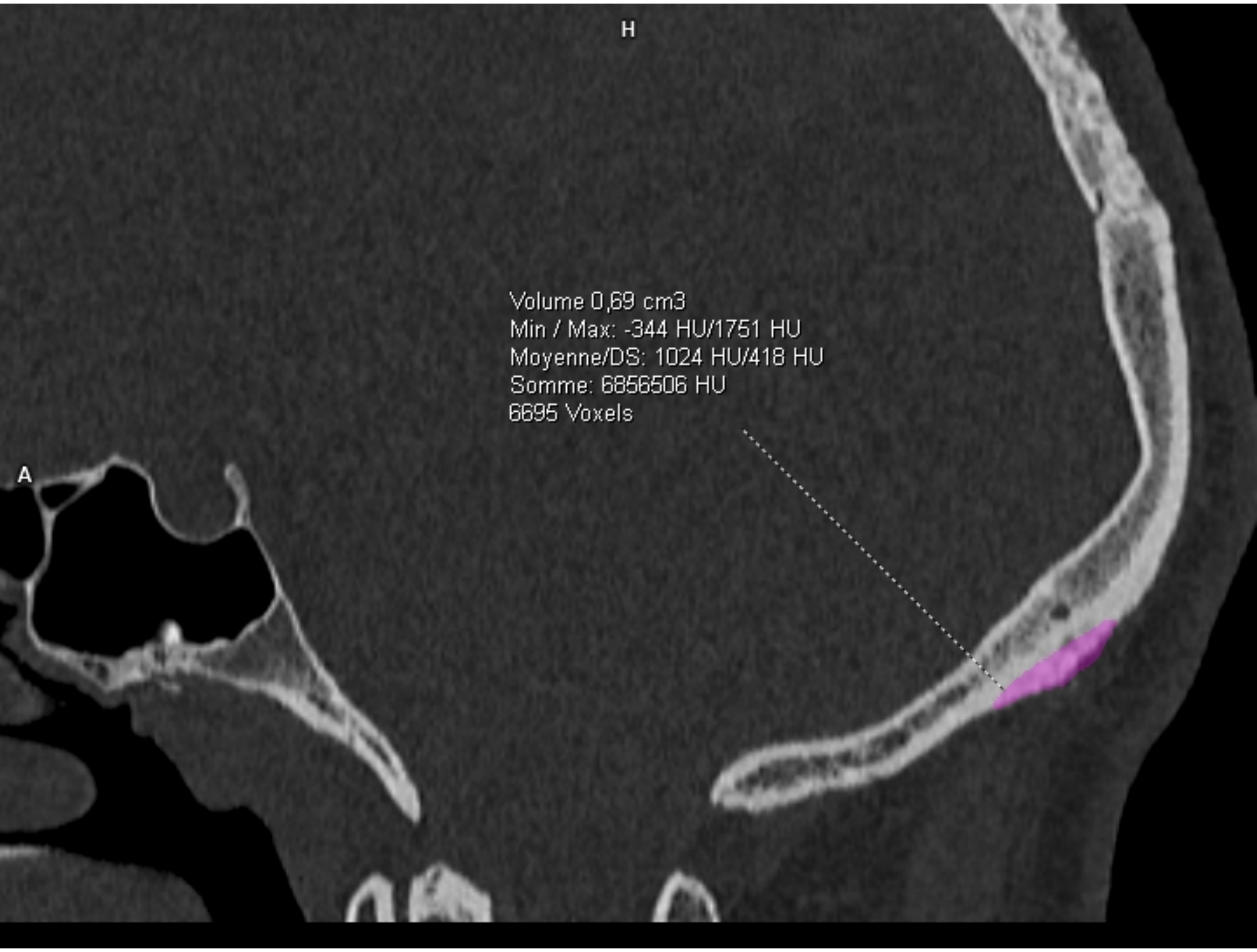
H
Volume 0,69 cm3
Min / Max: -344 HU/1751 HU
Moyenne/DS: 1024 HU/418 HU
Somme: 6856506 HU
6695 Voxels
A

19m29

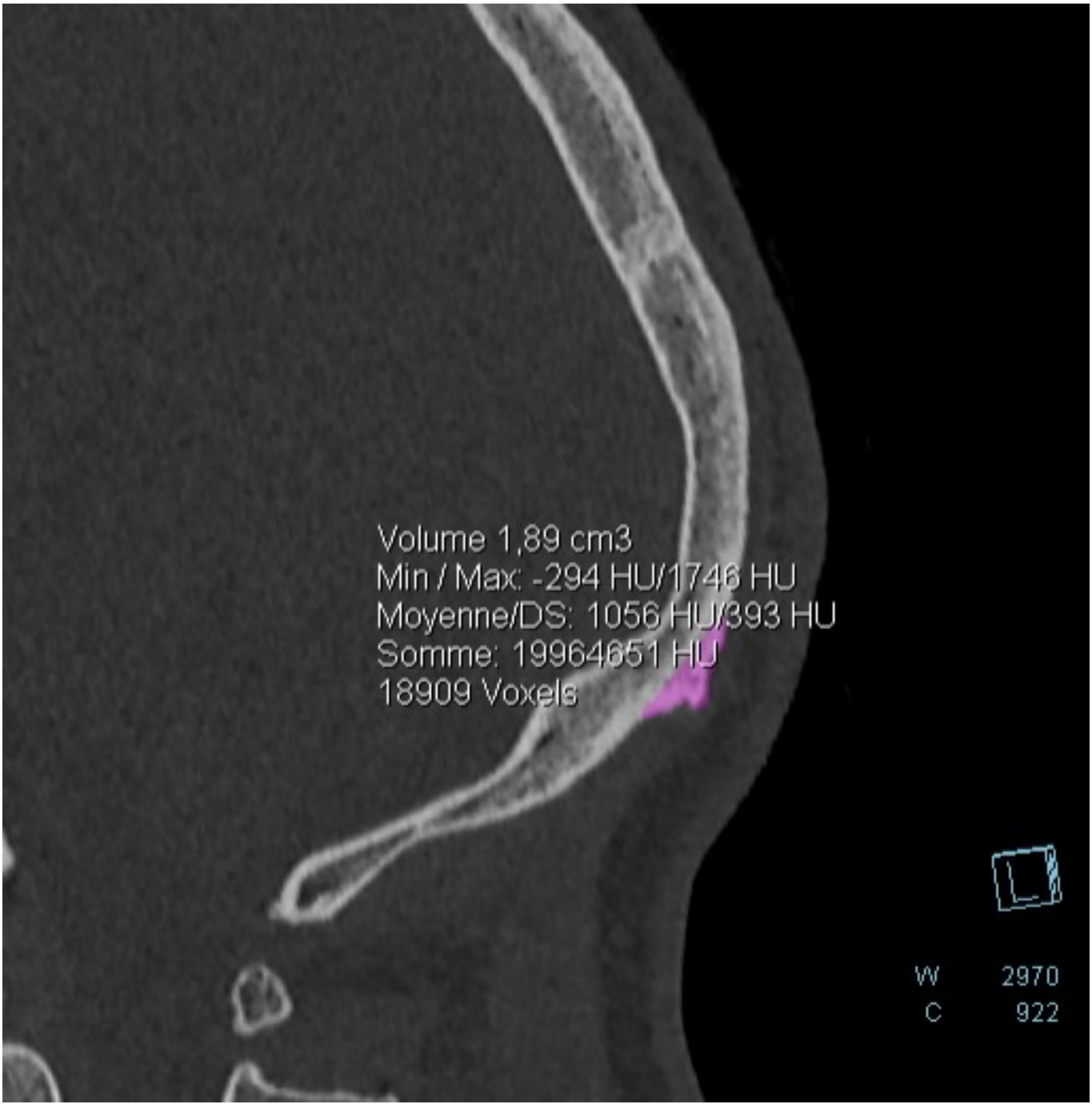
Volume 1,89 cm3
Min / Max: -294 HU/1746 HU
Moyenne/DS: 1056 HU/393 HU
Somme: 19964651 HU
18909 Voxels
W 2970
C 922

19m30

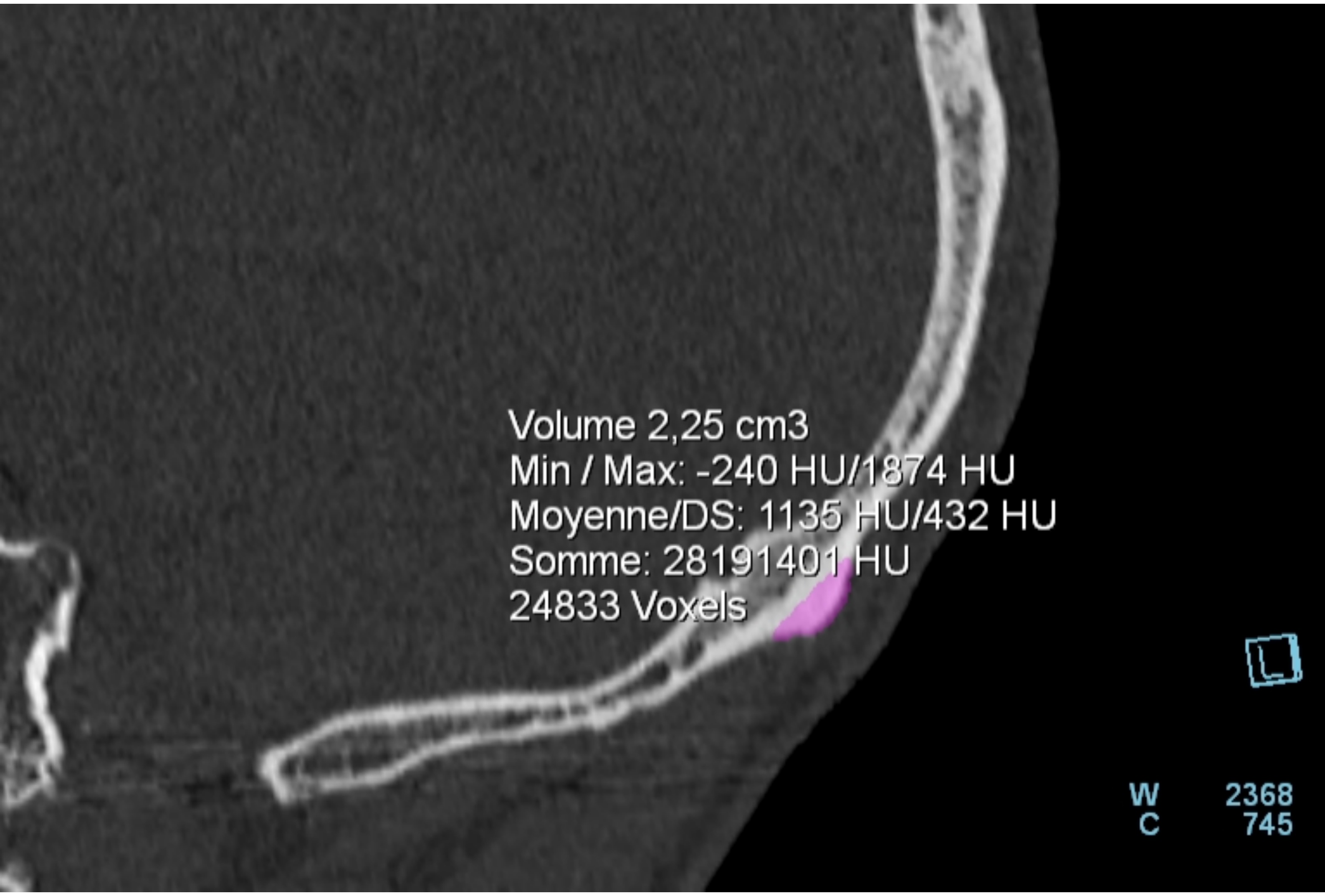
Volume 2,25 cm3
Min / Max: -240 HU/1874 HU
Moyenne/DS: 1135 HU/432 HU
Somme: 28191401 HU
24833 Voxels
W 2368
C 745

19m31

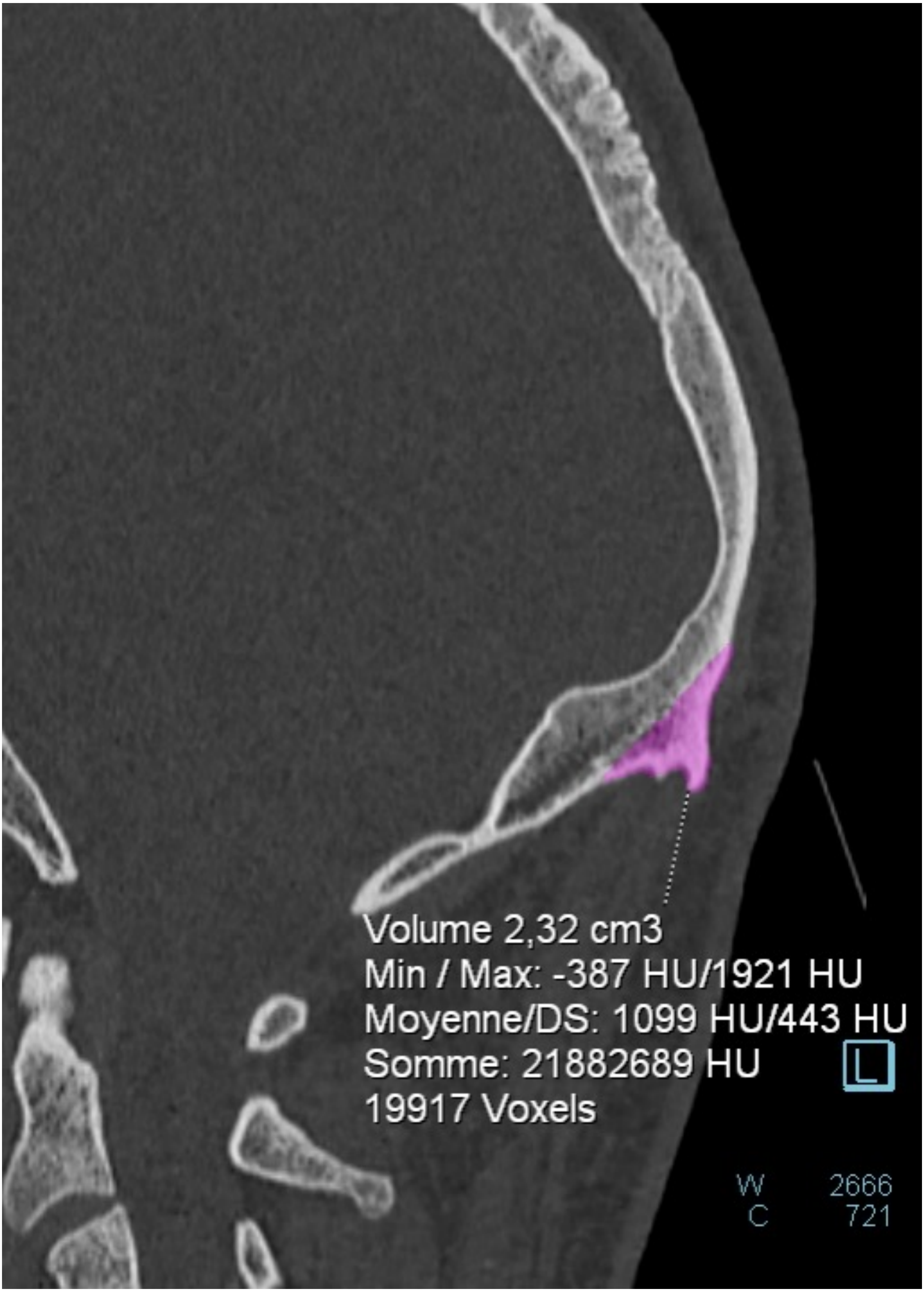
Volume 2,32 cm3
Min / Max: -387 HU/1921 HU
Moyenne/DS: 1099 HU/443 HU
Somme: 21882689 HU
19917 Voxels
L
W 2666
C 721

19m32

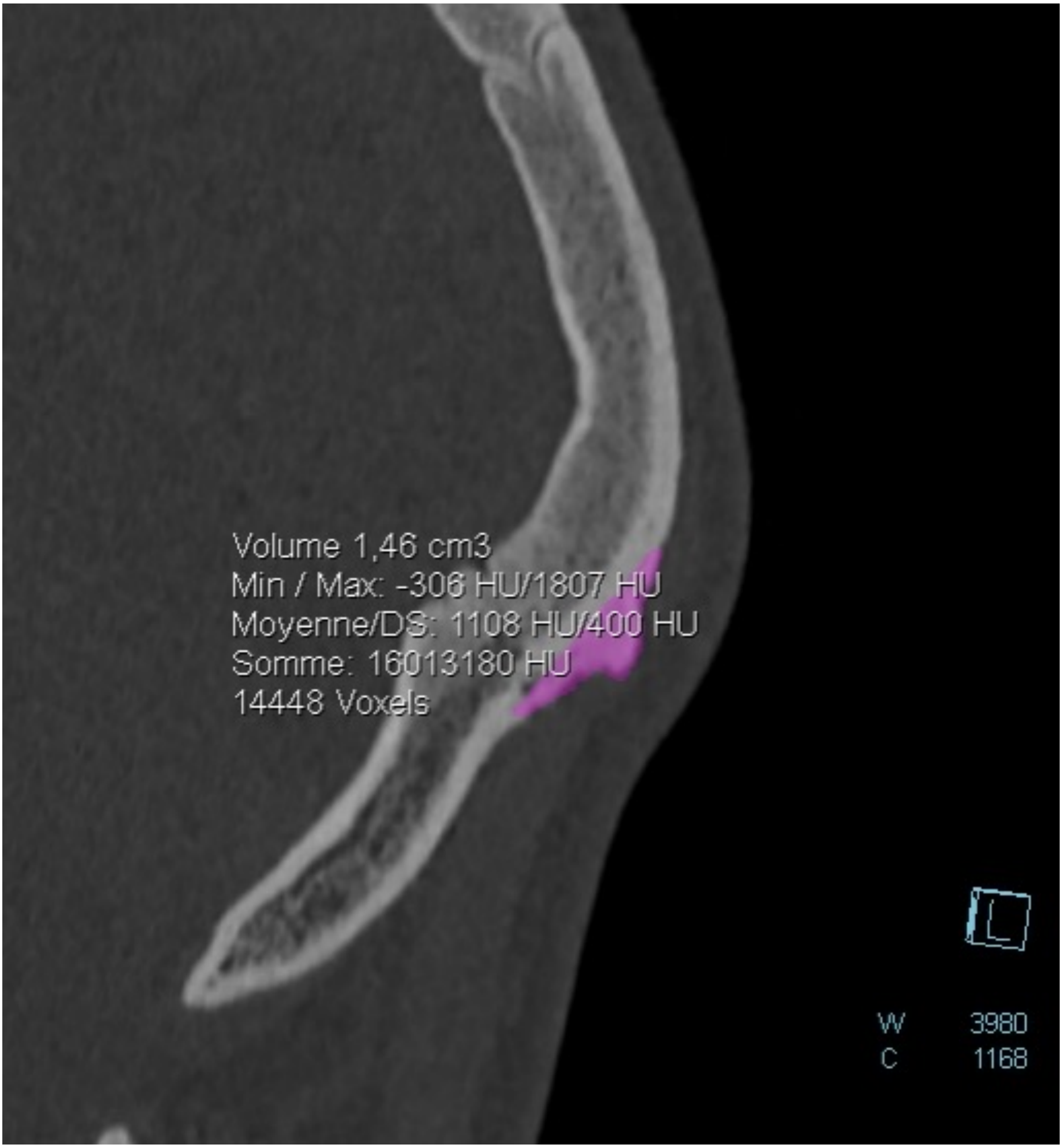
Volume 1,46 cm3
Min / Max: -306 HU/1807 HU
Moyenne/DS: 1108 HU/400 HU
Somme: 16013180 HU
14448 Voxels
W 3980
C 1168

19m33

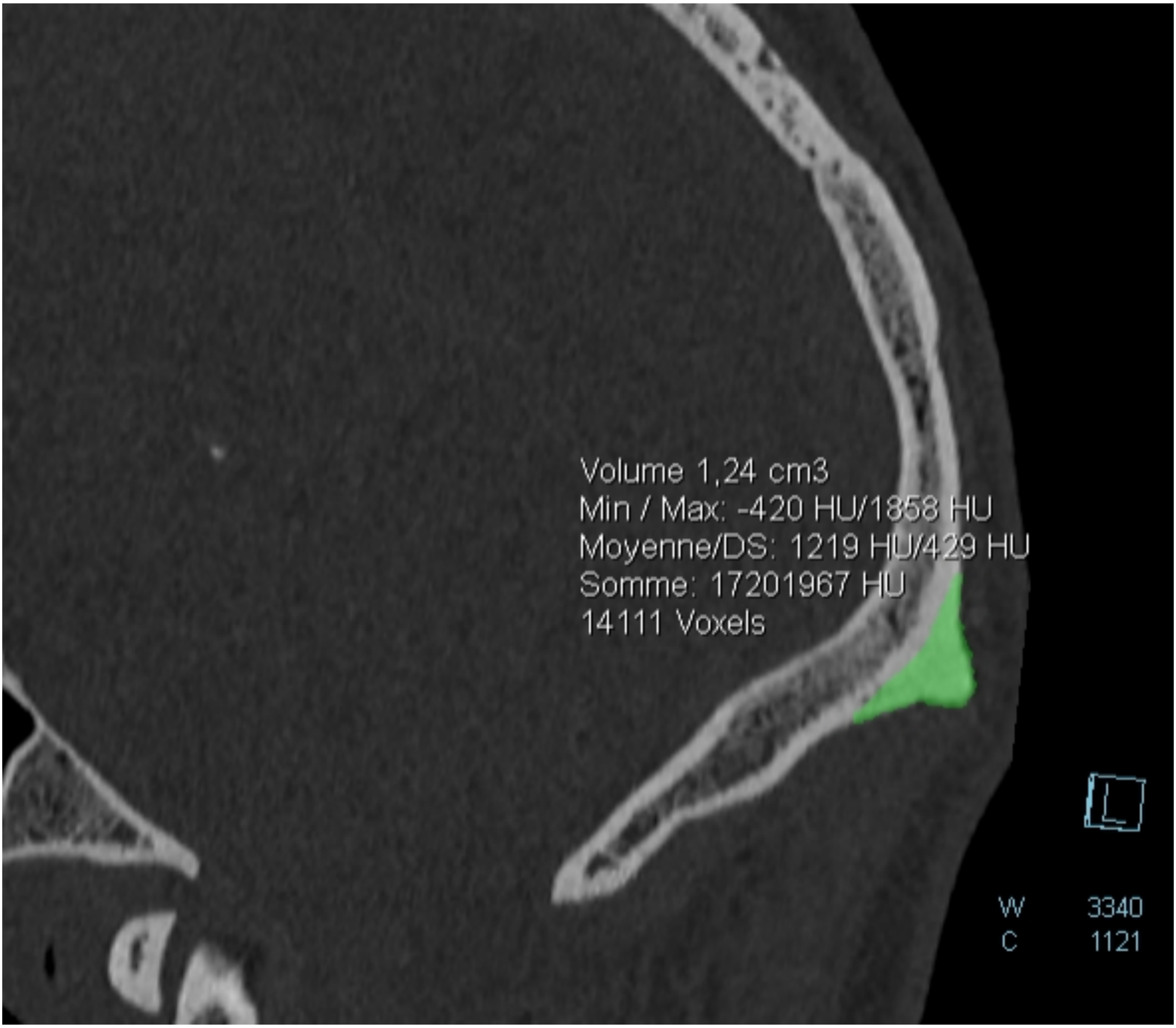
Volume 1,24 cm3
Min / Max: -420 HU/1858 HU
Moyenne/DS: 1219 HU/429 HU
Somme: 17201967 HU
14111 Voxels
W 3340
C 1121

19m34

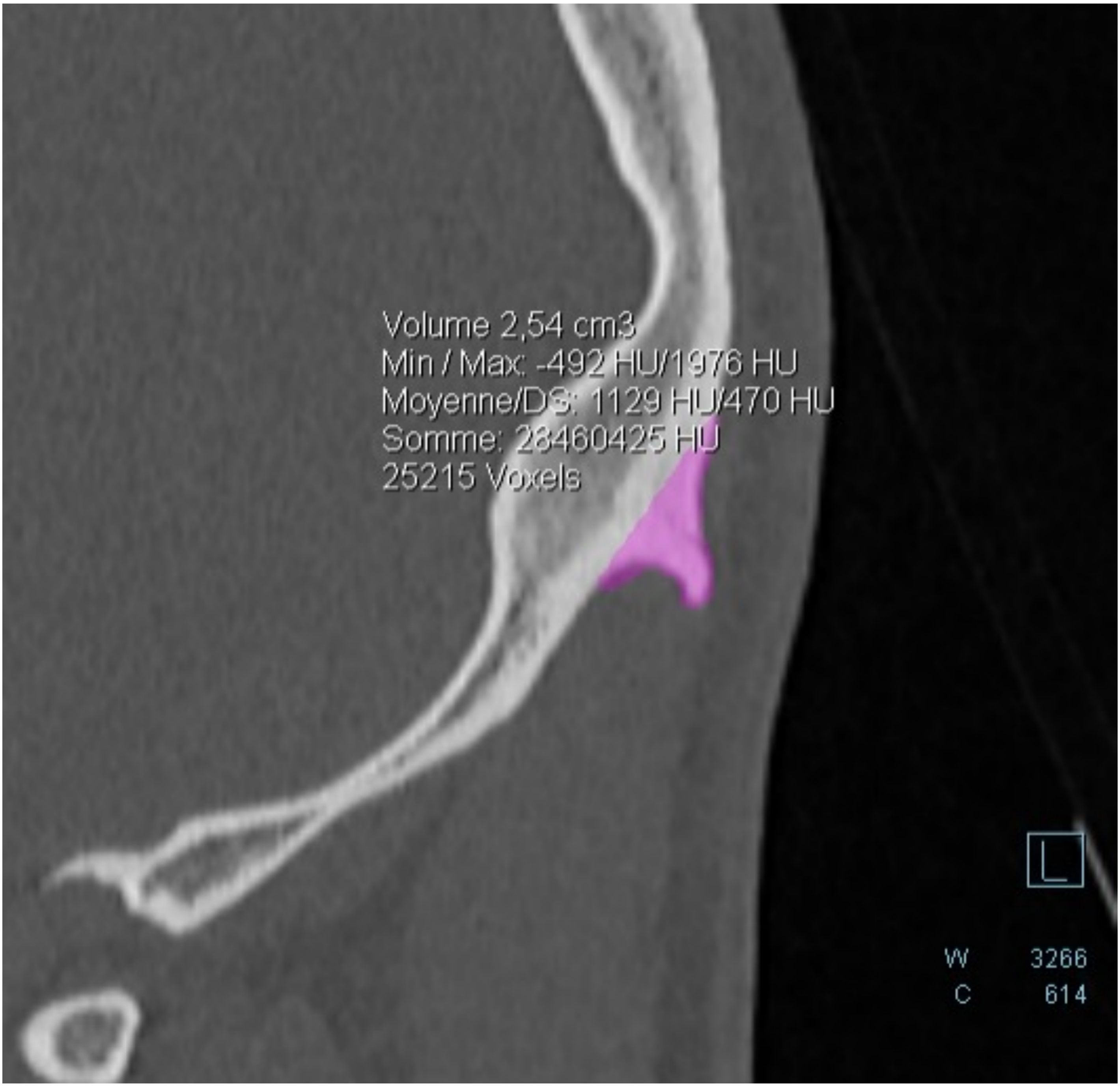

Volume 2,54 cm3
Min / Max: -492 HU/1976 HU
Moyenne/DS: 1129 HU/470 HU
Somme: 28460425 HU
25215 Voxels
L
W 3266
C 614

19m35

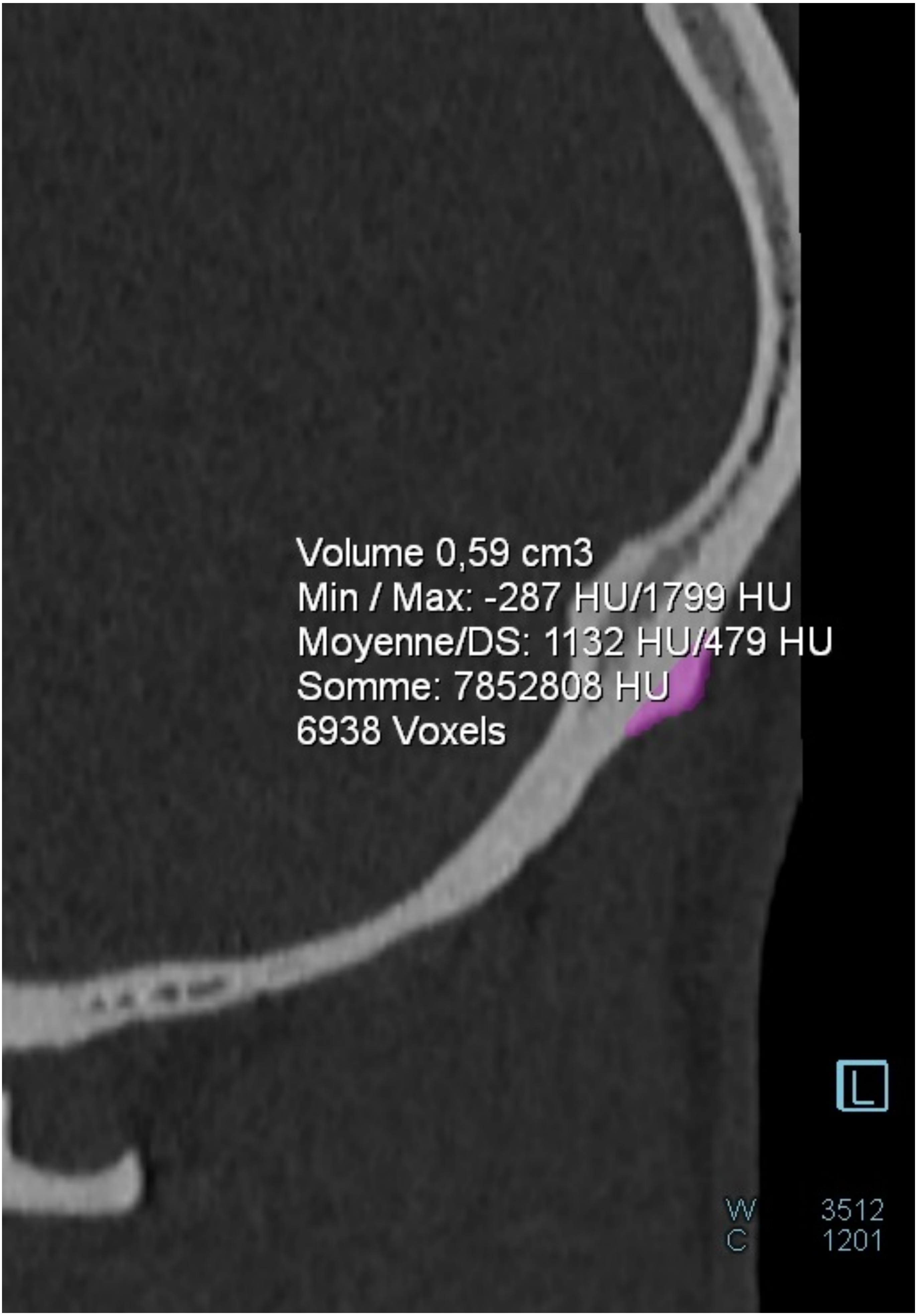
Volume 0,59 cm3
Min / Max: -287 HU/1799 HU
Moyenne/DS: 1132 HU/479 HU
Somme: 7852808 HU
6938 Voxels
L
W 3512
C 1201

19m36

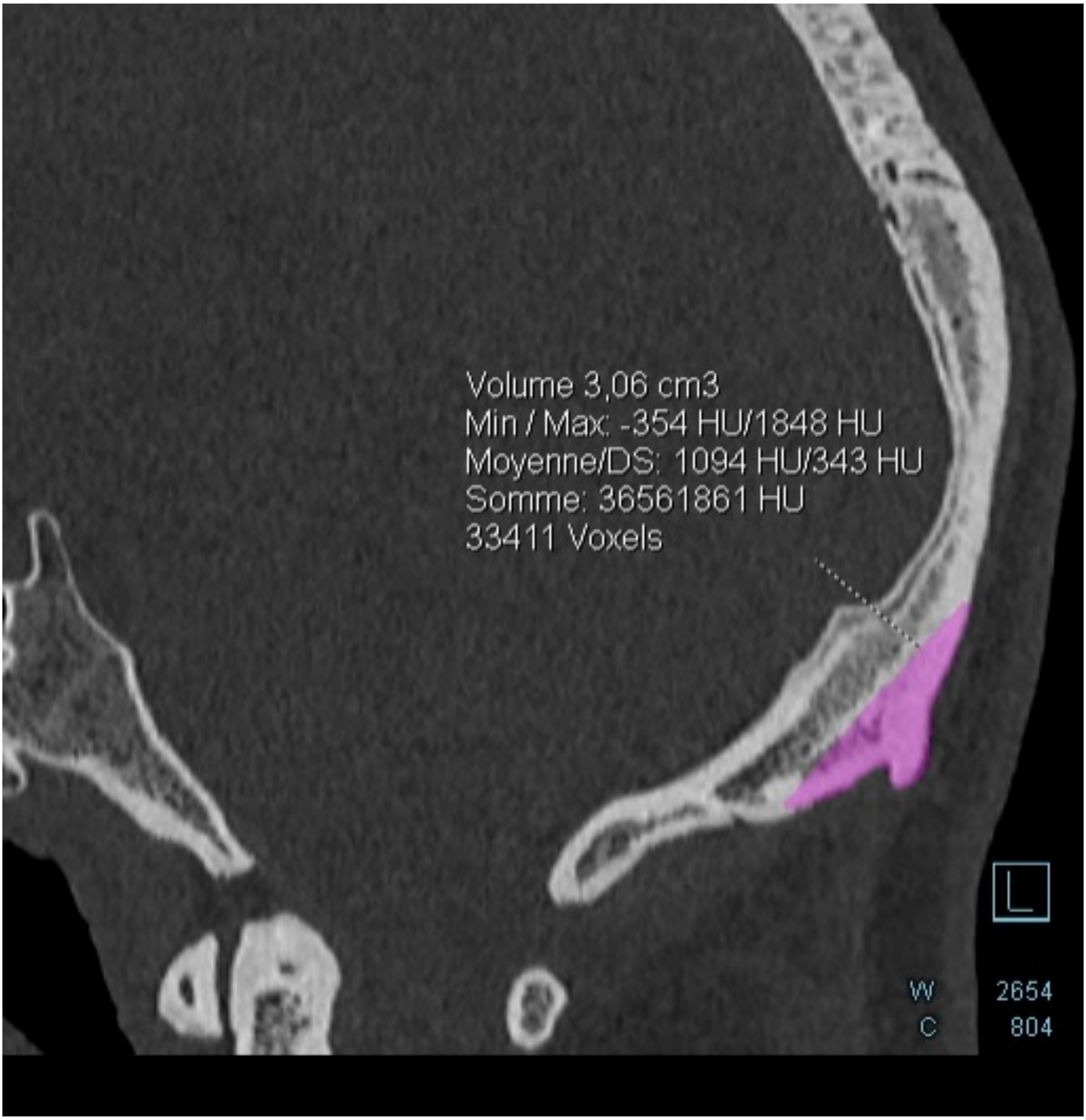
Volume 3,06 cm3
Min / Max: -354 HU/1848 HU
Moyenne/DS: 1094 HU/343 HU
Somme: 36561861 HU
33411 Voxels
L
W 2654
C 804

19m37

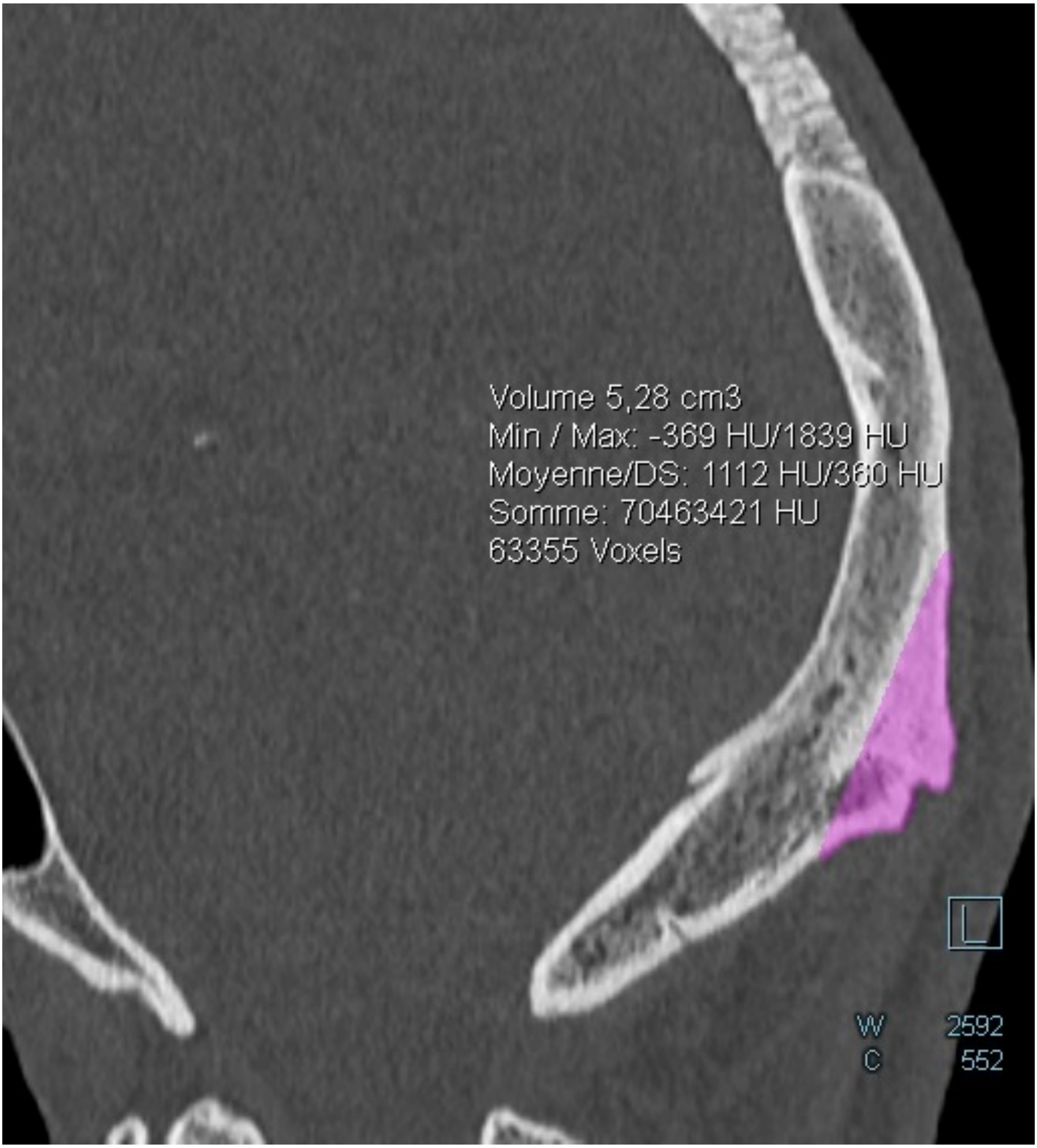
Volume 5,28 cm3
Min / Max: -369 HU/1839 HU
Moyenne/DS: 1112 HU/360 HU
Somme: 70463421 HU
63355 Voxels
L
W 2592
C 552

19m38

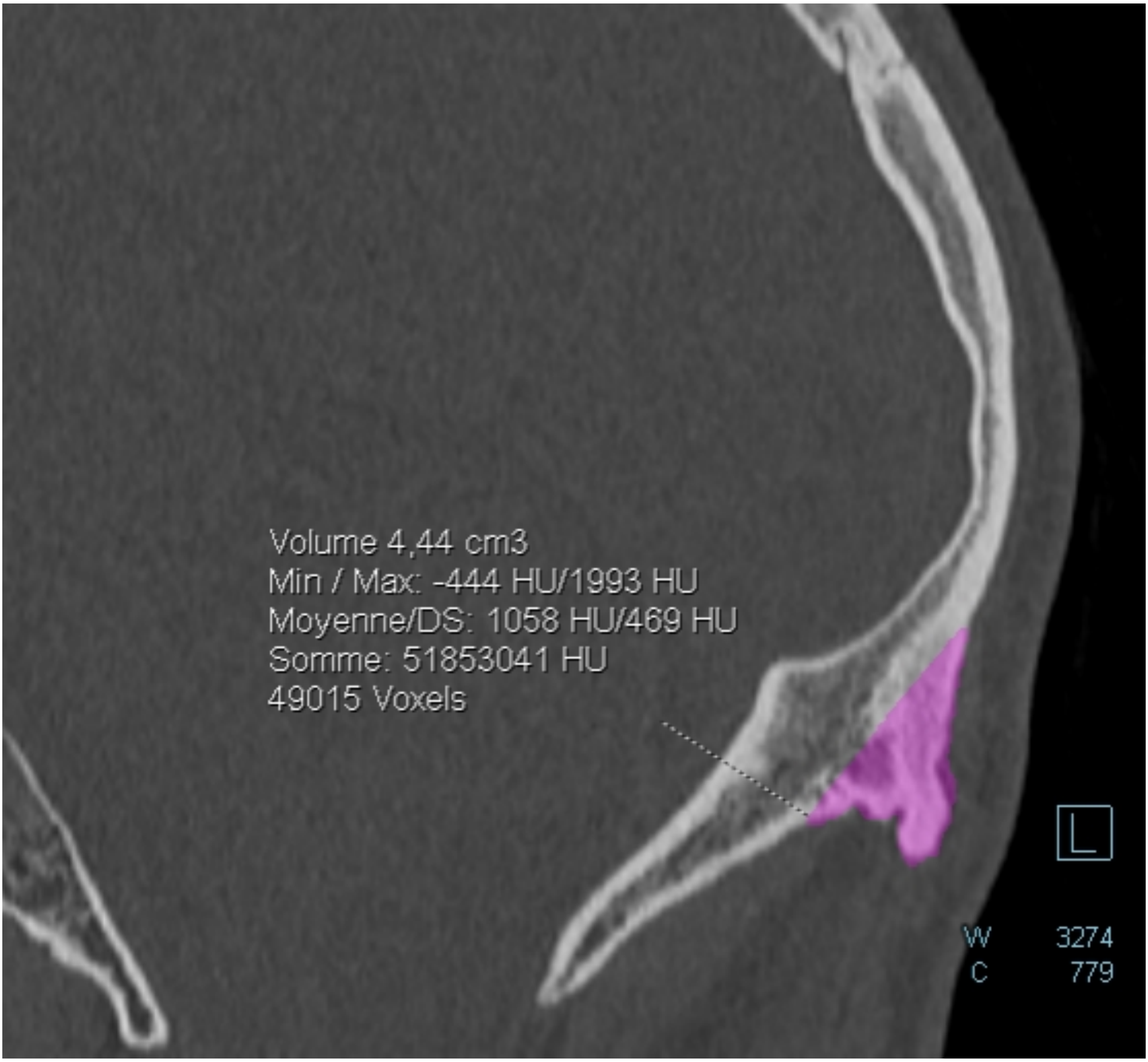
Volume 4,44 cm3
Min / Max: -444 HU/1993 HU
Moyenne/DS: 1058 HU/469 HU
Somme: 51853041 HU
49015 Voxels
L
W 3274
C 779

19m39

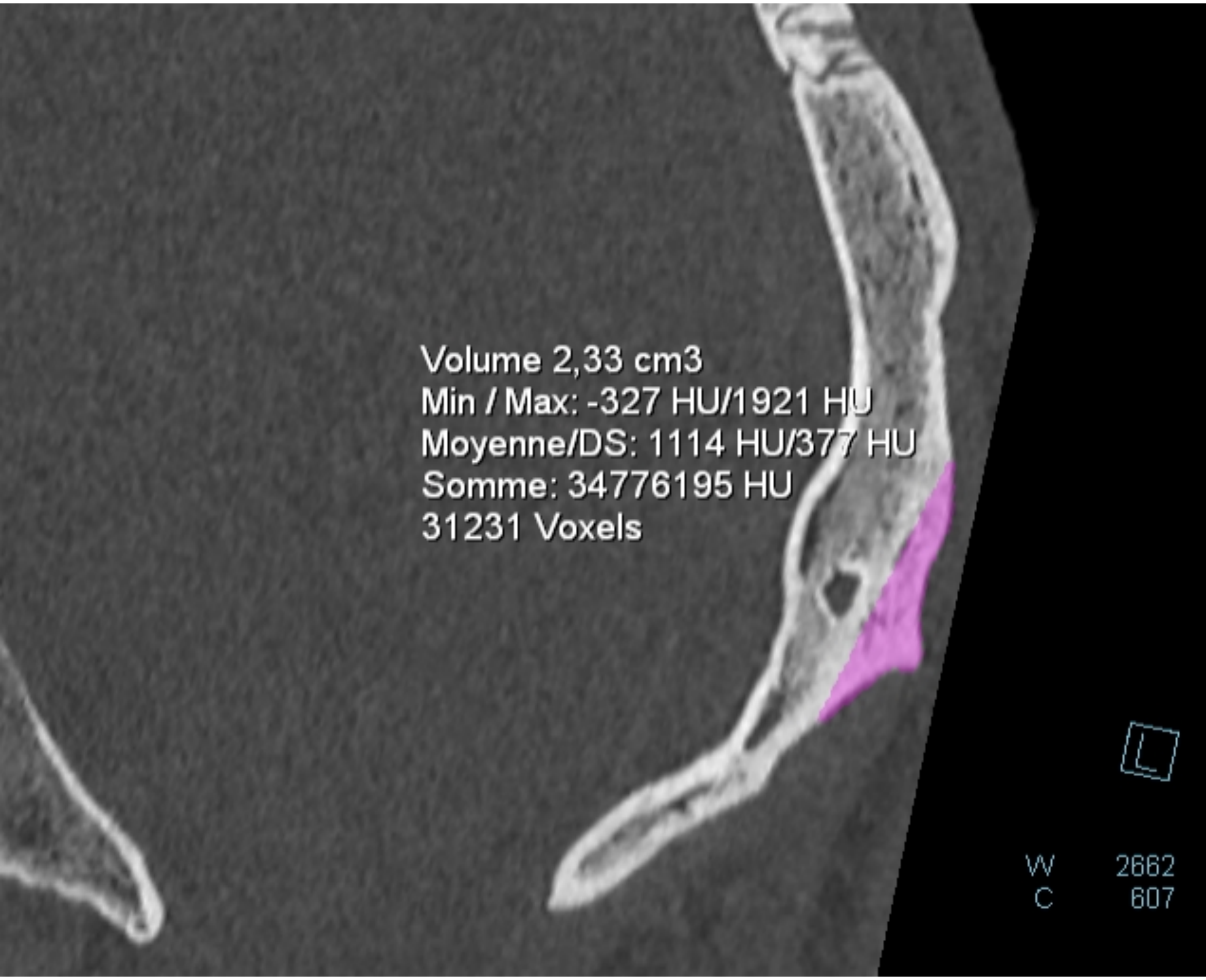
Volume 2,33 cm3
Min / Max: -327 HU/1921 HU
Moyenne/DS: 1114 HU/377 HU
Somme: 34776195 HU
31231 Voxels
L
W 2662
C 607

19m40

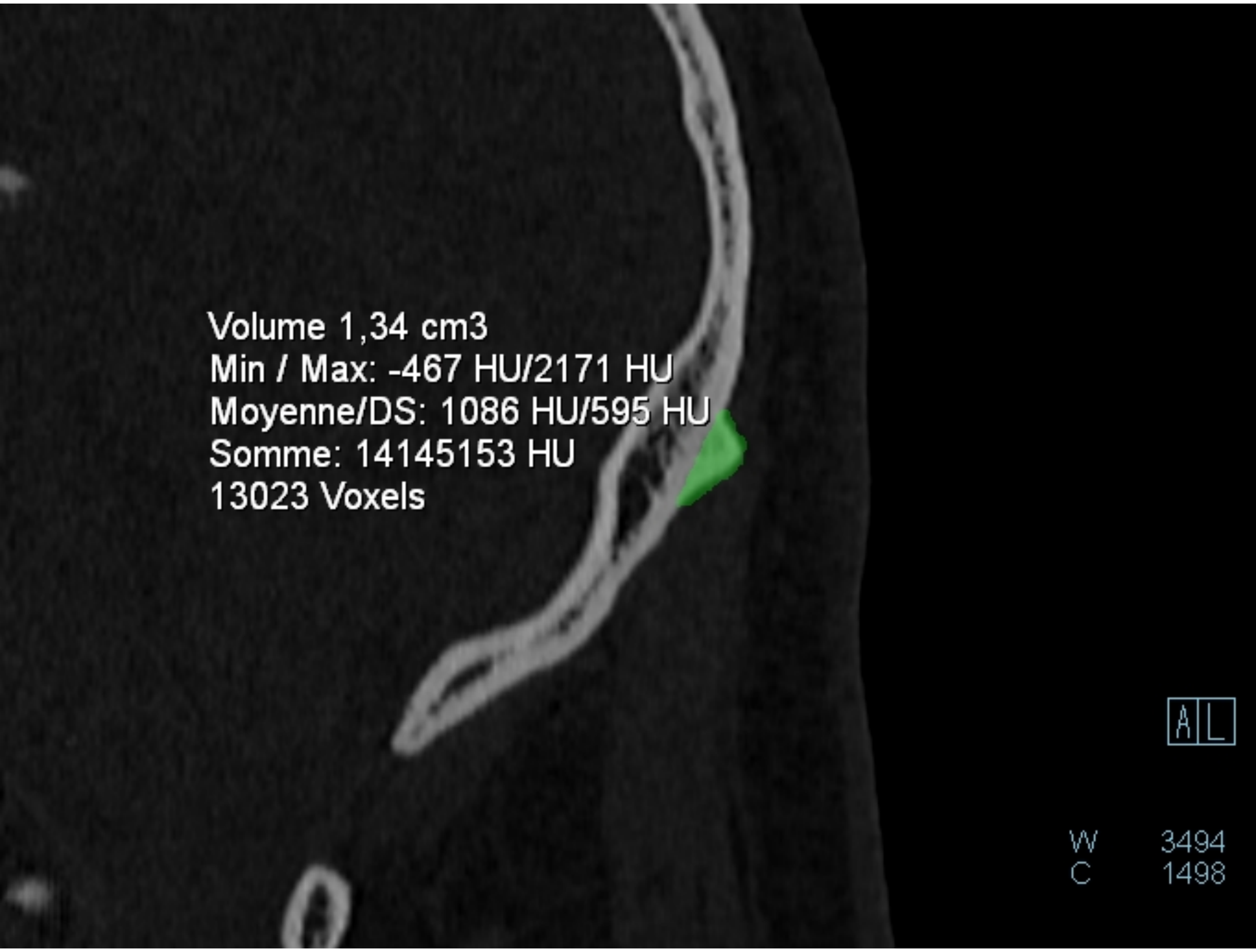
Volume 1,34 cm3
Min / Max: -467 HU/2171 HU
Moyenne/DS: 1086 HU/595 HU
Somme: 14145153 HU
13023 Voxels
A L
W 3494
C 1498

19m41

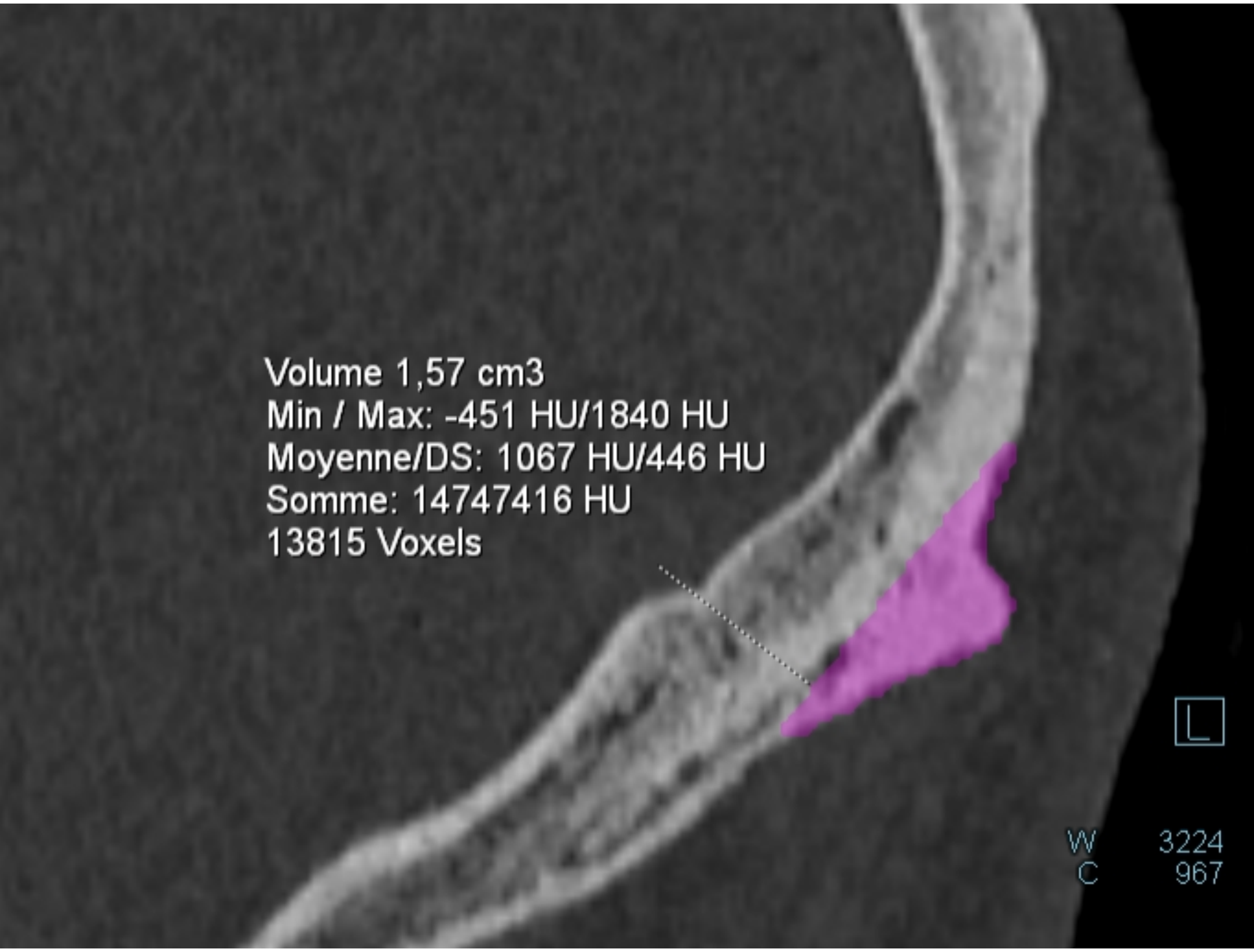
Volume 1,57 cm3
Min / Max: -451 HU/1840 HU
Moyenne/DS: 1067 HU/446 HU
Somme: 14747416 HU
13815 Voxels
L
W 3224
C 967

19m42

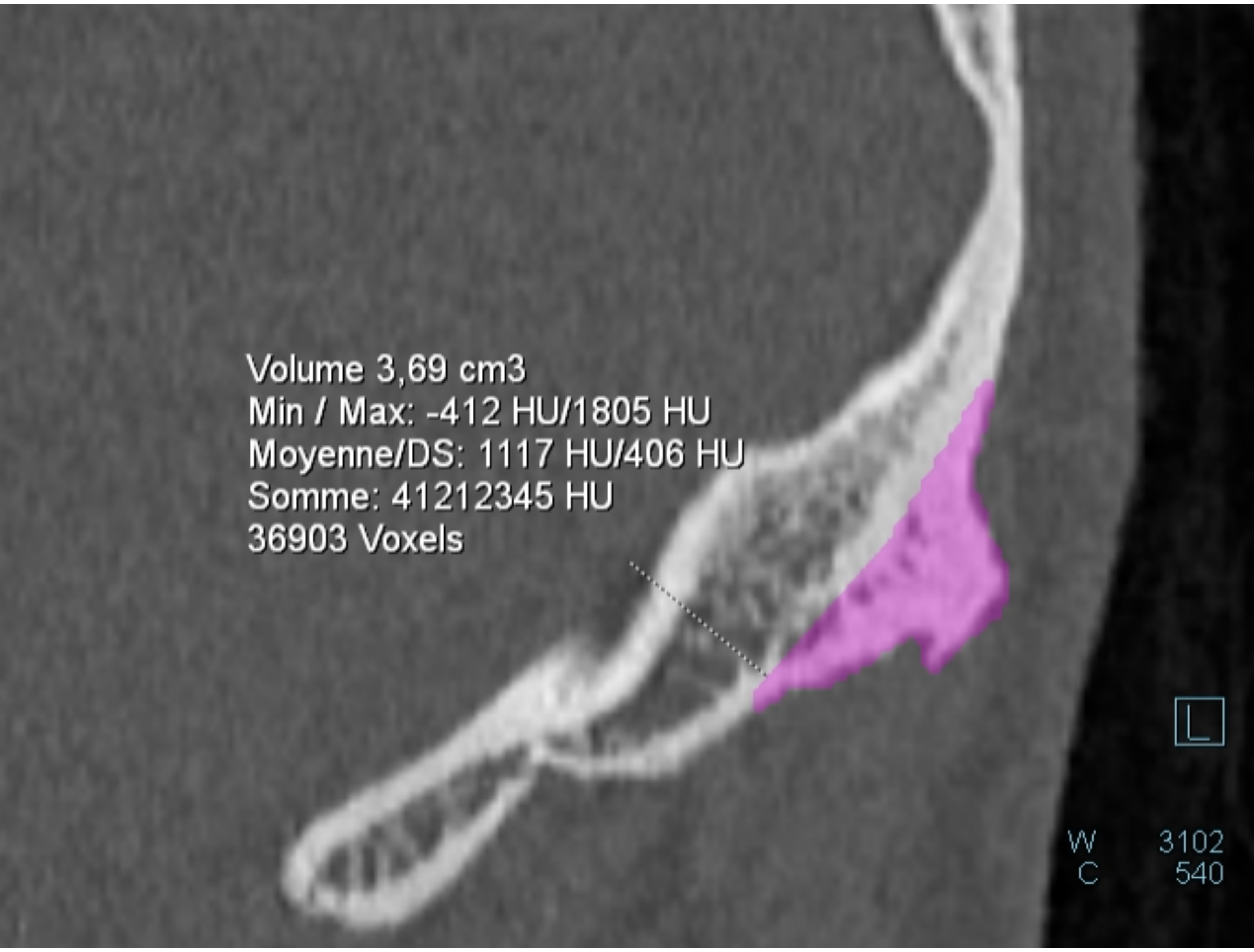
Volume 3,69 cm3
Min / Max: -412 HU/1805 HU
Moyenne/DS: 1117 HU/406 HU
Somme: 41212345 HU
36903 Voxels
L
W 3102
C 540

19m43

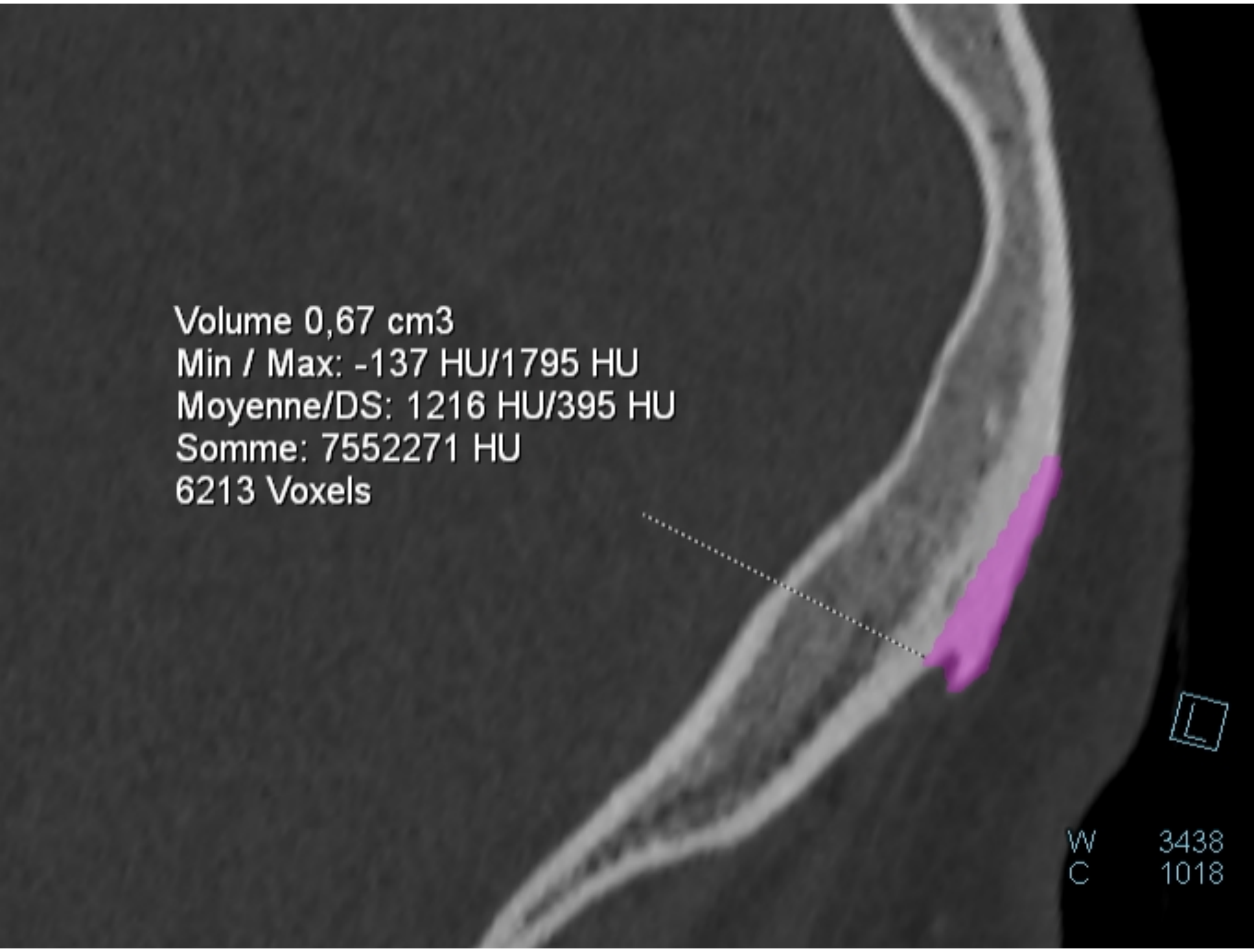
Volume 0,67 cm3
Min / Max: -137 HU/1795 HU
Moyenne/DS: 1216 HU/395 HU
Somme: 7552271 HU
6213 Voxels
L
W 3438
C 1018

19m44

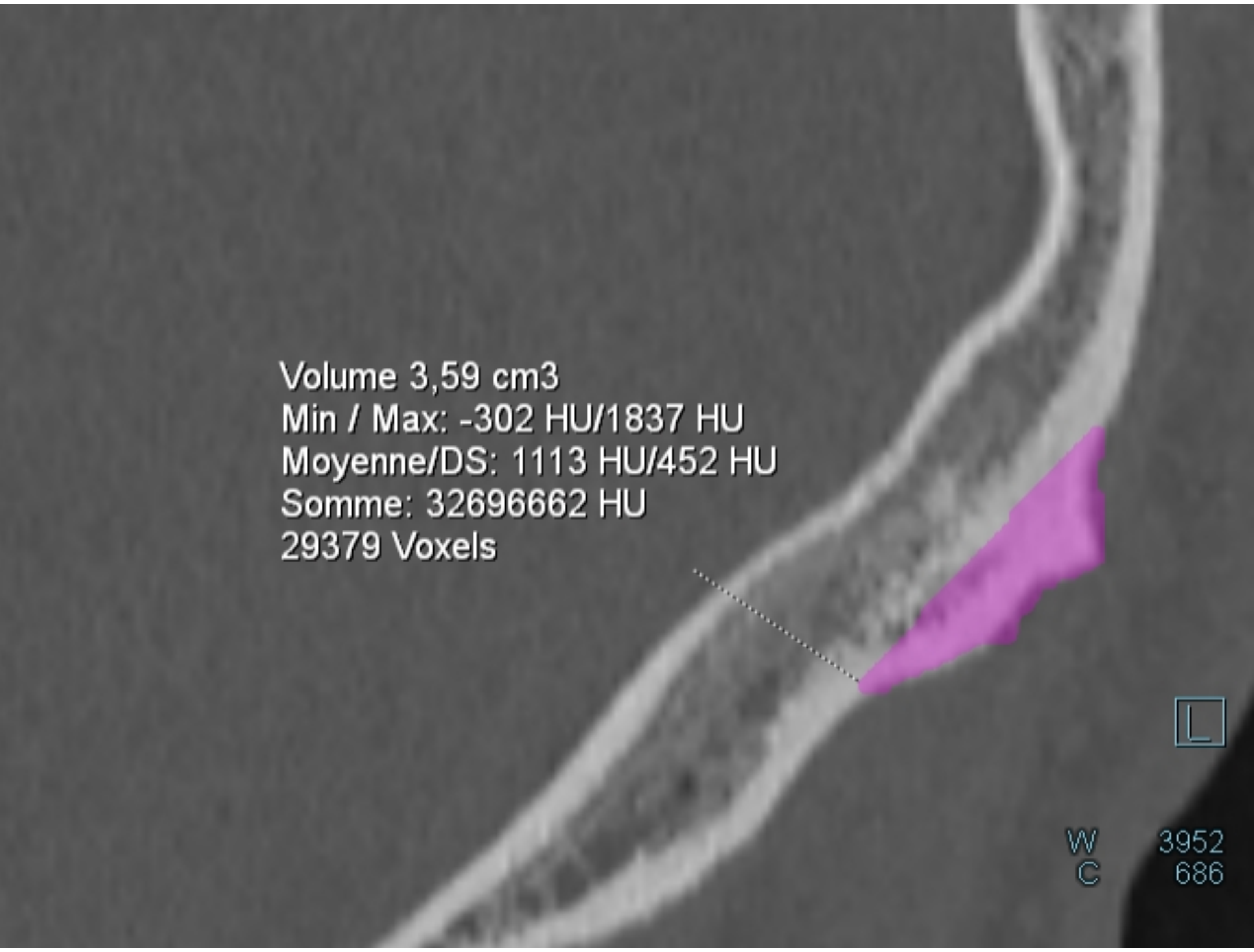
Volume 3,59 cm3
Min / Max: -302 HU/1837 HU
Moyenne/DS: 1113 HU/452 HU
Somme: 32696662 HU
29379 Voxels
L
W 3952
C 686

19m45

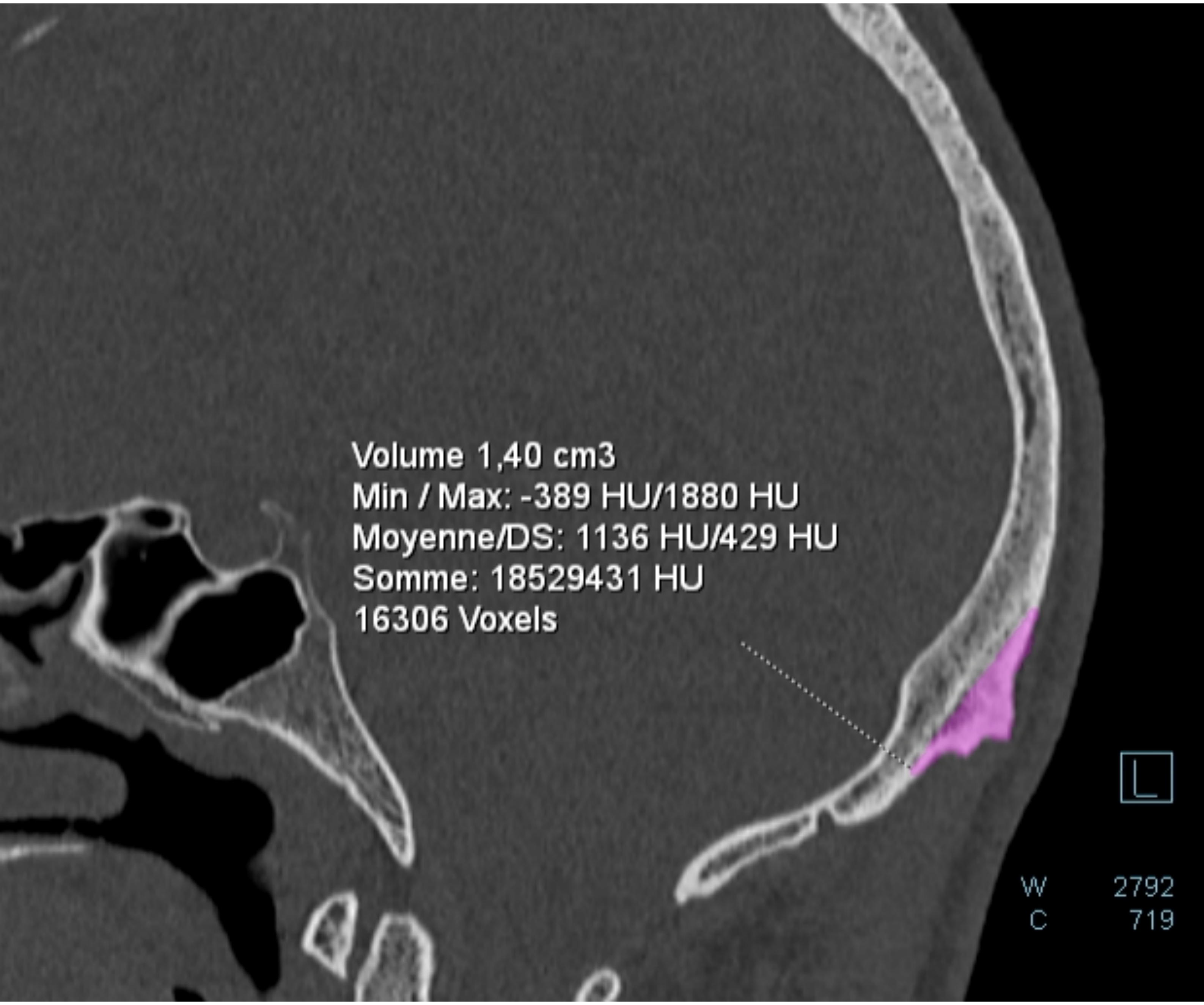

Volume 1,40 cm3
Min / Max: -389 HU/1880 HU
Moyenne/DS: 1136 HU/429 HU
Somme: 18529431 HU
16306 Voxels
L
W 2792
C 719

19m46

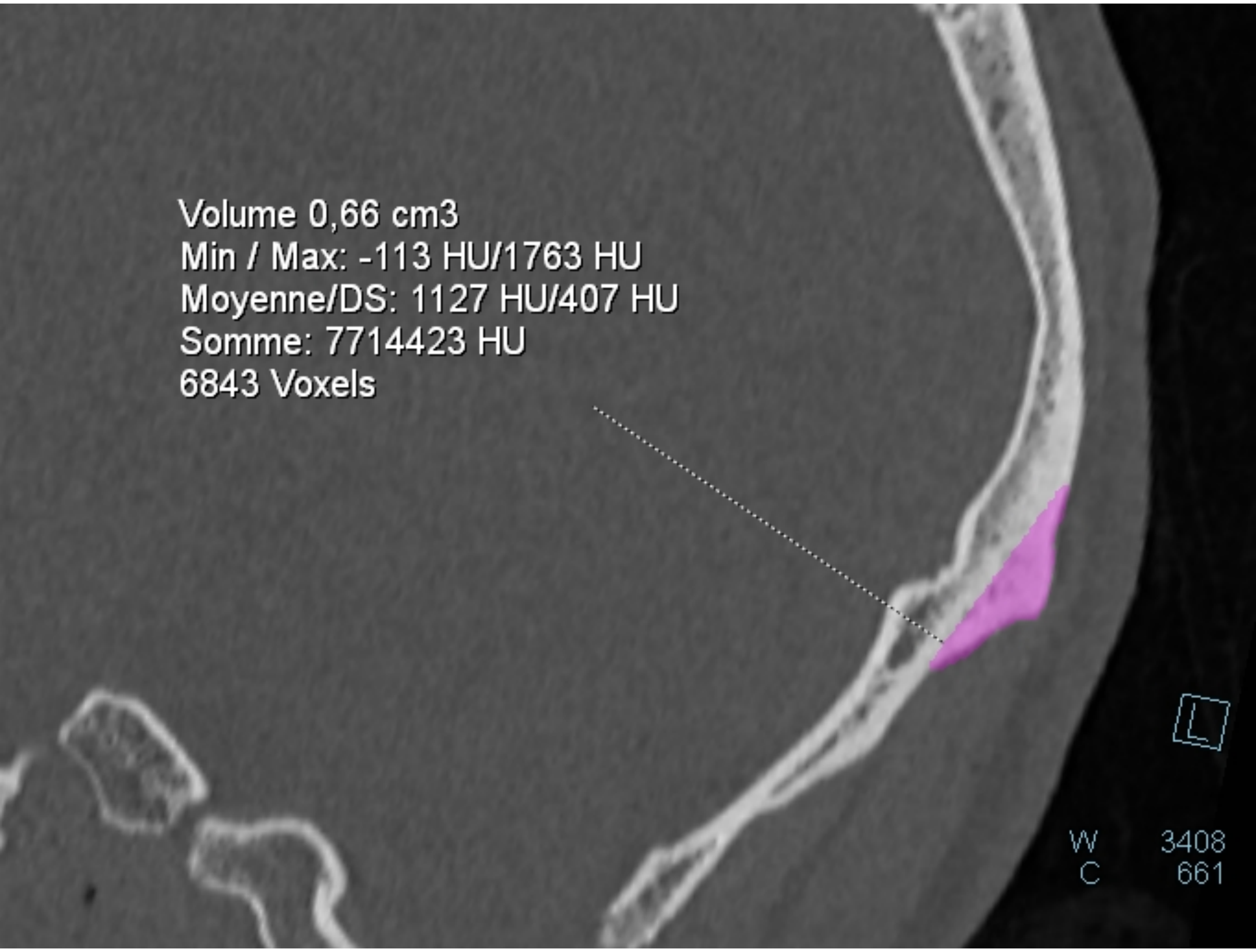
Volume 0,66 cm3
Min / Max: -113 HU/1763 HU
Moyenne/DS: 1127 HU/407 HU
Somme: 7714423 HU
6843 Voxels
L
W 3408
C 661

19m47

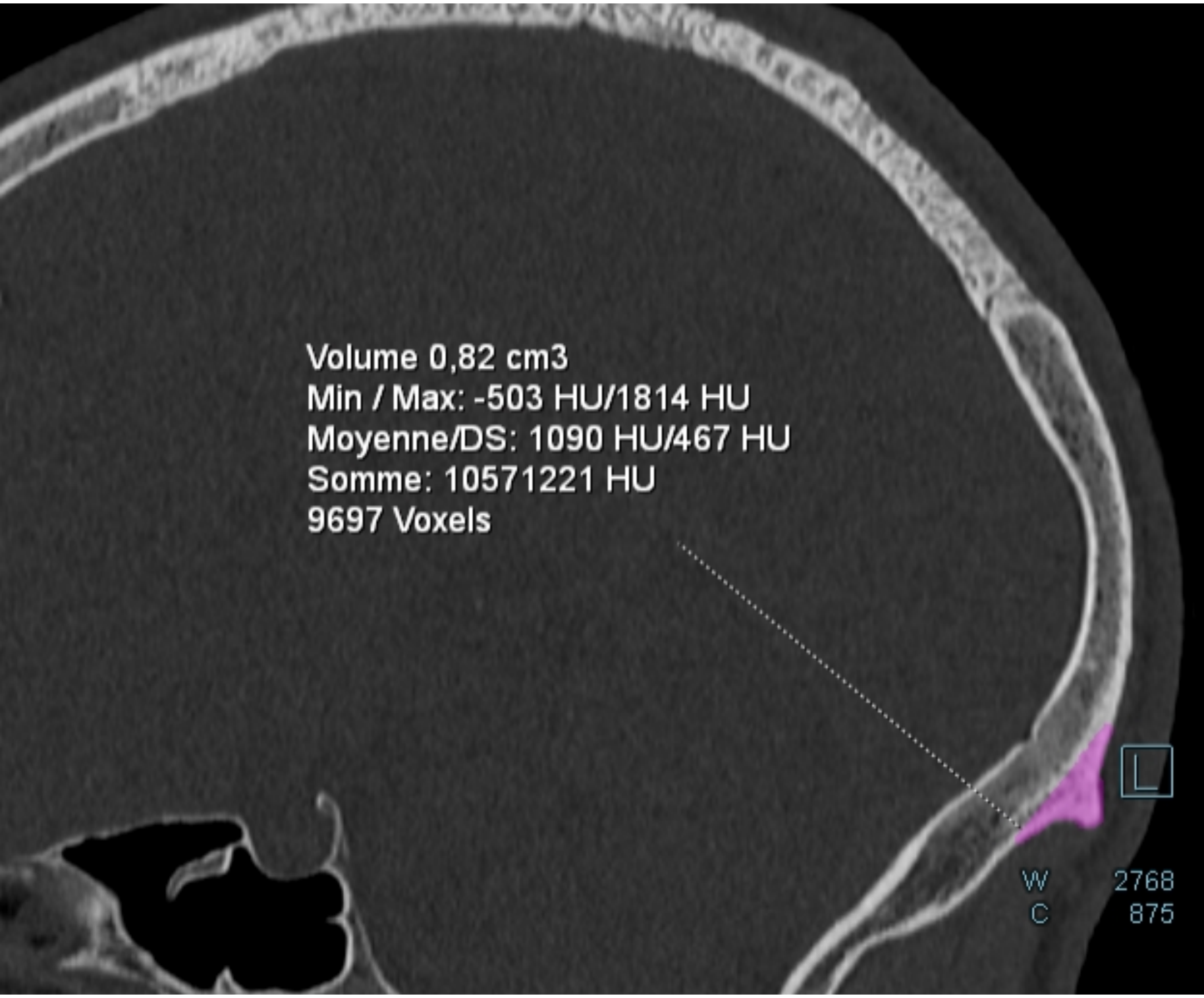
Volume 0,82 cm3
Min / Max: -503 HU/1814 HU
Moyenne/DS: 1090 HU/467 HU
Somme: 10571221 HU
9697 Voxels
L
W 2768
C 875

19m48

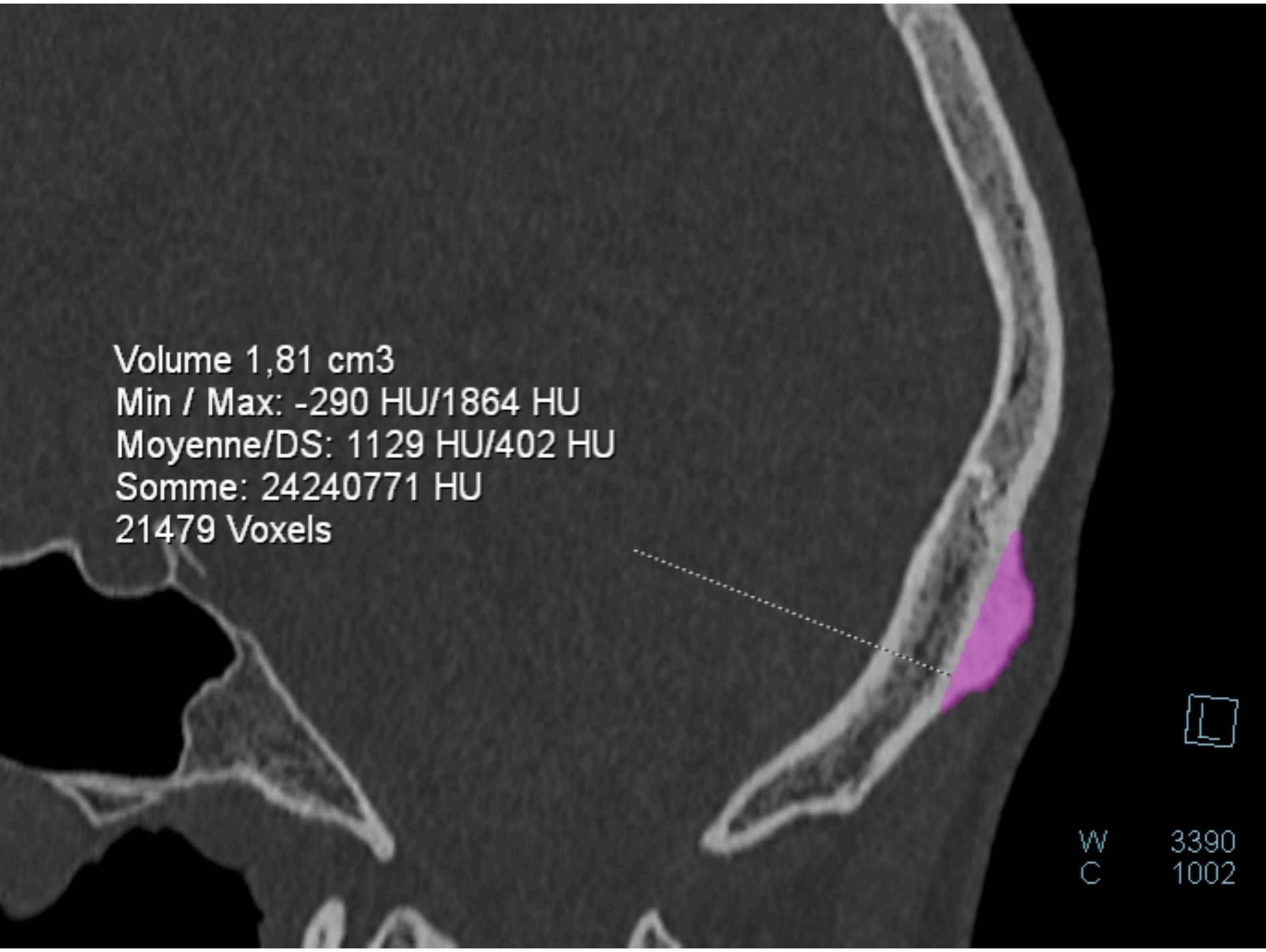

Volume 1,81 cm3
Min / Max: -290 HU/1864 HU
Moyenne/DS: 1129 HU/402 HU
Somme: 24240771 HU
21479 Voxels
W 3390
C 1002

19m49

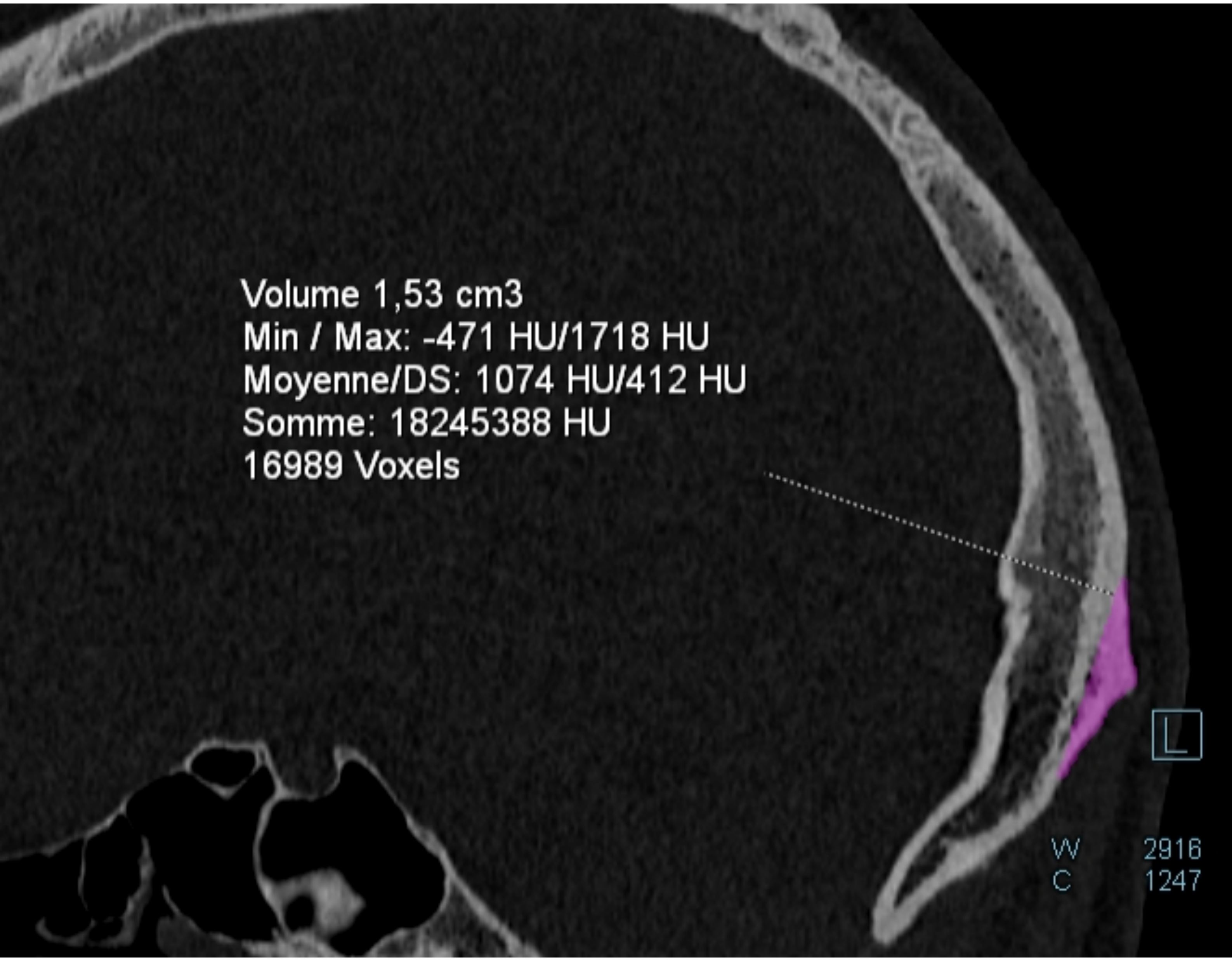
Volume 1,53 cm3
Min / Max: -471 HU/1718 HU
Moyenne/DS: 1074 HU/412 HU
Somme: 18245388 HU
16989 Voxels
L
W 2916
C 1247

19m50

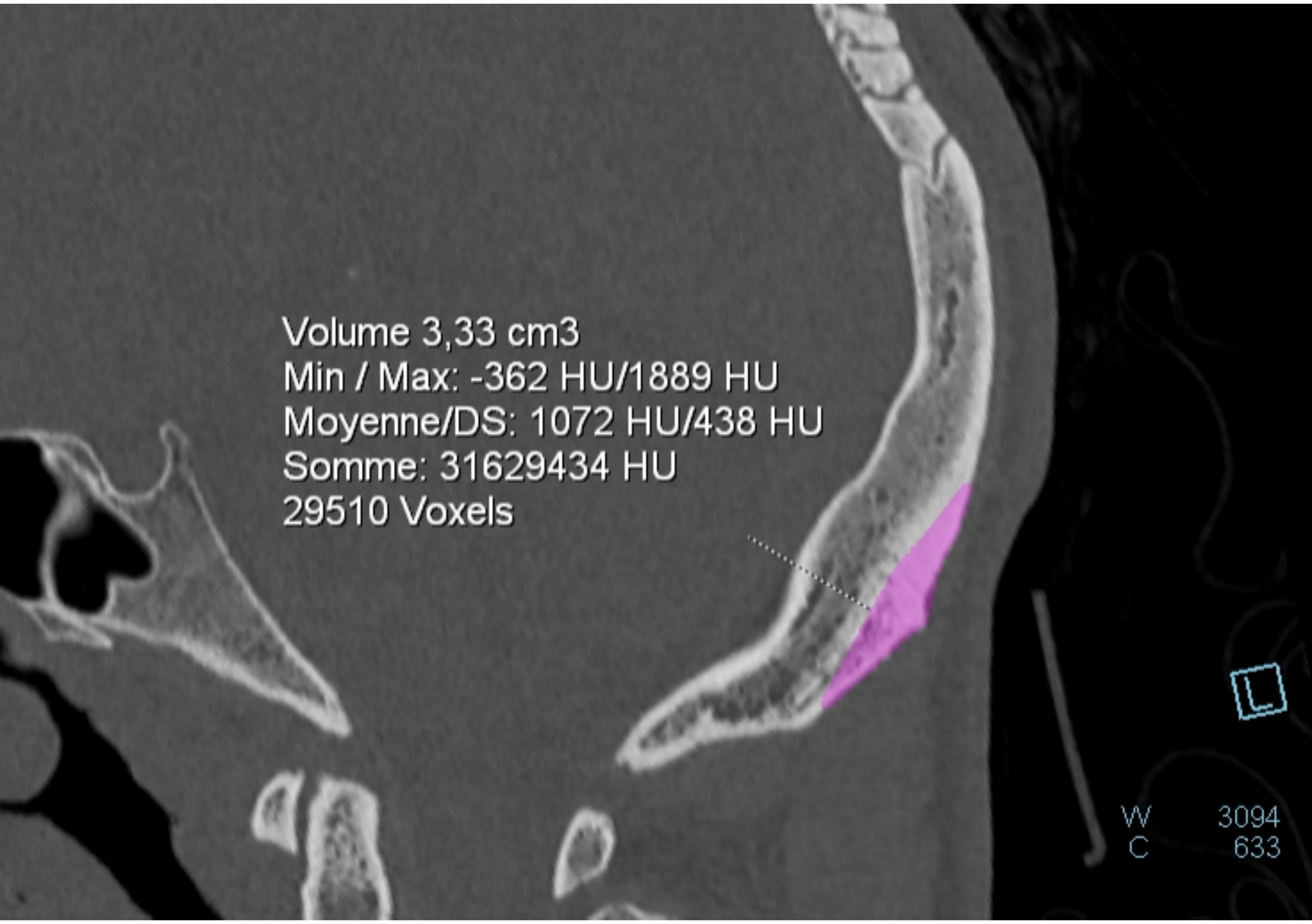
Volume 3,33 cm3
Min / Max: -362 HU/1889 HU
Moyenne/DS: 1072 HU/438 HU
Somme: 31629434 HU
29510 Voxels
W 3094
C 633

19m51

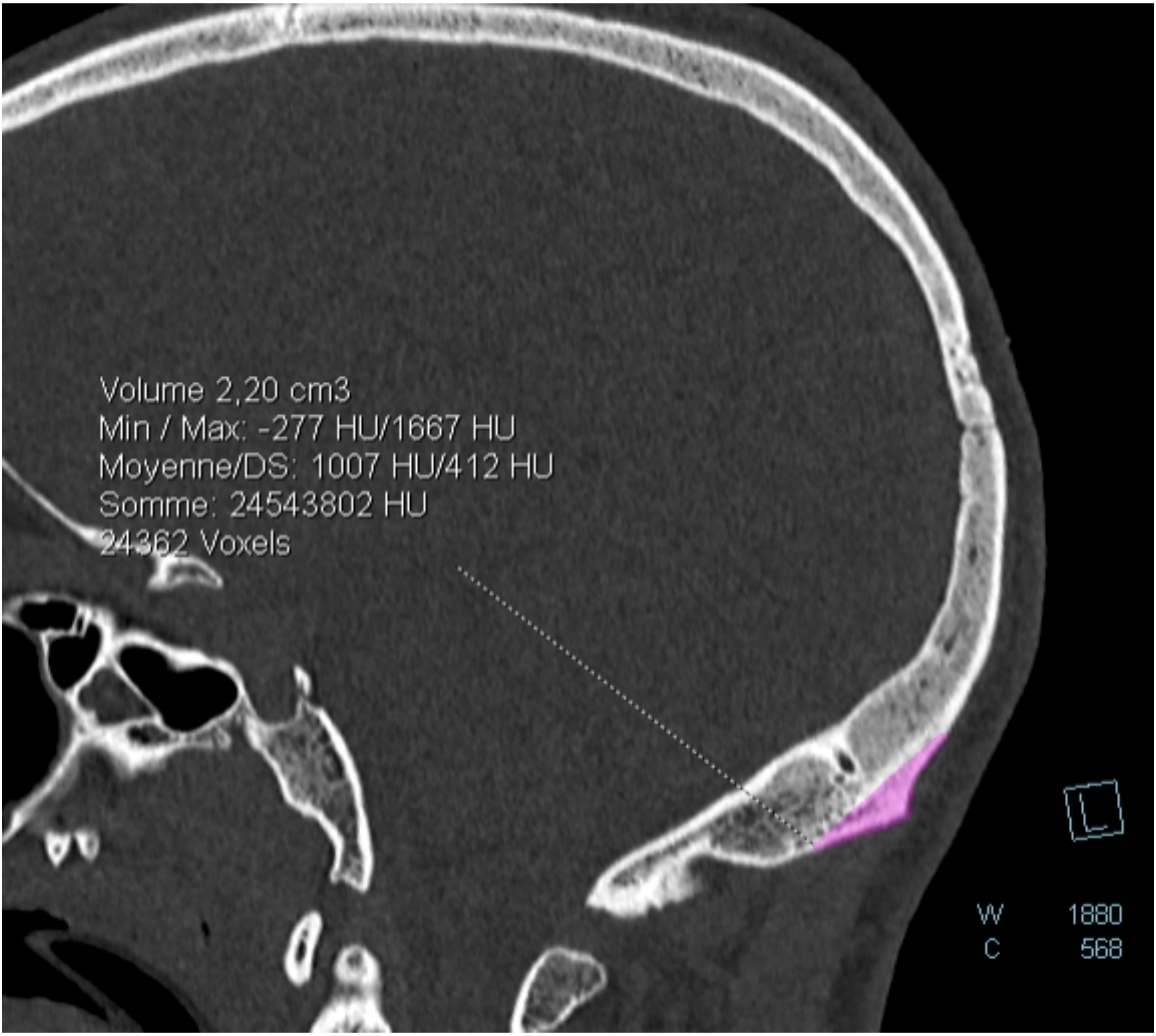

Volume 2,20 cm3
Min / Max: -277 HU/1667 HU
Moyenne/DS: 1007 HU/412 HU
Somme: 24543802 HU
24362 Voxels
W 1880
C 568

19m52

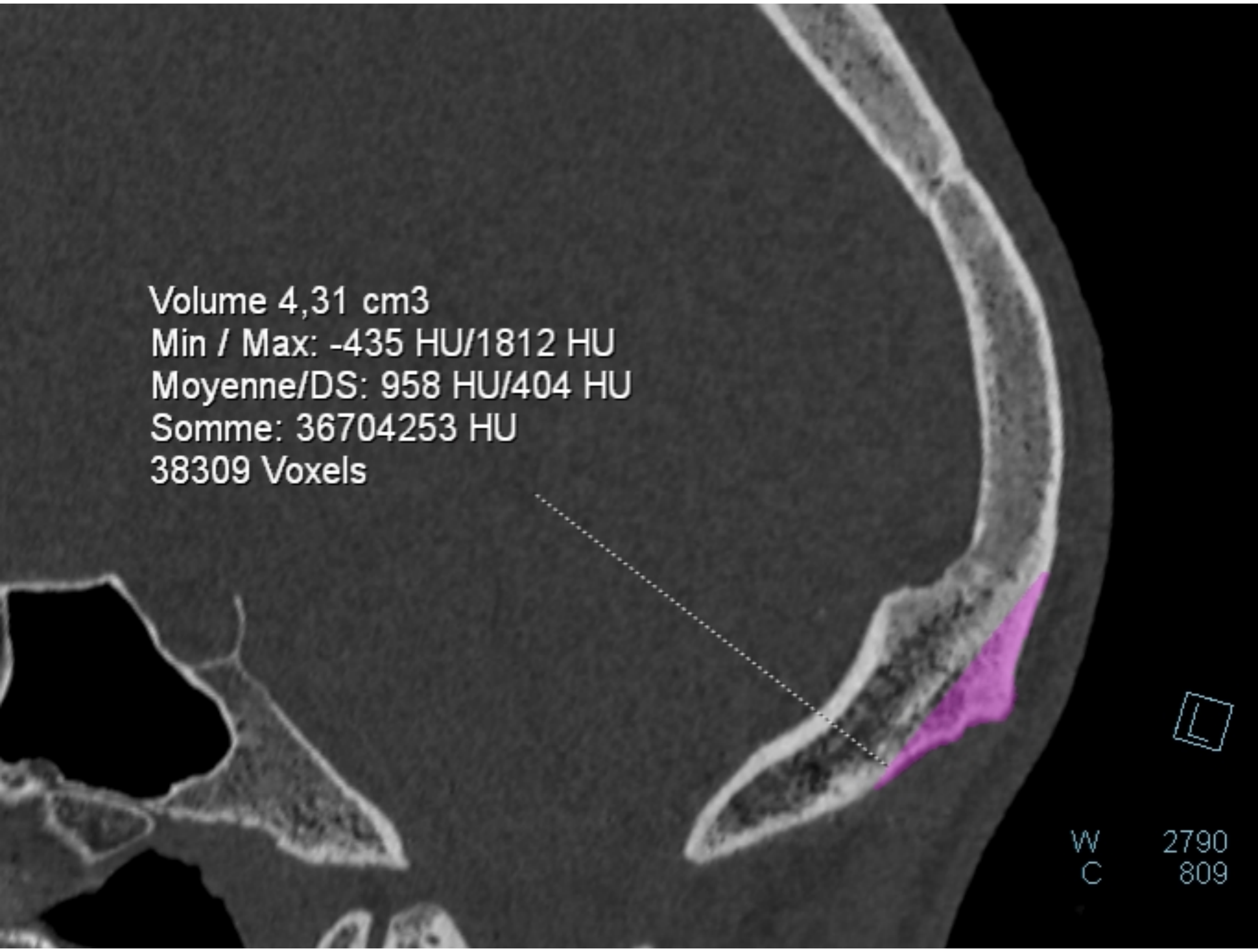
Volume 4,31 cm3
Min / Max: -435 HU/1812 HU
Moyenne/DS: 958 HU/404 HU
Somme: 36704253 HU
38309 Voxels
W 2790
C 809

19m53

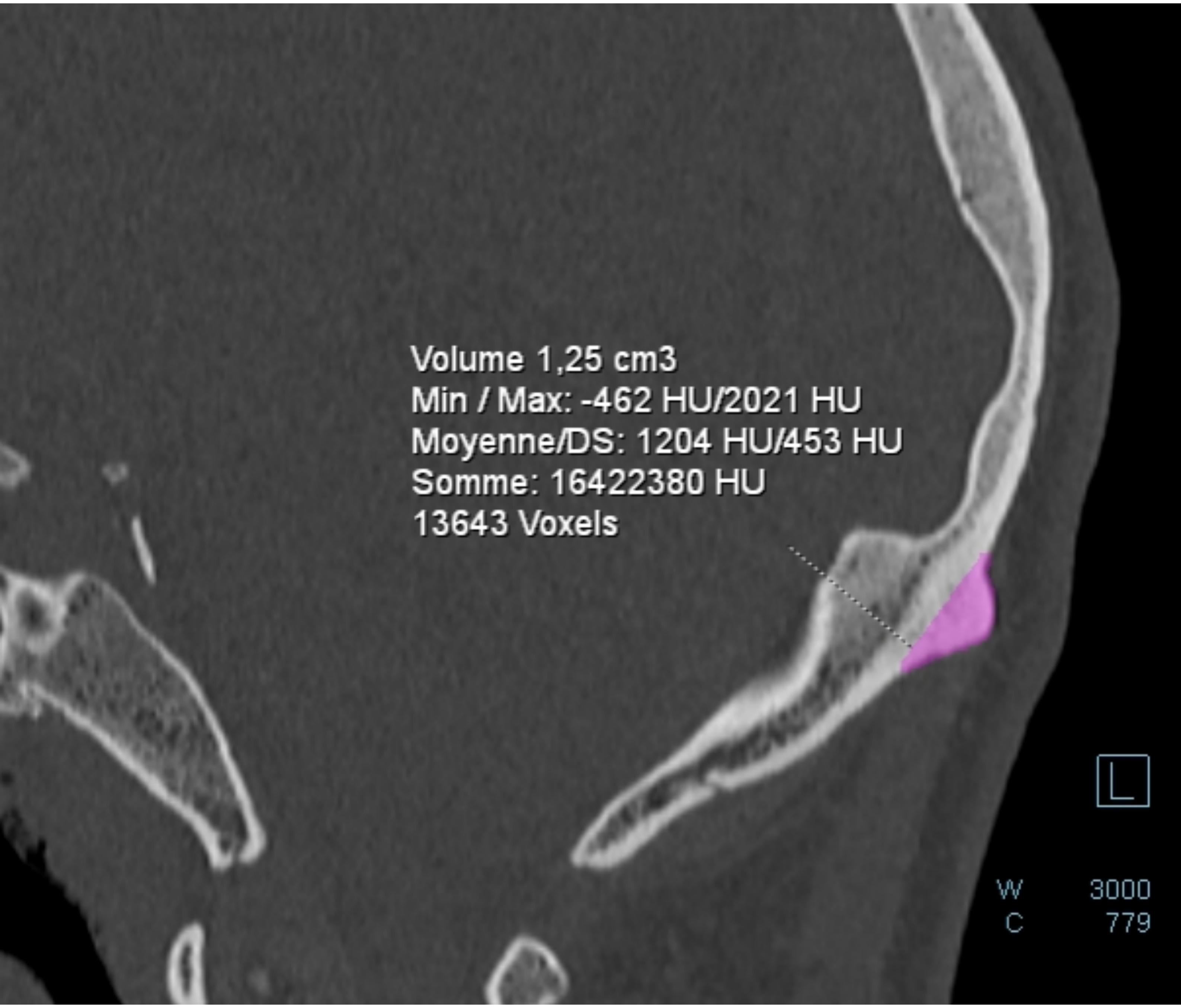
Volume 1,25 cm3
Min / Max: -462 HU/2021 HU
Moyenne/DS: 1204 HU/453 HU
Somme: 16422380 HU
13643 Voxels
L
W 3000
C 779

19m54

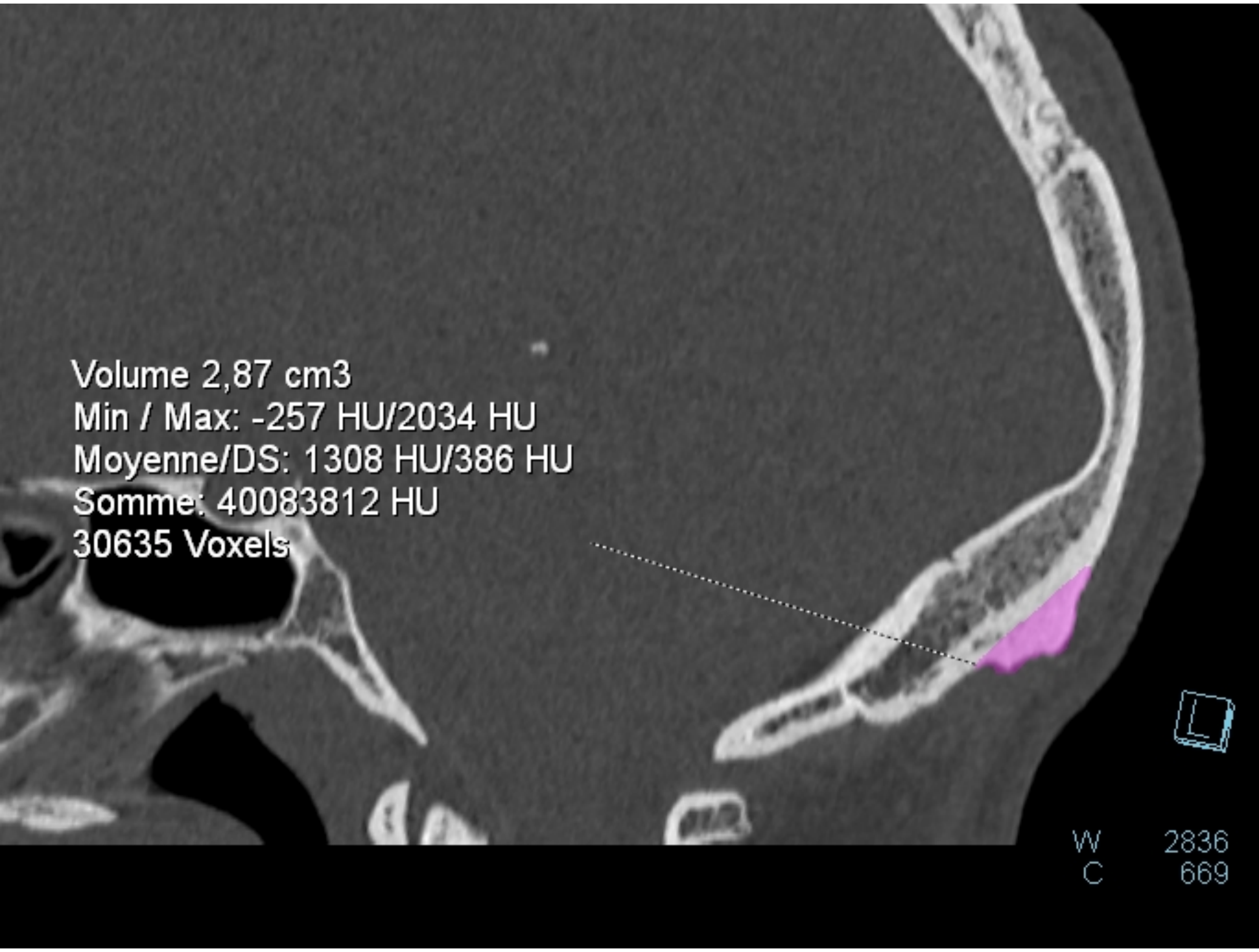

Volume 2,87 cm3
Min / Max: -257 HU/2034 HU
Moyenne/DS: 1308 HU/386 HU
Somme: 40083812 HU
30635 Voxels
W 2836
C 669

19m55

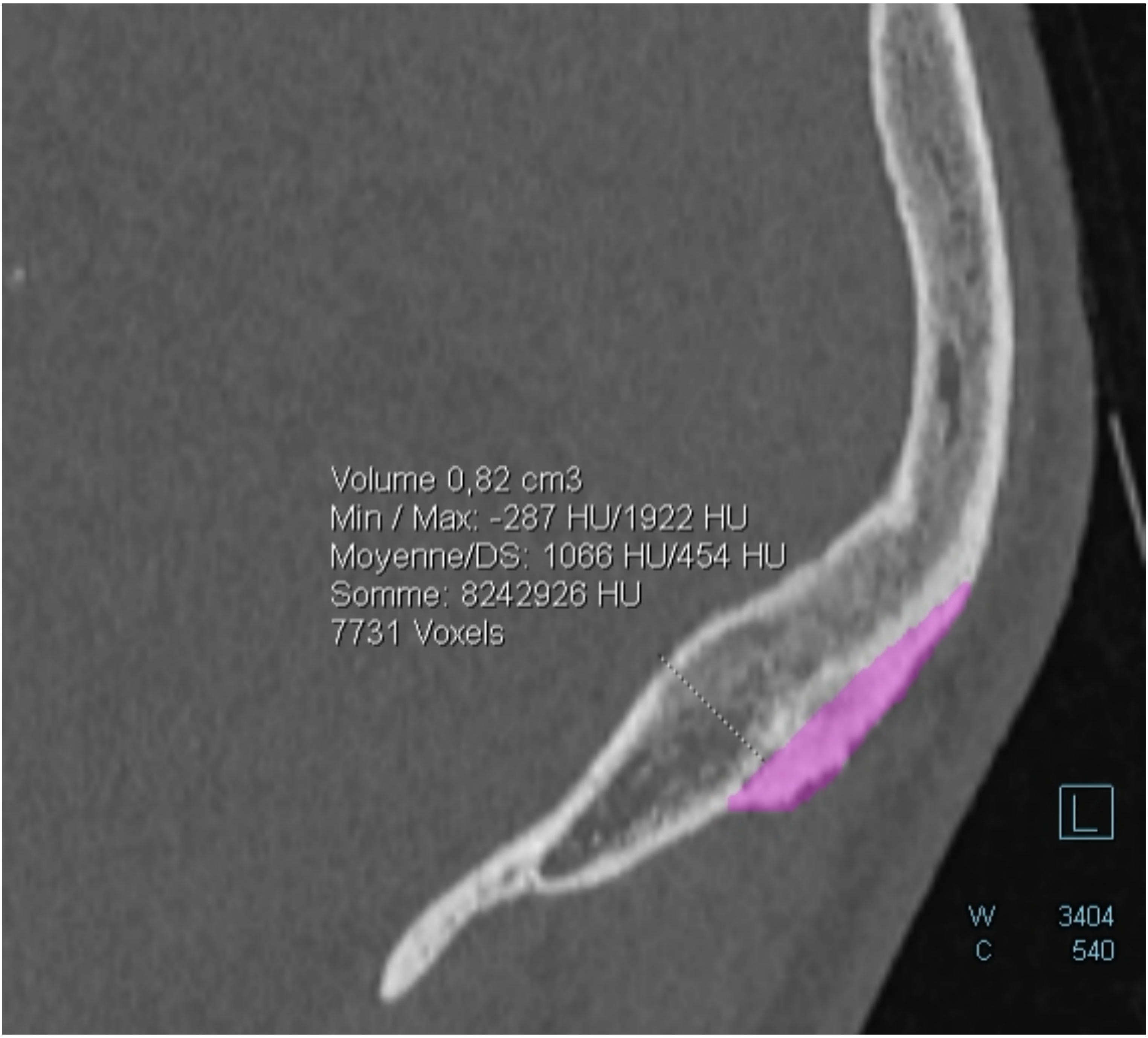
Volume 0,82 cm3
Min / Max: -287 HU/1922 HU
Moyenne/DS: 1066 HU/454 HU
Somme: 8242926 HU
7731 Voxels
L
W 3404
C 540

19m56

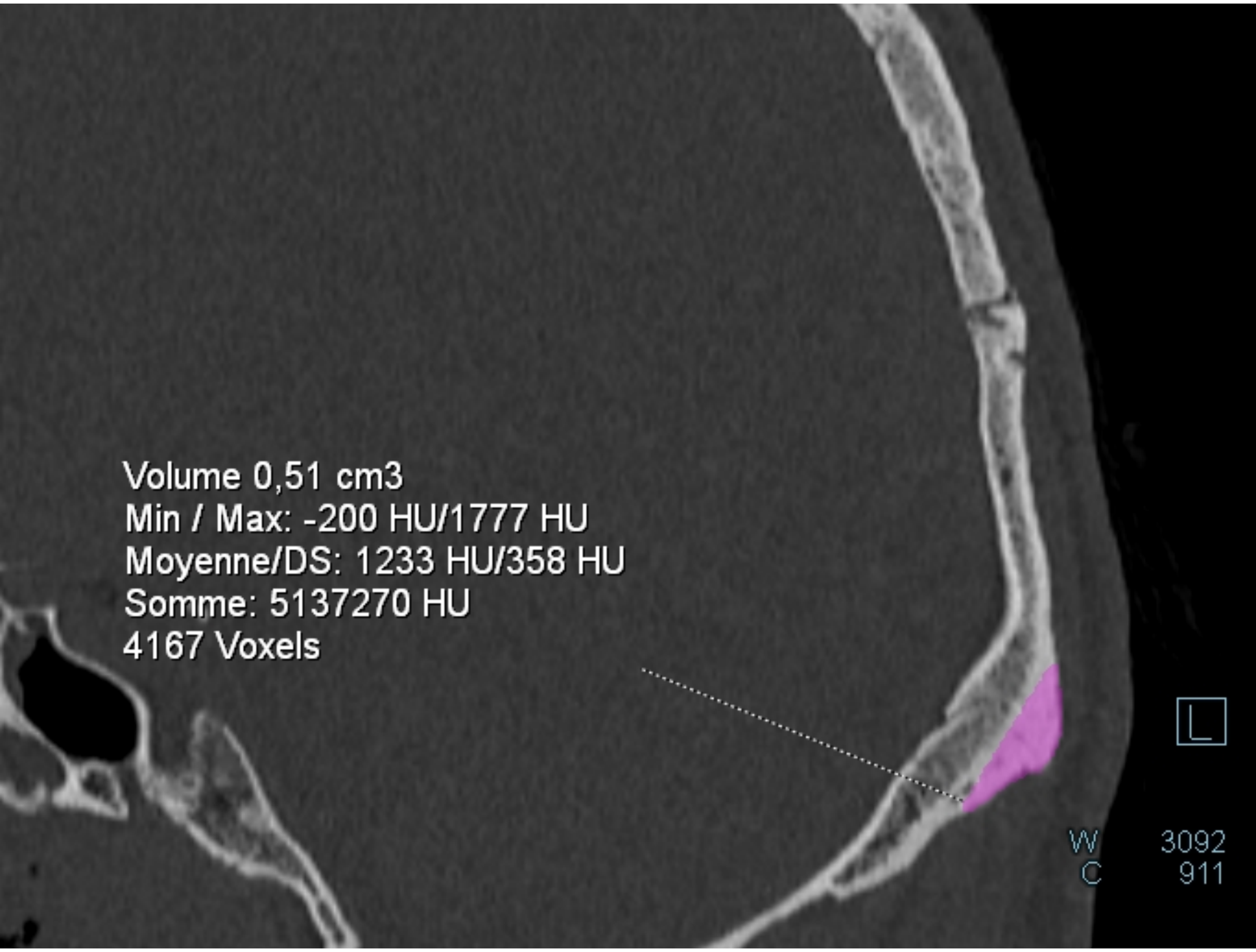
Volume 0,51 cm3
Min / Max: -200 HU/1777 HU
Moyenne/DS: 1233 HU/358 HU
Somme: 5137270 HU
4167 Voxels
L
W 3092
C 911

19m57

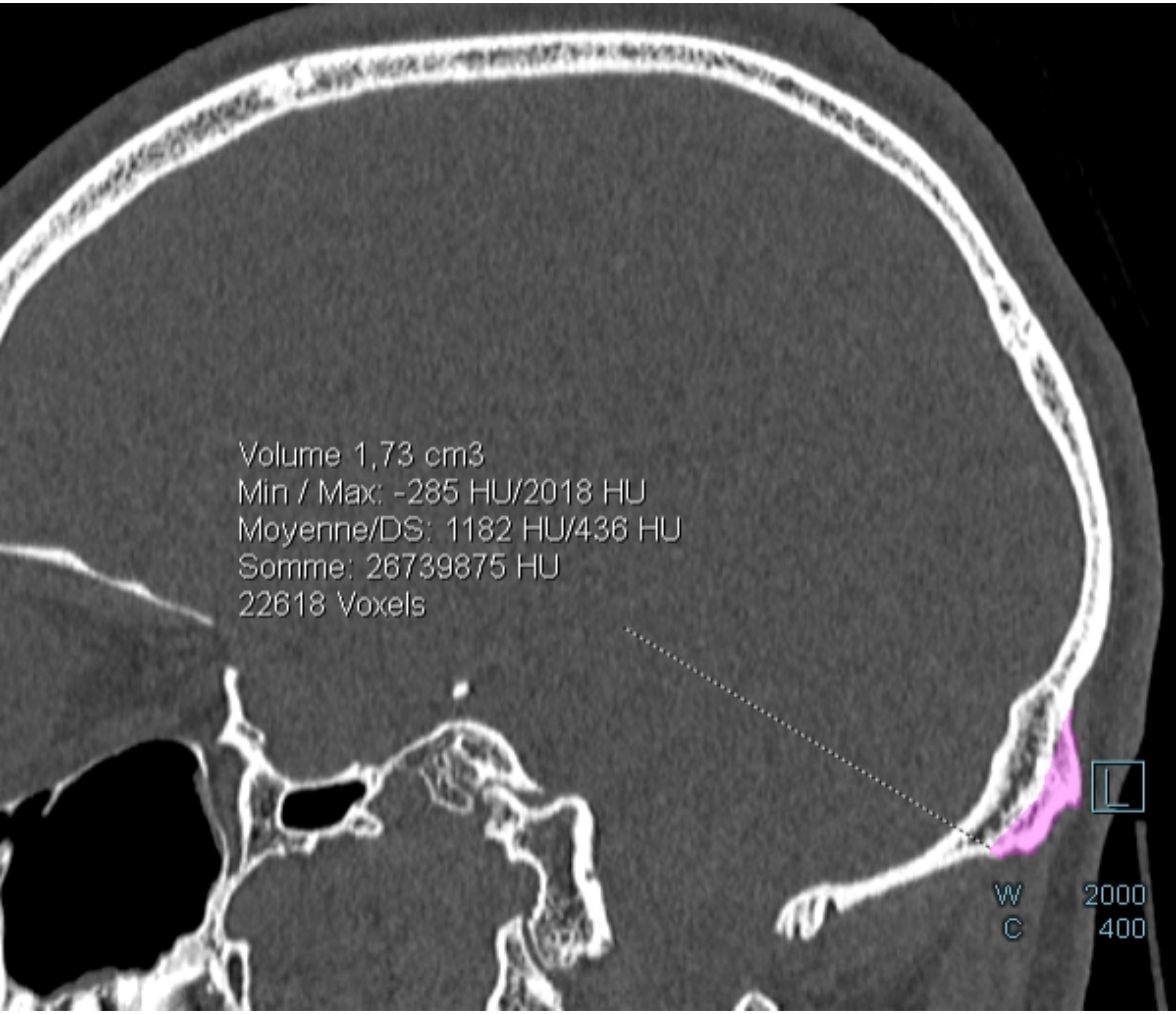
Volume 1,73 cm3
Min / Max: -285 HU/2018 HU
Moyenne/DS: 1182 HU/436 HU
Somme: 26739875 HU
22618 Voxels
L
W 2000
C 400

19m58

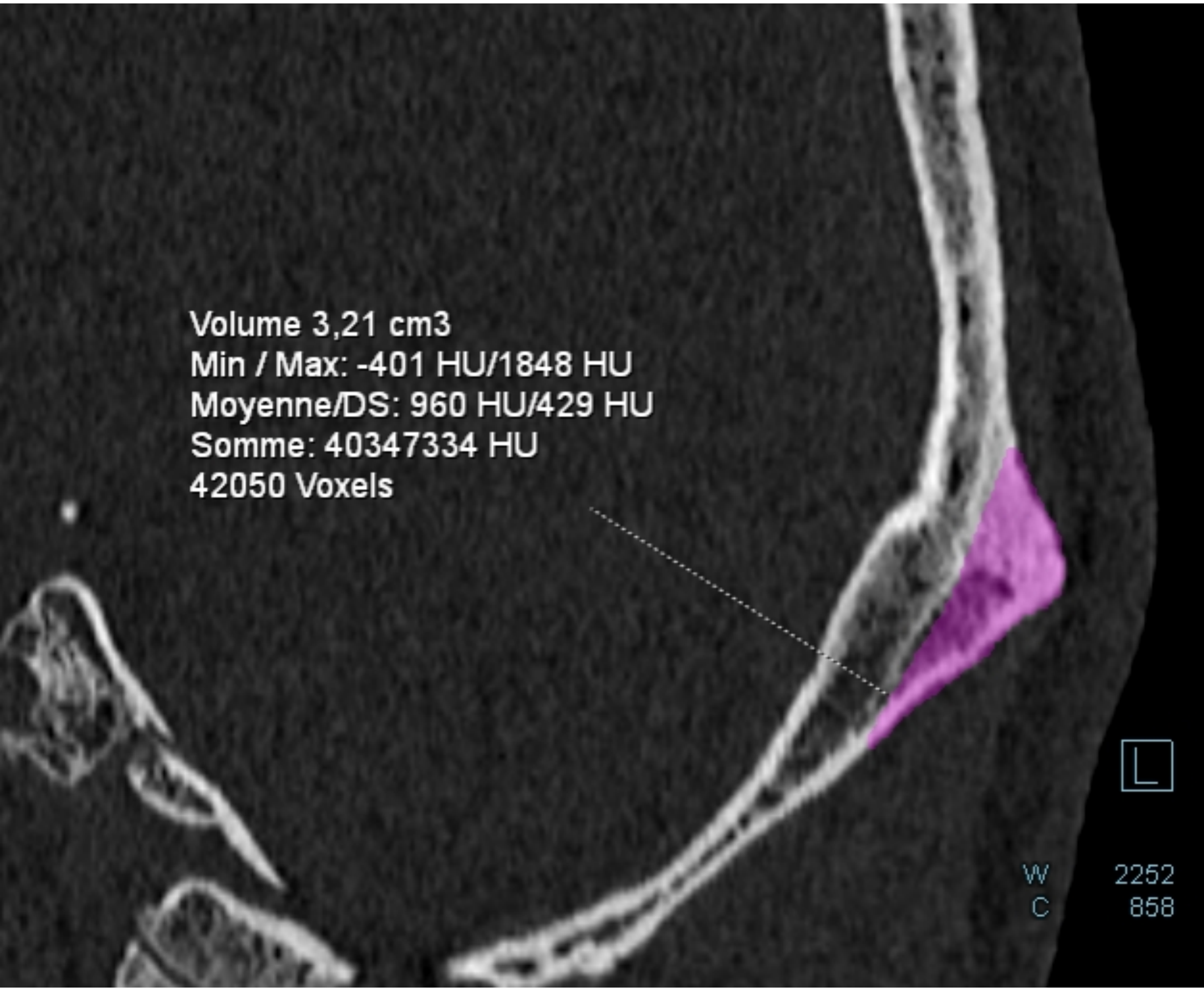

Volume 3,21 cm3
Min / Max: -401 HU/1848 HU
Moyenne/DS: 960 HU/429 HU
Somme: 40347334 HU
42050 Voxels
L
W 2252
C 858

19m59

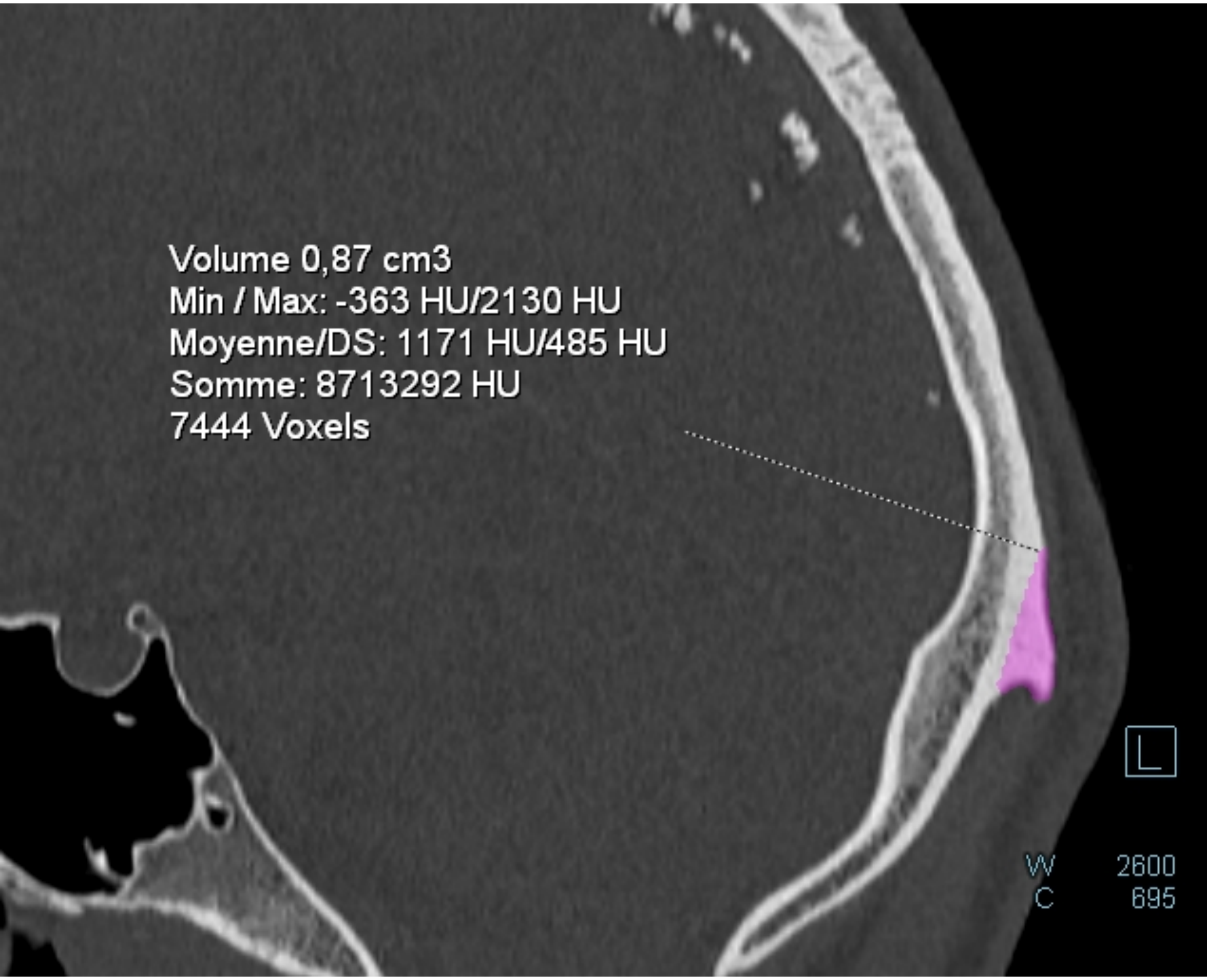
Volume 0,87 cm3
Min / Max: -363 HU/2130 HU
Moyenne/DS: 1171 HU/485 HU
Somme: 8713292 HU
7444 Voxels
L
W 2600
C 695

19m60

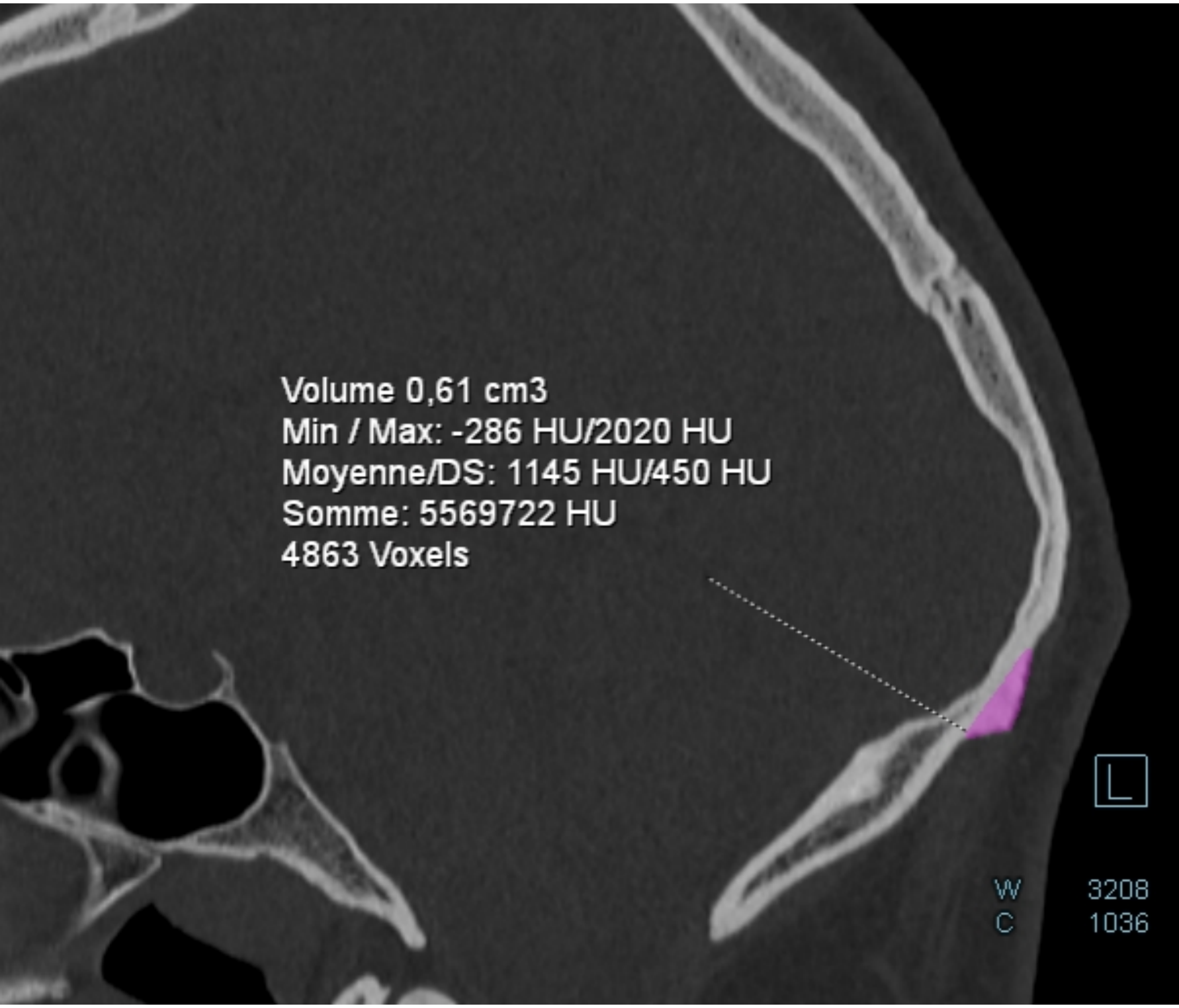

Volume 0,61 cm3
Min / Max: -286 HU/2020 HU
Moyenne/DS: 1145 HU/450 HU
Somme: 5569722 HU
4863 Voxels
L
W 3208
C 1036

19m61

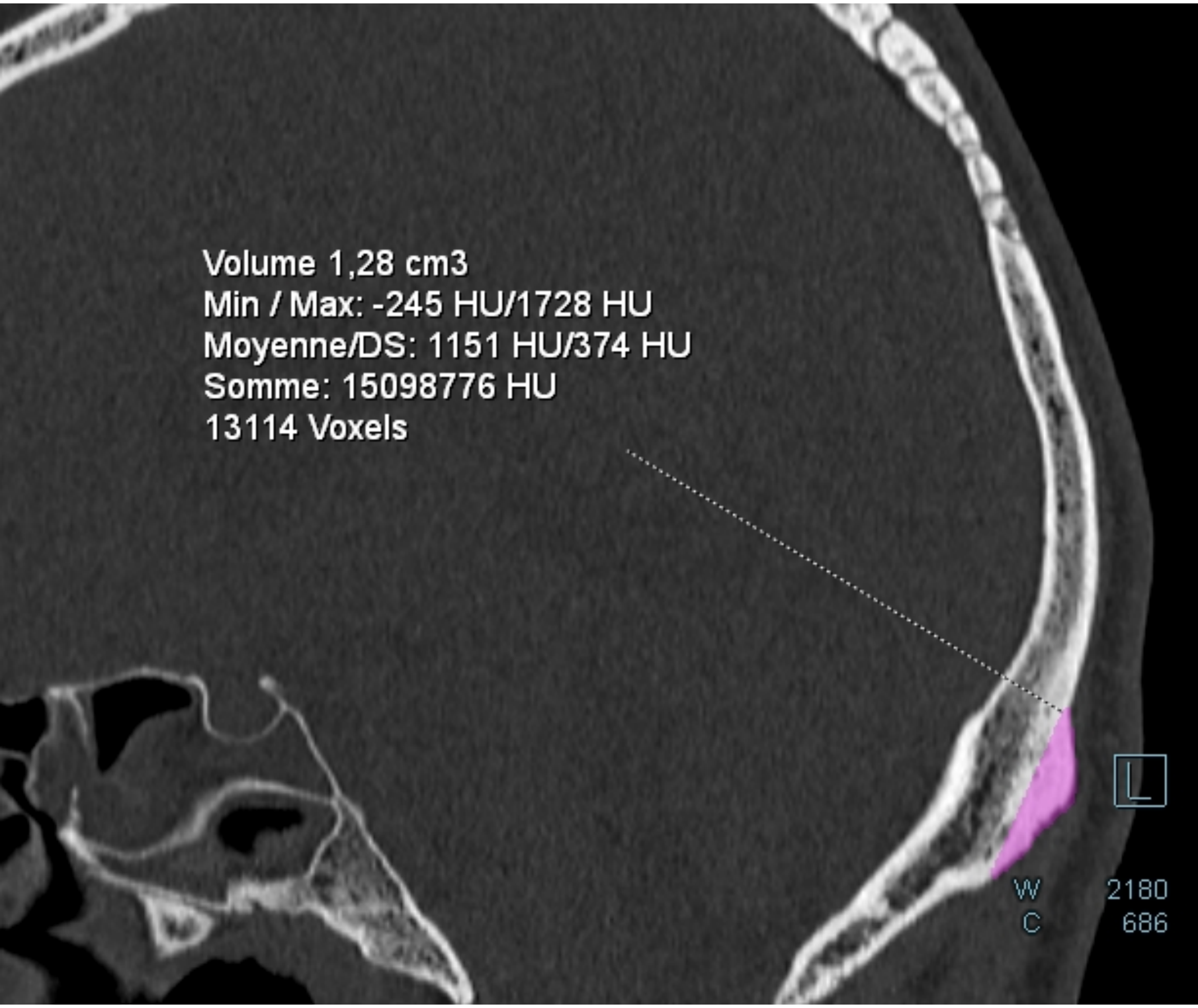
Volume 1,28 cm3
Min / Max: -245 HU/1728 HU
Moyenne/DS: 1151 HU/374 HU
Somme: 15098776 HU
13114 Voxels
L
W 2180
C 686

19m62

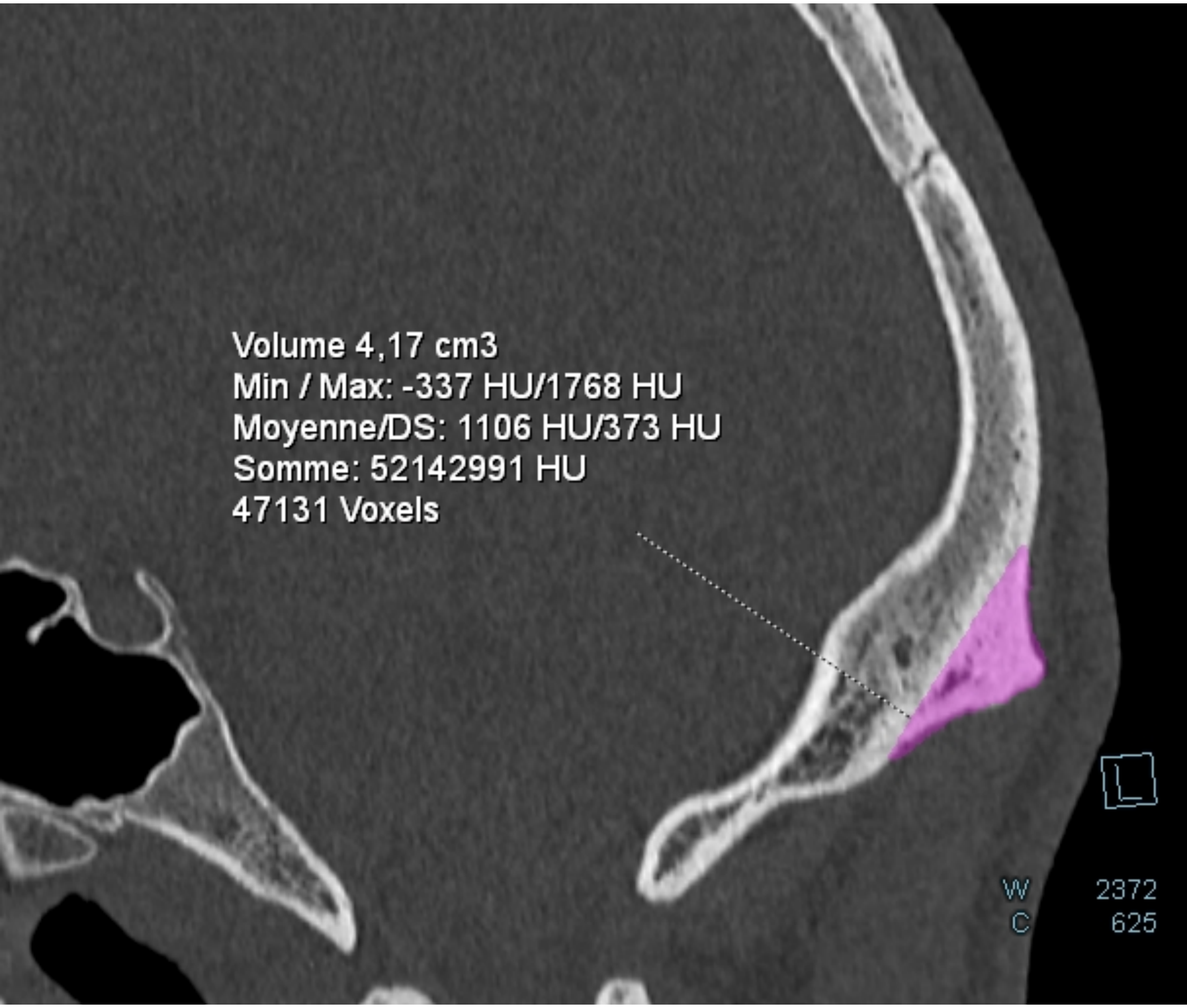

Volume 4,17 cm3
Min / Max: -337 HU/1768 HU
Moyenne/DS: 1106 HU/373 HU
Somme: 52142991 HU
47131 Voxels
W 2372
C 625

19m63

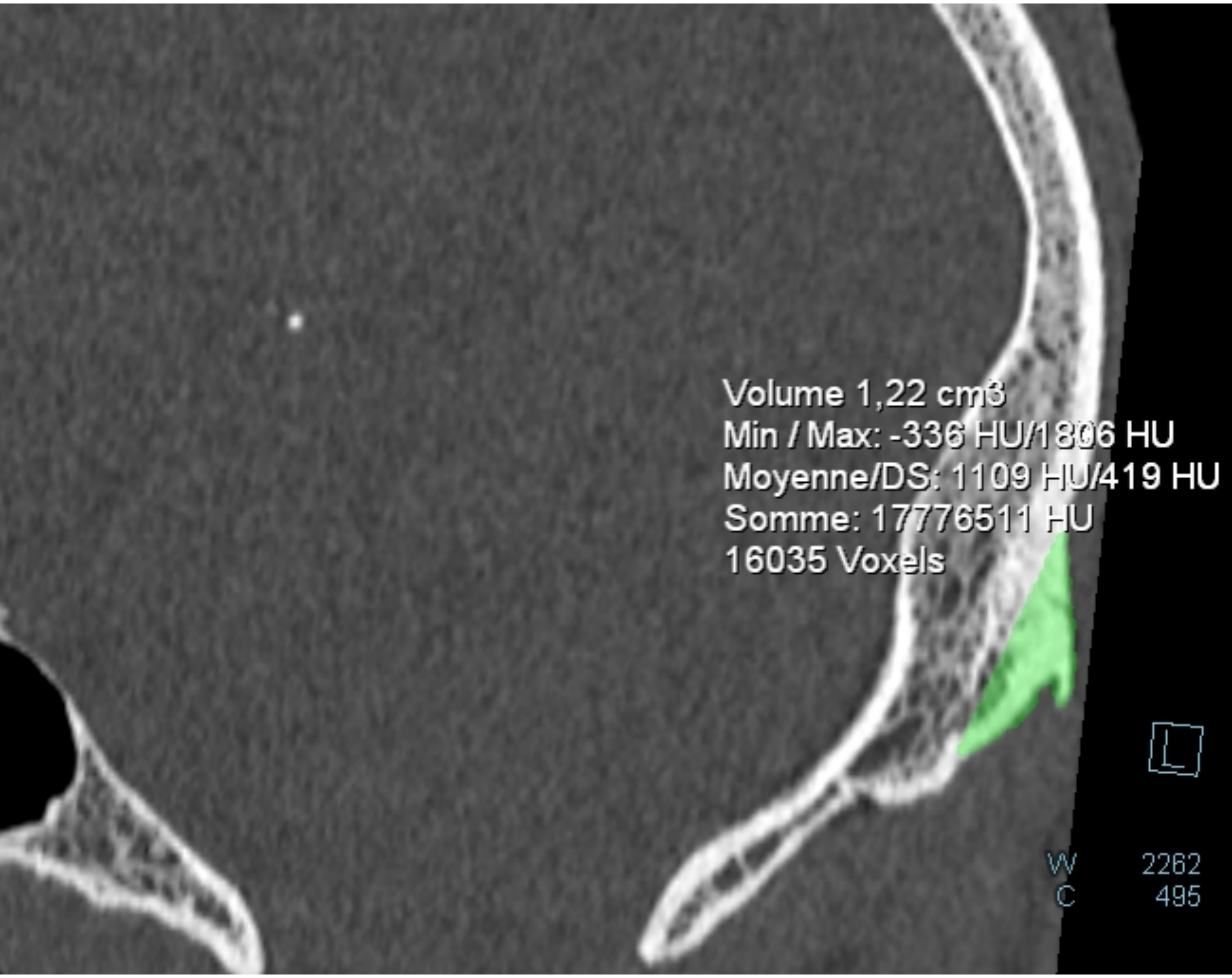
Volume 1,22 cm3
Min / Max: -336 HU/1806 HU
Moyenne/DS: 1109 HU/419 HU
Somme: 17776511 HU
16035 Voxels
W 2262
C 495

19m64

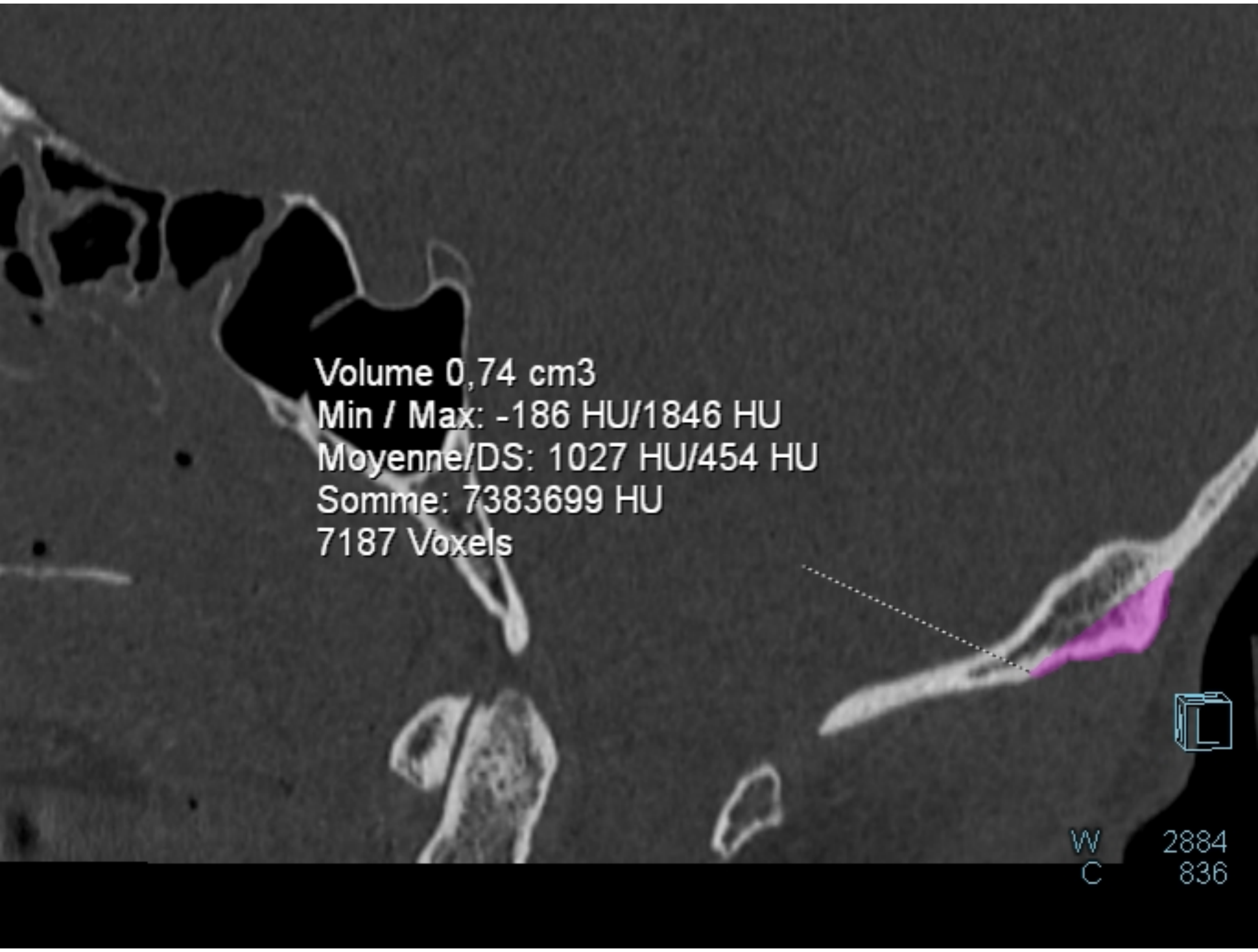
Volume 0,74 cm3
Min / Max: -186 HU/1846 HU
Moyenne/DS: 1027 HU/454 HU
Somme: 7383699 HU
7187 Voxels
W 2884
C 836

19m65

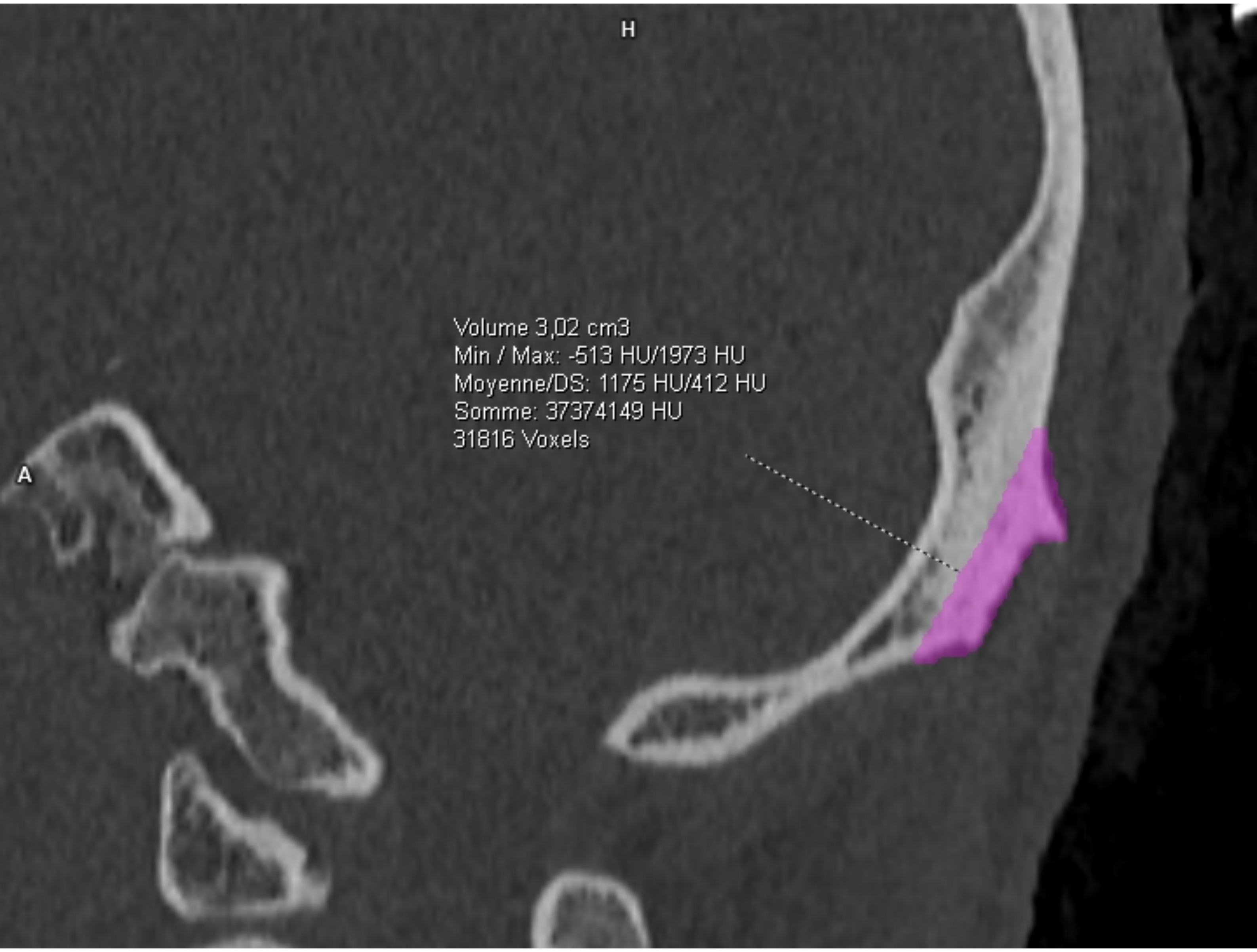
H
A
Volume 3,02 cm3
Min / Max: -513 HU/1973 HU
Moyenne/DS: 1175 HU/412 HU
Somme: 37374149 HU
31816 Voxels

19m66

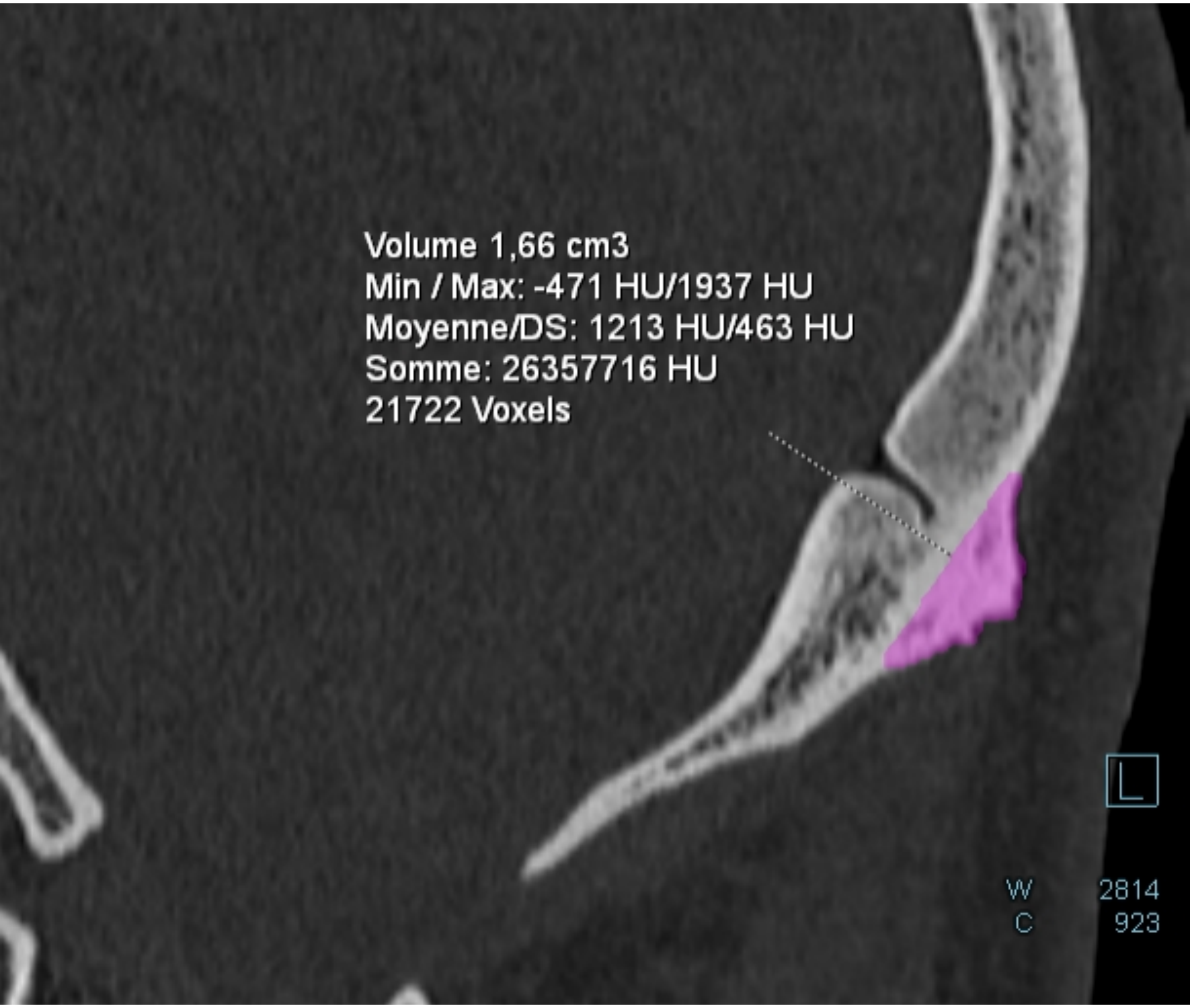

Volume 1,66 cm3
Min / Max: -471 HU/1937 HU
Moyenne/DS: 1213 HU/463 HU
Somme: 26357716 HU
21722 Voxels
L
W 2814
C 923

19m67

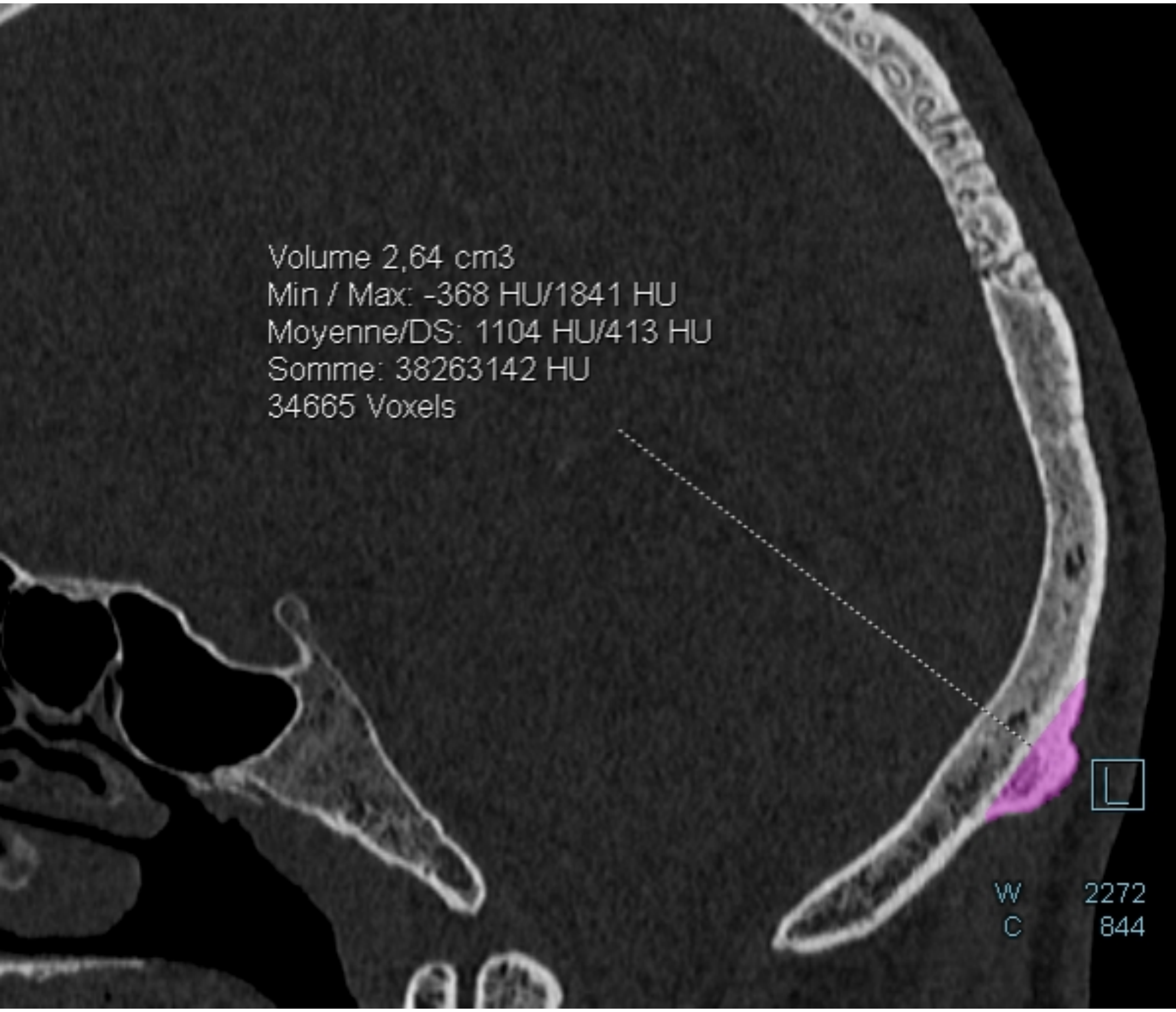
Volume 2,64 cm3
Min / Max: -368 HU/1841 HU
Moyenne/DS: 1104 HU/413 HU
Somme: 38263142 HU
34665 Voxels
L
W 2272
C 844

19m68

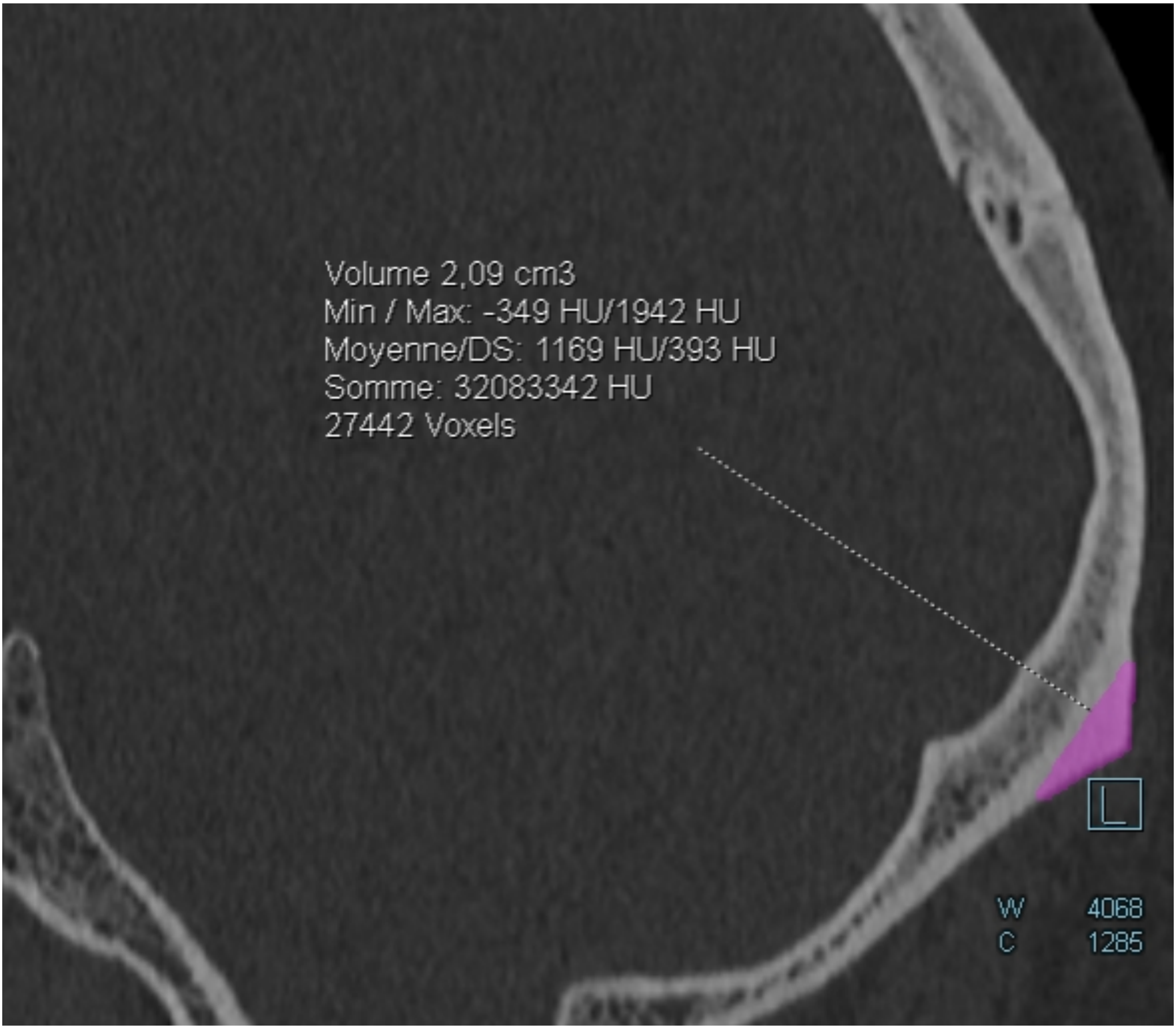
Volume 2,09 cm3
Min / Max: -349 HU/1942 HU
Moyenne/DS: 1169 HU/393 HU
Somme: 32083342 HU
27442 Voxels
L
W 4068
C 1285

19m69

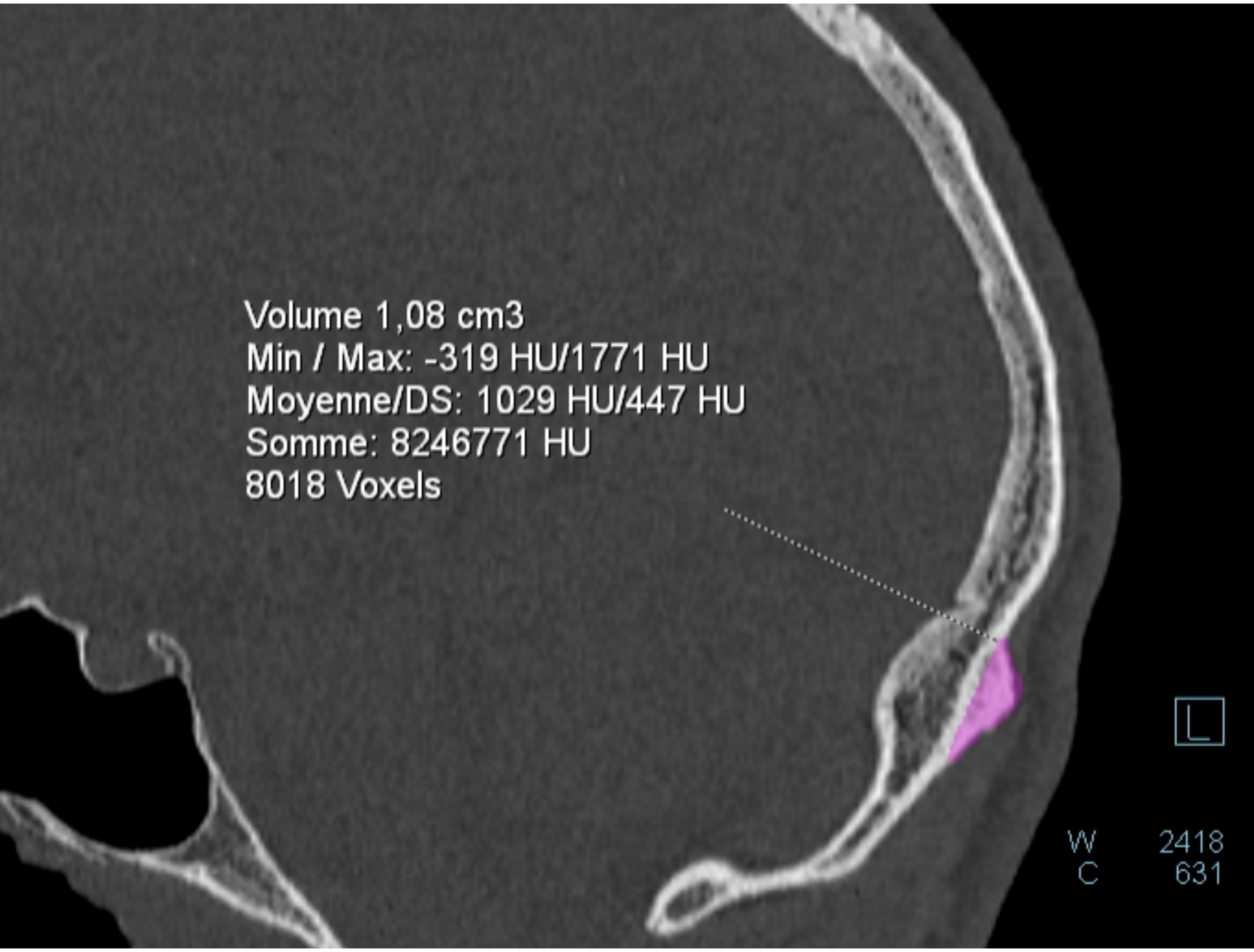
Volume 1,08 cm3
Min / Max: -319 HU/1771 HU
Moyenne/DS: 1029 HU/447 HU
Somme: 8246771 HU
8018 Voxels
L
W 2418
C 631

19m70

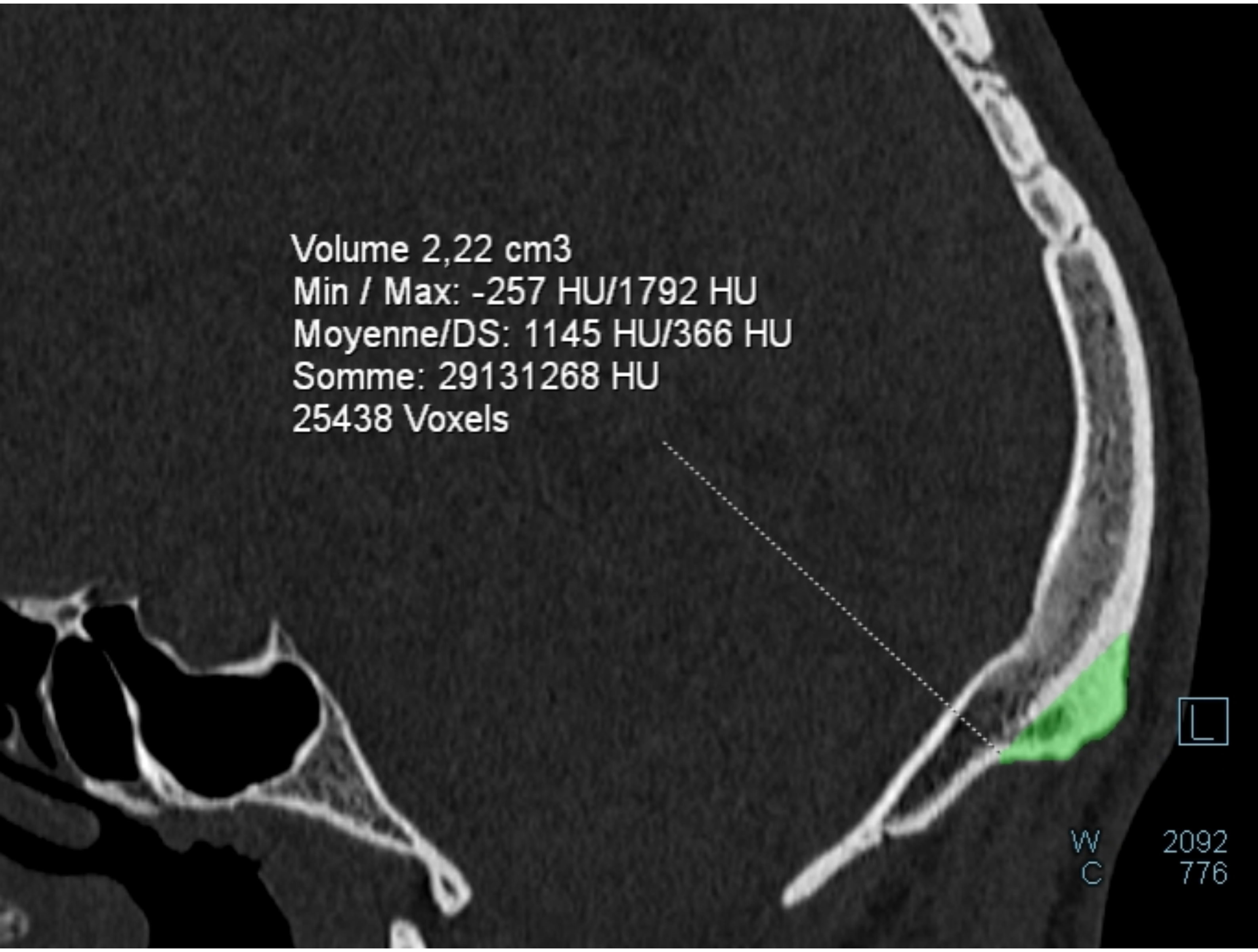
Volume 2,22 cm3
Min / Max: -257 HU/1792 HU
Moyenne/DS: 1145 HU/366 HU
Somme: 29131268 HU
25438 Voxels
L
W 2092
C 776

19m71

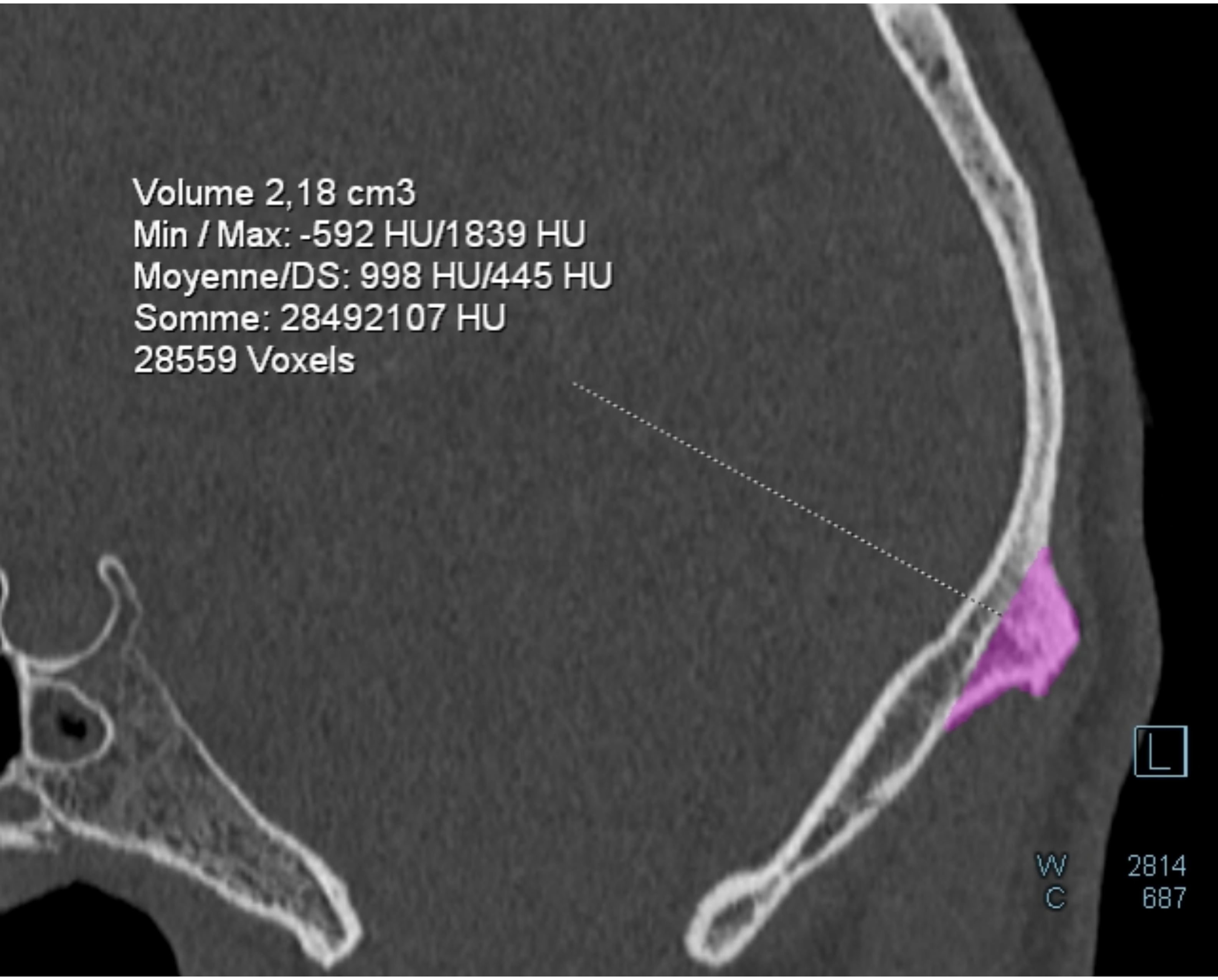
Volume 2,18 cm3
Min / Max: -592 HU/1839 HU
Moyenne/DS: 998 HU/445 HU
Somme: 28492107 HU
28559 Voxels
L
W 2814
C 687

19m72

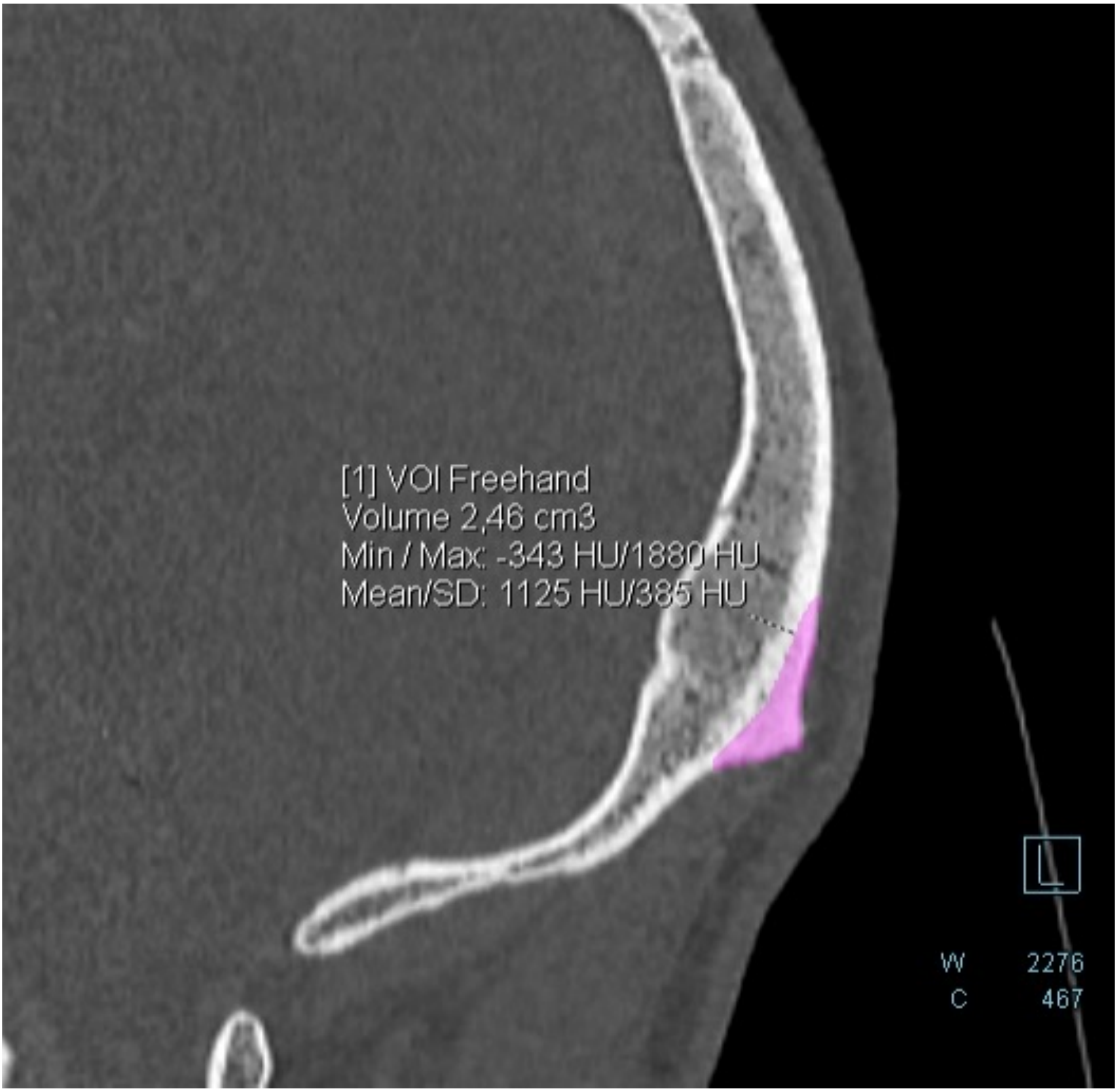
[1] VOI Freehand
Volume 2,46 cm3
Min / Max: -343 HU/1880 HU
Mean/SD: 1125 HU/385 HU
L
W 2276
C 467

19m73

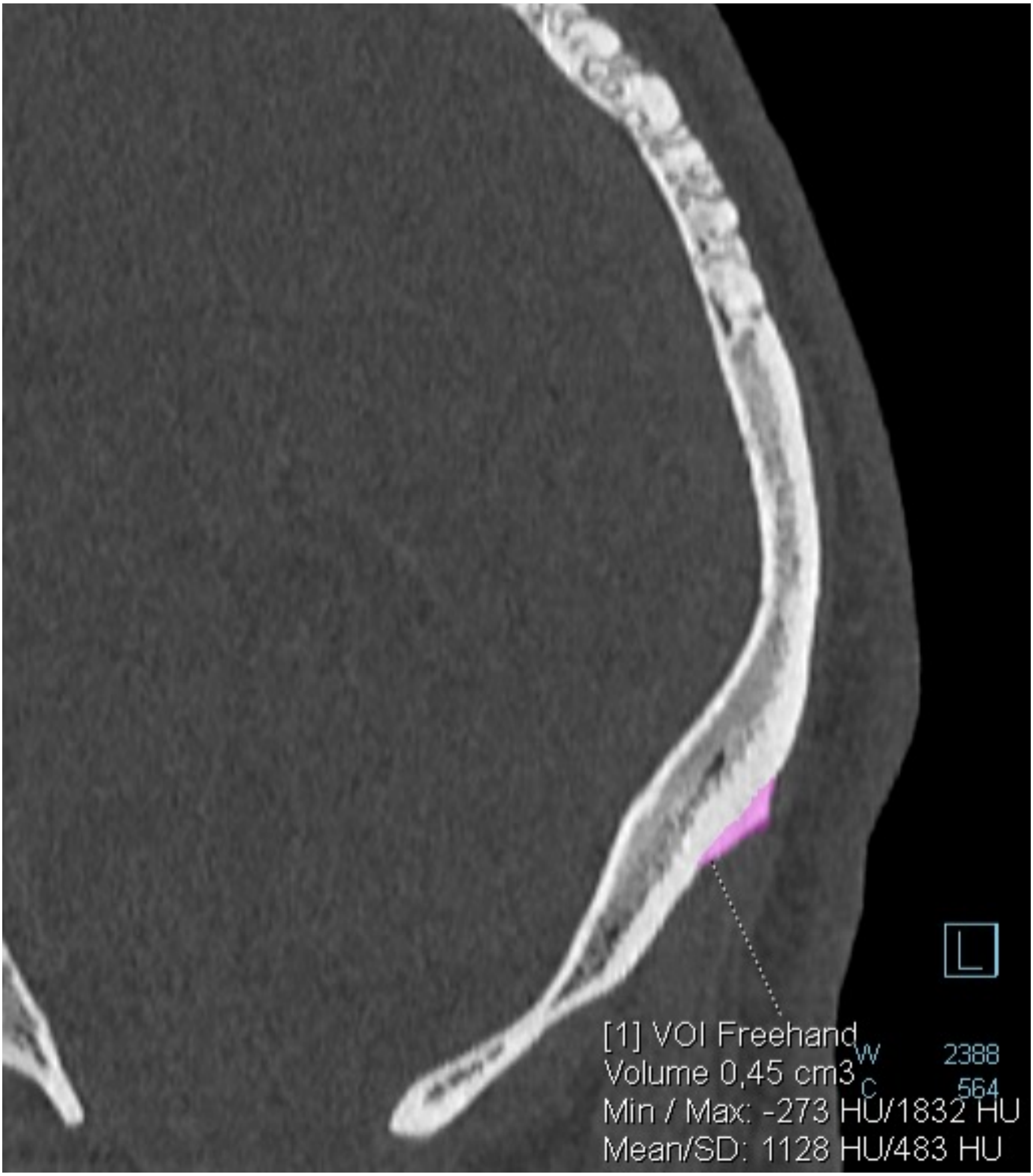
[1] VOI Freehand
Volume 0,45 cm3
Min / Max: -273 HU/1832 HU
Mean/SD: 1128 HU/483 HU
W 2388
C 564
L

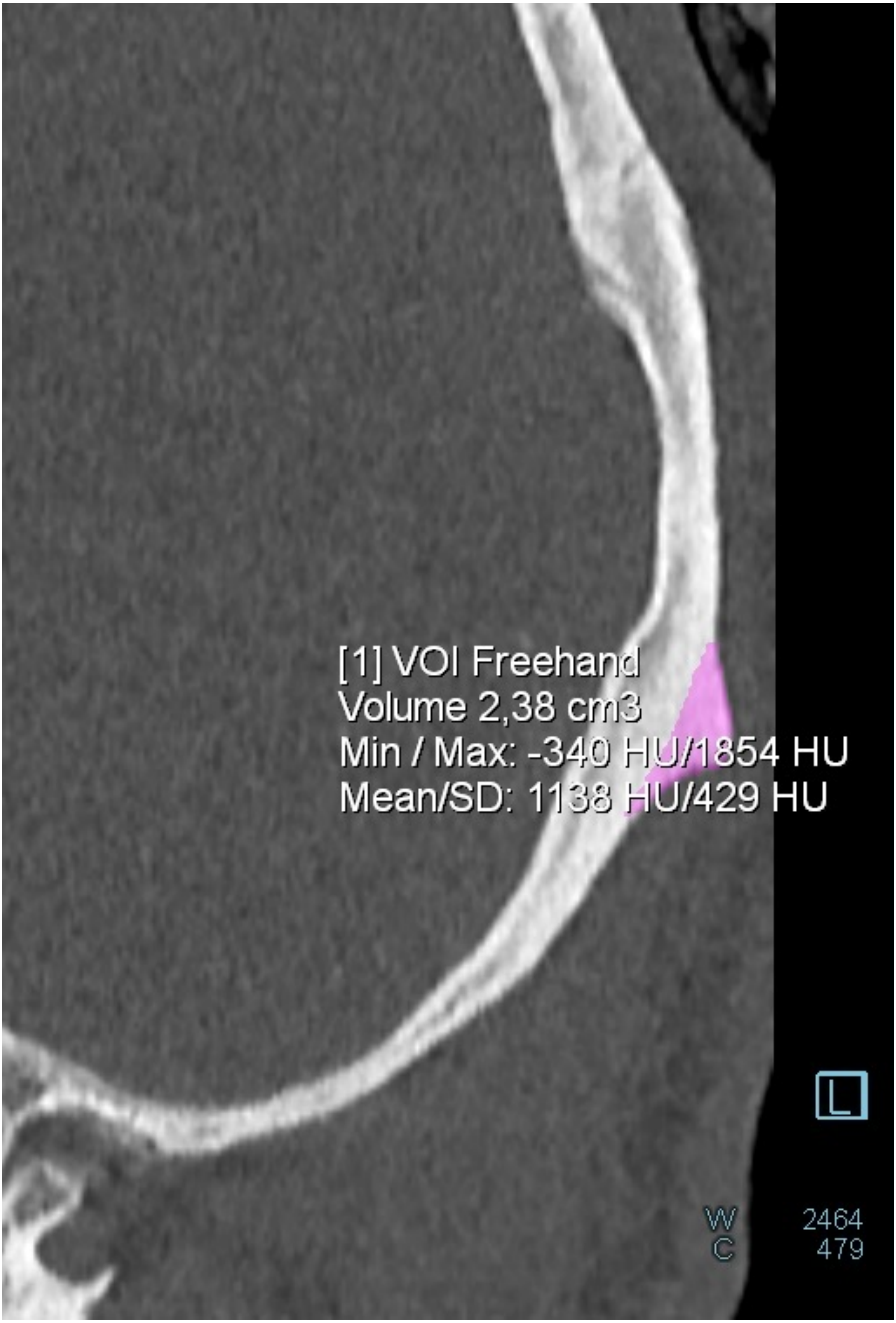
[1] VOI Freehand
Volume 2,38 cm3
Min / Max: -340 HU/1854 HU
Mean/SD: 1138 HU/429 HU
L
W 2464
C 479

19m75

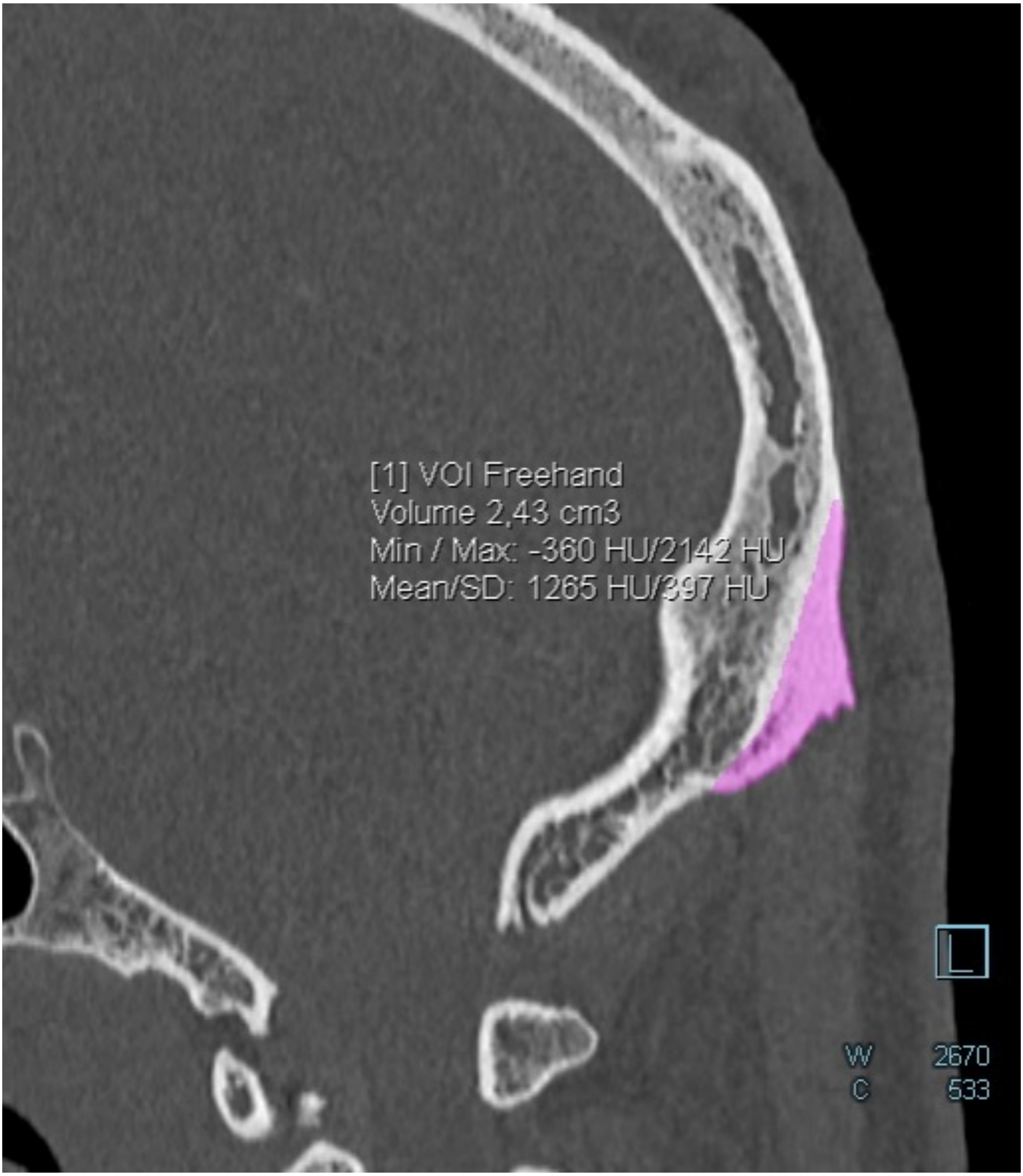

[1] VOI Freehand
Volume 2,43 cm3
Min / Max: -360 HU/2142 HU
Mean/SD: 1265 HU/397 HU
L
W 2670
C 533

19m76

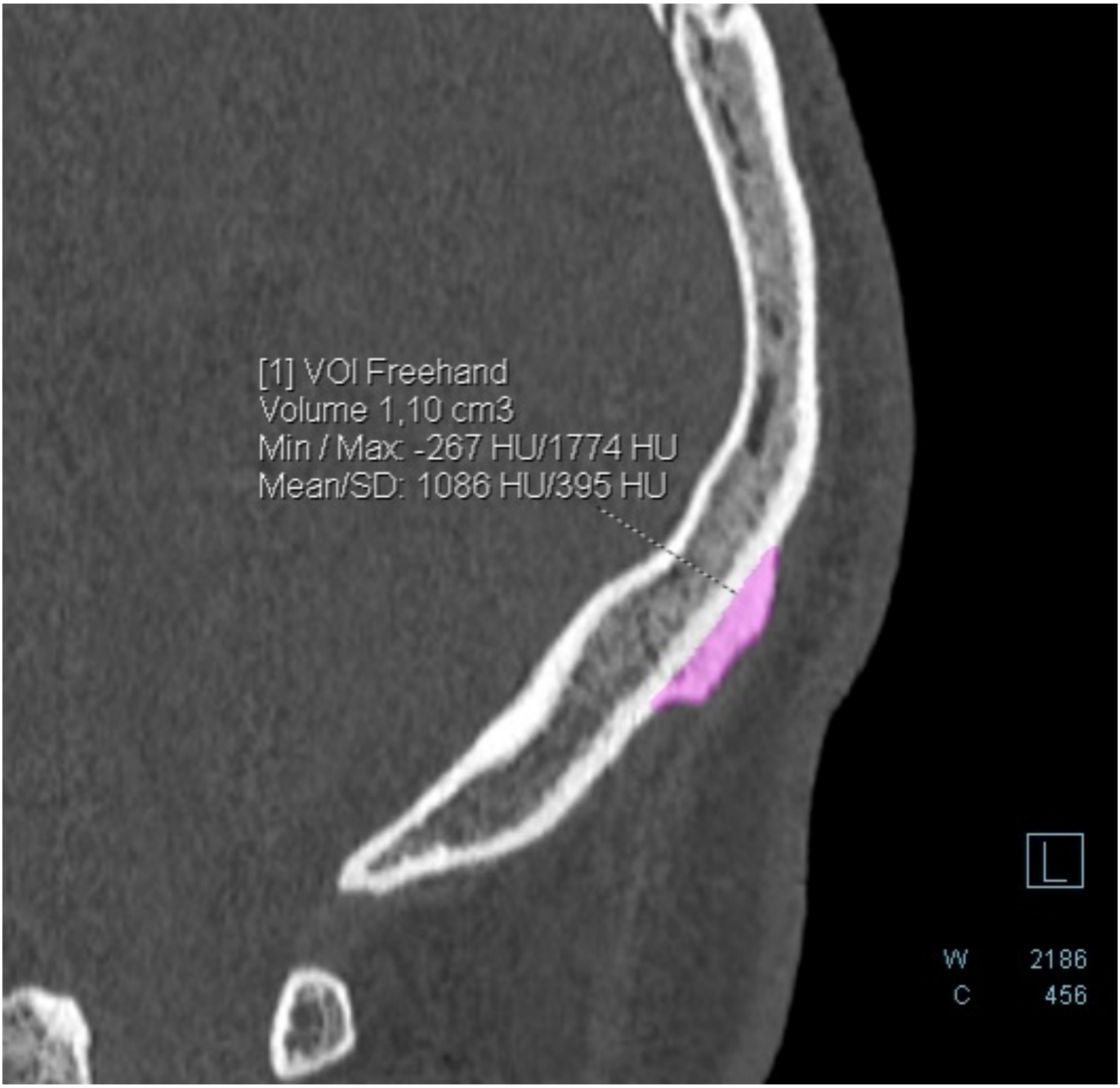

[1] VOI Freehand
Volume 1,10 cm3
Min / Max: -267 HU/1774 HU
Mean/SD: 1086 HU/395 HU
L
W 2186
C 456

19m77

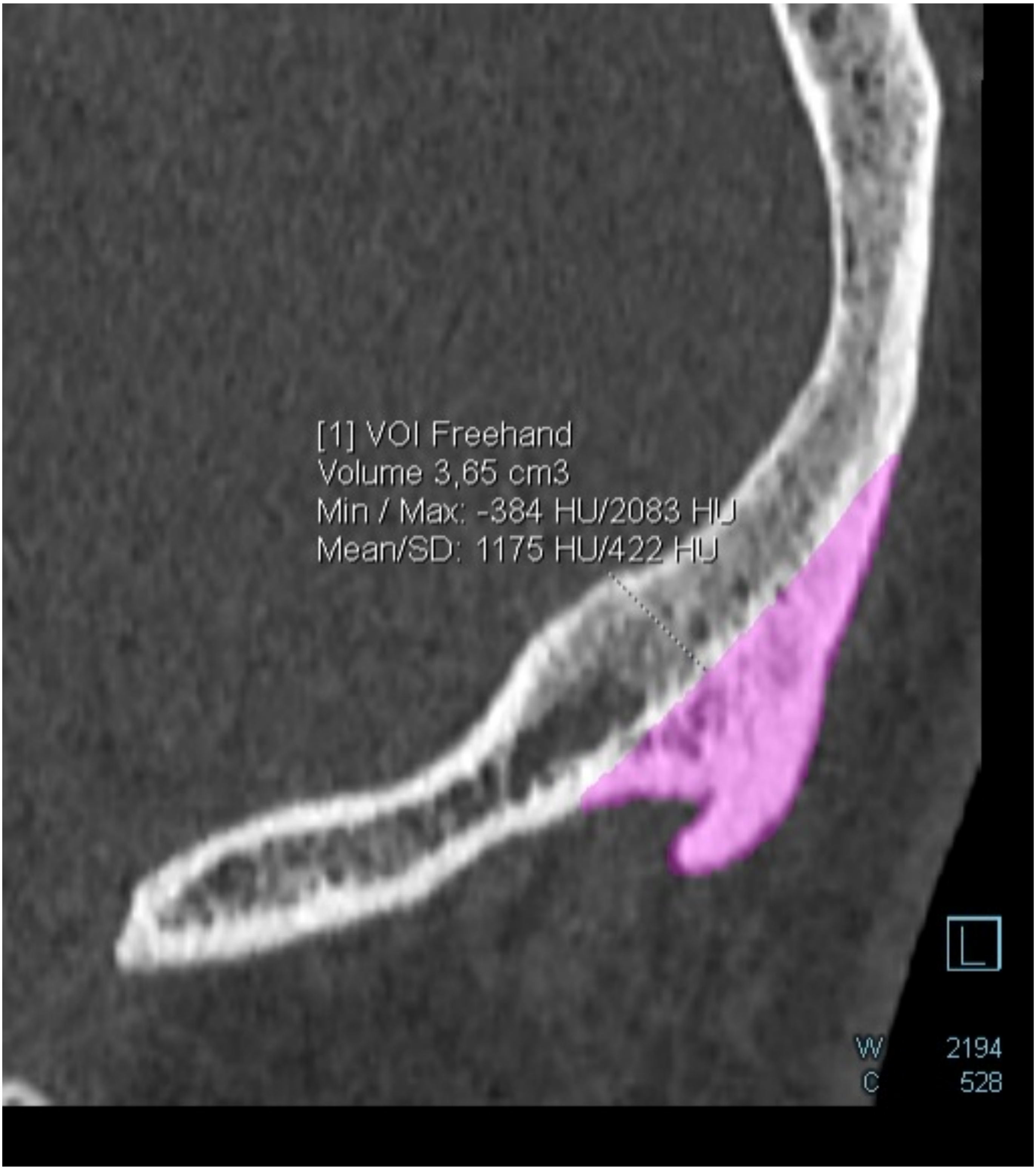
[1] VOI Freehand
Volume 3,65 cm3
Min / Max: -384 HU/2083 HU
Mean/SD: 1175 HU/422 HU
L
W 2194
C 528

19m78

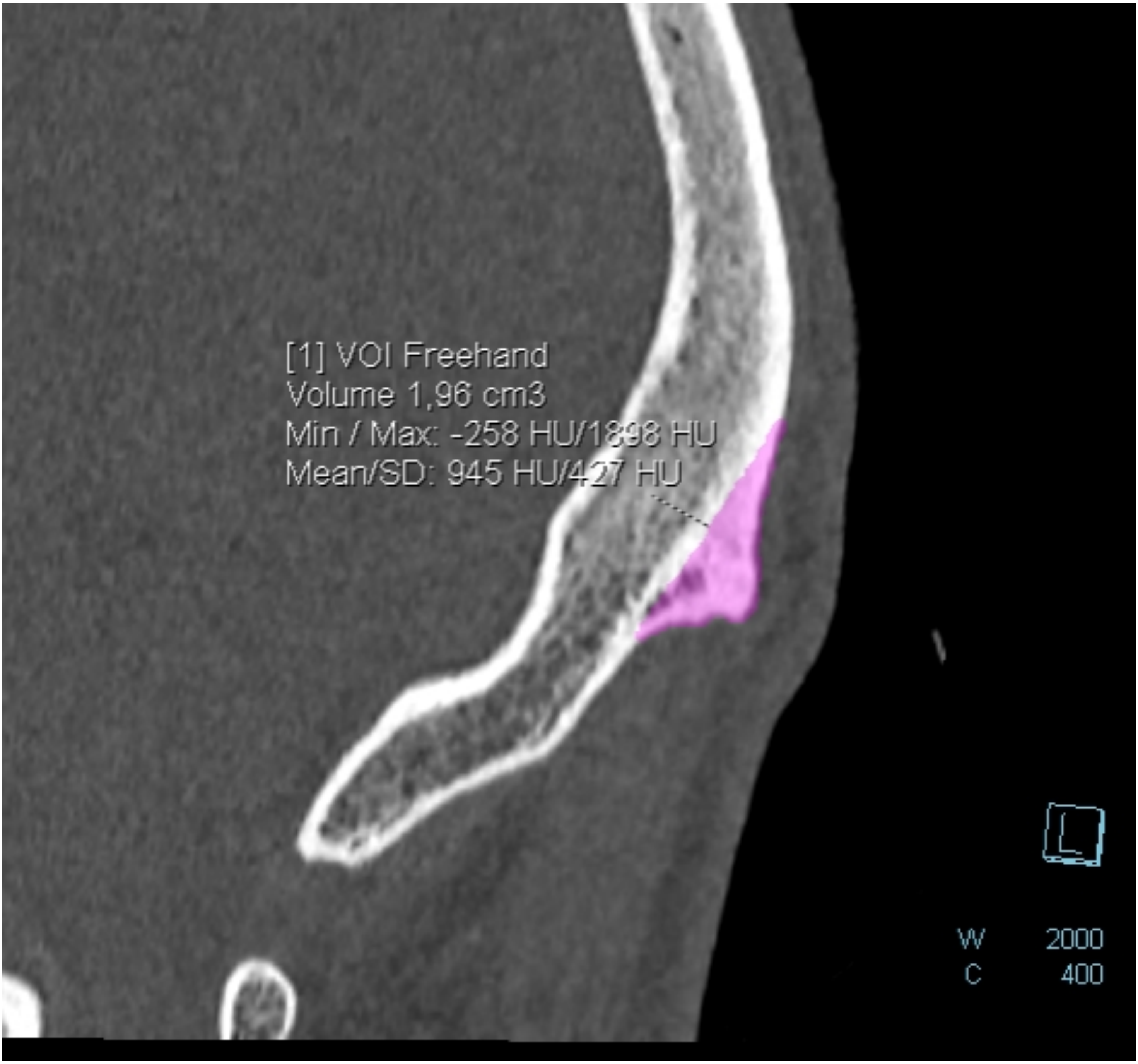
[1] VOI Freehand
Volume 1,96 cm3
Min / Max: -258 HU/1898 HU
Mean/SD: 945 HU/427 HU
W 2000
C 400

19m79

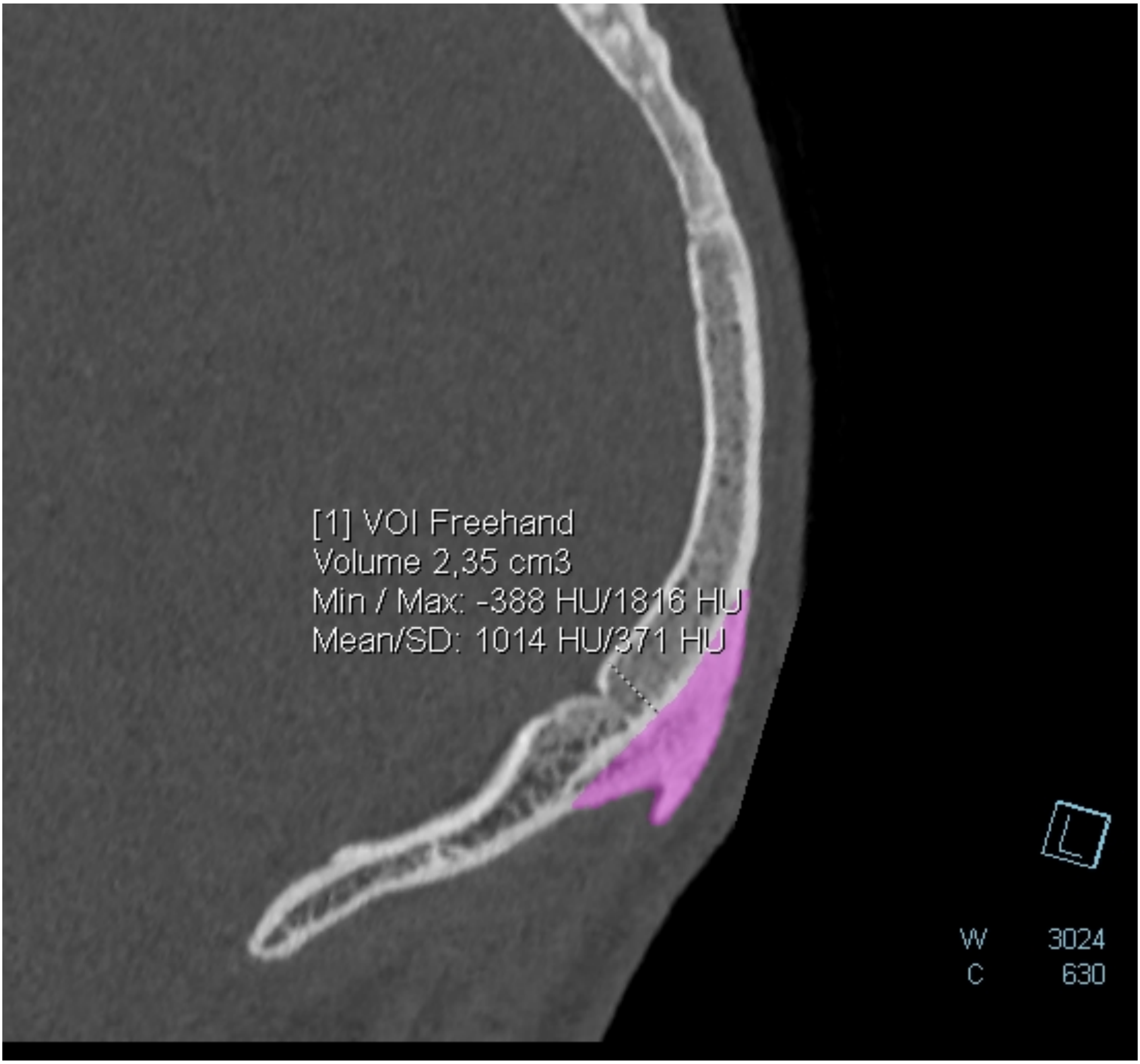
[1] VOI Freehand
Volume 2,35 cm3
Min / Max: -388 HU/1816 HU
Mean/SD: 1014 HU/371 HU
W 3024
C 630

19m80

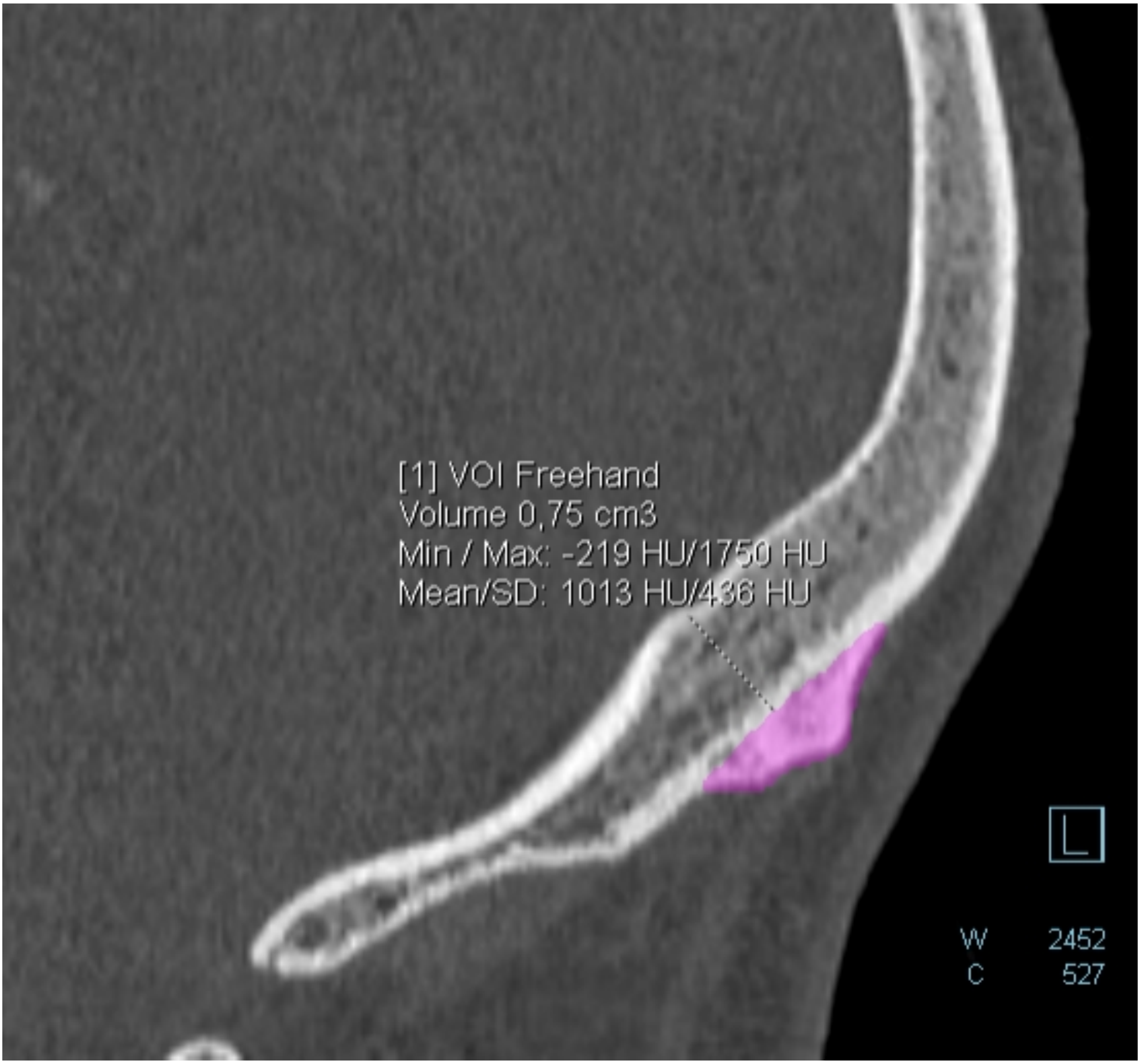
[1] VOI Freehand
Volume 0,75 cm3
Min / Max: -219 HU/1750 HU
Mean/SD: 1013 HU/436 HU
L
W 2452
C 527

19m81

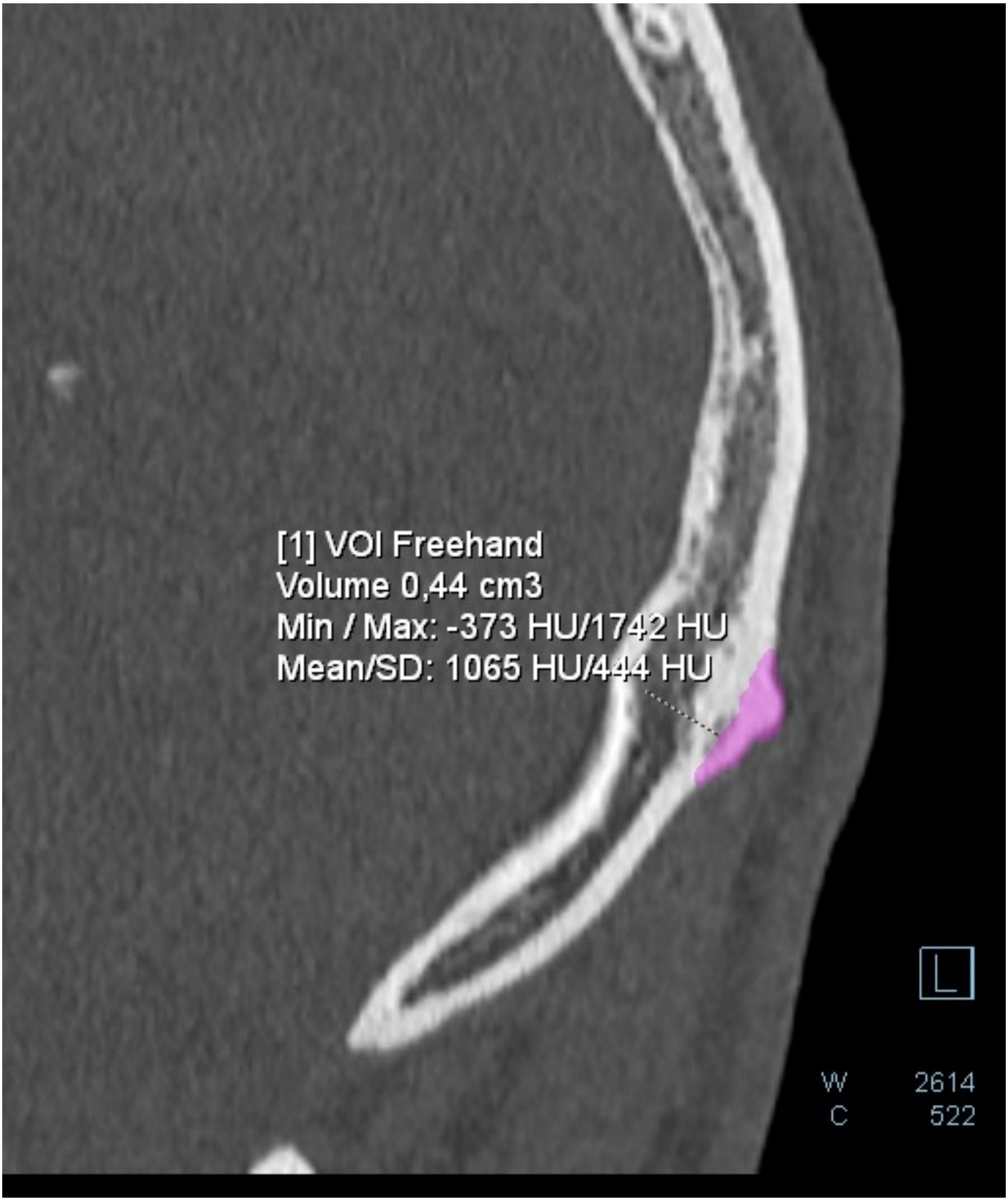
[1] VOI Freehand
Volume 0,44 cm3
Min / Max: -373 HU/1742 HU
Mean/SD: 1065 HU/444 HU
L
W 2614
C 522

19m82

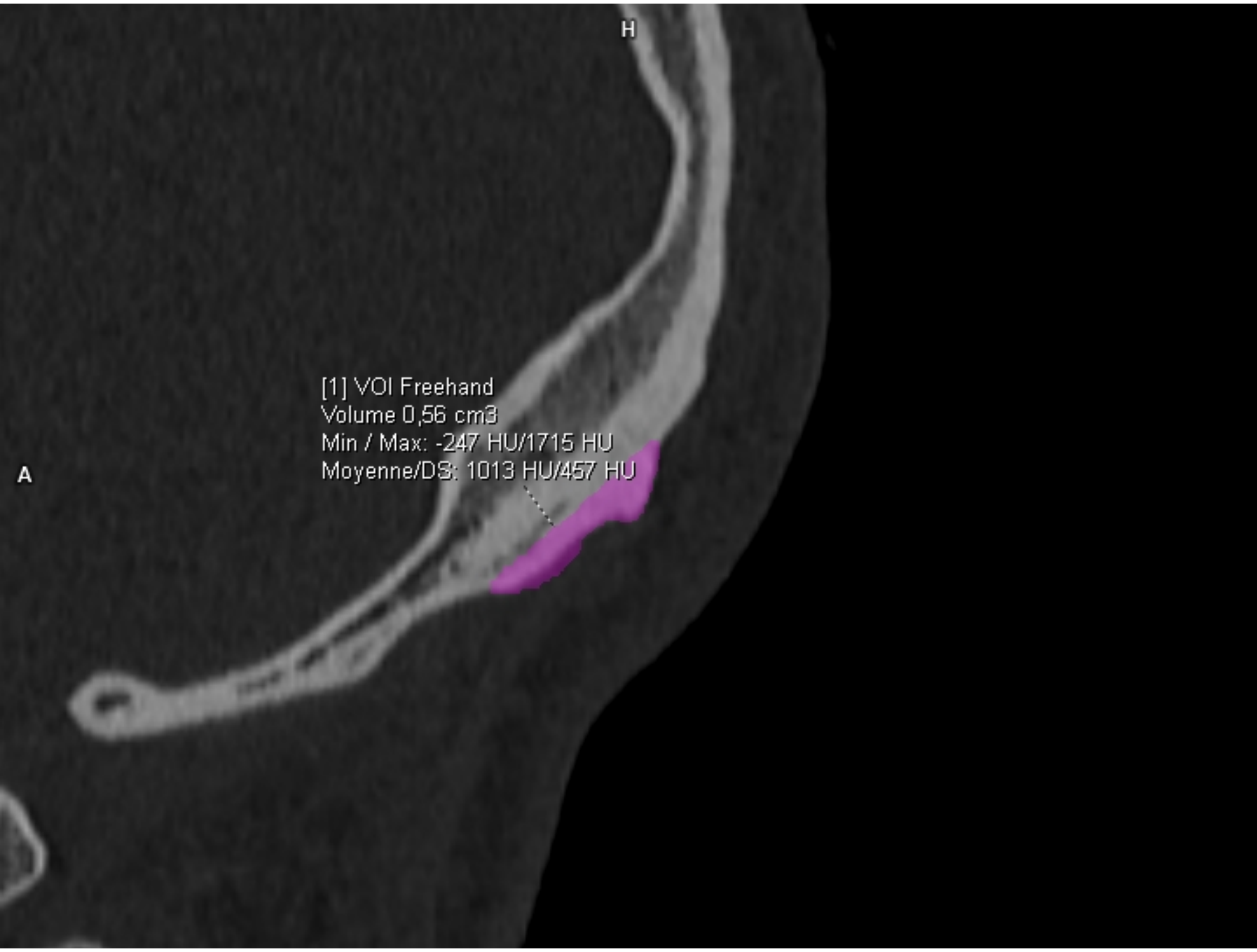
H
A
[1] VOI Freehand
Volume 0,56 cm3
Min / Max: -247 HU/1715 HU
Moyenne/DS: 1013 HU/457 HU

19m83

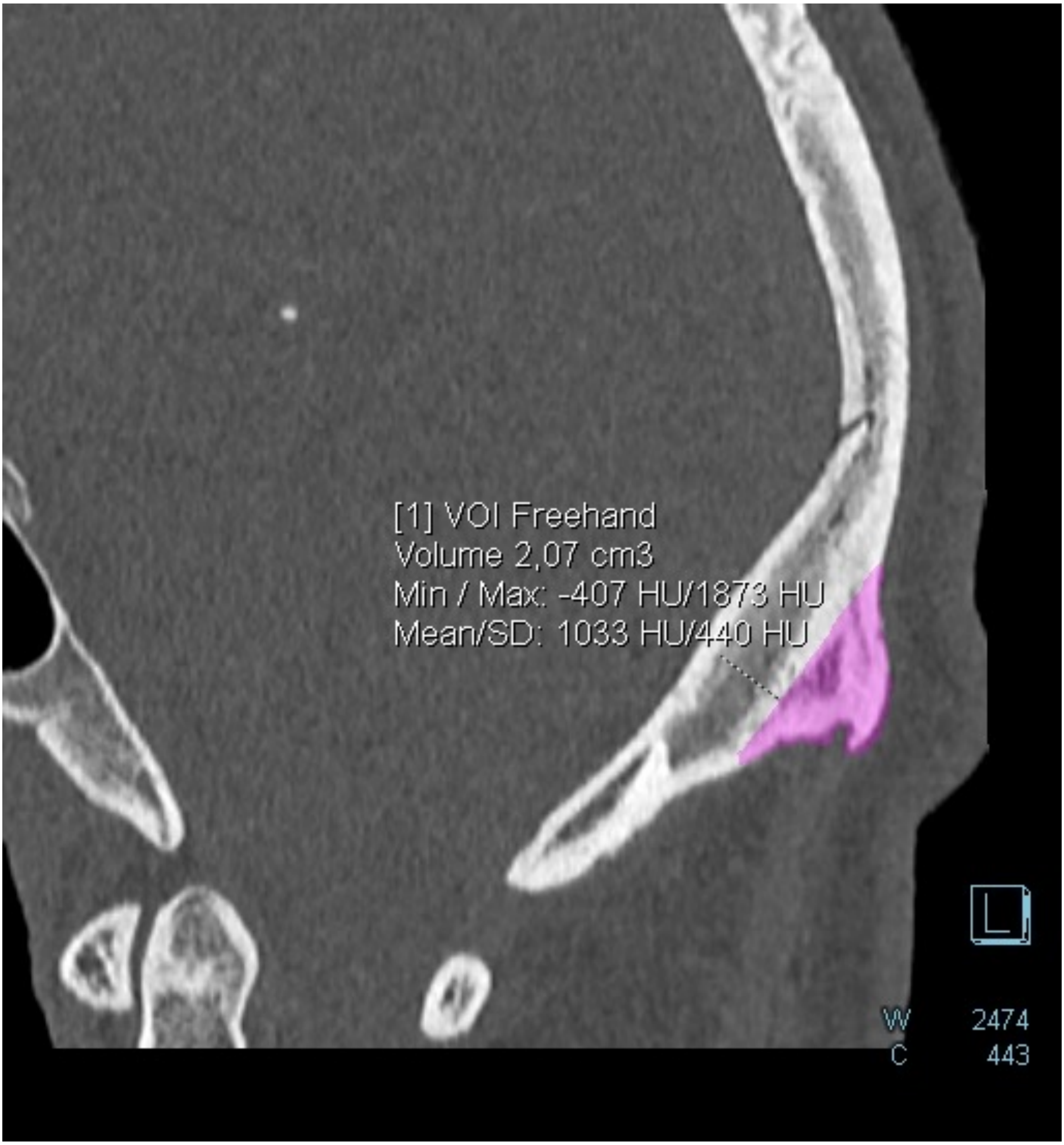
[1] VOI Freehand
Volume 2,07 cm3
Min / Max: -407 HU/1873 HU
Mean/SD: 1033 HU/440 HU
L
W 2474
C 443

19m84

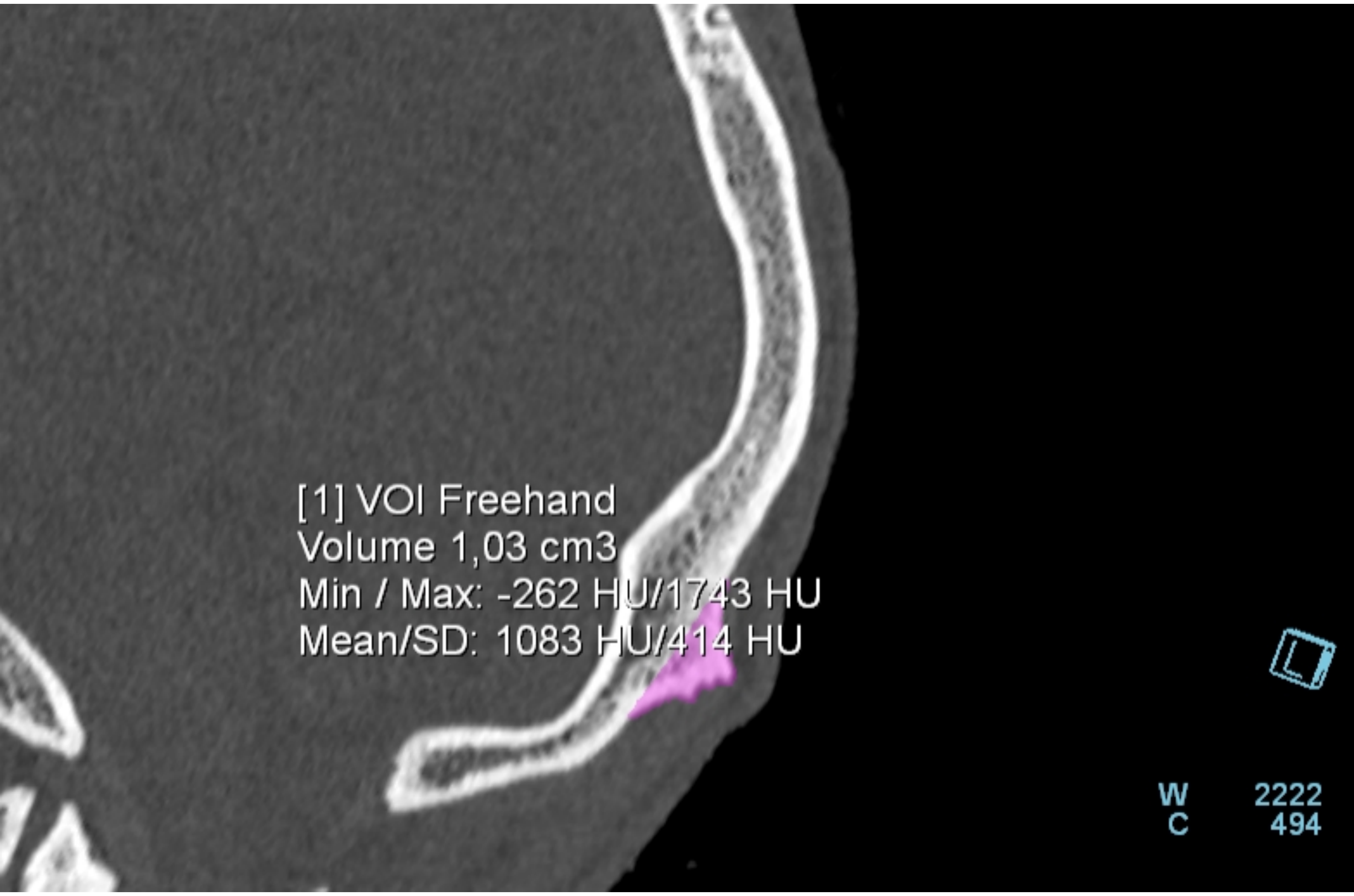
[1] VOI Freehand
Volume 1,03 cm3
Min / Max: -262 HU/1743 HU
Mean/SD: 1083 HU/414 HU
W 2222
C 494

19m85

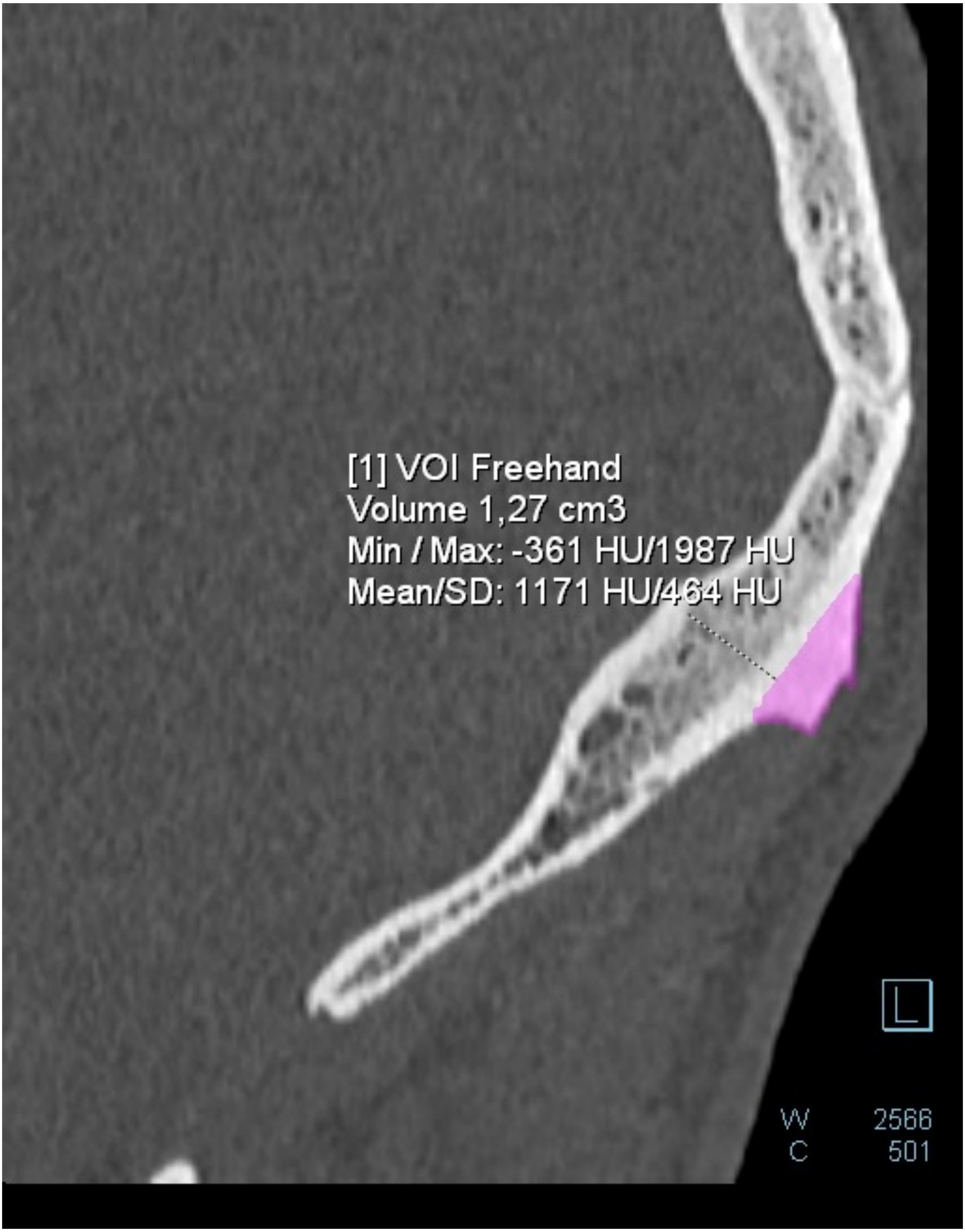
[1] VOI Freehand
Volume 1,27 cm3
Min / Max: -361 HU/1987 HU
Mean/SD: 1171 HU/464 HU
L
W 2566
C 501

19m86

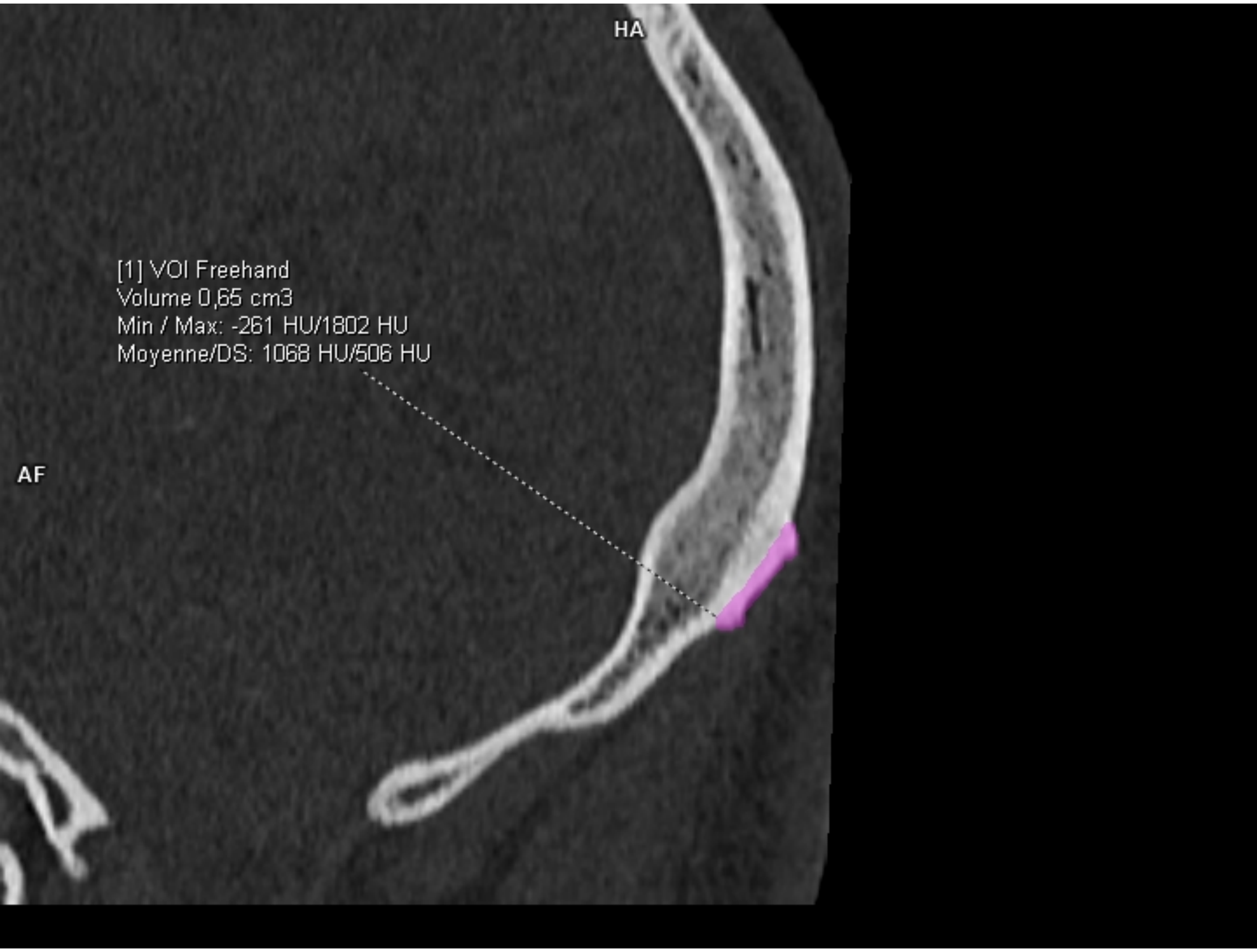
HA
[1] VOI Freehand
Volume 0,65 cm3
Min / Max: -261 HU/1802 HU
Moyenne/DS: 1068 HU/506 HU
AF

19m87

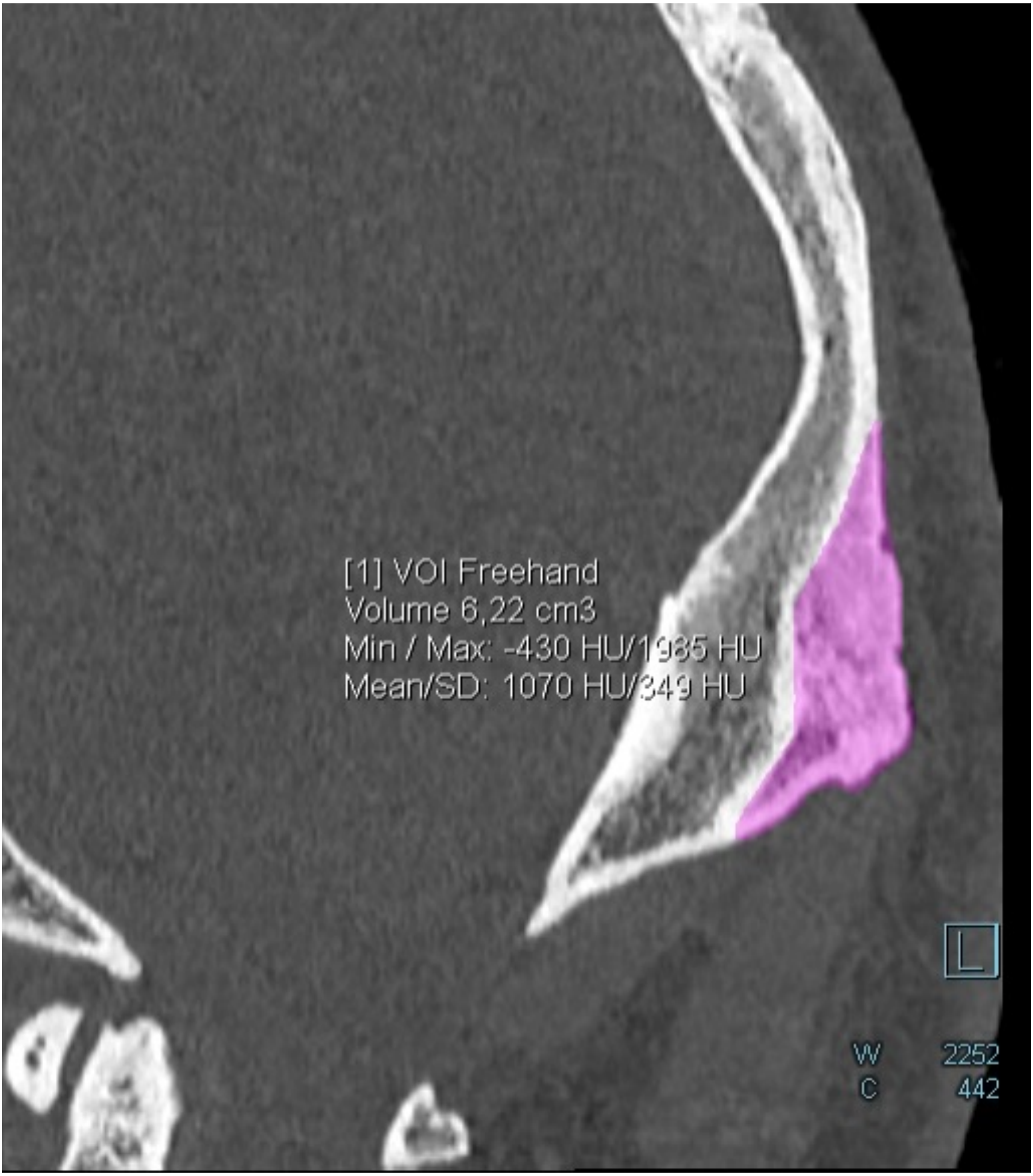
[1] VOI Freehand
Volume 6,22 cm3
Min / Max: -430 HU/1985 HU
Mean/SD: 1070 HU/349 HU
L
W 2252
C 442

19m88

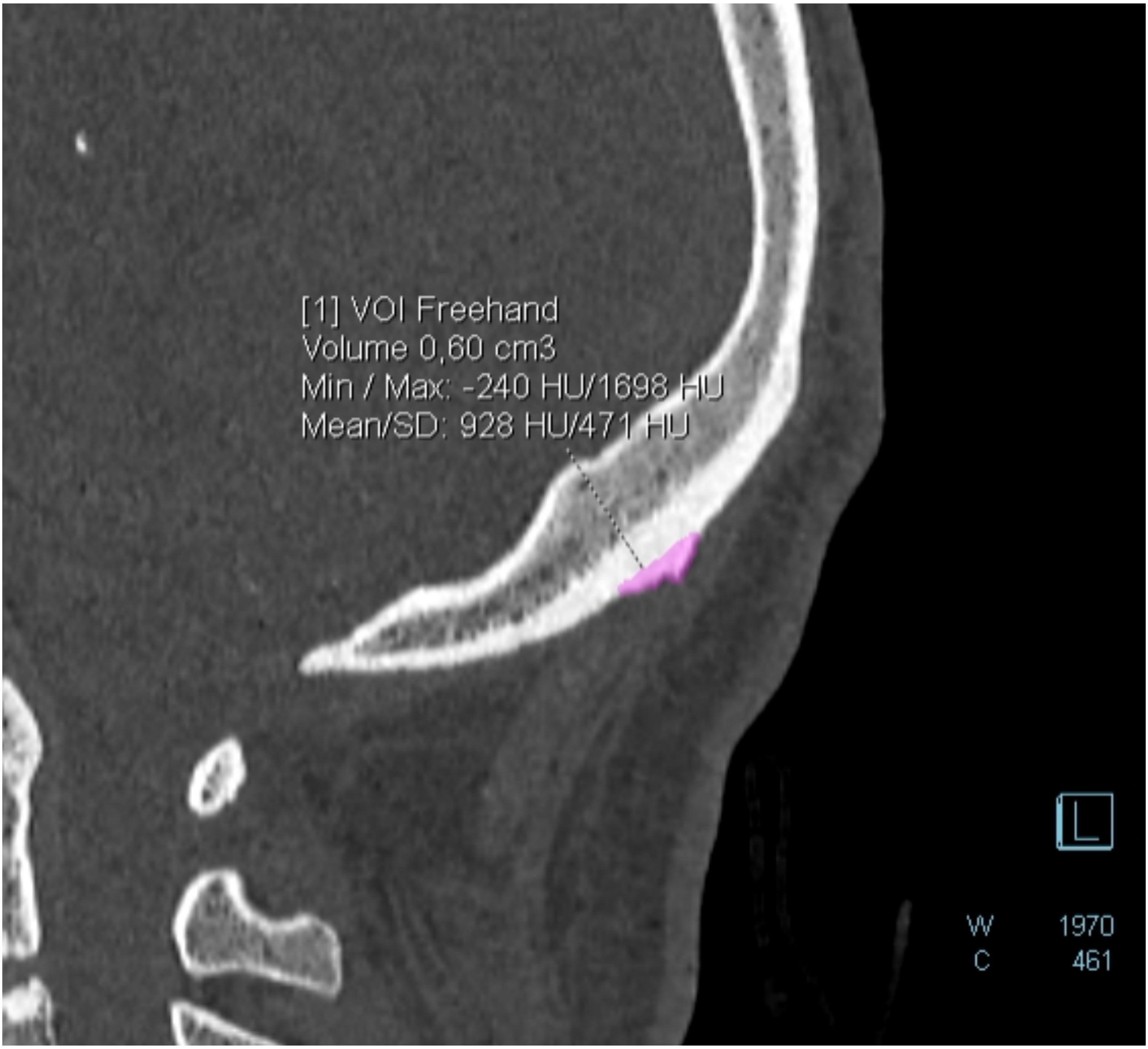
[1] VOI Freehand
Volume 0,60 cm3
Min / Max: -240 HU/1698 HU
Mean/SD: 928 HU/471 HU
L
W 1970
C 461

CM1

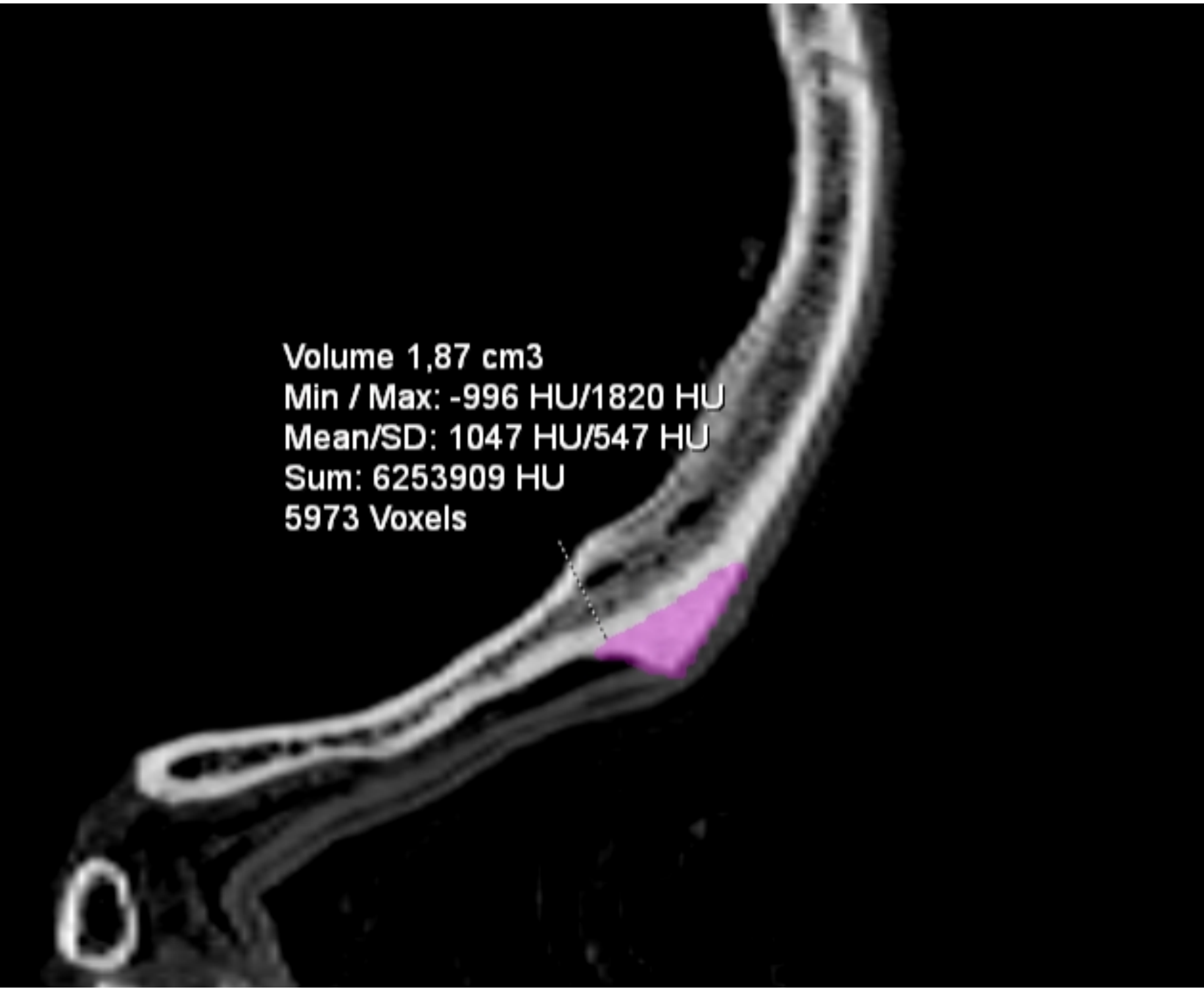
Volume 1,87 cm3
Min / Max: -996 HU/1820 HU
Mean/SD: 1047 HU/547 HU
Sum: 6253909 HU
5973 Voxels

CR1

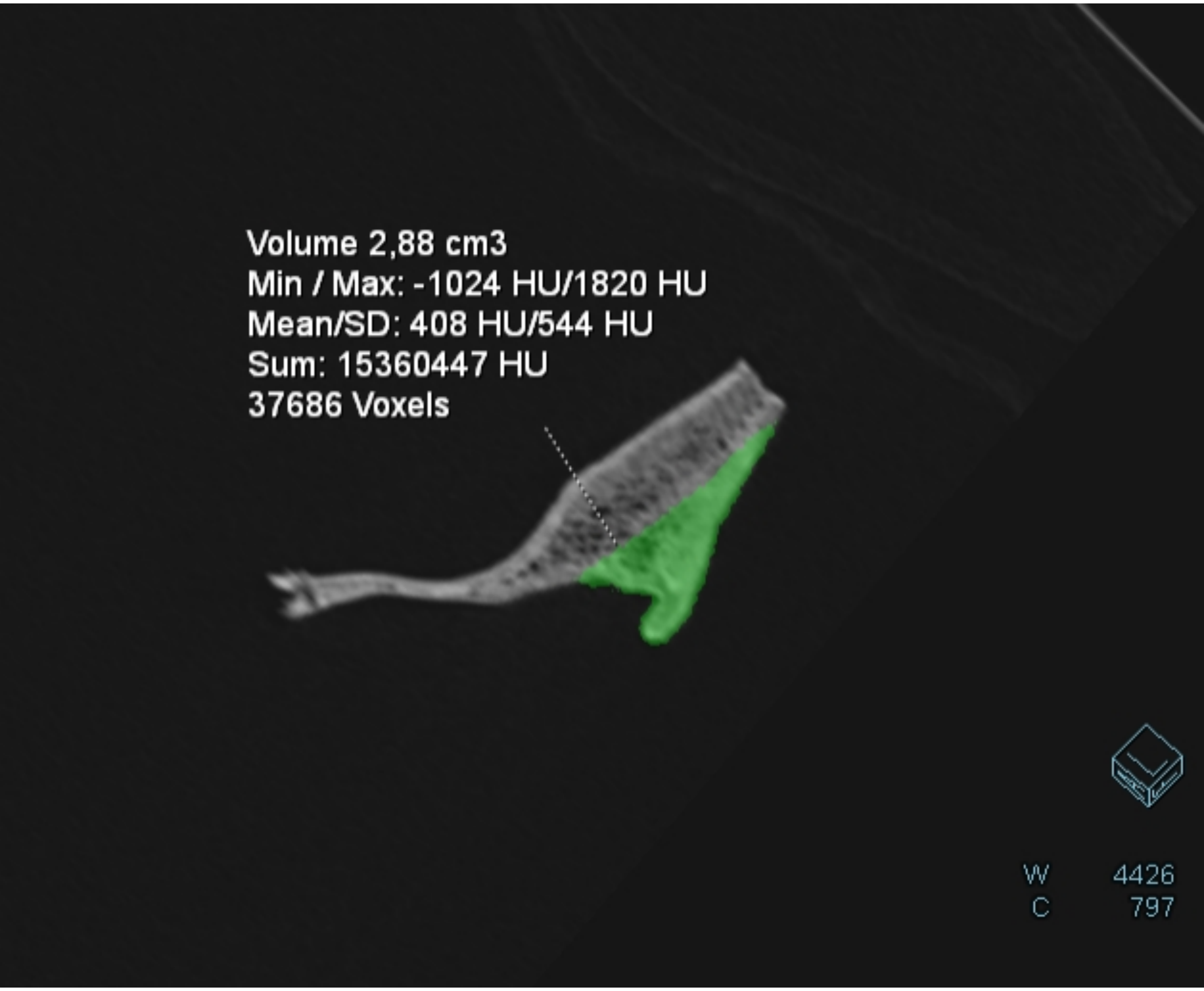

Volume 2,88 cm3
Min / Max: -1024 HU/1820 HU
Mean/SD: 408 HU/544 HU
Sum: 15360447 HU
37686 Voxels
W 4426
C 797
